# The effect of physical activity on health-related outcomes in children and adolescents with cancer: a systematic review and meta-analysis

**DOI:** 10.3389/fonc.2026.1773060

**Published:** 2026-03-31

**Authors:** Hehe Song, Meng Zhang, Yu Fan, Xu Jiang, Wenjie Huang, Zhide Liang, Chuanzhi Wang, Shudong Tian, Changshuang He, Huiwu Zuo, Peng Chen, Congxian Fan, Tao Liu

**Affiliations:** 1 College of Sports and Health Sciences, Xi’an Physical Education University, Xi’an, China; 2 School of Exercise and Health, Shanghai University of Sport, Shanghai, China; 3 Key lab of Sports Technology Analysis and Skill Assessment General Administration of Sport, Xi’an Physical Education University, Xi’an, China; 4 Department of Traditional Chinese Medicine Health, Zaozhuang Vocational College of Appled Techology, Zaozhuang, China; 5 Faculty of Health Sciences and Sports, Macao Polytechnic University, Macao, Macao SAR, China; 6 National Center for Translational Medicine, Shanghai Jiao Tong University, Shanghai, China; 7 Exercise Translational Medicine Center, Institute of Translational Medicine, Shanghai Jiao Tong University, Shanghai, China; 8 Lab of Regenerative Medicine in Sports Science, School of Physical Education and Sports Science, South China Normal University, Guangzhou, China; 9 School of Physical Education, Shandong University, Jinan, China; 10 College of Physical Education and Health, Anhui University Of Applied Technology, Hefei, China

**Keywords:** children and adolescents, oncology, physical activity, psychology, wellness

## Abstract

**Background:**

Although treatment-related adverse effects in children and adolescents with cancer are likely multifactorial, insufficient physical activity (PA) is a major contributor. However, evidence on the association between PA and adverse effects in these population remains limited. We aimed to estimate the effects of PA on health-related outcomes (HrO).

**Methods:**

We searched nine databases from inception to August 5, 2025, for randomized controlled trials evaluating the effects of PA on HrO in children and adolescents with cancer. Data extraction followed the Preferred Reporting Items for Systematic Reviews and Meta-Analyses reporting guideline. Independent reviewers extracted study data, and a random-effects model was used to pool results. Two reviewers independently extracted data, assessed the risk of bias, and evaluated the quality of evidence. The primary outcomes included quality of life (QoL), exercise capacity, and cognitive function indicators, while secondary outcomes encompassed social function and body composition indicators, as well as PA intervention acceptability and safety.

**Results:**

The search identified 3,037 studies, only 24 met the eligibility criteria, including nine articles on children, five on adolescents, and ten on both groups. For primary outcomes, PA significantly improved fatigue (SMD = 0.66), muscle strength (SMD = 1.77), trunk muscle strength (SMD = 4.20), PA behaviour (SMD = 1.04), PA levels (SMD = 0.97), six-minute walk test (SMD = 0.62), cognitive function (SMD = 0.32), and executive function (SMD = 0.47); however, PA had no significant effect on QoL, the QoL scale, upper and lower body muscle strength, balance, flexibility, athletic performance, peak oxygen uptake, and depressive symptoms. Regarding secondary outcomes, PA significantly improved social function (SMD = 0.23) but had no significant effect on bone density, BMI, fat percentage, NK cells, or inflammatory factors. Additionally, moderator analysis showed that PA conditions and participant traits significantly affected the outcomes.

**Conclusions:**

In children and adolescents with cancer, PA can improve HrO, ultimately helping to reduce the disease burden.

**Systematic Review Registration:**

https://www.crd.york.ac.uk/PROSPERO/view/CRD420251018626, identifier CRD420251018626.

## 1 Introduction

In the realm of children and adolescent healthcare, cancer is a formidable adversary (1). Despite the decrease in the incidence of cancer among children and adolescents and a significant increase in 5-year survival rates in recent years, cancer therapies and their adverse events are global challenges (1, 2). Unlike in adults, the low specificity of curative treatments for children and adolescents often leads to long-term and delayed effects due to these treatments impact normal, healthy tissues (3). Compared with their peers without cancer, children and adolescent patients face an increased risk of impaired quality of life (QoL), reduced exercise capacity, and cognitive dysfunction (4). These enduring health deficits not only interfere with daily functioning but also contribute to a substantially higher prevalence of chronic conditions in this population (5, 6). Therefore, identifying strategies to mitigate the negative consequences of cancer therapy in children and adolescents is essential.

Physical activity (PA), defined as any bodily movement generated by skeletal muscles that results in energy expenditure, and exercise—a planned, structured, and repetitive subset of PA aimed at maintaining or improving physical fitness and health—are two closely related yet distinct strategies that are important in cancer care (7). In this meta-analysis, we use the term PA to broadly refer to structured or incidental bodily movements involving energy expenditure (8). Substantial evidence and clinical guidelines support the role of PA in attenuating treatment-related adverse effects (AEs) and improving clinical and patient-reported outcomes in adult oncology populations (9–12). However, these findings cannot be directly extrapolated to children and adolescents (13). This population faces unique challenges, including a higher vulnerability to therapy-induced physical deconditioning and a subsequent vicious cycle of reduced PA, worsened exercise intolerance, and diminished QoL (14–17).

Since the first meta-analysis on this topic was published, several systematic reviews and meta-analyses have assessed the effects of PA interventions on specific health outcomes, including fatigue and cardiopulmonary function, in children and adolescents with cancer (18–24). For instance, Morales et al. (2018) performed a systematic review of eight studies and a meta-analysis of three studies and demonstrated that PA interventions were safe for childhood cancer survivors and did not increase the risk of mortality, recurrence, or other AEs in these population, even during intensive treatment. Moreover, these interventions improved physical function, body composition, PA levels, and QoL (22). Although these preliminary syntheses indicate that PA is safe and potentially beneficial for young patients with cancer, important evidence gaps persist. First, previous reviews have often been restricted to a single outcome domain (e.g., cognitive (21) or cardiopulmonary function (23)) or a specific patient subgroup (e.g., survivors post-treatment (24)), resulting in a fragmented evidence base. Second, and more importantly, comprehensive meta-analytic data quantifying the impact of PA interventions on treatment-related AEs—a central concern for both clinicians and families during active therapy—are lacking. Therefore, this study aims to address these gaps by providing a comprehensive and up-to-date synthesis of randomized controlled trials (RCTs) evidence. We evaluate the effects of PA interventions on a broad range of health-related outcomes (HrO) in children and adolescents across the cancer continuum, including those receiving active treatment. Through this comprehensive synthesis, the review aims to generate more robust, safety-focused evidence to support clinical implementation and guide the development of tailored rehabilitation guidelines for this vulnerable population.

## 2 Methods

### 2.1 Search strategy and selection criteria

This study adhered to the Cochrane Handbook for Systematic Reviews of Interventions and the Preferred Reporting Items for Systematic Reviews and Meta-Analyses Statement (25). RCTs on PA interventions for children and adolescents with cancer were identified by searching MEDLINE, Embase, Web of Science, Cochrane Library, CINAHL Plus with Full Text, APA PsycINFO, SPORTDiscus, Educational Resource Information Center, and Scopus from inception to 26 October, 2025, without language restrictions. An experienced medical librarian reviewed and optimised the search terms for each database. The search strategies are detailed in the (**Supplementary Files 1**). Additional eligible studies were identified by reviewing the references of the included studies and systematic reviews. We included randomized controlled trials investigating the effects of exercise interventions on HrO in children and adolescents aged 6–18 years with cancer. Studies with an intervention period of less than 3 weeks were excluded because cancer is a long-term chronic condition, and physical activity intervention itself was a process that required sustained duration to elicit meaningful physiological and psychological adaptations. A 3-week threshold was therefore established to ensure that the assessed effects of PA were attributable to a substantive intervention period. In addition, short interventions may limit the analysis of clinically meaningful changes and are less likely to predict long-term effects, which are crucial for meeting the ongoing needs of cancer patients and their families. Studies that combined PA with cognitive behavioural therapy, as well as those incorporating additional interventions such as dietary modifications, were excluded to isolate the effects of PA alone, enabling an accurate assessment of its direct effect on psychological and physical function.

To meet the inclusion criteria, intervention groups must be compared with control groups that receive no intervention, a placebo, or a low-intensity intervention serving as a control therapy; this control therapy is not expected to improve physical function but may benefit participants. We assessed the effects of PA on HrO as well as intervention acceptability and safety. HrO included QoL, exercise capacity, cognitive function, social function and body composition indicators. Safety was assessed by identifying adverse events. Acceptability was evaluated by measuring the risk ratio (RR), which was the percentage of patients who withdrew from the study for any reason.

EndNote X9 (Clarivate, Philadelphia, PA, USA) was used to perform de-duplication and to select studies based on titles and abstracts. Data were extracted using Microsoft Excel. Two researchers (HHS and MZ) independently screened the studies by title and abstract. Data extraction and bias risk assessment were performed using the Cochrane Collaboration’s Risk of Bias tool for RCTs (RoB 2) (26). Disagreements in bias risk assessment were resolved by consensus or by a third reviewer. The study protocol was registered with PROSPERO (Registration No. CRD420251018626).

### 2.2 Data analysis

For studies that met the inclusion criteria, we calculated the means and standard deviations (SDs) of baseline and endpoint measurements in the intervention and control groups. Standard errors were converted to SDs (27). When such data were missing, the means and SDs were calculated from the sample size, medians, interquartile ranges (IQRs), and minimum and maximum values (28). A correlation coefficient of 0.5 was used as recommended by RoB 2 (26). The weight of each study was determined based on the inverse of its variance. Since health outcomes are continuous variables, we used a random-effects model to calculate the effect size of each intervention. Data were presented as standardised mean differences (SMDs) and 95% confidence intervals (CIs) (29, 30).

To correct for small sample bias in effect size estimates, SMDs were adjusted using Hedges’ g. The estimated between-study variance, τ², was calculated using the restricted maximum likelihood method (31). The Knapp–Hartung adjustment was used to account for uncertainties in between-study heterogeneity and reduce the risk of false-positive results (32). SMDs were classified as small (~0.2), moderate (~0.5), and large (~0.8), as described previously (33). We evaluated heterogeneity using Cochran’s Q statistic, Higgins–Thompson I², prediction intervals, and average dispersion in effect sizes τ² (34–36). Publication bias was assessed using funnel plots and Egger’s test (37, 38). The trim-and-fill method was used to assess the effect of publication bias on the interpretation of the results (39).

A sensitivity analysis was performed using the leave-one-out method (40). Subgroup analysis was conducted considering covariates that might influence the intervention effects. A random-effects meta-regression analysis was performed to identify the sources of heterogeneity and examine how continuous covariates moderate intervention outcomes. Acceptability was calculated using a random-effects model rather than a fixed-effects model because of heterogeneity across studies and their small number (41). To exclude zero-event trials without using correction methods, we employed the Mantel–Haenszel method to ensure the robustness and validity of the analysis (42). Data were analysed using R software version 4.3.3 (R Foundation for Statistical Computing, Vienna, Austria) (43). A p-value of less than 0·05 was considered statistically significant.

The quality of evidence for each outcome was assessed using the Grading of Recommendations Assessment, Development and Evaluation (GRADE) method (44). The reliability of the evidence was rated as high, moderate, low, or very low based on five criteria: risk of bias in studies, inconsistency (I² >90%), indirectness, imprecision, and publication bias (Appendix pages 13 and 14) (45, 46). The quality of evidence was further evaluated according to the standards of the American College of Sports Medicine (ACSM) (13).

## 3 Results

### 3.1 Study characteristics

**Figure 1:**
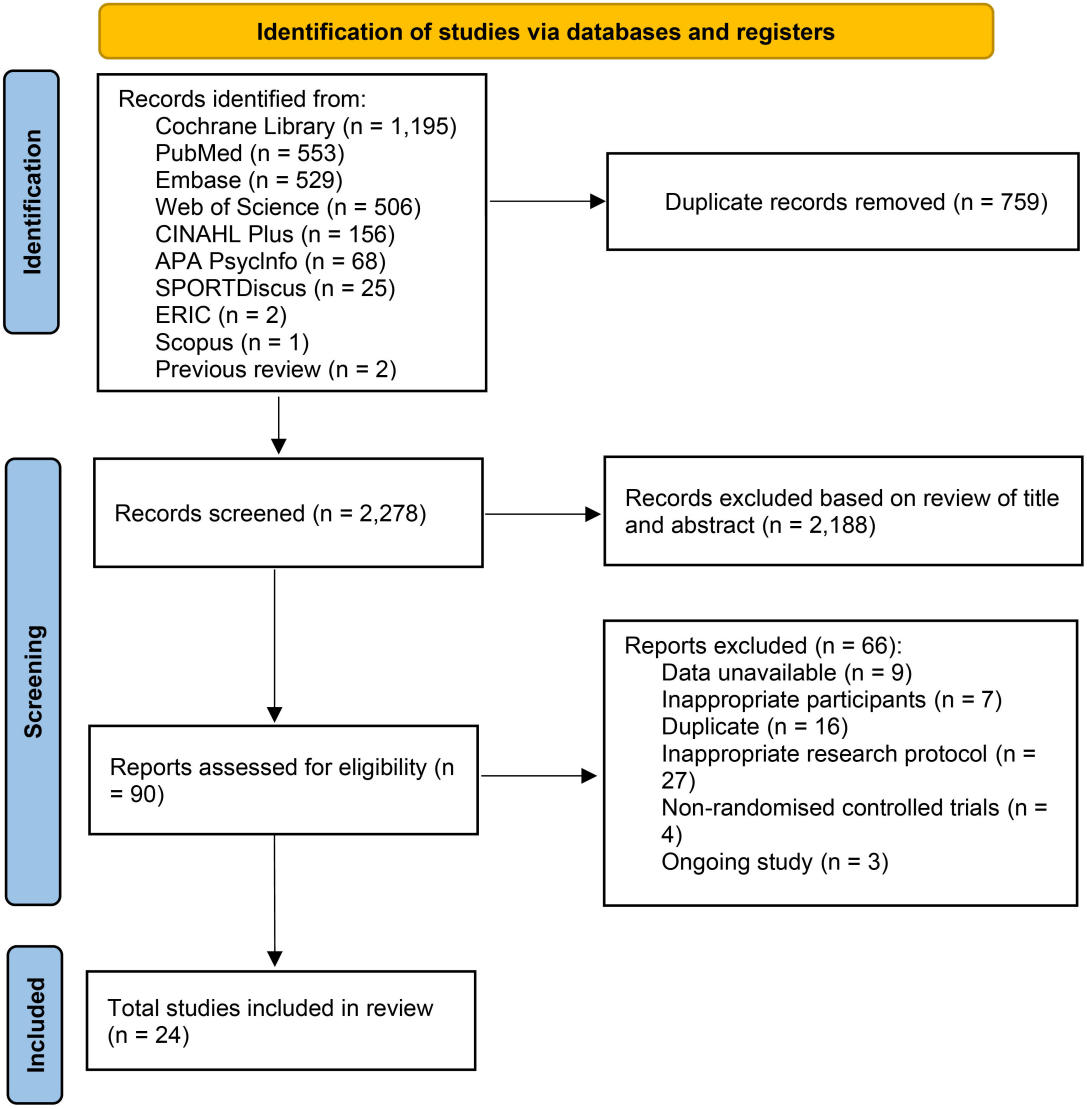
PRISMA flow diagram.

A search of nine databases and two previous studies identified 3,037 studies. After removing 759 duplicates, 2,278 studies were screened by title and abstract, and 90 were screened by full text. Of these, 24 met the eligibility criteria (**Figure 1**). Excluded studies and the reasons for their exclusion were listed in the **Supplementary Files 2**. The characteristics of the selected RCTs were shown in **Table 1**. The RCTs included 1,074 children and adolescents with cancer (607 males and 467 females). Nine studies focused solely on children (median age = 9.59 years, IQR = 9.04–10.5), five on adolescents (median age = 12.6 years, IQR = 12.6–13.25), and ten on both groups (median age = 12.12 years, IQR = 11.75–12.65). Sixteen RCTs included participants in the acute treatment phase, and eight included cancer survivors. Most studies used standard care, but its definition was broad (**Supplementary Files 3**). Fifteen RCTs had a low risk of bias, and nine had a higher risk (**Supplementary Files 4**).

**Table 1:** Characteristics of included studies.

Study	Population		Information of disease			Intervention			
	Sample sizes (female %); age (mean ± SD or range)	Country	Types	Treatment stage	Medicine (Y/N)	Length (weeks)	Sessions (per week)	Sessions duration (Min)	Exercise intensity
Masoud (59)	EG: 22 (45.45), 9.04 ± 2.35; CG: 23 (43.48), 9.04 ± 2.47	Saudi Arabia	ALL	Under	Y	3	2	60	Light ~ Moderate
Braam (60)	EG: 22 (45.45), 13.40 ± 3.10; CG: 33 (55.00), 13.10 ± 3.10	Netherlands	Multiform	Under	Y	12	2	45	Moderate
Saultier (61)	EG: 37 (44.00), 11.40 ± 0.60; CG: 25 (41.00), 11.20 ± 0.60	France	Multiform	Under	Y	24	NA	90	Light ~ Moderate
Ruble (62)	EG: 6 (44.44), 10.30, 8.00 ~ 12.00; CG: 7 (0.00), 9.60, 8.00 ~ 12.00	American	Multiform	Post	N	24	NA	150	Light ~ Moderate
Tanriverdi (63)	EG: 11 (62.50), 12.90 ± 5.80; CG: 13 (62.50), 12.90 ± 5.80	Turkey	ALL	Post	N	12	2	45	Moderate
Fiuza-Luces (64)	EG: 24 (29.00), 10.00 ± 1.00; CG: 25 (28.00), 11.00 ± 1.00	Spanish	EST	Under	Y	20	3	60	Light ~ Moderate
Caru (65)	EG: 20 (30.00), 13.70 ± 2.40; CG: 20 (40.00), 13.60 ± 2.20	American	Multiform	Post	NA	12	7	60	Moderate ~ Vigorous
Manchola-González (66)	EG: 7 (NA), 11.00 ± 3.70; CG: 12 (NA), 11.80 ± 4.40	Spanish	ALL	Post	NA	16	3	30	Light ~ Vigorous
Lam (67)	EG: 37 (45.90), 12.80 ± 2.50; CG: 33 (54.50), 12.50 ± 2.50	China	Multiform	Under	Y	24	1-2	60	Light ~ Moderate
Tanir (68)	EG: 19 (21.10), 10.21 ± 1.51; CG: 21 (57.10), 10.72 ± 1.51	Turkey	ALL	Post	NA	12	7	60	Moderate ~ Vigorous
Stössel (69)	EG: 13 (37.50), 10.60 ± 5.20; CG: 15 (41.18), 11.40 ± 4.30	Germany	Multiform	Under	Y	6-8	3	45-60	Light ~ Moderate
Hartman (70)	EG: 20 (44.00), 5.30, 5.00 ~ 15.00; CG: 21 (38.00), 6.20, 6.00 ~ 17.00	Netherlands	ALL	Under	Y	96	7	NA	Light
Müller (71)	EG: 10 (60.00), 15.20 ± 2.00; CG: 11 (54.55), 12.20 ± 2.60	Germany	Multiform	Under	Y	24	5	15-45	Moderate ~ Vigorous
Marchese (72)	EG: 13 (38.46), 7.60, 4.30 ~ 10.60; CG: 15 (20.00), 8.60, 5.10 ~ 15.80	American	ALL	Under	Y	12	7	NA	Light
Yeh (73)	EG: 12 (50.00), 11.01 ± 3.56; CG: 10 (40.00), 12.48 ± 3.86	American	ALL	Under	Y	6	3	30	Light
Chamorro-Viña (74)	EG: 3 (66.67), 12.67 ± 3.30; CG: 3 (100.00), 13.33 ± 4.50	Canada	HSCT	Under	Y	10	3	50-60	Light ~ Moderate
Fiuza-Luces (75)	EG: 9 (22.22), 11.00 ± 4.00; CG: 11 (45.45), 12.00 ± 4.00	Spain	EST	Under	Y	17	3	60-70	Light ~ Moderate
Li (76)	EG: 103 (46.60), 12.80 ± 2.60; CG: 89 (46.20), 12.40 ± 2.6	China	Multiform	Post	N	NA	NA	NA	NA
Senn-Malashonak (77)	EG: 28 (28.57), 11.00, 5.00 ~ 17.00; CG: 29 (34.29), 12.00, 6.00 ~ 18.00	Germany	HSCT	Under	Y	NA	NA	NA	NA
Elnaggar (78)	EG: 29 (34.50), 13.31 ± 2.61; CG: 29 (44.80), 14.21 ± 2.35	Saudi Arabia	ALL	Post	N	12	3	25-50	Light ~ Moderate
Khodashenas (79)	EG: 10 (40.00), 10.10, 5.00 ~ 12.00; CG: 10 (40.00), 8.80, 5.00 ~ 12.00	Iran	ALL	Under	Y	12	3	60	Light ~ Moderate
Waked (82)	EG: 23 (65.20), 9.26 ± 2.39; CG: 23 (21.70), 9.91 ± 2.09	Saudi Arabia	ALL	Under	Y	72	1-2	30-45	Light
Şahin (80)	EG: 52 (46.20), 12.35 ± 3.43; CG: 41 (48.80), 11.89 ± 3.56	Turkey	Multiform	Under	Y	4	5	40-60	Light
Dubnov-Raz (81)	EG: 10 (60.00), 11.10, 7.80 ~ 13.80; CG: 11 (50.00), 11.80, 9.00 ~ 12.80	Israel	Multiform	Post	N	24	3	60	Light ~ Vigorous

ALL, acute lymphoblastic leukaemia; EST, extracranial solid tumour; HSCT, hematopoietic stem cell transplantation; N, no; NA, not available; Post, post treatment; Under, under treatment; Y, yes.

### 3.2 Meta-analysis results

**Figure 2:**
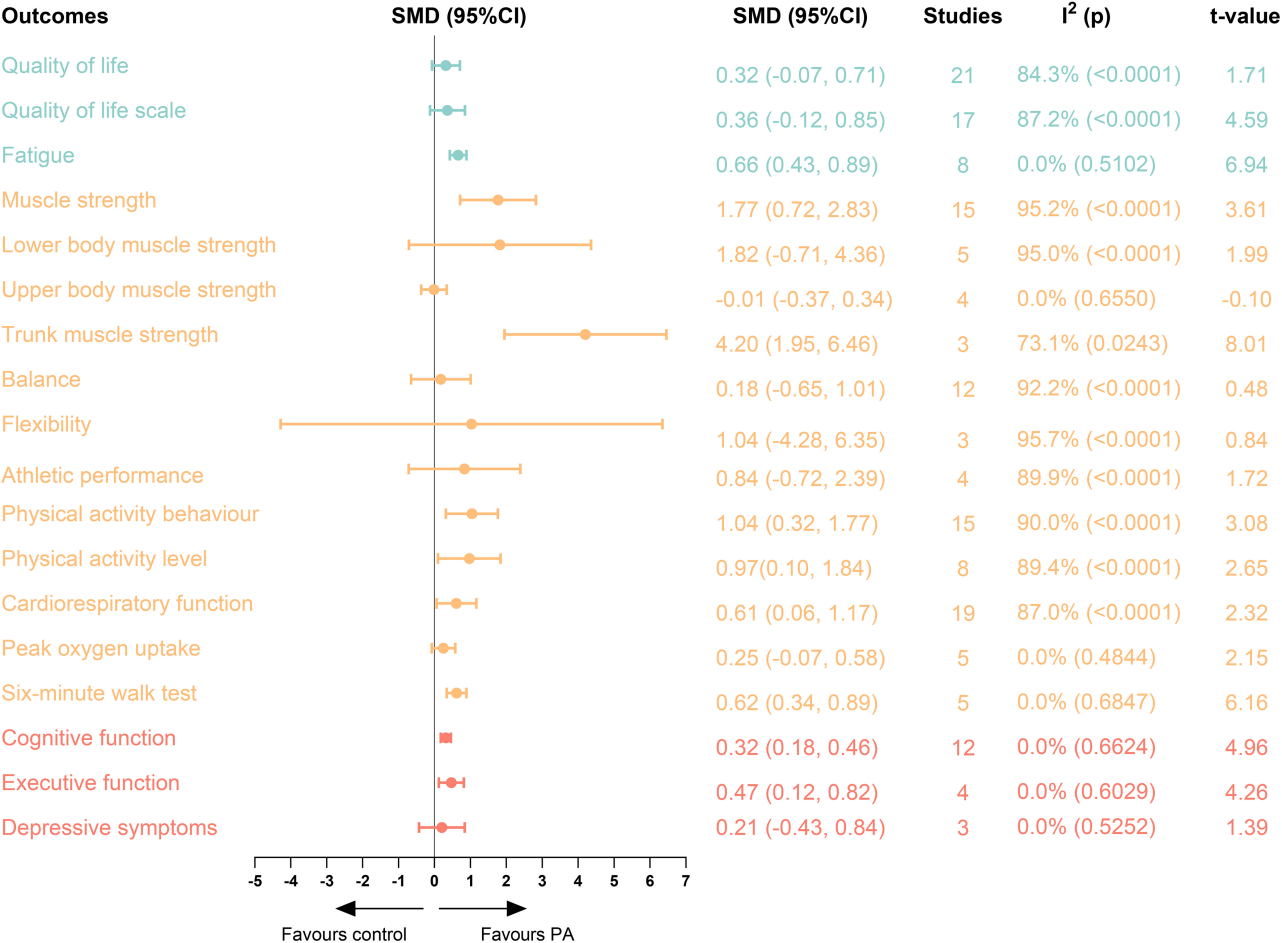
Primary outcomes of included studies. Blue:quality of life indicators, yellow: exercise capacity indicators, red: cognitive function indicators. SMD, standardised mean differences, CI, confidence intervals, PA, physical activity.

For primary outcomes, PA interventions significantly improved fatigue (SMD = 0.66; 95% CI = 0.43 to 0.89), muscle strength (SMD = 1.77; 95% CI = 0.72 to 2.83), trunk muscle strength (SMD = 4.20; 95% CI = 1.95 to 6.46), PA behaviour (SMD = 1.04; 95% CI = 0.32 to 1.77), PA levels (SMD = 0.97; 95% CI = 0.10 to 1.84), cardiorespiratory function (SMD = 0.61; 95% CI = 0.06 to 1.17), six-minute walk test (SMD = 0.62; 95% CI = 0.34 to 0.89), cognitive function (SMD = 0.32; 95% CI = 0.18 to 0.46), and executive function (SMD = 0.47; 95% CI = 0.12 to 0.82). Furthermore, the analysis indicated no statistically significant effects for QoL (SMD = 0.32; 95% CI = -0.07 to 0.71), QoL scale (SMD = 0.36; 95% CI = -0.12 to 0.85), lower body muscle strength (SMD = 1.82; 95% CI = -0.71 to 4.36), upper body muscle strength (SMD = -0.01; 95% CI = -0.37 to 0.34), balance (SMD = 0.18; 95% CI = -0.65 to 1.01), flexibility (SMD = 1.04; 95% CI = -4.28 to 6.35), athletic performance (SMD = 0.84; 95% CI = -0.72 to 2.39), peak oxygen uptake (SMD = 0.25; 95% CI = -0.07 to 0.58), depressive symptoms (SMD = 0.21; 95% CI = -0.43 to 0.84) (**Figure 2**).

The random-effects regression model showed that with regard QoL indicators, differences in length, sessions duration, and disease types all significantly affect the effect of PA. As for exercise capacity indicators, covariates such as age, treatment stage, region, medication, as well as length, intensity, and sessions duration of PA all had significantly effect. Region and length also affected the effect of PA on cognitive function indicators. Furthermore, the results of the subgroup analysis indicated that age, treatment stage, medication, and conditions of PA intervention were significantly associated with improvements in the primary outcomes. Detailed information regarding the regression and subgroup analyses was provided in **Supplementary Files 12 and 13**.

**Figure 3:**
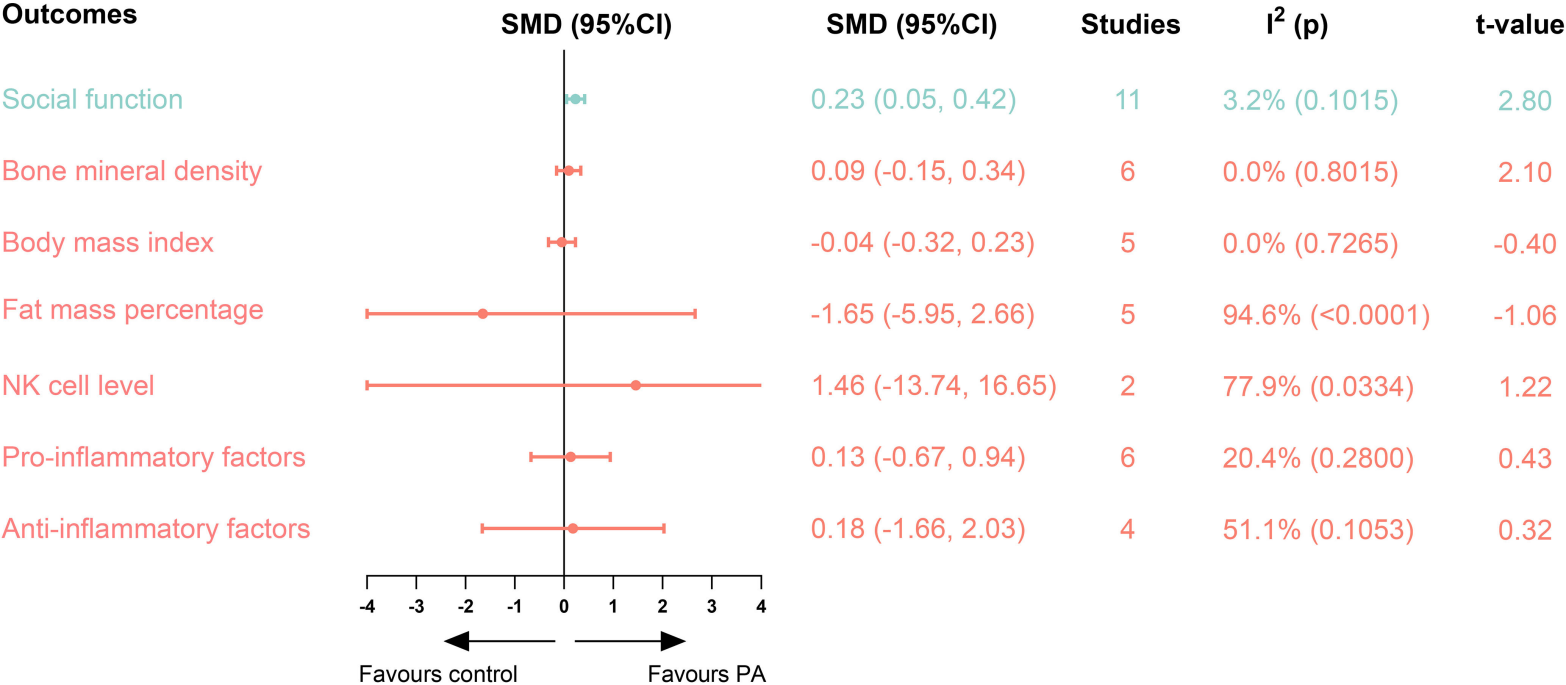
Secondary outcomes of included studies. Blue:social function indicators, red: body composition indicators. SMD, standardised mean differences, CI, confidence intervals, PA, physical activity.

For secondary outcomes, PA was found to significantly improve social functioning (SMD = 0.23; 95% CI = 0.05 to 0.42), while no significant effects were observed for other outcomes (**Figure 3**). Furthermore, moderator analysis demonstrated that conditions of PA intervention and disease type significantly affected the effects of PA. Detailed results and forest plots for regression and subgroup analyses on secondary outcomes were shown in the **Supplementary Files 12 and 13**.

### 3.3 Adverse events and acceptance

One RCT reported minor adverse events such as pain and poor mental status, and four reported serious adverse events, including relapse, severe complications, and death. The acceptance of PA interventions was higher in the experimental group than in the control group, though without statistical significance (RR = 0.79, 95% CI = 0.54 to 1.17).

### 3.4 Sensitivity analysis, publication bias and GRADE

Based on GRADE standards, the overall quality of evidence for outcomes ranged from very low to moderate. The quality of evidence for all outcomes was downgraded by one level because of the risk of bias in allocation concealment in three RCTs (**Supplementary Files 9**). Sensitivity analysis supports the overall results (**Supplementary Files 6**). There was publication bias in the effects of PA on muscle strength, cardiopulmonary function, and pro-inflammatory factors. The effects of PA on cardiopulmonary function and pro-inflammatory factors decreased after correcting for publication bias using the trim and fill method (**Supplementary Files 8**).

## 4 Discussion

### 4.1 Evidence summary

This systematic review and meta-analysis of 24 RCTs involving over 1,000 children and adolescents with cancer provided evidence that PA interventions significantly improve fatigue, muscle strength, trunk muscle strength, PA levels, PA behaviour, cardiopulmonary function, SMWT, cognitive function, executive function, and social function compared to control groups. These effects varied depending on disease types, treatment stage, medication, regional economic conditions, and sessions, sessions duration, and intensity of PA. In turn, gender had no significant effect. These results support the implementation of PA interventions to improve the health of children and adolescents with cancer.

Fatigue is highly prevalent during anticancer treatment, impairs daily functioning, and contributes to sedentary behaviour and reduced PA levels (47, 48). However, standardized PA management guidelines to mitigate treatment−related AEs are lacking in children and adolescents with cancer. A previous study demonstrated that supervised exercises reduced fatigue in these population (24). In our meta-analysis, eight RCTs showed that PA interventions had a moderate to large positive effect on fatigue in these population, which is consistent with previous findings (49). Moreover, lower PA levels in these population are strongly associated with cardiovascular disease, osteoporosis, and all-cause mortality (50). Kim et al. (2025) (51) and Langworthy et al. (2023) (52) both provided robust evidence in support of exercise in paediatric cancer populations: Kim et al. conducted a comprehensive meta-analysis of patients and survivors, demonstrating beneficial effects on cardiopulmonary health and functional recovery, whereas Langworthy et al. focused on adolescents undergoing active treatment and reported significant improvements in PA levels and fatigue. In addition, previous reviews treated outcomes such as QoL, muscle strength, and cardiorespiratory function as single−dimensional. In contrast, our review conceptualised these outcomes as composites of multiple dimensions—for example, cardiorespiratory function was assessed as the combined effect of peak oxygen uptake, respiratory exchange ratio, heart rate, and SMWT. The combination of these outcomes increased between-study heterogeneity but provided a more complete and realistic view of the effects of PA on health. Our findings indicated that PA interventions could effectively improve multidimensional indicators, including cardiopulmonary function, muscle strength, and PA behaviour.

We also sought to elucidate PA dose-response relationships for HrO. The intensity, sessions, sessions duration, and length of PA have distinct effects on HrO. Our results indicated that the PA protocols for improving fatigue, muscle strength, physical activity behaviour, cognitive function, and QoL were usually similar, with low-intensity PA, 1-3 sessions per week, and a sessions duration of 30-60 minutes could yield positive effects. To improve cardiopulmonary function, SMWT and PA levels, it seemed that moderate to vigorous intensity PA, higher sessions (7 sessions per week), and a sessions duration of 30-60 minutes were associated with greater effects. The effect of length was roughly similar across HrO, indicating that long-term PA had yielded relatively favourable health improvement effects in children and adolescents with cancer. Furthermore, in addition to the aforementioned sessions, intensity, sessions duration, and length, the PA types also has a significant effect on the effectiveness of PA interventions (53). Seventeen RCTs had focused on the effects of aerobic exercises or resistance training, four on skill-based exercises (e.g., basketball and badminton), two on PA games, and one on functional exercises. Although the types of exercise were similar between the studies, there was considerable variation in outcome measures, and some outcomes were not evaluated. Combining aerobic exercises with resistance training may yield better outcomes in children and adolescents with cancer (54).

We assessed the effects of PA on HrO according to ACSM standards (at least five RCTs, studies with more than 150 participants, and consistent beneficial effects across studies) (13). Most of the primary outcomes met these standards (**Supplementary Files 11**), which can help inform future clinical guidelines. In addition, there was high between-study heterogeneity in our study, partially explained by regression analysis. High heterogeneity may be due to covariates or measurement errors affecting effect estimates. Outcome assessment methods remain a critical determinant of study validity, irrespective of the overall quality of the intervention design. The included RCTs evaluated body composition, cardiopulmonary function, and muscle strength using objective measures. Furthermore, most indicators of exercise capacity were measured using validated tests, while PA levels were assessed using questionnaires. PA levels were measured using accelerometers in four studies and using questionnaires in five studies. Although the questionnaires were validated against accelerometer data, their accuracy remains relatively low (55). Fatigue and QoL scale also were assessed using questionnaires. Fatigue was measured using the PedsQL™ Multidimensional Fatigue Scale (four studies), the Chinese version of the Fatigue Scale for Children (two studies), or the PROMIS Paediatric Fatigue Scale (one study). However, the single-dimensional nature of the PROMIS Paediatric Fatigue Scale may not fully reflect the complexity of fatigue (56). Most studies (77%) evaluated QoL scale using PedsQL 3.0 and 4.0 (57, 58). Additionally, the interventions received by the control groups varied significantly, increasing the heterogeneity in outcomes. We included 24 RCTs from America, Europe, and Asia, representing various cultural backgrounds.

### 4.2 Limitations

This review has limitations. First, the poor methodological quality of the reviewed RCTs. Second, included studies on children and adolescents with cancer, representing a broad age range (6 to 18 years). Third, there was a consistent lack of detailed reporting regarding both PA parameters and QoL measures in the primary studies, limiting deeper interpretation. Fourth, PA levels were measured using questionnaires in five studies. Although the questionnaires were validated against accelerometer data, their accuracy remains relatively low. Finally, in children and adolescents with cancer, PA can improve HrO, ultimately helping to reduce the disease burden. However, it is necessary to make a more thorough and serious distinction based on age groups. Teenagers are different from school-age children.

## 5 Conclusion

In conclusion, PA can effectively reduce treatment-related adverse events in children and adolescents with cancer. Given the profound impact of cancer therapy on physical, psychological, emotional, cognitive, and social functioning, even low-intensity PA interventions—when leading to modest improvements or the maintenance of baseline status—may yield clinically meaningful benefits in HrO.

## Data Availability

The original contributions presented in the study are included in the article/**Supplementary Material**. Further inquiries can be directed to the corresponding author.
