# Supplementary material for: The effect of physical activity on health-related outcomes in children and adolescents with cancer: a systematic review and meta-analysis
Source: Front Oncol. 2026 Mar 31;16:1773060. doi: 10.3389/fonc.2026.1773060 (PMC13076115; doi:10.3389/fonc.2026.1773060)
Supplement: Supplementary file 1 [file DataSheet1.docx]

**The effect of physical activity on health-related outcomes in children and adolescents with cancer: A systematic review and meta-analysis**

**CONTENTS**

[**1. Search strategies 2**](#_Toc4795)

[**1.1 MEDLINE 2**](#_Toc17716)

[**1.2 Embase 4**](#_Toc20306)

[**1.3 Web of science 5**](#_Toc29597)

[**1.4 Cochrane Library 6**](#_Toc2390)

[**1.5 SPORTDiscus**](#_Toc27986) **6**

[**1.6 CINAHL 8**](#_Toc15184)

[**1.7 Psyclnfo 9**](#_Toc12756)

[**1.8 ERIC 10**](#_Toc7366)

[**1.9 Scopes 11**](#_Toc7366)

**2. Literature exclusion reasons 13**

**3. Implementation measures for intervention and control groups 19**

[**4.** **Risk of bias 21**](#_Toc30297)

[**4.1 Risk of bias graph 21**](#_Toc763)

[**4.2 Risk of bias summary 22**](#_Toc24267)

[**5.** **Meta-analysis results 23**](#_Toc30297)

[**6. Sensitivity analysis 48**](#_Toc30297)

[**7. Publication bias 66**](#_Toc30297)

[**8. Results of the trim and fill method 91**](#_Toc30297)

[**9. GRADE assessment 93**](#_Toc30297)

[**10. Adverse events and acceptability analyses 99**](#_Toc30297)

[**10.1 Acceptability analyses 99**](#_Toc763)

[**10.2 Adverse events 100**](#_Toc763)

[**11. ACSM round table standards 101**](#_Toc30297)

[**12. Regression analysis 102**](#_Toc30297)

[**13. Subgroup analysis 221**](#_Toc30297)

**14. Included literature 484**

[**15. PRISMA checklist 487**](#_Toc30297)

[**16. Reference 492**](#_Toc30297)

**1. Search strategies**

**1.1 MEDLINE**

#1 " Adolescent"[Mesh]

#2 (((((((((((((((Adolescents) OR (Adolescence)) OR (Adolescents, Female)) OR (Adolescent, Female)) OR (Female Adolescent)) OR (Female Adolescents)) OR (Adolescents, Male)) OR (Adolescent, Male)) OR (Male Adolescent)) OR (Male Adolescents)) OR (Youth)) OR (Youths)) OR (Teens)) OR (Teen)) OR (Teenagers)) OR (Teenager)

#3 (" Adolescent"[Mesh]) OR(((((((((((((((Adolescents) OR (Adolescence)) OR (Adolescents, Female)) OR (Adolescent, Female)) OR (Female Adolescent)) OR (Female Adolescents)) OR (Adolescents, Male)) OR (Adolescent, Male)) OR (Male Adolescent)) OR (Male Adolescents)) OR (Youth)) OR (Youths)) OR (Teens)) OR (Teen)) OR (Teenagers)) OR (Teenager)

#4 " Child "[Mesh]

#5 (Children) OR (CHILD)

#6 " Child "[Mesh] OR((Children) OR (CHILD))

#7 " Student "[Mesh]

#8 ((((Student) OR (School Enrollment)) OR (Enrollment, School)) OR (Enrollments, School)) OR (School Enrollments)

#9 " Student "[Mesh] OR (((((Student) OR (School Enrollment)) OR (Enrollment, School)) OR (Enrollments, School)) OR (School Enrollments))

#10 "Exercise"[Mesh]

#11 (Exercises[Title/Abstract]) OR (Physical Activity[Title/Abstract]) OR (Activities, Physical[Title/Abstract]) OR (Activity, Physical[Title/Abstract]) OR (Physical Activities[Title/Abstract]) OR (Exercise, Physical[Title/Abstract]) OR (Exercises, Physical[Title/Abstract]) OR (Physical Exercise[Title/Abstract]) OR (Physical Exercises[Title/Abstract]) OR (Exercise, Acute[Title/Abstract]) OR (Exercises, Acute[Title/Abstract]) OR (Exercise, Isometric[Title/Abstract]) OR (Exercises, Isometric[Title/Abstract]) OR (Isometric Exercises[Title/Abstract]) OR (Isometric Exercise[Title/Abstract]) OR (Exercise, Aerobic[Title/Abstract]) OR (Aerobic Exercise[Title/Abstract]) OR (Aerobic Exercises[Title/Abstract]) OR (Exercises, Aerobic[Title/Abstract]) OR (Exercise Training[Title/Abstract]) OR (Exercise Trainings[Title/Abstract]) OR (Training, Exercise[Title/Abstract]) OR (Trainings, Exercise[Title/Abstract])

#12 ("Exercise"[Mesh]) OR ((Exercises[Title/Abstract]) OR (Physical Activity[Title/Abstract]) OR (Activities, Physical[Title/Abstract]) OR (Activity, Physical[Title/Abstract]) OR (Physical Activities[Title/Abstract]) OR (Exercise, Physical[Title/Abstract]) OR (Exercises, Physical[Title/Abstract]) OR (Physical Exercise[Title/Abstract]) OR (Physical Exercises[Title/Abstract]) OR (Exercise, Acute[Title/Abstract]) OR (Exercises, Acute[Title/Abstract]) OR (Exercise, Isometric[Title/Abstract]) OR (Exercises, Isometric[Title/Abstract]) OR (Isometric Exercises[Title/Abstract]) OR (Isometric Exercise[Title/Abstract]) OR (Exercise, Aerobic[Title/Abstract]) OR (Aerobic Exercise[Title/Abstract]) OR (Aerobic Exercises[Title/Abstract]) OR (Exercises, Aerobic[Title/Abstract]) OR (Exercise Training[Title/Abstract]) OR (Exercise Trainings[Title/Abstract]) OR (Training, Exercise[Title/Abstract]) OR (Trainings, Exercise[Title/Abstract]))

#13 " Neoplasms "[Mesh]

#14 ((((((((((((((((Tumors[Title/Abstract]) OR (Neoplasia[Title/Abstract])) OR (Neoplasias[Title/Abstract])) OR (Neoplasm[Title/Abstract])) OR (Tumor[Title/Abstract])) OR (Cancer[Title/Abstract])) OR (Cancers[Title/Abstract])) OR (Malignant Neoplasm[Title/Abstract])) OR (Malignancy[Title/Abstract])) OR (Malignancies[Title/Abstract])) OR (Malignant Neoplasms[Title/Abstract])) OR (Neoplasm, Malignant[Title/Abstract])) OR (Neoplasms, Malignant[Title/Abstract])) OR (Benign Neoplasms[Title/Abstract])) OR (Neoplasms, Benign[Title/Abstract])) OR (Neoplasm, Benign[Title/Abstract])) OR (Benign Neoplasm[Title/Abstract])

#15 " Neoplasms "[Mesh] OR ((((((((((((((((Tumors[Title/Abstract]) OR (Neoplasia[Title/Abstract])) OR (Neoplasias[Title/Abstract])) OR (Neoplasm[Title/Abstract])) OR (Tumor[Title/Abstract])) OR (Cancer[Title/Abstract])) OR (Cancers[Title/Abstract])) OR (Malignant Neoplasm[Title/Abstract])) OR (Malignancy[Title/Abstract])) OR (Malignancies[Title/Abstract])) OR (Malignant Neoplasms[Title/Abstract])) OR (Neoplasm, Malignant[Title/Abstract])) OR (Neoplasms, Malignant[Title/Abstract])) OR (Benign Neoplasms[Title/Abstract])) OR (Neoplasms, Benign[Title/Abstract])) OR (Neoplasm, Benign[Title/Abstract])) OR (Benign Neoplasm[Title/Abstract])

#16 randomized controlled trial[Publication Type] OR randomized[Title/Abstract] OR placebo[Title/Abstract]

#17 ((((((Child[MeSH Terms]) OR (Children[Title/Abstract])) OR ((Adolescent[MeSH Terms]) OR ((((((((((((((((Adolescents[Title/Abstract]) OR (Adolescence[Title/Abstract])) OR (Adolescents, Female[Title/Abstract])) OR (Adolescent, Female[Title/Abstract])) OR (Female Adolescent[Title/Abstract])) OR (Female Adolescents[Title/Abstract])) OR (Adolescents, Male[Title/Abstract])) OR (Adolescent, Male[Title/Abstract])) OR (Male Adolescent[Title/Abstract])) OR (Male Adolescents[Title/Abstract])) OR (Youth[Title/Abstract])) OR (Youths[Title/Abstract])) OR (Teens[Title/Abstract])) OR (Teen[Title/Abstract])) OR (Teenagers[Title/Abstract])) OR (Teenager[Title/Abstract])))) OR ((Students[MeSH Terms]) OR (((((Student[Title/Abstract]) OR (School Enrollment[Title/Abstract])) OR (Enrollment, School[Title/Abstract])) OR (Enrollments, School[Title/Abstract])) OR (School Enrollments[Title/Abstract])))) AND ((cancer[MeSH Terms]) OR (((((((((((((((((Tumors[Title/Abstract]) OR (Neoplasia[Title/Abstract])) OR (Neoplasias[Title/Abstract])) OR (Neoplasm[Title/Abstract])) OR (Tumor[Title/Abstract])) OR (Cancer[Title/Abstract])) OR (Cancers[Title/Abstract])) OR (Malignant Neoplasm[Title/Abstract])) OR (Malignancy[Title/Abstract])) OR (Malignancies[Title/Abstract])) OR (Malignant Neoplasms[Title/Abstract])) OR (Neoplasm, Malignant[Title/Abstract])) OR (Neoplasms, Malignant[Title/Abstract])) OR (Benign Neoplasms[Title/Abstract])) OR (Neoplasms, Benign[Title/Abstract])) OR (Neoplasm, Benign[Title/Abstract])) OR (Benign Neoplasm[Title/Abstract])))) AND ((Exercise[MeSH Terms]) OR (((((((((((((((((((((((((Exercises) OR (Exercise, Physical)) OR (Exercises, Physical)) OR (Physical Exercise)) OR (Physical Exercises)) OR (Physical Activity)) OR (Activities, Physical)) OR (Activity, Physical)) OR (Physical Activities)) OR (Exercise, Aerobic)) OR (Aerobic Exercise)) OR (Aerobic Exercises)) OR (Exercises, Aerobic)) OR (Exercise, Isometric)) OR (Exercises, Isometric)) OR (Isometric Exercises)) OR (Isometric Exercise)) OR (Acute Exercise)) OR (Acute Exercises)) OR (Exercise, Acute)) OR (Exercises, Acute)) OR (Exercise Training)) OR (Exercise Trainings)) OR (Training, Exercise)) OR (Trainings, Exercise)))) AND (randomized controlled trial[Publication Type] OR randomized[Title/Abstract] OR placebo[Title/Abstract])

**1.2 Embase**

#1 'adolescent'/exp

#2 'Adolescents':ab,ti OR 'Adolescence':ab,ti OR 'Adolescents, Female':ab,ti OR 'Adolescent, Female':ab,ti OR 'Female Adolescent':ab,ti OR 'Female Adolescents':ab,ti OR 'Adolescents, Male':ab,ti OR 'Adolescent, Male':ab,ti OR 'Male Adolescent':ab,ti OR 'Male Adolescents':ab,ti OR 'Youth':ab,ti OR 'Youths':ab,ti OR 'Teens':ab,ti OR 'Teen':ab,ti OR 'Teenagers':ab,ti OR 'Teenager':ab,ti

#3 #1 OR #2

#4 'child'/exp

#5 child:ab,ti OR children:ab,ti

#6 #1 OR #2

#7 ' Students '/exp

#8 'Student':ab,ti OR 'School Enrollment':ab,ti OR 'Enrollment, School':ab,ti OR 'Enrollments, School':ab,ti OR 'School Enrollments':ab,ti

#9 #7 OR #8

#10 #3 OR #6 OR #9

#11 'exercise'/exp

#12 exercise:ab,ti OR exercises:ab,ti OR 'exercise, physical':ab,ti OR 'exercises, physical':ab,ti OR 'physical exercise':ab,ti OR 'physical exercises':ab,ti OR 'physical activity':ab,ti OR 'activities, physical':ab,ti OR 'activity, physical':ab,ti OR 'physical activities':ab,ti OR 'exercise, aerobic':ab,ti OR 'aerobic exercise':ab,ti OR 'aerobic exercises':ab,ti OR 'exercises, aerobic':ab,ti OR 'exercise, isometric':ab,ti OR 'exercises, isometric':ab,ti OR 'isometric exercises':ab,ti OR 'isometric exercise':ab,ti OR 'acute exercise':ab,ti OR 'acute exercises':ab,ti OR 'exercises, acute':ab,ti OR 'exercise training':ab,ti OR 'exercise trainings':ab,ti OR 'training, exercise':ab,ti OR 'trainings, exercise':ab,ti

#13 #11 OR #12

#14 ' Neoplasms '/exp

#15 ' Tumors’:ab,ti OR ‘Neoplasia’:ab,ti OR ‘Neoplasias’:ab,ti OR ‘Neoplasm’:ab,ti OR ‘Tumor’:ab,ti OR ‘Cancer’:ab,ti OR ‘Cancers’:ab,ti OR ‘Malignant Neoplasm’:ab,ti OR ‘Malignancy’:ab,ti OR ‘Malignancies’:ab,ti OR ‘Malignant Neoplasms’:ab,ti OR ‘Neoplasm, Malignant’:ab,ti OR ‘Neoplasms, Malignant’:ab,ti OR ‘Benign Neoplasms’:ab,ti OR ‘Neoplasms, Benign’:ab,ti OR ‘Neoplasm, Benign’:ab,ti OR ‘Benign Neoplasm ':ab,ti

#16 #14 OR #15

#17 'randomized controlled trial':ab,ti OR 'randomized':ab,ti OR 'placebo':ab,ti

#18 #10 AND #13 AND #16 AND #17

**1.3 Web of science**

#1 TS=Adolescent

#2 ((((((((((((((TS=(Adolescents) OR TS=(Adolescence)) OR TS=(Adolescents, Female)) OR TS=(Adolescent, Female)) OR TS=(Female Adolescent)) OR TS=(Female Adolescents)) OR TS=(Adolescents, Male)) OR TS=(Adolescent, Male)) OR TS=(Male Adolescent)) OR TS=(Male Adolescents)) OR TS=(Youth)) OR TS=(Youths)) OR TS=(Teens)) OR TS=(Teen)) OR TS=(Teenagers)) OR TS=(Teenager)

#3 #1 OR #2

#4 TS=Child

#5 (TS=(Children)) OR TS=(CHILD)

#6 #4 OR #5

#7 TS=Students

#8 (((TS=(Student) OR TS=(School Enrollment)) OR TS=(Enrollment, School)) OR TS=(Enrollments, School)) OR TS=(School Enrollments)

#9 #7 OR #8

#10 #3 OR #6 OR #9

#11 TS=Exercise

#12 (((((((((((((((((((((((TS=(Exercises) OR TS=(Exercise, Physical)) OR TS=(Exercises, Physical)) OR TS=(Physical Exercise)) OR TS=(Physical Exercises)) OR TS=(Physical Activity)) OR TS=(Activities, Physical)) OR TS=(Activity, Physical)) OR TS=(Physical Activities)) OR TS=(Exercise, Aerobic)) OR TS=(Aerobic Exercise)) OR TS=(Aerobic Exercises)) OR TS=(Exercises, Aerobic)) OR TS=(Exercise, Isometric)) OR TS=(Exercises, Isometric)) OR TS=(Isometric Exercises)) OR TS=(Isometric Exercise)) OR TS=(Acute Exercise)) OR TS=(Acute Exercises)) OR TS=(Exercise, Acute)) OR TS=(Exercises, Acute)) OR TS=(Exercise Training)) OR TS=(Exercise Trainings)) OR TS=(Training, Exercise)) OR TS=(Trainings, Exercise)

#13 #11 OR #12

#14 TS= Neoplasms

#15 TS=(cancer OR Tumors OR Neoplasia OR Neoplasias OR Neoplasm OR Tumor OR Cancer OR Cancers OR Malignant Neoplasm OR Malignancy OR Malignancies OR Malignant Neoplasms OR Neoplasm, Malignant OR Neoplasms, Malignant OR Benign Neoplasms OR Neoplasms, Benign OR Neoplasm, Benign OR Benign Neoplasm)

#16 #15 OR #14

#17 ((TS=(randomized controlled trial)) OR TS=(randomized)) OR TS=(placebo)

#18 #10 AND #13 AND #16 AND #17

**1.4 Cochrane Library**

#1 (Adolescent):ti,ab,kw

#2 (Adolescents):ti,ab,kw OR (Adolescence):ti,ab,kw OR (Adolescents, Female):ti,ab,kw OR (Adolescent, Female):ti,ab,kw OR (Female Adolescent):ti,ab,kw OR (Female Adolescents):ti,ab,kw OR (Adolescents, Male):ti,ab,kw OR (Adolescent, Male):ti,ab,kw OR (Male Adolescent):ti,ab,kw OR (Male Adolescents):ti,ab,kw OR (Youth):ti,ab,kw OR (Youths):ti,ab,kw OR (Teens):ti,ab,kw OR (Teen):ti,ab,kw OR (Teenagers):ti,ab,kw OR (Teenager):ti,ab,kw

#3 #1 OR #2

#4 (Child):ti,ab

#5 (Children):ti,ab,kw OR (CHILD):ti,ab,kw

#6 #4 OR #5

#7 (Students):ti,ab

#8 (Student):ti,ab,kw OR (School Enrollment):ti,ab,kw OR (Enrollment, School):ti,ab,kw OR (Enrollments, School):ti,ab,kw OR (School Enrollments):ti,ab,kw

#9 #7 OR #8

#10 #3 OR #6 OR #9

#11 (Exercise):ti,ab

#12 (Exercises):ti,ab,kw OR (Exercise, Physical):ti,ab,kw OR (Exercises, Physical):ti,ab,kw OR (Physical Exercise):ti,ab,kw OR (Physical Exercises):ti,ab,kw OR (Physical Activity):ti,ab,kw OR (Activities, Physical):ti,ab,kw OR (Activity, Physical):ti,ab,kw OR (Physical Activities):ti,ab,kw OR (Exercise, Aerobic):ti,ab,kw OR (Aerobic Exercise):ti,ab,kw OR (Aerobic Exercises):ti,ab,kw OR (Exercises, Aerobic):ti,ab,kw OR (Exercise, Isometric):ti,ab,kw OR (Exercises, Isometric):ti,ab,kw OR (Isometric Exercises):ti,ab,kw OR (Isometric Exercise):ti,ab,kw OR (Acute Exercise):ti,ab,kw OR (Acute Exercises):ti,ab,kw OR (Exercise, Acute):ti,ab,kw OR (Exercises, Acute):ti,ab,kw OR (Exercise Training):ti,ab,kw OR (Exercise Trainings):ti,ab,kw OR (Training, Exercise):ti,ab,kw OR (Trainings, Exercise):ti,ab,kw

#13 #11 OR #12

#14 (Neoplasms):ti,ab

#15 (Tumors):ti,ab,kw OR (Neoplasia):ti,ab,kw OR (Neoplasias):ti,ab,kw OR (Neoplasm):ti,ab,kw OR (Tumor):ti,ab,kw OR (Cancer):ti,ab,kw OR (Cancers):ti,ab,kw OR (Malignant Neoplasm):ti,ab,kw OR (Malignancy):ti,ab,kw OR (Malignancies):ti,ab,kw OR (Malignant Neoplasms):ti,ab,kw OR (Neoplasm, Malignant):ti,ab,kw OR (Neoplasms, Malignant):ti,ab,kw OR (Benign Neoplasms):ti,ab,kw OR (Neoplasms, Benign):ti,ab,kw OR (Neoplasm, Benign):ti,ab,kw OR (Benign Neoplasm):ti,ab,kw

#16 #14 OR #15

#17 #10 and #13 and #16

**1.5 SPORTDiscus**

#1 ( TI Adolescent OR AB Adolescent ) OR ( TI (((((((((((((((Adolescents) OR (Adolescence)) OR (Adolescents, Female)) OR (Adolescent, Female)) OR (Female Adolescent)) OR (Female Adolescents)) OR (Adolescents, Male)) OR (Adolescent, Male)) OR (Male Adolescent)) OR (Male Adolescents)) OR (Youth)) OR (Youths)) OR (Teens)) OR (Teen)) OR (Teenagers)) OR (Teenager) OR AB (((((((((((((((Adolescents) OR (Adolescence)) OR (Adolescents, Female)) OR (Adolescent, Female)) OR (Female Adolescent)) OR (Female Adolescents)) OR (Adolescents, Male)) OR (Adolescent, Male)) OR (Male Adolescent)) OR (Male Adolescents)) OR (Youth)) OR (Youths)) OR (Teens)) OR (Teen)) OR (Teenagers)) OR (Teenager) )

#2 ( TI Child OR AB Child ) OR ( TI (Children) OR (CHILD) OR AB (Children) OR (CHILD) )

#3 ( TI Students OR AB Students ) OR ( TI ((((Student) OR (School Enrollment)) OR (Enrollment, School)) OR (Enrollments, School)) OR (School Enrollments) OR AB ((((Student) OR (School Enrollment)) OR (Enrollment, School)) OR (Enrollments, School)) OR (School Enrollments) )

#4 #1 OR #2 OR #3

#5 ( TI Exercise OR AB Exercise ) OR ( TI ((((((((((((((((((((((((Exercises) OR (Exercise, Physical)) OR (Exercises, Physical)) OR (Physical Exercise)) OR (Physical Exercises)) OR (Physical Activity)) OR (Activities, Physical)) OR (Activity, Physical)) OR (Physical Activities)) OR (Exercise, Aerobic)) OR (Aerobic Exercise)) OR (Aerobic Exercises)) OR (Exercises, Aerobic)) OR (Exercise, Isometric)) OR (Exercises, Isometric)) OR (Isometric Exercises)) OR (Isometric Exercise)) OR (Acute Exercise)) OR (Acute Exercises)) OR (Exercise, Acute)) OR (Exercises, Acute)) OR (Exercise Training)) OR (Exercise Trainings)) OR (Training, Exercise)) OR (Trainings, Exercise) OR AB ((((((((((((((((((((((((Exercises) OR (Exercise, Physical)) OR (Exercises, Physical)) OR (Physical Exercise)) OR (Physical Exercises)) OR (Physical Activity)) OR (Activities, Physical)) OR (Activity, Physical)) OR (Physical Activities)) OR (Exercise, Aerobic)) OR (Aerobic Exercise)) OR (Aerobic Exercises)) OR (Exercises, Aerobic)) OR (Exercise, Isometric)) OR (Exercises, Isometric)) OR (Isometric Exercises)) OR (Isometric Exercise)) OR (Acute Exercise)) OR (Acute Exercises)) OR (Exercise, Acute)) OR (Exercises, Acute)) OR (Exercise Training)) OR (Exercise Trainings)) OR (Training, Exercise)) OR (Trainings, Exercise) )

#6 ( TI Sleep OR AB Sleep ) OR ( TI (((((((Sleeping Habits) OR (Sleep Habits)) OR (Habit, Sleep)) OR (Habits, Sleep)) OR (Sleep Habit)) OR (Sleeping Habit)) OR (Habit, Sleeping)) OR (Habits, Sleeping) OR AB (((((((Sleeping Habits) OR (Sleep Habits)) OR (Habit, Sleep)) OR (Habits, Sleep)) OR (Sleep Habit)) OR (Sleeping Habit)) OR (Habit, Sleeping)) OR (Habits, Sleeping) )

#7 TI(Tumors OR Neoplasia OR Neoplasias OR Neoplasm OR Tumor OR Cancer OR Cancers OR Malignant Neoplasm OR Malignancy OR Malignancies OR Malignant Neoplasms OR Neoplasm, Malignant OR Neoplasms, Malignant OR Benign Neoplasms OR Neoplasms, Benign OR Neoplasm, Benign OR Benign Neoplasm) OR AB(Tumors OR Neoplasia OR Neoplasias OR Neoplasm OR Tumor OR Cancer OR Cancers OR Malignant Neoplasm OR Malignancy OR Malignancies OR Malignant Neoplasms OR Neoplasm, Malignant OR Neoplasms, Malignant OR Benign Neoplasms OR Neoplasms, Benign OR Neoplasm, Benign OR Benign Neoplasm)

#8 #6 OR #7

#9 TI (randomized controlled trial OR randomized OR placebo) OR AB (randomized controlled trial OR randomized OR placebo)

#10 #4 AND #5 AND #8 AND #9

**1.6 CINAHL**

#1 ( TI Adolescent OR AB Adolescent ) OR ( TI (((((((((((((((Adolescents) OR (Adolescence)) OR (Adolescents, Female)) OR (Adolescent, Female)) OR (Female Adolescent)) OR (Female Adolescents)) OR (Adolescents, Male)) OR (Adolescent, Male)) OR (Male Adolescent)) OR (Male Adolescents)) OR (Youth)) OR (Youths)) OR (Teens)) OR (Teen)) OR (Teenagers)) OR (Teenager) OR AB (((((((((((((((Adolescents) OR (Adolescence)) OR (Adolescents, Female)) OR (Adolescent, Female)) OR (Female Adolescent)) OR (Female Adolescents)) OR (Adolescents, Male)) OR (Adolescent, Male)) OR (Male Adolescent)) OR (Male Adolescents)) OR (Youth)) OR (Youths)) OR (Teens)) OR (Teen)) OR (Teenagers)) OR (Teenager) )

#2 ( TI Child OR AB Child ) OR ( TI (Children) OR (CHILD) OR AB (Children) OR (CHILD) )

#3 ( TI Students OR AB Students ) OR ( TI ((((Student) OR (School Enrollment)) OR (Enrollment, School)) OR (Enrollments, School)) OR (School Enrollments) OR AB ((((Student) OR (School Enrollment)) OR (Enrollment, School)) OR (Enrollments, School)) OR (School Enrollments) )

#4 #1 OR #2 OR #3

#5 ( TI Exercise OR AB Exercise ) OR ( TI ((((((((((((((((((((((((Exercises) OR (Exercise, Physical)) OR (Exercises, Physical)) OR (Physical Exercise)) OR (Physical Exercises)) OR (Physical Activity)) OR (Activities, Physical)) OR (Activity, Physical)) OR (Physical Activities)) OR (Exercise, Aerobic)) OR (Aerobic Exercise)) OR (Aerobic Exercises)) OR (Exercises, Aerobic)) OR (Exercise, Isometric)) OR (Exercises, Isometric)) OR (Isometric Exercises)) OR (Isometric Exercise)) OR (Acute Exercise)) OR (Acute Exercises)) OR (Exercise, Acute)) OR (Exercises, Acute)) OR (Exercise Training)) OR (Exercise Trainings)) OR (Training, Exercise)) OR (Trainings, Exercise) OR AB ((((((((((((((((((((((((Exercises) OR (Exercise, Physical)) OR (Exercises, Physical)) OR (Physical Exercise)) OR (Physical Exercises)) OR (Physical Activity)) OR (Activities, Physical)) OR (Activity, Physical)) OR (Physical Activities)) OR (Exercise, Aerobic)) OR (Aerobic Exercise)) OR (Aerobic Exercises)) OR (Exercises, Aerobic)) OR (Exercise, Isometric)) OR (Exercises, Isometric)) OR (Isometric Exercises)) OR (Isometric Exercise)) OR (Acute Exercise)) OR (Acute Exercises)) OR (Exercise, Acute)) OR (Exercises, Acute)) OR (Exercise Training)) OR (Exercise Trainings)) OR (Training, Exercise)) OR (Trainings, Exercise) )

#6 ( TI Sleep OR AB Sleep ) OR ( TI (((((((Sleeping Habits) OR (Sleep Habits)) OR (Habit, Sleep)) OR (Habits, Sleep)) OR (Sleep Habit)) OR (Sleeping Habit)) OR (Habit, Sleeping)) OR (Habits, Sleeping) OR AB (((((((Sleeping Habits) OR (Sleep Habits)) OR (Habit, Sleep)) OR (Habits, Sleep)) OR (Sleep Habit)) OR (Sleeping Habit)) OR (Habit, Sleeping)) OR (Habits, Sleeping) )

#7 TI(Tumors OR Neoplasia OR Neoplasias OR Neoplasm OR Tumor OR Cancer OR Cancers OR Malignant Neoplasm OR Malignancy OR Malignancies OR Malignant Neoplasms OR Neoplasm, Malignant OR Neoplasms, Malignant OR Benign Neoplasms OR Neoplasms, Benign OR Neoplasm, Benign OR Benign Neoplasm) OR AB(Tumors OR Neoplasia OR Neoplasias OR Neoplasm OR Tumor OR Cancer OR Cancers OR Malignant Neoplasm OR Malignancy OR Malignancies OR Malignant Neoplasms OR Neoplasm, Malignant OR Neoplasms, Malignant OR Benign Neoplasms OR Neoplasms, Benign OR Neoplasm, Benign OR Benign Neoplasm)

#8 #6 OR #7

#9 TI (randomized controlled trial OR randomized OR placebo) OR AB (randomized controlled trial OR randomized OR placebo)

#10 #4 AND #5 AND #8 AND #9

**1.7 Psyclnfo**

#1 ( TI Adolescent OR AB Adolescent ) OR ( TI (((((((((((((((Adolescents) OR (Adolescence)) OR (Adolescents, Female)) OR (Adolescent, Female)) OR (Female Adolescent)) OR (Female Adolescents)) OR (Adolescents, Male)) OR (Adolescent, Male)) OR (Male Adolescent)) OR (Male Adolescents)) OR (Youth)) OR (Youths)) OR (Teens)) OR (Teen)) OR (Teenagers)) OR (Teenager) OR AB (((((((((((((((Adolescents) OR (Adolescence)) OR (Adolescents, Female)) OR (Adolescent, Female)) OR (Female Adolescent)) OR (Female Adolescents)) OR (Adolescents, Male)) OR (Adolescent, Male)) OR (Male Adolescent)) OR (Male Adolescents)) OR (Youth)) OR (Youths)) OR (Teens)) OR (Teen)) OR (Teenagers)) OR (Teenager) )

#2 ( TI Child OR AB Child ) OR ( TI (Children) OR (CHILD) OR AB (Children) OR (CHILD) )

#3 ( TI Students OR AB Students ) OR ( TI ((((Student) OR (School Enrollment)) OR (Enrollment, School)) OR (Enrollments, School)) OR (School Enrollments) OR AB ((((Student) OR (School Enrollment)) OR (Enrollment, School)) OR (Enrollments, School)) OR (School Enrollments) )

#4 #1 OR #2 OR #3

#5 ( TI Exercise OR AB Exercise ) OR ( TI ((((((((((((((((((((((((Exercises) OR (Exercise, Physical)) OR (Exercises, Physical)) OR (Physical Exercise)) OR (Physical Exercises)) OR (Physical Activity)) OR (Activities, Physical)) OR (Activity, Physical)) OR (Physical Activities)) OR (Exercise, Aerobic)) OR (Aerobic Exercise)) OR (Aerobic Exercises)) OR (Exercises, Aerobic)) OR (Exercise, Isometric)) OR (Exercises, Isometric)) OR (Isometric Exercises)) OR (Isometric Exercise)) OR (Acute Exercise)) OR (Acute Exercises)) OR (Exercise, Acute)) OR (Exercises, Acute)) OR (Exercise Training)) OR (Exercise Trainings)) OR (Training, Exercise)) OR (Trainings, Exercise) OR AB ((((((((((((((((((((((((Exercises) OR (Exercise, Physical)) OR (Exercises, Physical)) OR (Physical Exercise)) OR (Physical Exercises)) OR (Physical Activity)) OR (Activities, Physical)) OR (Activity, Physical)) OR (Physical Activities)) OR (Exercise, Aerobic)) OR (Aerobic Exercise)) OR (Aerobic Exercises)) OR (Exercises, Aerobic)) OR (Exercise, Isometric)) OR (Exercises, Isometric)) OR (Isometric Exercises)) OR (Isometric Exercise)) OR (Acute Exercise)) OR (Acute Exercises)) OR (Exercise, Acute)) OR (Exercises, Acute)) OR (Exercise Training)) OR (Exercise Trainings)) OR (Training, Exercise)) OR (Trainings, Exercise) )

#6 ( TI Sleep OR AB Sleep ) OR ( TI (((((((Sleeping Habits) OR (Sleep Habits)) OR (Habit, Sleep)) OR (Habits, Sleep)) OR (Sleep Habit)) OR (Sleeping Habit)) OR (Habit, Sleeping)) OR (Habits, Sleeping) OR AB (((((((Sleeping Habits) OR (Sleep Habits)) OR (Habit, Sleep)) OR (Habits, Sleep)) OR (Sleep Habit)) OR (Sleeping Habit)) OR (Habit, Sleeping)) OR (Habits, Sleeping) )

#7 TI(Tumors OR Neoplasia OR Neoplasias OR Neoplasm OR Tumor OR Cancer OR Cancers OR Malignant Neoplasm OR Malignancy OR Malignancies OR Malignant Neoplasms OR Neoplasm, Malignant OR Neoplasms, Malignant OR Benign Neoplasms OR Neoplasms, Benign OR Neoplasm, Benign OR Benign Neoplasm) OR AB(Tumors OR Neoplasia OR Neoplasias OR Neoplasm OR Tumor OR Cancer OR Cancers OR Malignant Neoplasm OR Malignancy OR Malignancies OR Malignant Neoplasms OR Neoplasm, Malignant OR Neoplasms, Malignant OR Benign Neoplasms OR Neoplasms, Benign OR Neoplasm, Benign OR Benign Neoplasm)

#8 #6 OR #7

#9 TI (randomized controlled trial OR randomized OR placebo) OR AB (randomized controlled trial OR randomized OR placebo)

#10 #4 AND #5 AND #8 AND #9

**1.8 ERIC**

#1 ( TI Adolescent OR AB Adolescent ) OR ( TI (((((((((((((((Adolescents) OR (Adolescence)) OR (Adolescents, Female)) OR (Adolescent, Female)) OR (Female Adolescent)) OR (Female Adolescents)) OR (Adolescents, Male)) OR (Adolescent, Male)) OR (Male Adolescent)) OR (Male Adolescents)) OR (Youth)) OR (Youths)) OR (Teens)) OR (Teen)) OR (Teenagers)) OR (Teenager) OR AB (((((((((((((((Adolescents) OR (Adolescence)) OR (Adolescents, Female)) OR (Adolescent, Female)) OR (Female Adolescent)) OR (Female Adolescents)) OR (Adolescents, Male)) OR (Adolescent, Male)) OR (Male Adolescent)) OR (Male Adolescents)) OR (Youth)) OR (Youths)) OR (Teens)) OR (Teen)) OR (Teenagers)) OR (Teenager) )

#2 ( TI Child OR AB Child ) OR ( TI (Children) OR (CHILD) OR AB (Children) OR (CHILD) )

#3 ( TI Students OR AB Students ) OR ( TI ((((Student) OR (School Enrollment)) OR (Enrollment, School)) OR (Enrollments, School)) OR (School Enrollments) OR AB ((((Student) OR (School Enrollment)) OR (Enrollment, School)) OR (Enrollments, School)) OR (School Enrollments) )

#4 #1 OR #2 OR #3

#5 ( TI Exercise OR AB Exercise ) OR ( TI ((((((((((((((((((((((((Exercises) OR (Exercise, Physical)) OR (Exercises, Physical)) OR (Physical Exercise)) OR (Physical Exercises)) OR (Physical Activity)) OR (Activities, Physical)) OR (Activity, Physical)) OR (Physical Activities)) OR (Exercise, Aerobic)) OR (Aerobic Exercise)) OR (Aerobic Exercises)) OR (Exercises, Aerobic)) OR (Exercise, Isometric)) OR (Exercises, Isometric)) OR (Isometric Exercises)) OR (Isometric Exercise)) OR (Acute Exercise)) OR (Acute Exercises)) OR (Exercise, Acute)) OR (Exercises, Acute)) OR (Exercise Training)) OR (Exercise Trainings)) OR (Training, Exercise)) OR (Trainings, Exercise) OR AB ((((((((((((((((((((((((Exercises) OR (Exercise, Physical)) OR (Exercises, Physical)) OR (Physical Exercise)) OR (Physical Exercises)) OR (Physical Activity)) OR (Activities, Physical)) OR (Activity, Physical)) OR (Physical Activities)) OR (Exercise, Aerobic)) OR (Aerobic Exercise)) OR (Aerobic Exercises)) OR (Exercises, Aerobic)) OR (Exercise, Isometric)) OR (Exercises, Isometric)) OR (Isometric Exercises)) OR (Isometric Exercise)) OR (Acute Exercise)) OR (Acute Exercises)) OR (Exercise, Acute)) OR (Exercises, Acute)) OR (Exercise Training)) OR (Exercise Trainings)) OR (Training, Exercise)) OR (Trainings, Exercise) )

#6 ( TI Sleep OR AB Sleep ) OR ( TI (((((((Sleeping Habits) OR (Sleep Habits)) OR (Habit, Sleep)) OR (Habits, Sleep)) OR (Sleep Habit)) OR (Sleeping Habit)) OR (Habit, Sleeping)) OR (Habits, Sleeping) OR AB (((((((Sleeping Habits) OR (Sleep Habits)) OR (Habit, Sleep)) OR (Habits, Sleep)) OR (Sleep Habit)) OR (Sleeping Habit)) OR (Habit, Sleeping)) OR (Habits, Sleeping) )

#7 TI(Tumors OR Neoplasia OR Neoplasias OR Neoplasm OR Tumor OR Cancer OR Cancers OR Malignant Neoplasm OR Malignancy OR Malignancies OR Malignant Neoplasms OR Neoplasm, Malignant OR Neoplasms, Malignant OR Benign Neoplasms OR Neoplasms, Benign OR Neoplasm, Benign OR Benign Neoplasm) OR AB(Tumors OR Neoplasia OR Neoplasias OR Neoplasm OR Tumor OR Cancer OR Cancers OR Malignant Neoplasm OR Malignancy OR Malignancies OR Malignant Neoplasms OR Neoplasm, Malignant OR Neoplasms, Malignant OR Benign Neoplasms OR Neoplasms, Benign OR Neoplasm, Benign OR Benign Neoplasm)

#8 #6 OR #7

#9 TI (randomized controlled trial OR randomized OR placebo) OR AB (randomized controlled trial OR randomized OR placebo)

#10 #4 AND #5 AND #8 AND #9

**1.9** **Scopes**

#1 TITLE-ABS-KEY(Child OR Children)

#2 TITLE-ABS-KEY(Adolescent OR Adolescents OR Adolescence OR Adolescents, Female OR Adolescent, Female OR Female Adolescent OR Female Adolescents OR Adolescents, Male OR Adolescent, Male OR Male Adolescent OR Male Adolescents OR Youth OR Youths OR Teens OR Teen OR Teenagers OR Teenager)

#3 TITLE-ABS-KEY(Students OR Student OR School Enrollment OR Enrollment, School OR Enrollments, School OR School Enrollments)

#4 #1OR #2 OR #3

#5 TITLE-ABS-KEY(Neoplasms OR Tumors OR Neoplasia OR Neoplasias OR Neoplasm OR Tumor OR Cancer OR Cancers OR Malignant Neoplasm OR Malignancy OR Malignancies OR Malignant Neoplasms OR Neoplasm, Malignant OR Neoplasms, Malignant OR Benign Neoplasms OR Neoplasms, Benign OR Neoplasm, Benign OR Benign Neoplasm)

#6 Exercise OR Exercises OR Physical Exercises OR Physical Exercise OR Physical Activity OR Physical Activities OR Aerobic Exercise OR Aerobic Exercises OR Exercises Isometric OR Isometric Exercises OR Acute Exercise OR Acute Exercise OR Training OR Trainings

#7 TITLE-ABS-KEY(randomized controlled trial OR randomized OR placebo)

#8 #4 OR #5 OR #6 OR #7

**2. Literature exclusion reasons**

| Study | Exclude reasons |
| --- | --- |
| Inhospital Exercise Training in Children With Cancer: Does It Work for All? | No control group |
| Randomized web-based physical activity intervention in adolescent survivors of childhood cancer | Non-exercise intervention |
| Effects of Cognitive Training and Exergaming in Pediatric Cancer Survivors-A Randomized Clinical Trial | Data could not be obtained |
| A randomized controlled trial of a structured exercise intervention after the completion of acute cancer treatment in adolescents and young adults | Age not meet |
| Feasibility and Preliminary Effects of a 1-Week Vestibular Rehabilitation Day Camp in Children with Developmental Coordination Disorder | subject incompatibility |
| Impact of physical activity on postural stability and coordination in children with posterior fossa tumor: randomized control phase III trial | Three types of exercise interventions |
| Effects of a combined physical and psychosocial training for children with cancer: a randomized controlled trial | Repeat |
| Effects of strength exercise interventions on activities of daily living, motor performance, and physical activity in children and adolescents with leukemia or non-Hodgkin lymphoma: Results from the randomized controlled ActiveADL Study | Two types of exercise interventions |
| Exercise reduces systemic immune inflammation index (SII) in childhood cancer patients | Data could not be obtained |
| Effect of virtual reality-based exercise intervention on sleep quality in children with acute lymphoblastic leukemia and healthy siblings: A randomized controlled trial | Repeat |
| Feasibility of FitSurvivor: A technology-enhanced group-based fitness intervention for adolescent and young adult survivors of childhood cancer | Non-exercise intervention |
| Whole-Body Vibration Training Designed to Improve Functional Impairments After Pediatric Inpatient Anticancer Therapy: A Pilot Study | The test programme does not meet |
| Influence of a Moderate-Intensity Exercise Program on Early NK Cell Immune Recovery in Pediatric Patients After Reduced-Intensity Hematopoietic Stem Cell Transplantation | Repeat |
| Feasibility of FitSurvivor: A technology-enhanced group-based fitness intervention for adolescent and young adult survivors of childhood cancer | Repeat |
| Clinical field testing of an enhanced-activity intervention in hospitalized children with cancer | The test programme does not meet |
| Effect of Early Physical Therapy on Children of School Age With Hematological Cancer: A Quasi-Randomized Controlled Pilot Study | The test programme does not meet |
| A randomized trial investigating an exercise program to prevent reduction of Bone mineral density and impairment of motor performance during treatment for childhood acute lymphoblastic leukemia | Data could not be obtained |
| Effect of adapted physical activity sessions in the hospital on health-related quality of life for children with cancer: a cross-over randomized trial | Cross trial |
| Investigation of the effect of task-orientated rehabilitation program on motor skills of children with childhood cancer: a randomized-controlled trial | Repeat |
| Inhospital Exercise Training in Children With Cancer: Does It Work for All? | Repeat |
| Effectiveness of a healthy lifestyle program based on a mobile serious game for childhood cancer survivors: A quasi-randomized trial | Semi-randomized trials |
| Children with cancer and their cardiorespiratory fitness and physical function-the long-term effects of a physical activity program during treatment: a multicenter non-randomized controlled trial | Semi-randomized trials |
| Adventure-based training to promote physical activity and reduce Fatigue among childhood cancer survivors: A randomized controlled trial | Repeat |
| A randomised controlled trial investigating the ability for supervised exercise to reduce treatment-related decline in adolescent and young adult cancer patients | Age not meet |
| The effect of an aerobic exercise program on the quality of life in children with cancer | Repeat |
| Whole-body vibration training in addition to muscle-strengthening exercises alone in improving muscle function in children with Neurofibromatosis Type 1 - a randomised interventional trial | Includes other interventions |
| Exercise Intervention in Pediatric Patients with Solid Tumors: The Physical Activity in Pediatric Cancer Trial | Repeat |
| EFFECT OF TREADMILL TRAINING ON BALANCE AFTER CHEMOTHERAPY IN CHILDREN WITH ACUTE LYMPHOBLASTIC LEUKEMIA | Pilot programme |
| A Bout of High-Intensity Interval Training (HIIT) in Children and Adolescents during Acute Cancer Treatment—A Pilot Feasibility Study | The test programme does not meet |
| QOL-26. PLAYFUL SENSORIMOTOR TRAINING TO REDUCE THE SYMPTOMS OF CHEMOTHERAPY-INDUCED PERIPHERAL NEUROPATHY IN PEDIATRIC BRAIN TUMOR PATIENTS- A RANDOMIZED CONTROLLED TRIAL (RESET) | Unable to access articles |
| Home physical activity intervention to improve cognitive late effects in children treated with radiation for brain tumors: Descriptive feasibility data from a pilot randomized controlled trial (RCT) | Unable to access articles |
| Short term effects on physical fitness of a 12-week exercise and psychosocial training program in childhood cancer patients | Data could not be obtained |
| Physical activity through homebased exercise-gaming after childhood brain tumour treatment-A method to improve motor and process function | Data could not be obtained |
| Effects of an adapted physical activity program with a playful pedagogy in a service of paediatric oncology | Data could not be obtained |
| The effect of an aerobic exercise program on the quality of life in children with cancer | Repeat |
| Metabolic response to exercise in childhood brain tumor survivors: A pilot controlled study. | Healthy control group |
| Treinamento muscular inspiratório em crianças com leucemia aguda: resultados preliminares | The test programme does not meet |
| Fitness of Children With Standard-risk Acute Lymphoblastic Leukemia During Maintenance Therapy | Includes other interventions |
| Active video gaming improves body coordination in survivors of childhood brain tumours | Cross trial |
| Mobilization patterns of children on a hematology/oncology inpatient ward | The test programme does not meet |
| Intrahospital supervised exercise training: a complementary tool in the therapeutic armamentarium against childhood leukemia | The test programme does not meet |
| Modifying Bone mineral density, physical function, and quality of life in children with acute lymphoblastic leukemia | Two types of exercise interventions |
| Sustainability of an Integrated Adventure-Based Training and Health Education Program to Enhance quality of life Among Chinese Childhood Cancer Survivors A Randomized Controlled Trial | Repeat |
| Evaluation of a combined supervised inpatient and online-home-based exercise pro-grams to promote physical activity behaviour in children and adolescents with oncological diseases undergoing medical therapy: HAPPY - a randomized controlled trail | Pilot programme |
| Investigation of the effect of task-orientated rehabilitation program on motor skills of children with childhood cancer: a randomized-controlled trial | The test programme does not meet |
| Evaluation of a combined supervised inpatient and online-home-based exercise pro-grams to promote physical activity behaviour in children and adolescents with oncological diseases undergoing medical therapy: HAPPY - a randomized controlled trail | Repeat |
| [Effects of physically active video gaming on cognition and activities of daily living in childhood brain tumor survivors: a randomized pilot study](https://pubmed.ncbi.nlm.nih.gov/?size=50&term=Sabel+M&cauthor_id=31385977) | Cross trial |
| Psychological Effects of a Structured Exercise Intervention During Umbilical Cord Blood Transplantation in Children and Adolescents | Data could not be obtained |
| Effect of a physical activity intervention on lower body bone health in childhood cancer survivors: A randomized controlled trial (SURfit) | Age not met |
| Randomized web-based physical activity intervention in adolescent survivors of childhood cancer | Repeat |
| Effects of a structured counselling-based intervention to improve physical activity behaviour of adolescents and young adult cancer survivors – the randomized phase II Motivate AYA – MAYA trial | Age not meet |
| Exercise Intervention in Pediatric Patients with Solid Tumors: The Physical Activity in Pediatric Cancer Trial | Repeat |
| Sustainability of an Integrated Adventure-Based Training and Health Education Program to Enhance quality of life Among Chinese Childhood Cancer Survivors A Randomized Controlled Trial | The test programme does not meet |
| Cost-effectiveness of a combined physical exercise and psychosocial training intervention for children with cancer: Results from the quality of life in motion study | Includes other interventions |
| A randomised controlled trial investigating the ability for supervised exercise to reduce treatment-related decline in adolescent and young adult cancer patients | Age not meet |
| Effect of Early Physical Therapy on Children of School Age With Hematological Cancer: A Quasi-Randomized Controlled Pilot Study | Repeat |
| Implementation of structured physical activity in the pediatric stem cell transplantation | Repeat |
| Effects of a combined physical and psychosocial intervention program for childhood cancer patients on quality of life and psychosocial functioning: results of the QLIM randomized clinical trial | Includes other interventions |
| Effects of acute exercise on neutrophils in pediatric acute lymphoblastic leukemia survivors: a pilot study | Non-randomised controlled trials |
| A randomised controlled trial investigating the ability for supervised exercise to reduce treatment-related decline in adolescent and young adult cancer patients | Age not meet |
| [Effects of a Four-Week Rehabilitation Program on Motor Performance, quality of life and Fatigue in Childhood Cancer Patients and Healthy Siblings] | Non-randomised controlled trials |
| Efficacy of Dual Task Training on Children With Ataxia After Medulloblastoma Resection | The test programme does not meet |
| Effect of Early Physical Therapy on Children of School Age With Hematological Cancer: A Quasi-Randomized Controlled Pilot Study | The test programme does not meet |
| Feasibility of a combined supervised and home‐based whole‐body vibration intervention in children after inpatient oncological treatment | Non-exercise intervention |
| Feasibility and effects of a home-based intervention using activity trackers on achievement of individual goals, quality of life and motor performance in patients with paediatric cancer | The test programme does not meet |
| Evaluation of a combined supervised inpatient and online-home-based exercise pro-grams to promote physical activity behaviour in children and adolescents with oncological diseases undergoing medical therapy: HAPPY - a randomized controlled trail | Pilot programme |

**3. Implementation measures for intervention and control groups**

| Study ID | Intervention group measures | Control group measures |
| --- | --- | --- |
| Masoud 2023 | Exercise games that include aerobic and Balance exercises | Orientation programme for physical activity benefits |
| Braam 2018 | Aerobics and strength training | Routine care |
| Saultier 2021 | Skilled sports such as dance, basketball, skiing, etc. | Recreational games |
| Ruble 2016 | Skill-based sports such as rock climbing, basketball, gymnastics, etc. | Non-participation in exercise |
| Tanriverdi 2022 | Exercise games with a focus on aerobic exercise | Routine care |
| Fiuza-Luces 2017 | Aerobics and strength training | Physiotherapy |
| Caru 2024 | Aerobics and strength training | Routine care |
| Manchola-González 2020 | Aerobics, strength training and Flexibility exercises | Orientation programme for physical activity benefits |
| Lam 2018 | Aerobics, strength training and Flexibility exercises | Orientation programme for physical activity benefits |
| Tanir 2013 | Aerobics, strength training and Flexibility exercises | Non-participation in exercise |
| Stössel 2020 | Aerobics and strength training | Routine care |
| Müller 2014 | Skill-based exercises such as deep squats, basketball, football, etc. | Physiotherapy |
| Marchese 2004 | Aerobics and strength training | Routine care |
| Yeh 2011 | Aerobic exercise | Routine care |
| Chamorro-Viña 2017 | Aerobics and strength training | Routine care |
| Fiuza-Luces 2017 | Aerobics and strength training | Physiotherapy |
| Li 2018 | Adventure group sports such as shuttle running, rock climbing and rope descents | Orientation programme for physical activity benefits |
| Senn-Malashonak 2019 | Aerobics, strength training and Flexibility exercises | Mental relaxation training |
| Elnaggar 2024 | Aerobic exercise | Physiotherapy |
| Khodashenas 2017 | Aerobic exercise | Routine care |
| Waked 2018 | Aerobic exercise | Non-participation in exercise |
| Şahin 2020 | Functional trainings | Home-based treatment programmes |
| Dubnov-Raz 2015 | Aerobics and strength training | Non-participation in exercise |

**4. Risk of bias**

**4.1 Figure S1. Risk of bias graph**

**Figure S1. Risk of bias graph**

**4.2 Figure S2. The detail of risk of bias**


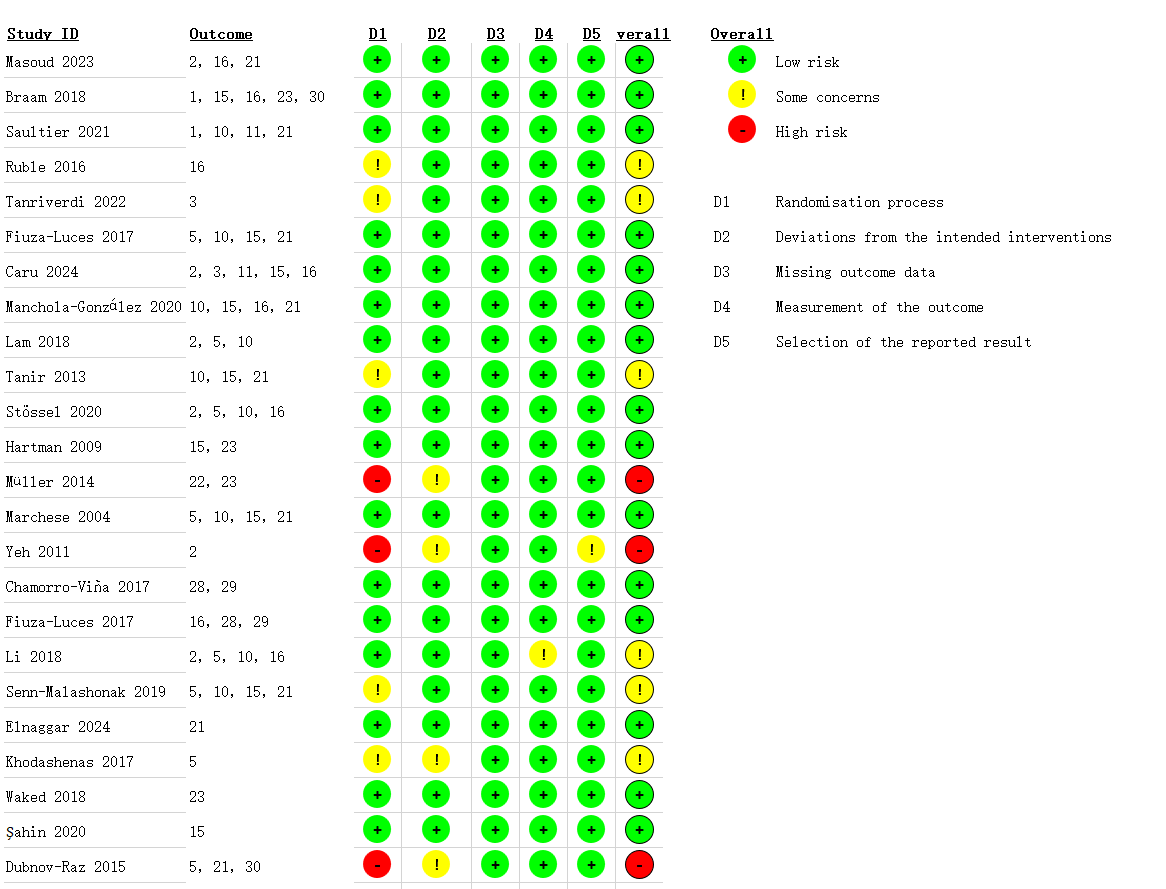


**Figure S2. The detail of risk of bias**

**5. Meta-analysis results**

**5.1 Quality of life - quality of life scale**


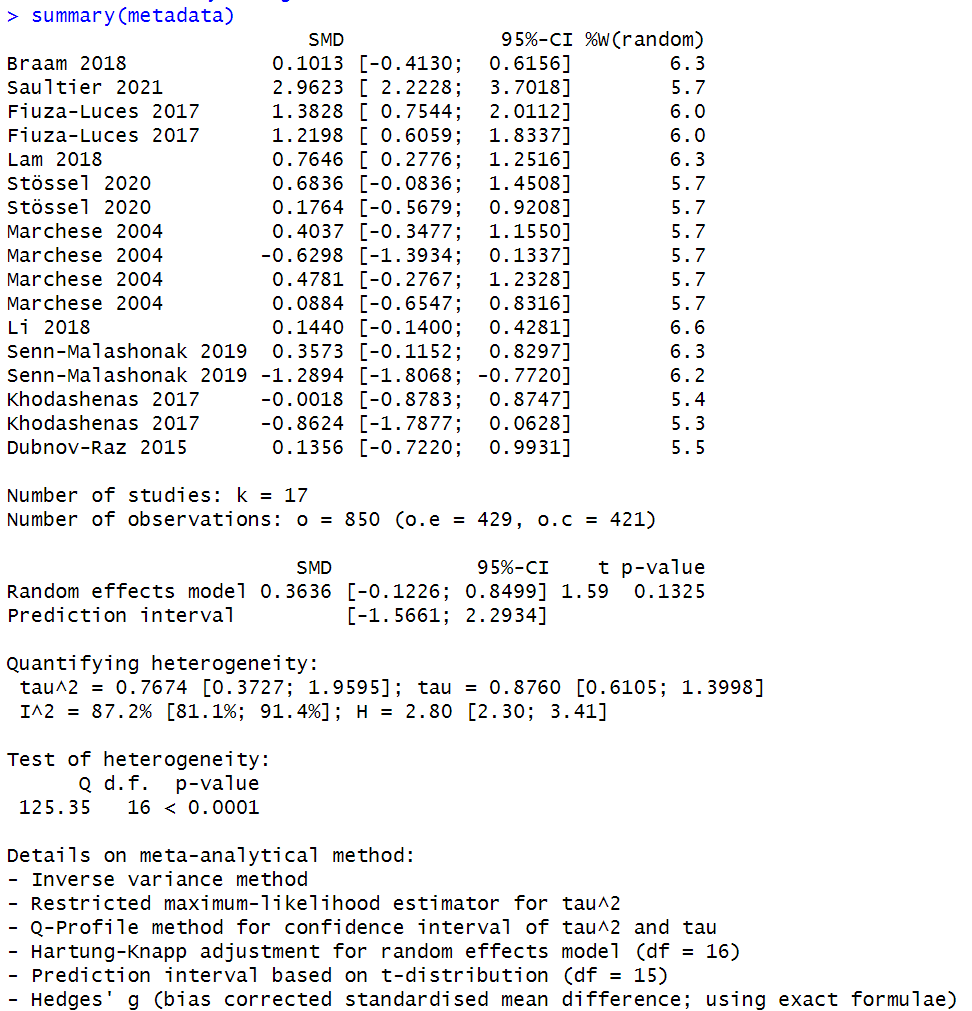


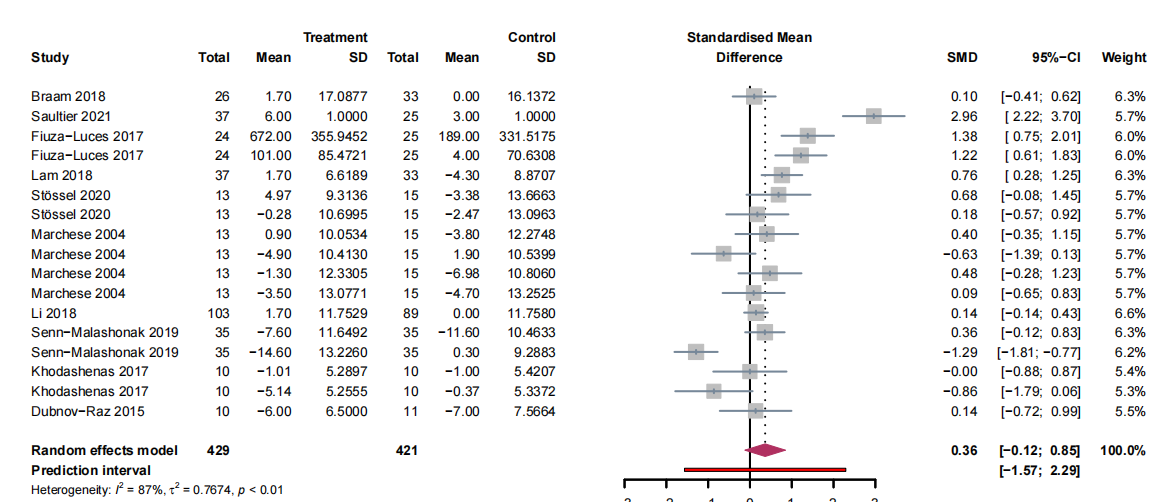


**5.2 Quality of life - fatigue**


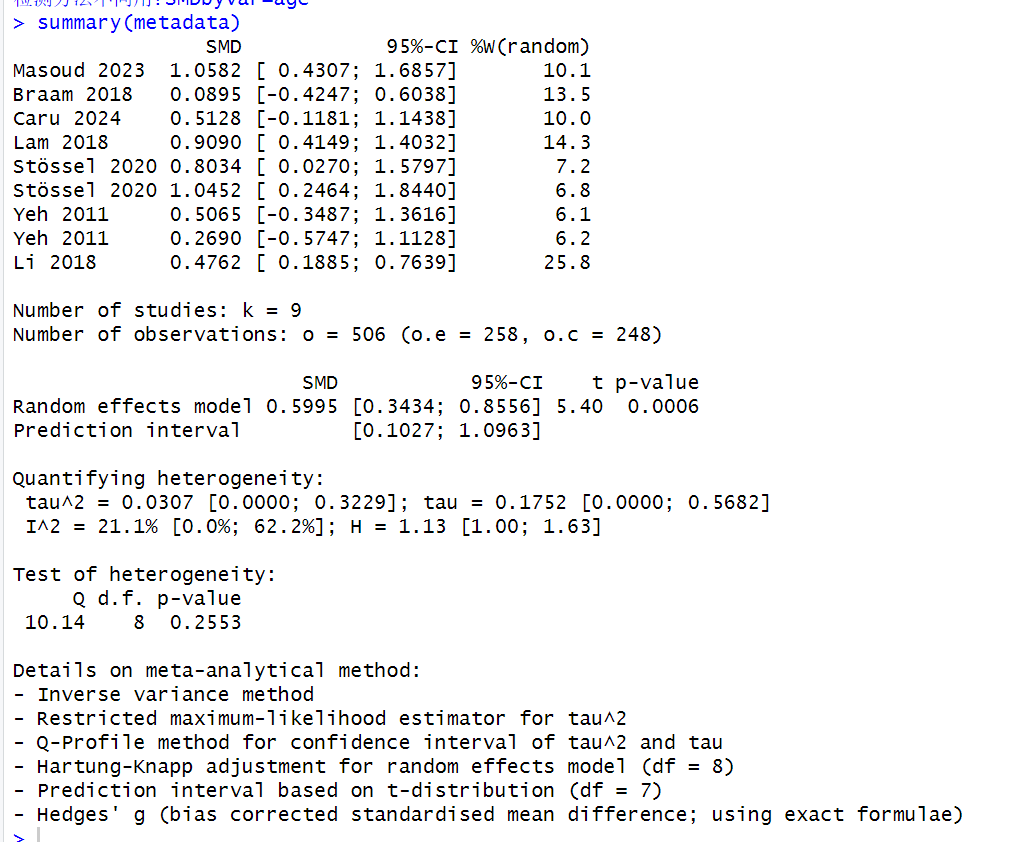


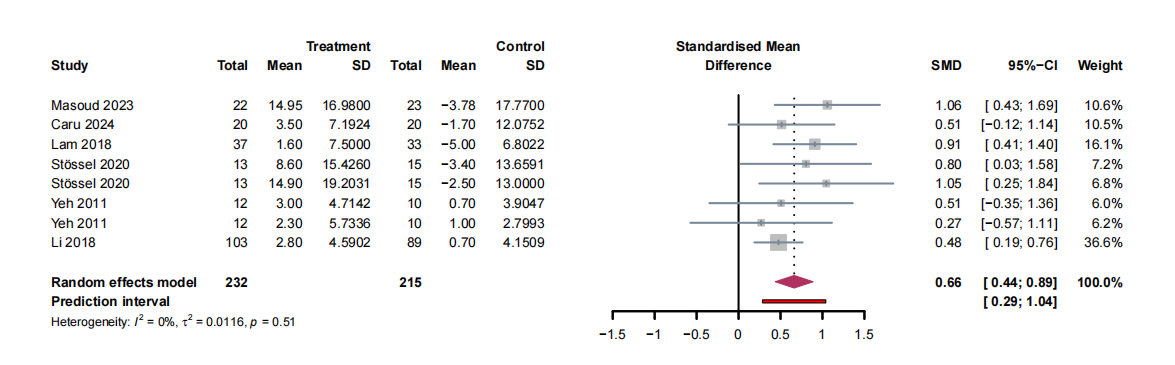


**5.3 Quality of life**

**
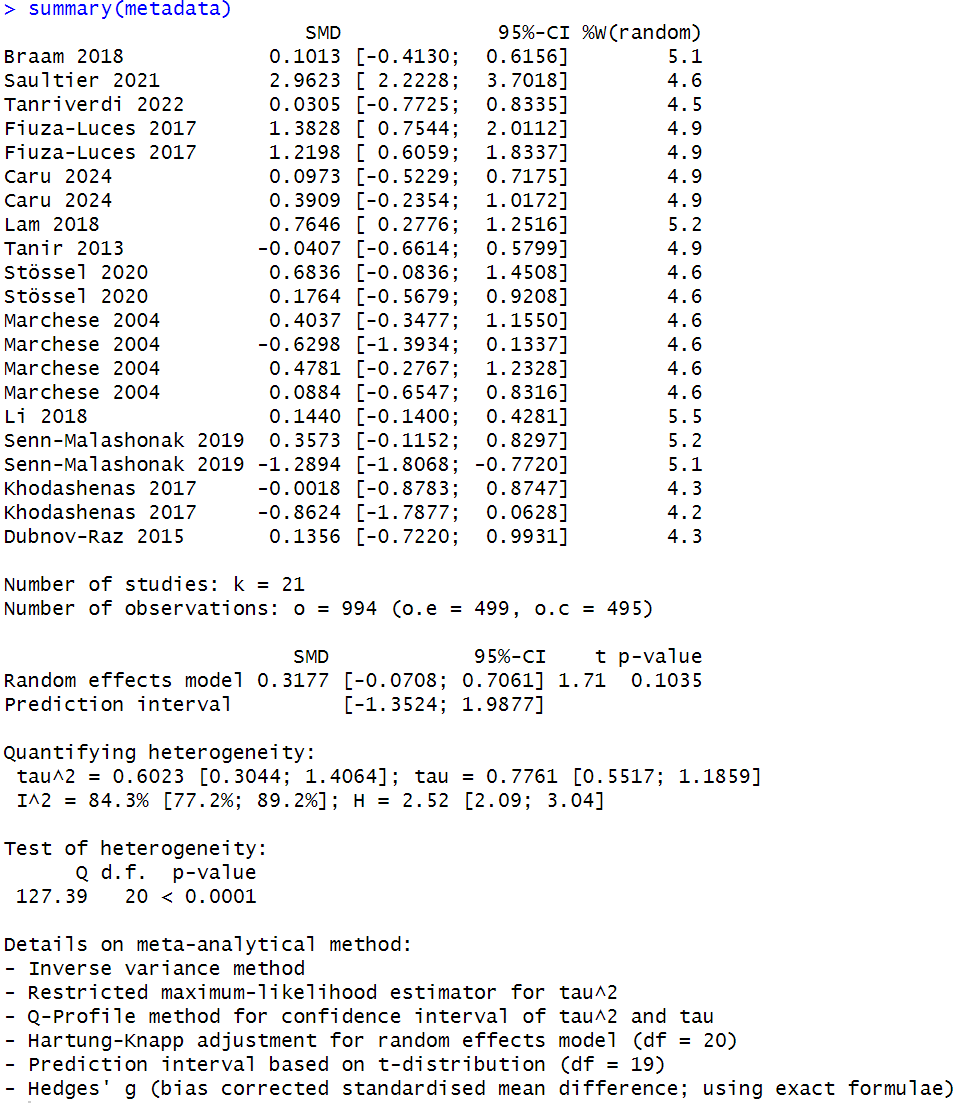
**

**
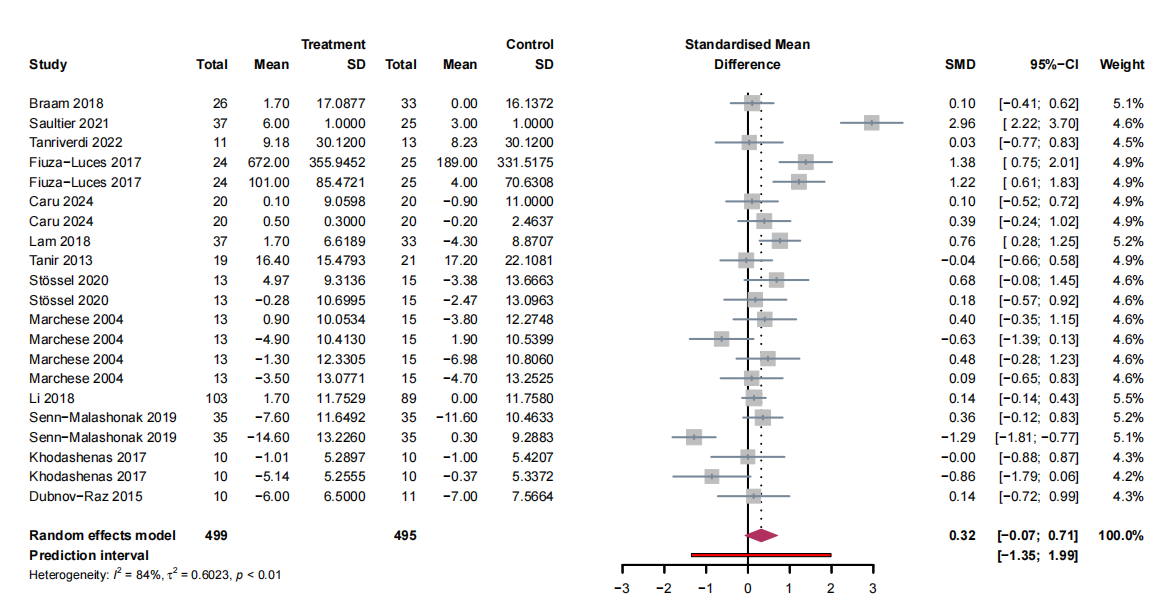
**

**5.4 Exercise capacity - lower body muscle strength**


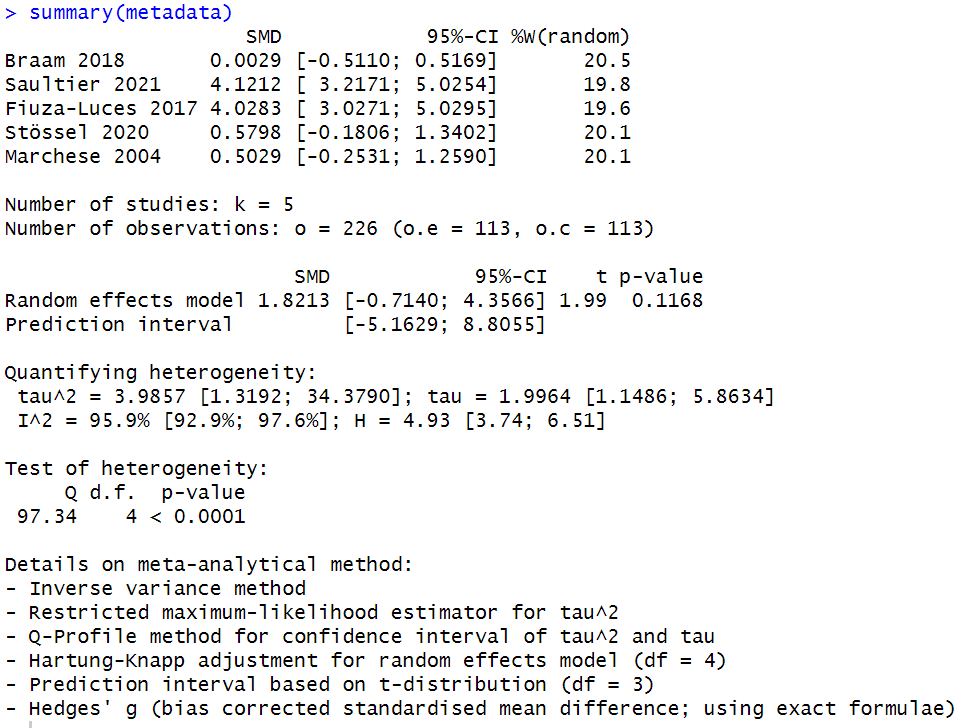


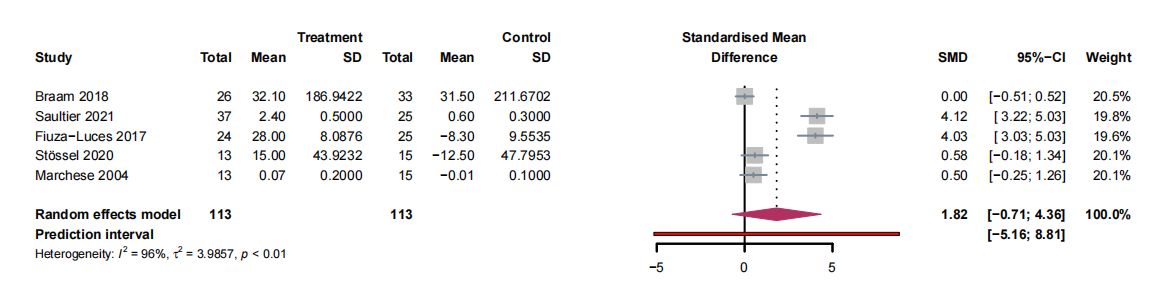


**5.5 Exercise capacity - upper body muscle strength**


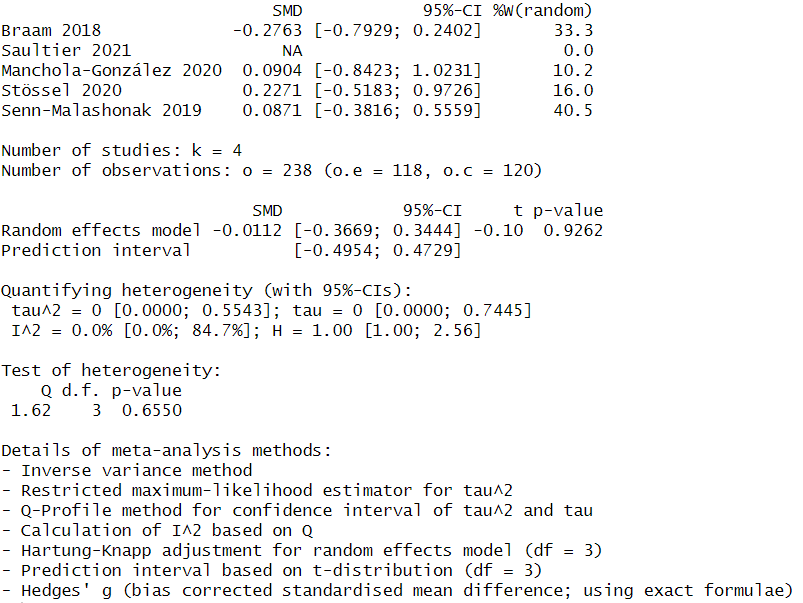


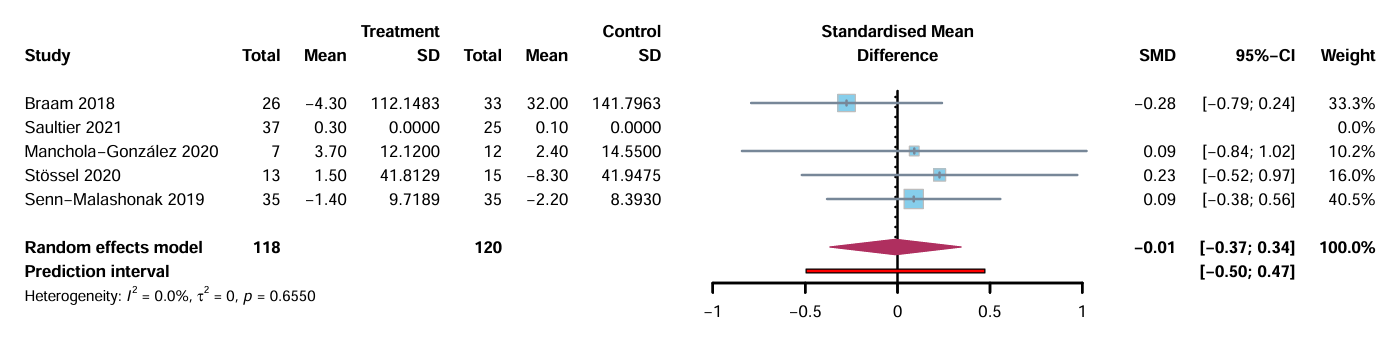


**5.6 Exercise capacity - trunk muscle strength**


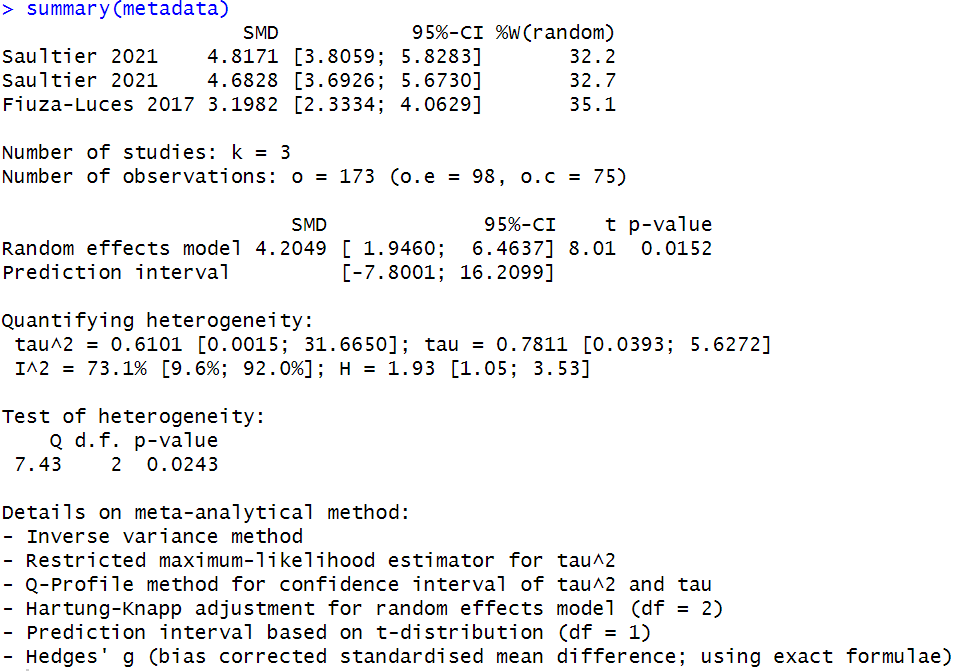


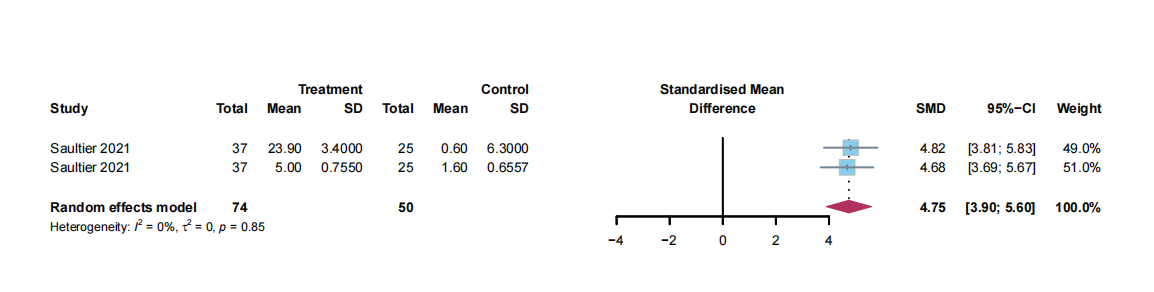


**5.7 Exercise capacity - muscle strength**


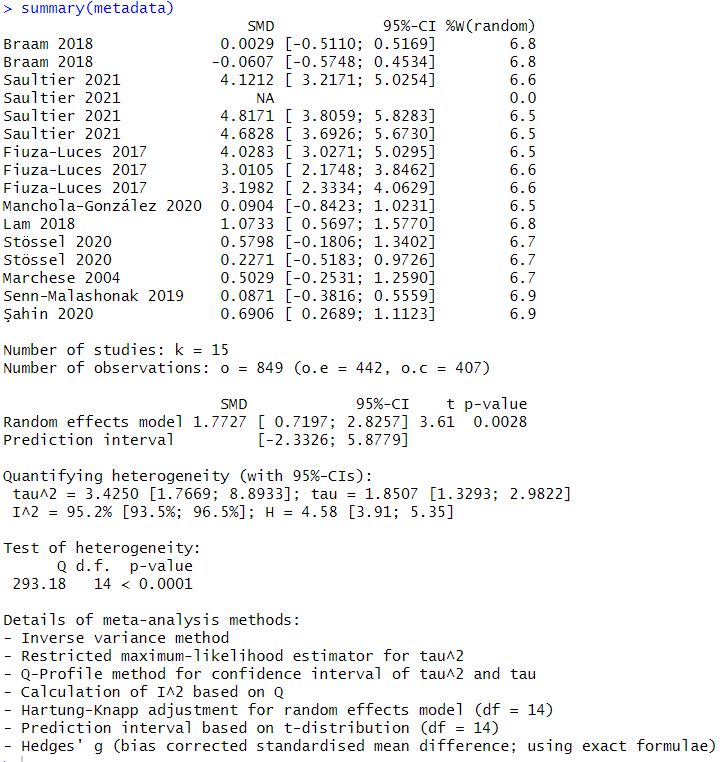


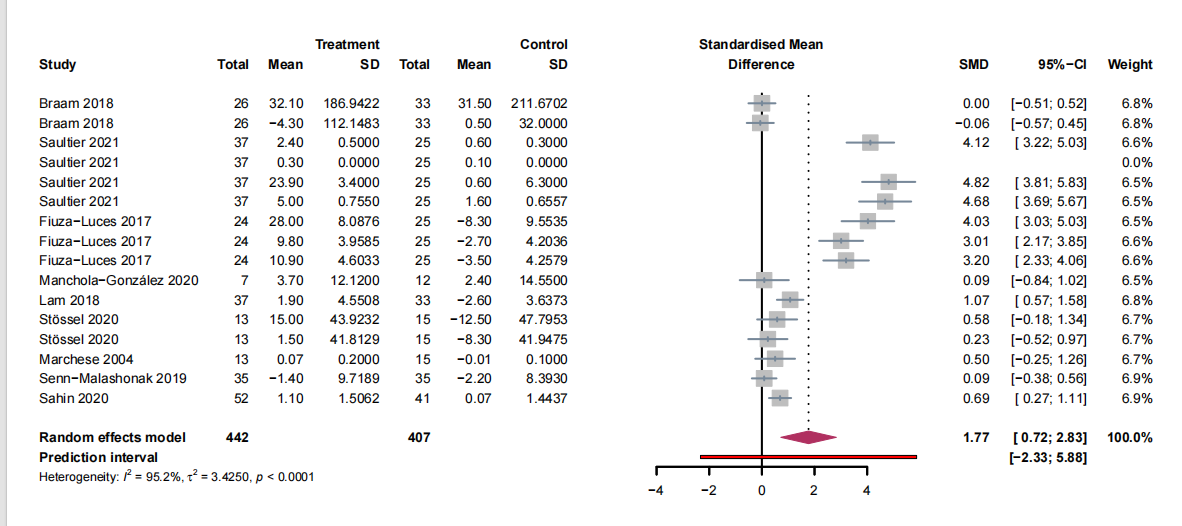


**5.8 Exercise capacity - balance**


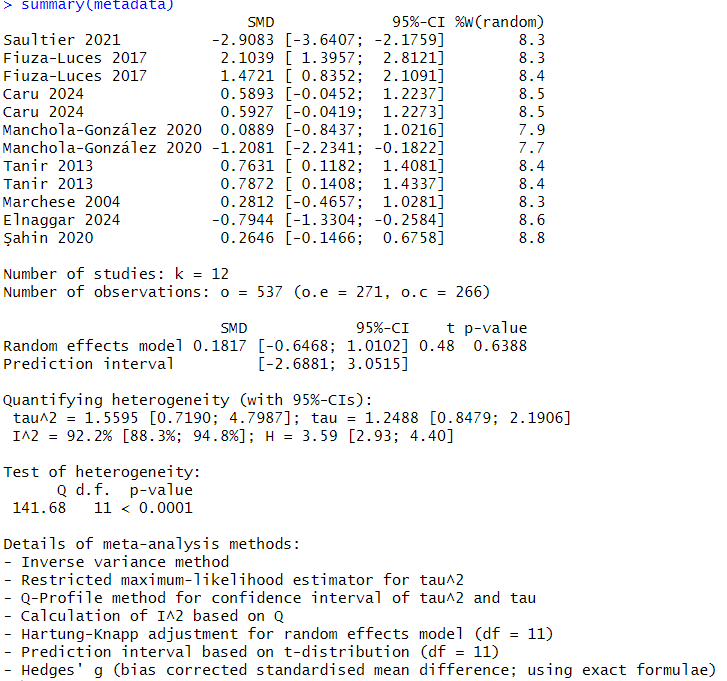


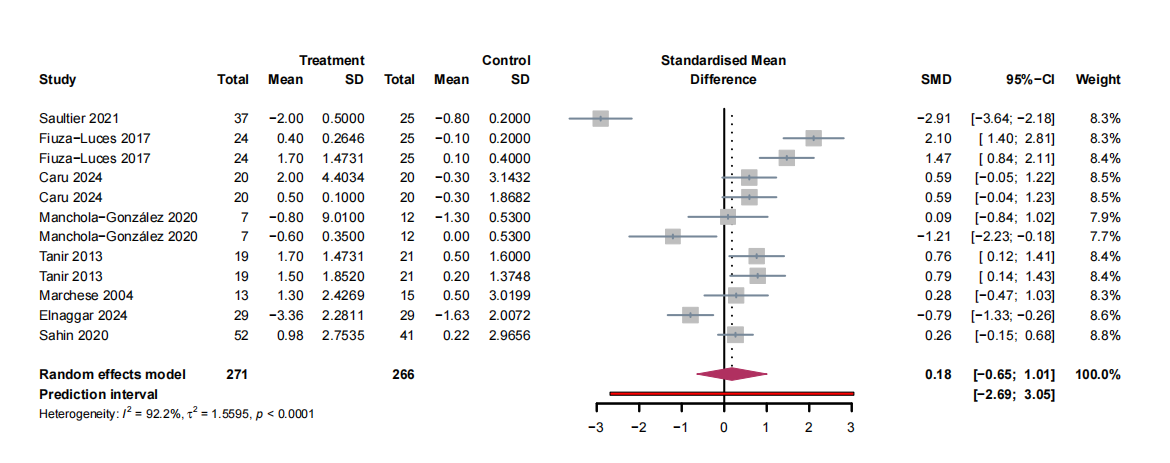


**5.9 Exercise capacity - flexibility**


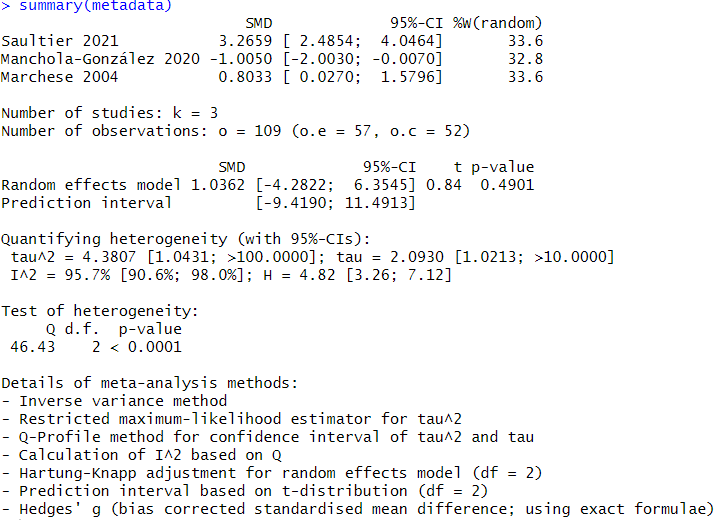


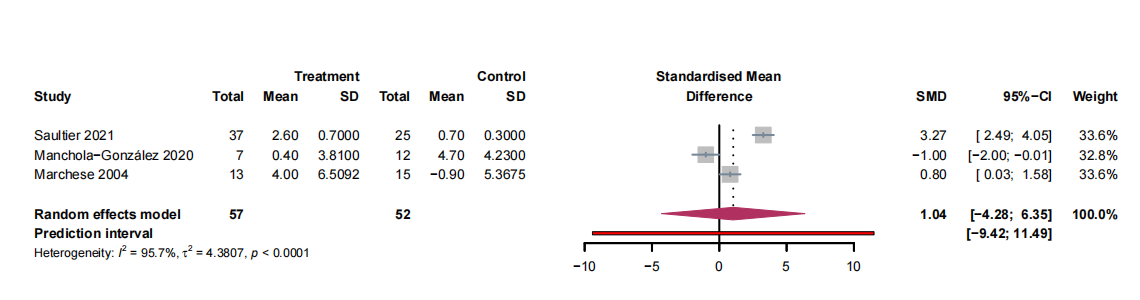


**5.10 Exercise capacity - athletic performance**


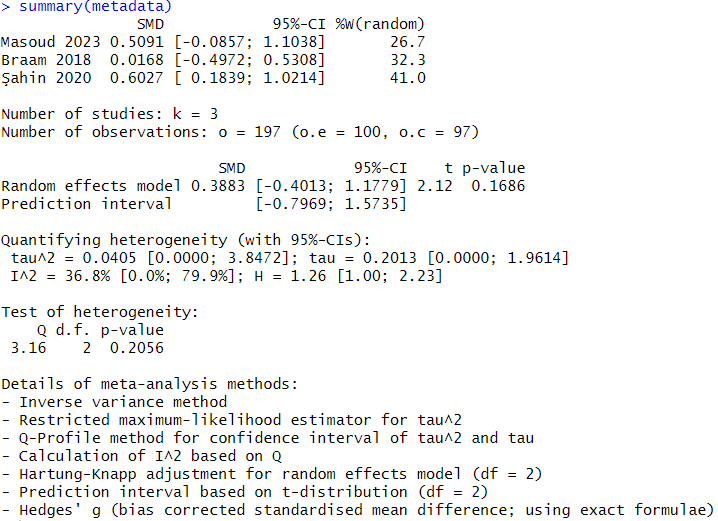


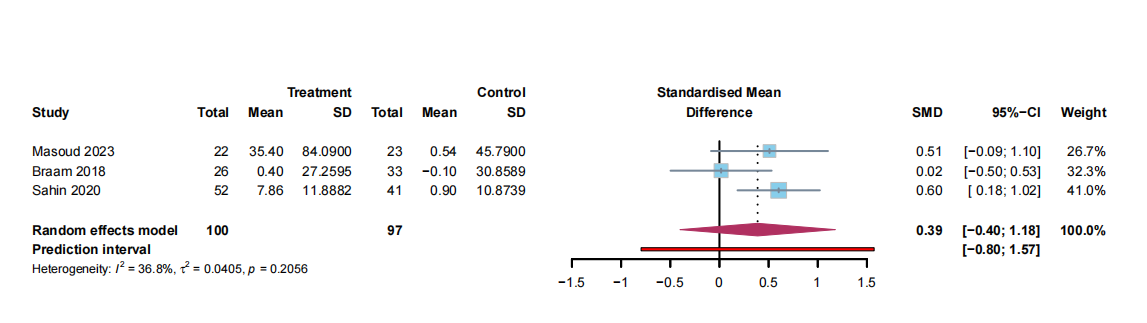


**5.11 Exercise capacity - physical activity behaviour**


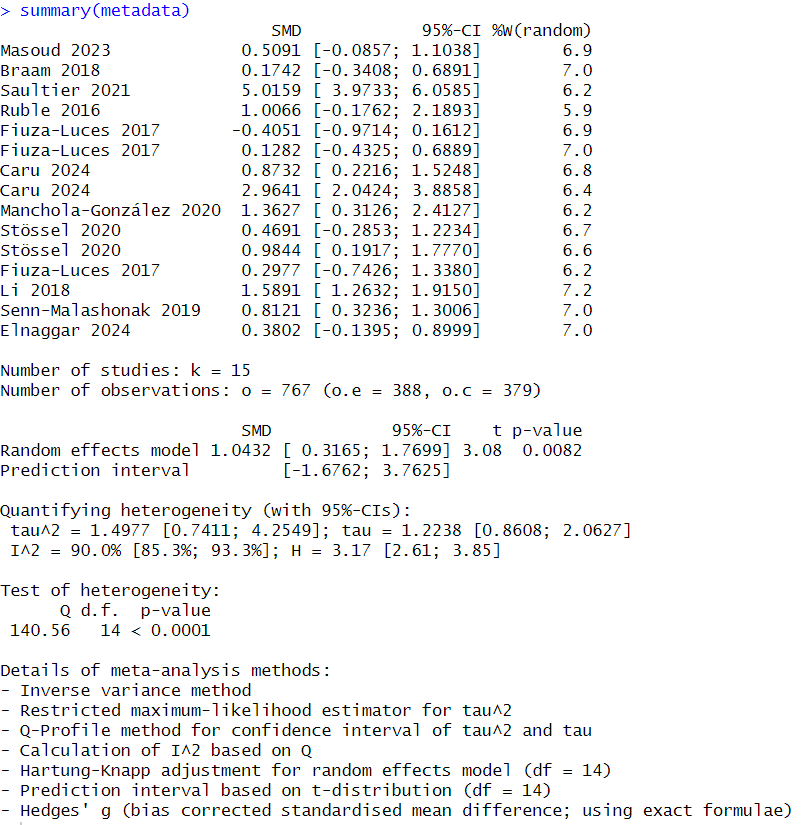


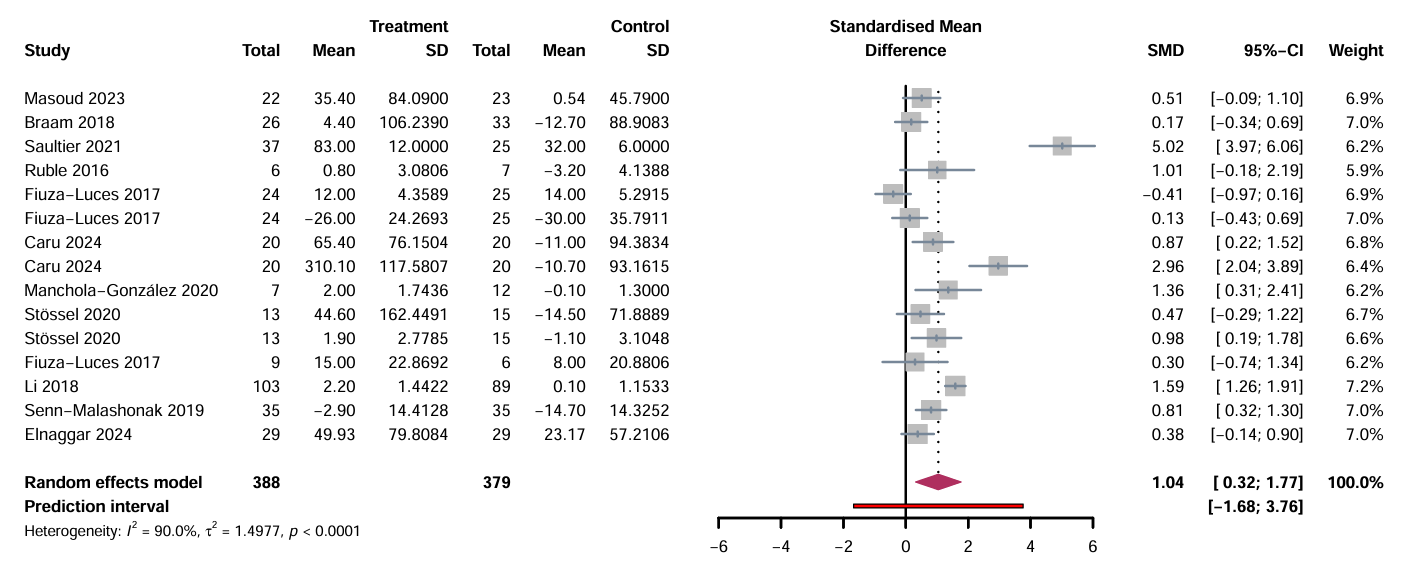


**5.12 Exercise capacity - physical activity level**


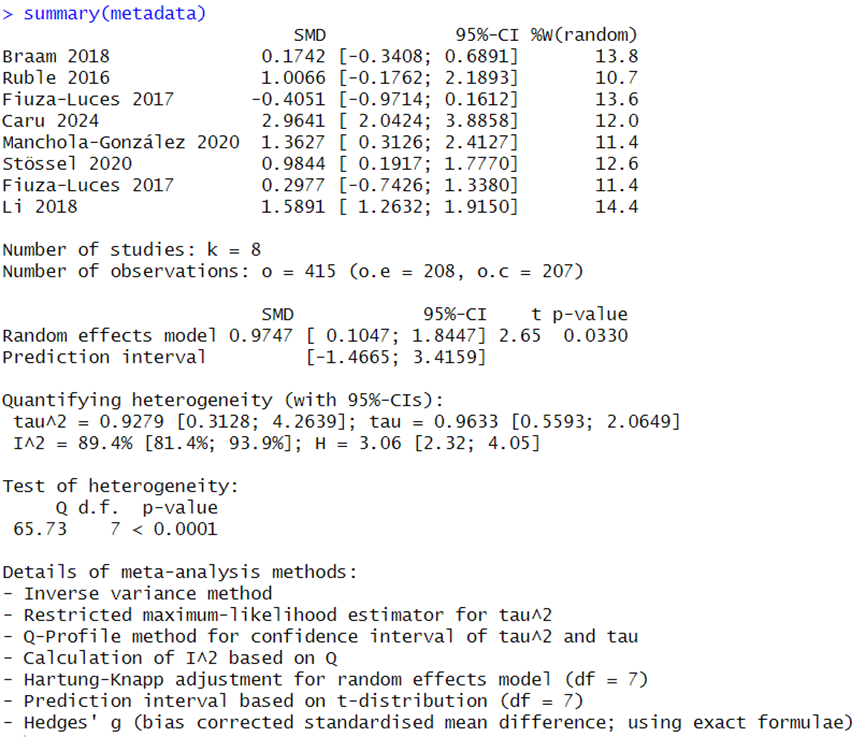


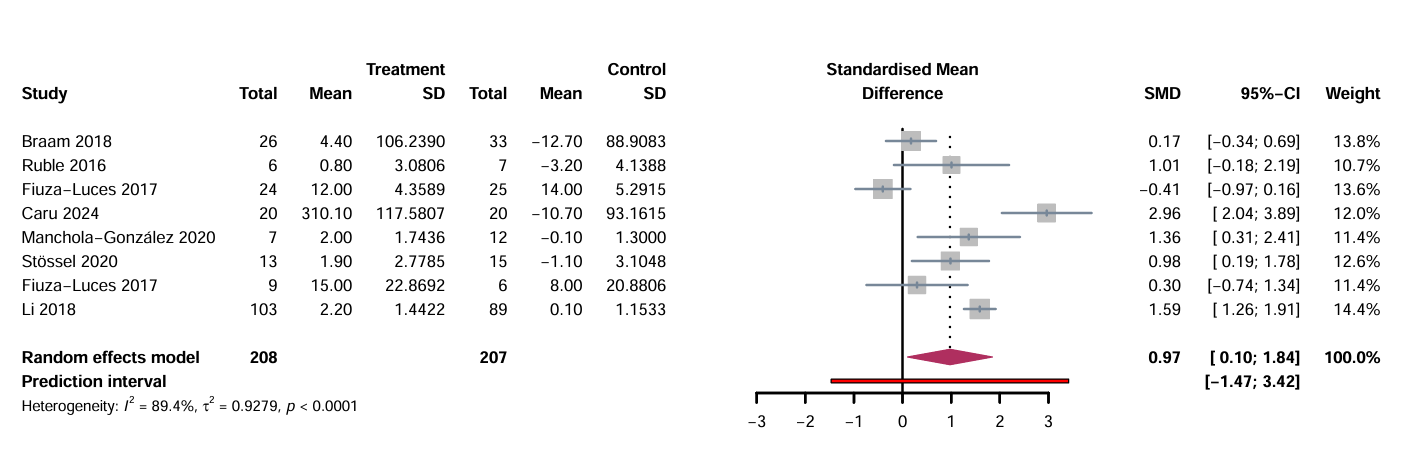


**5.13 Exercise capacity - cardiorespiratory function**


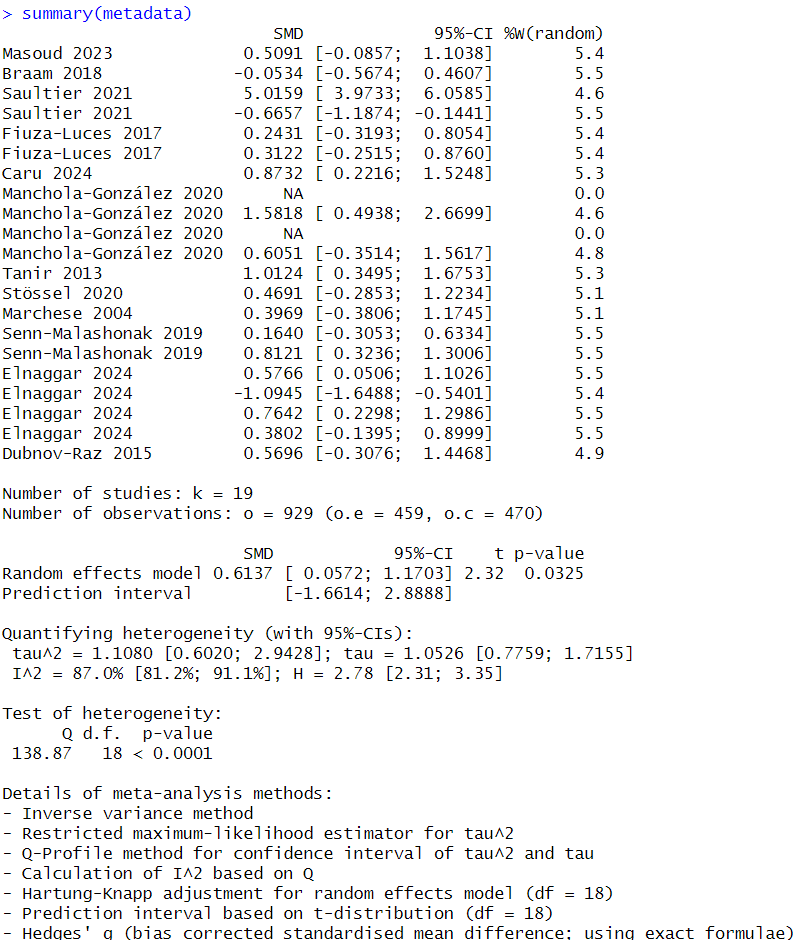


**5.14 Exercise capacity - peak oxygen uptake**


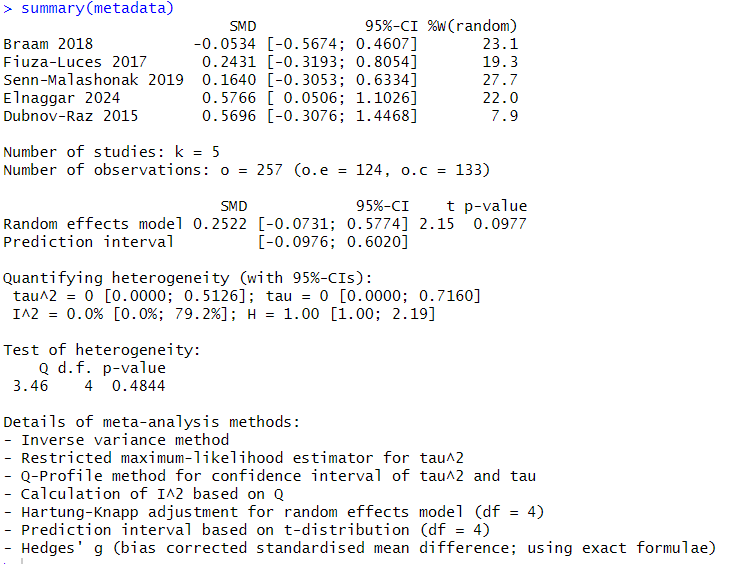


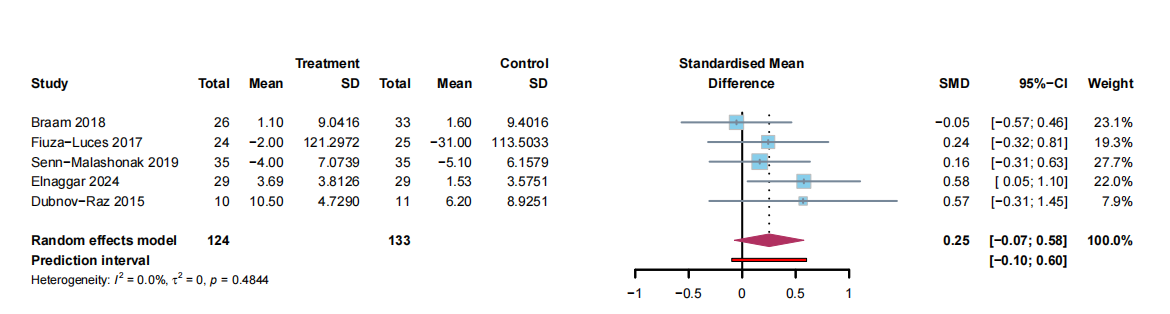


**5.15 Exercise capacity - six-minute walk test**


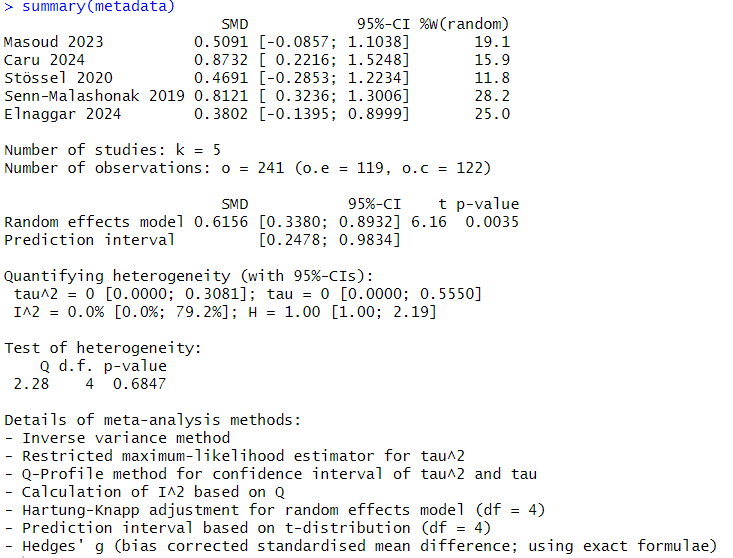


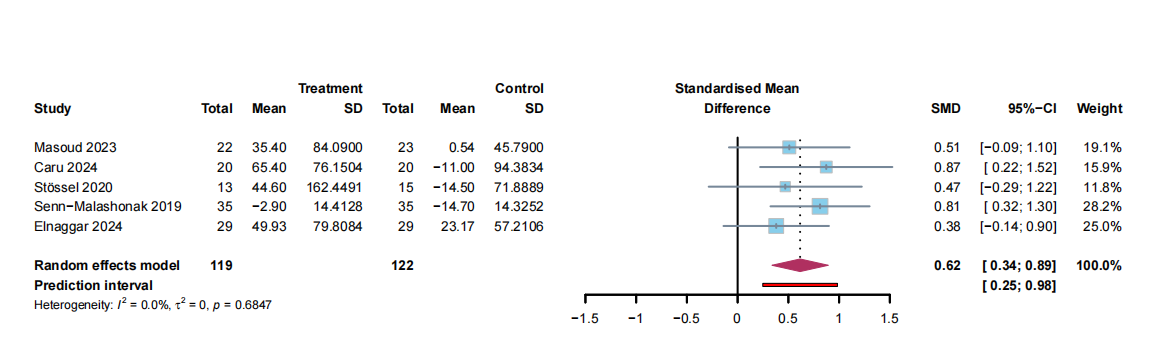


**5.16 Cognitive function**


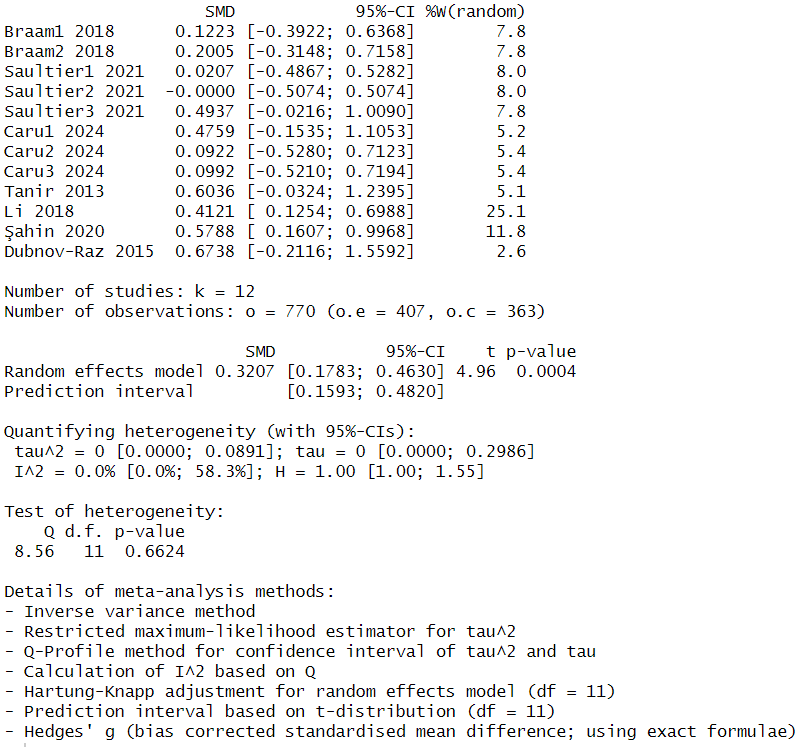


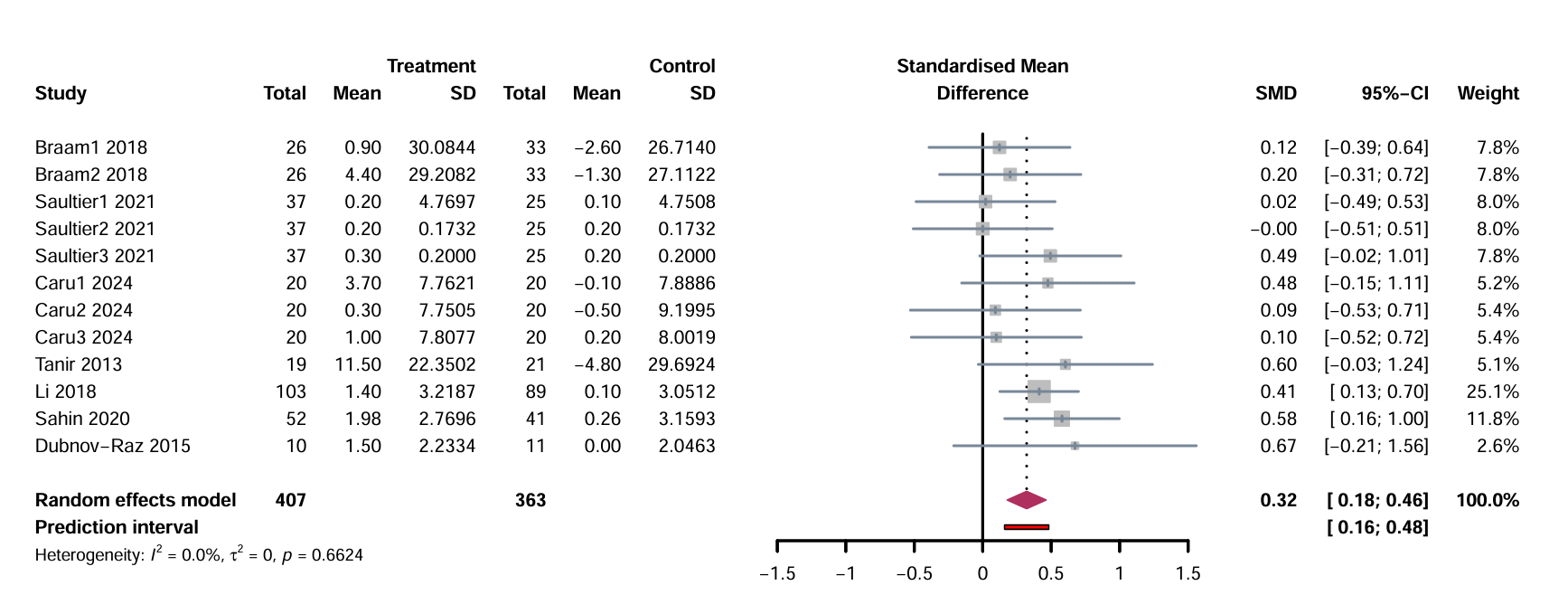


**5.17 Cognitive function - executive functions**


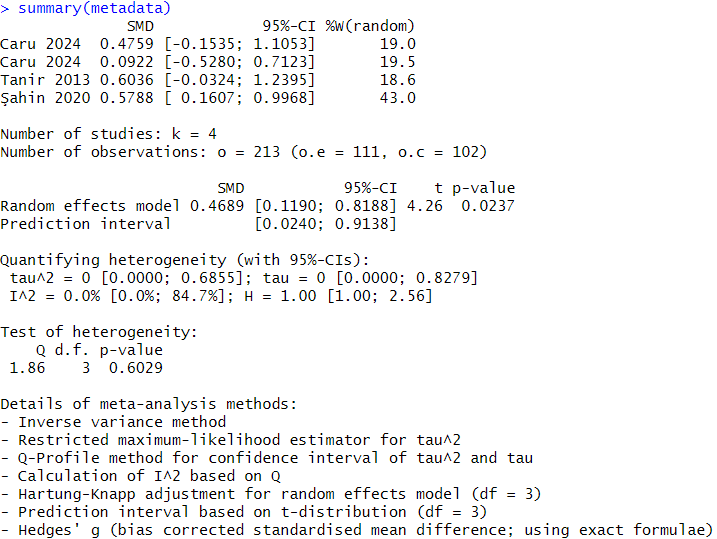


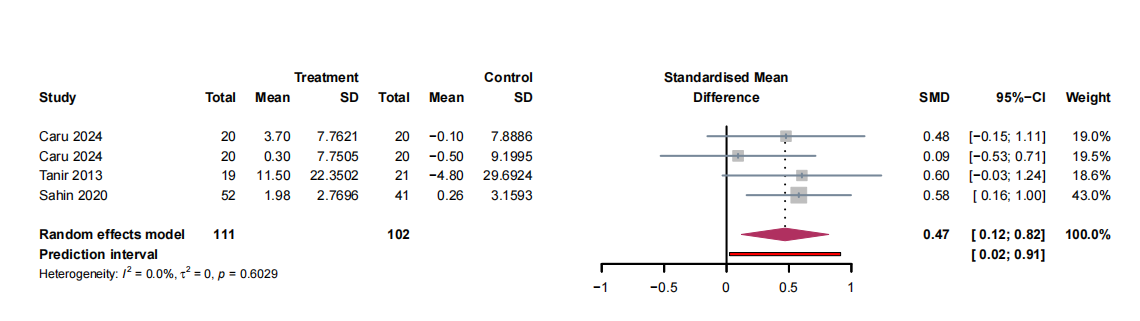


**5.18 Cognitive function - depressive symptoms**


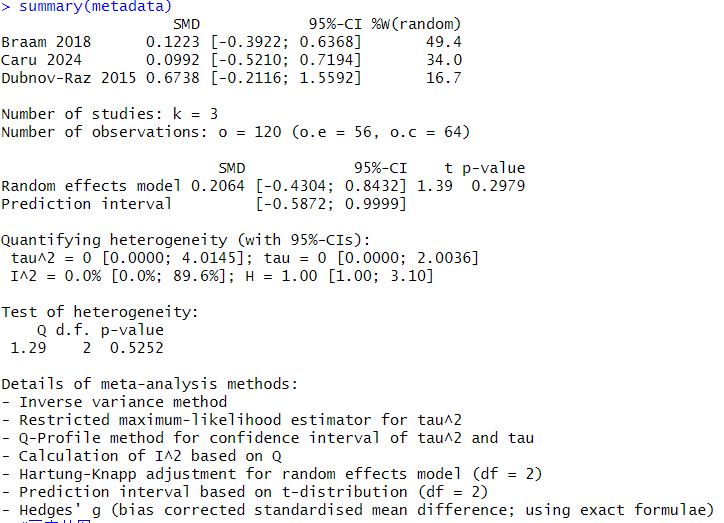


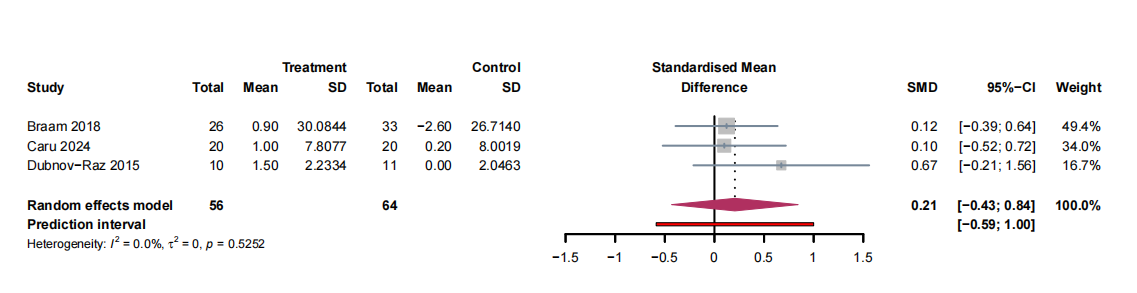


**5.19 Social function**


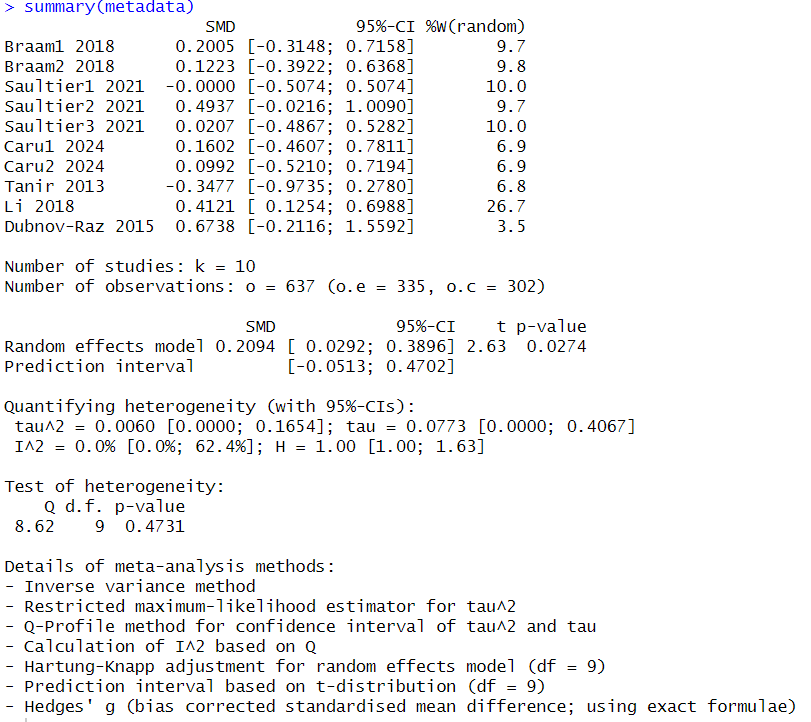


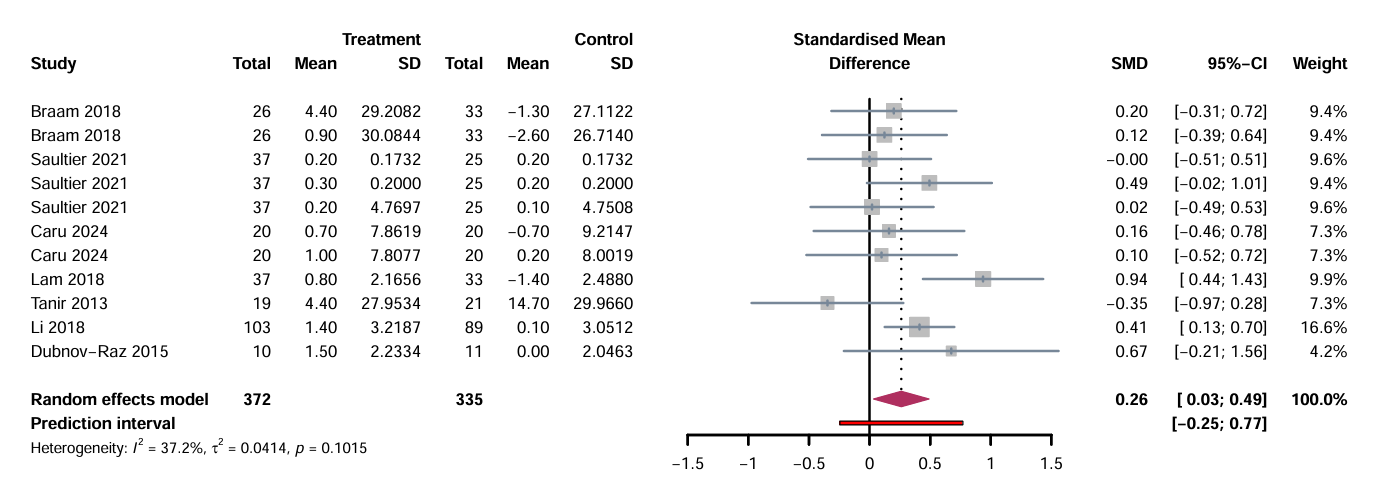


**5.20 Body composition - bone mineral density**


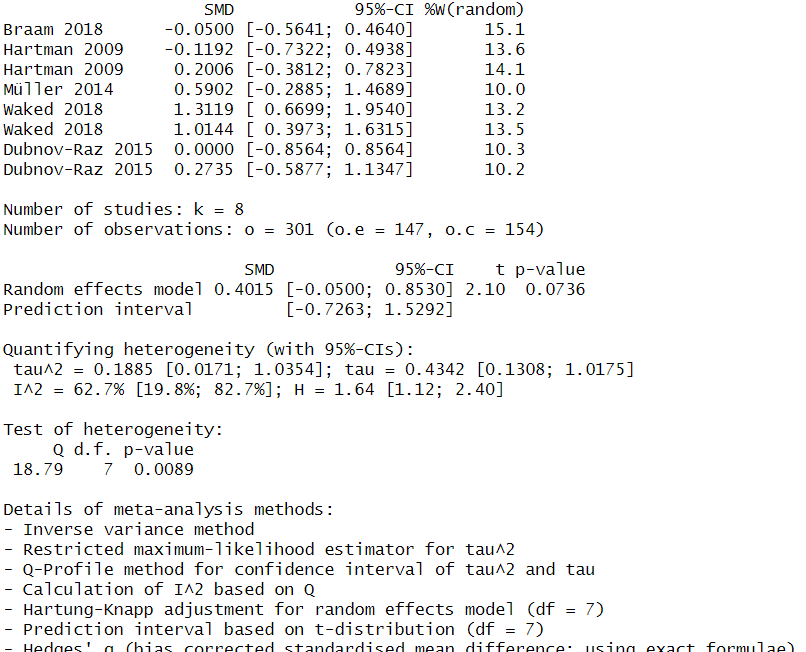


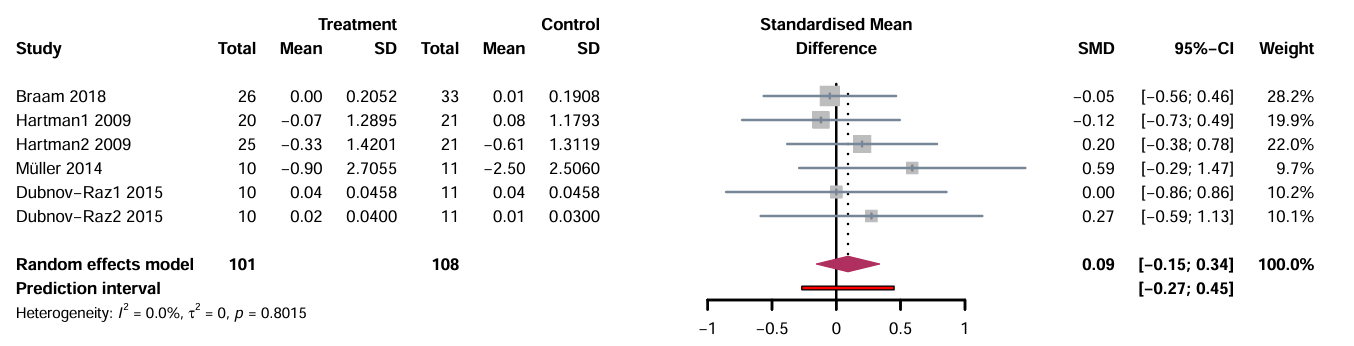


**5.21 Body composition - body mass index**


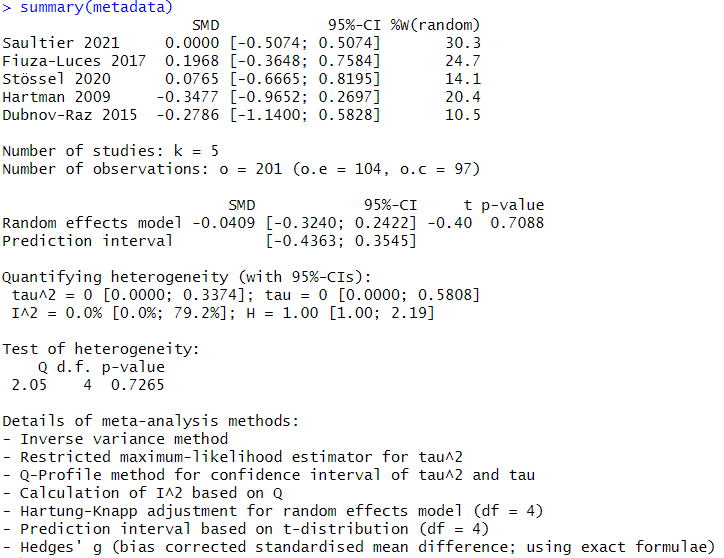


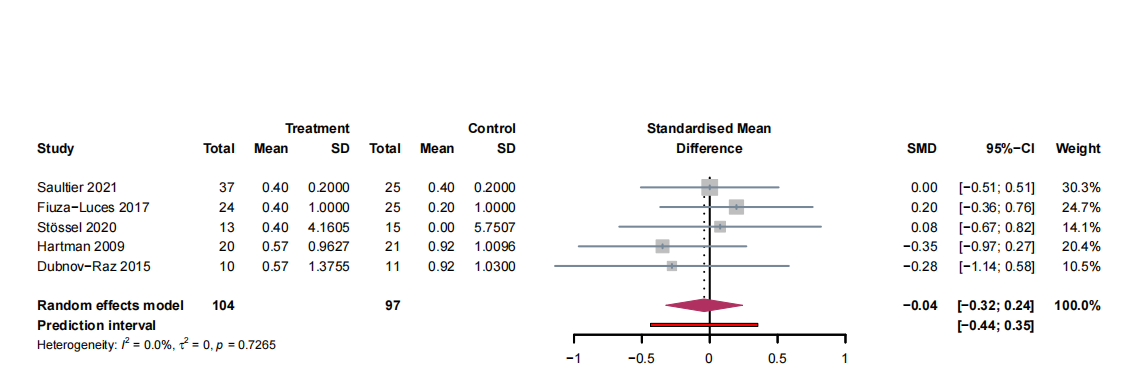


**5.22 Body composition - fat mass percentage**


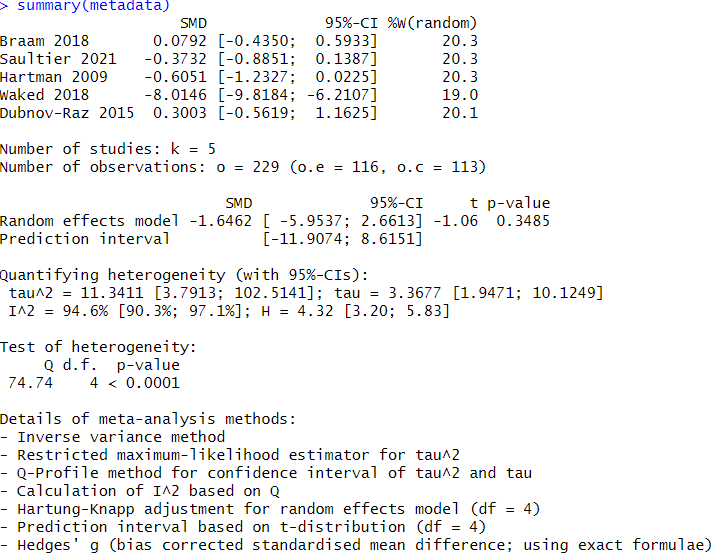


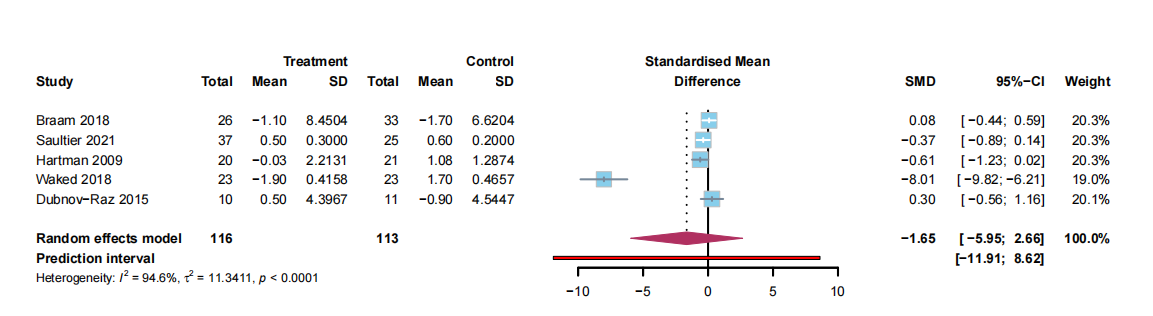


**5.23 Body composition - NK cell level**


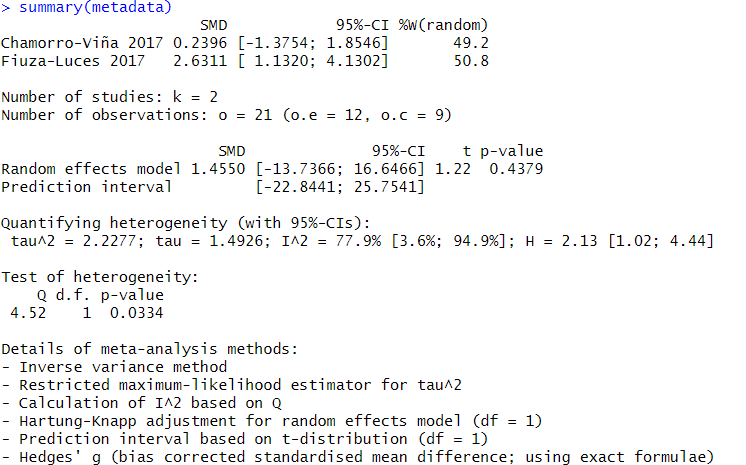


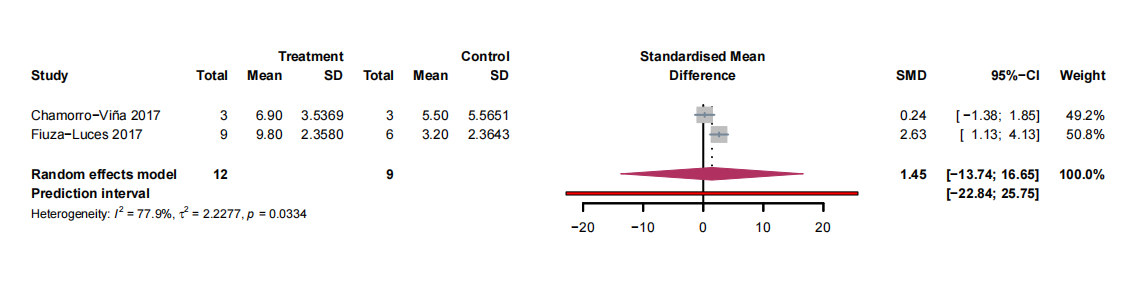


**5.24 Body composition - pro-inflammatory factor**


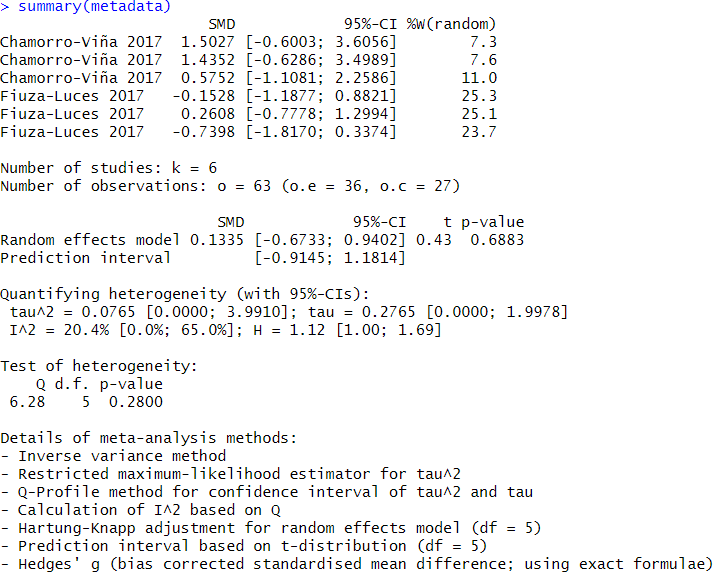


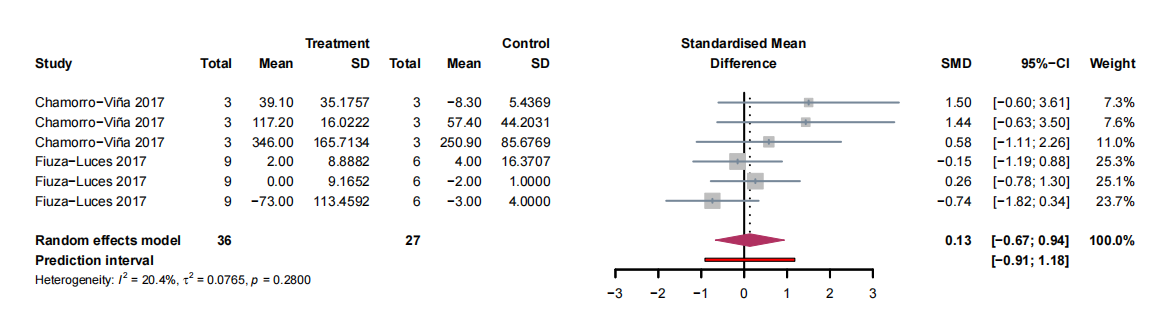


**5.25 Body composition – anti-inflammatory factor**


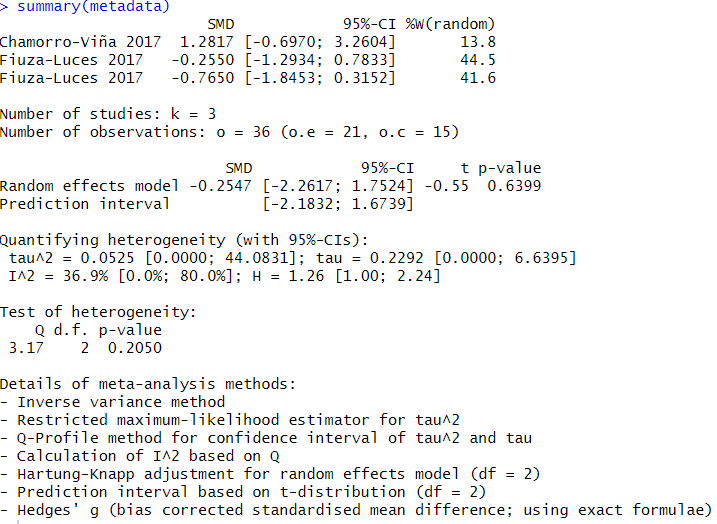


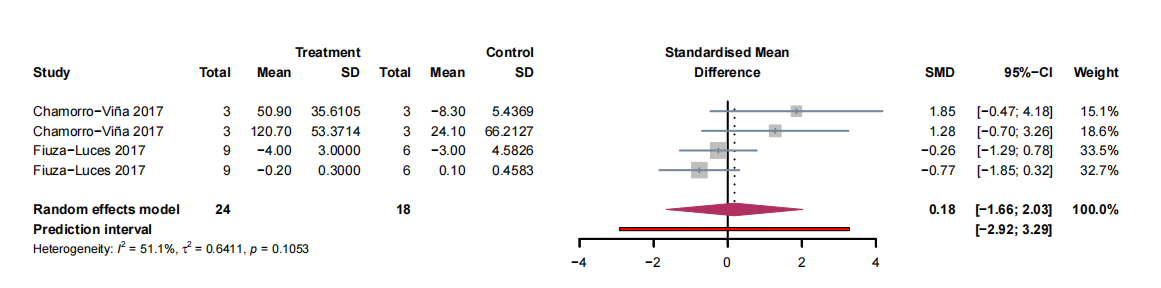


**6. Sensitivity Analysis**

**6.1 Sensitivity analysis based on quality of life scale**


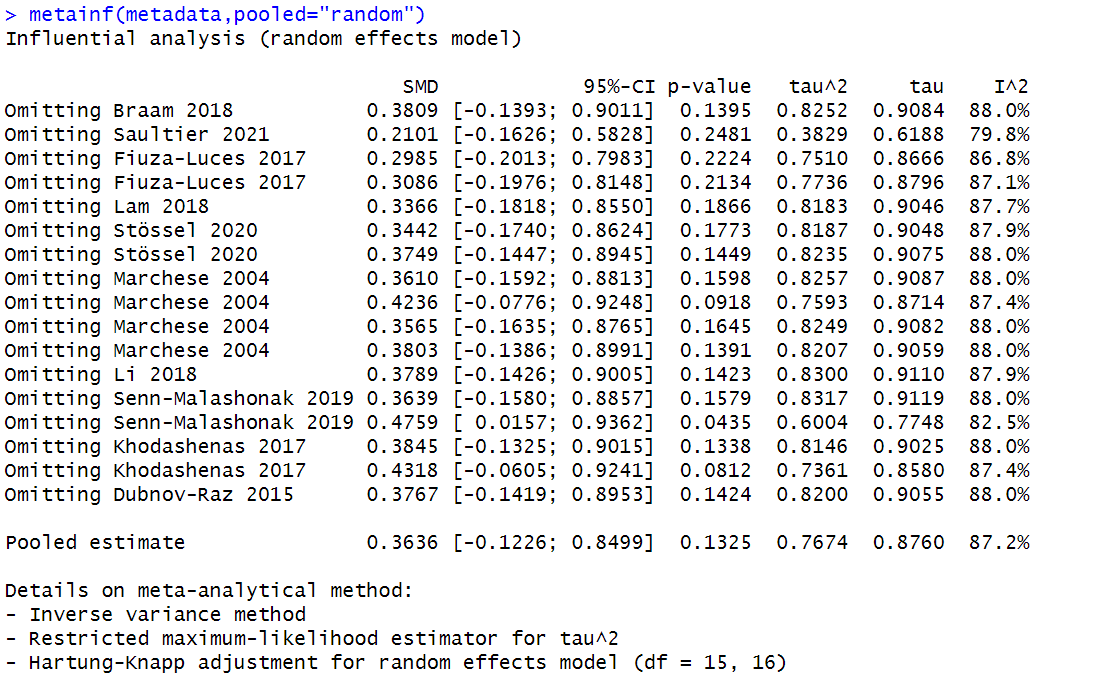


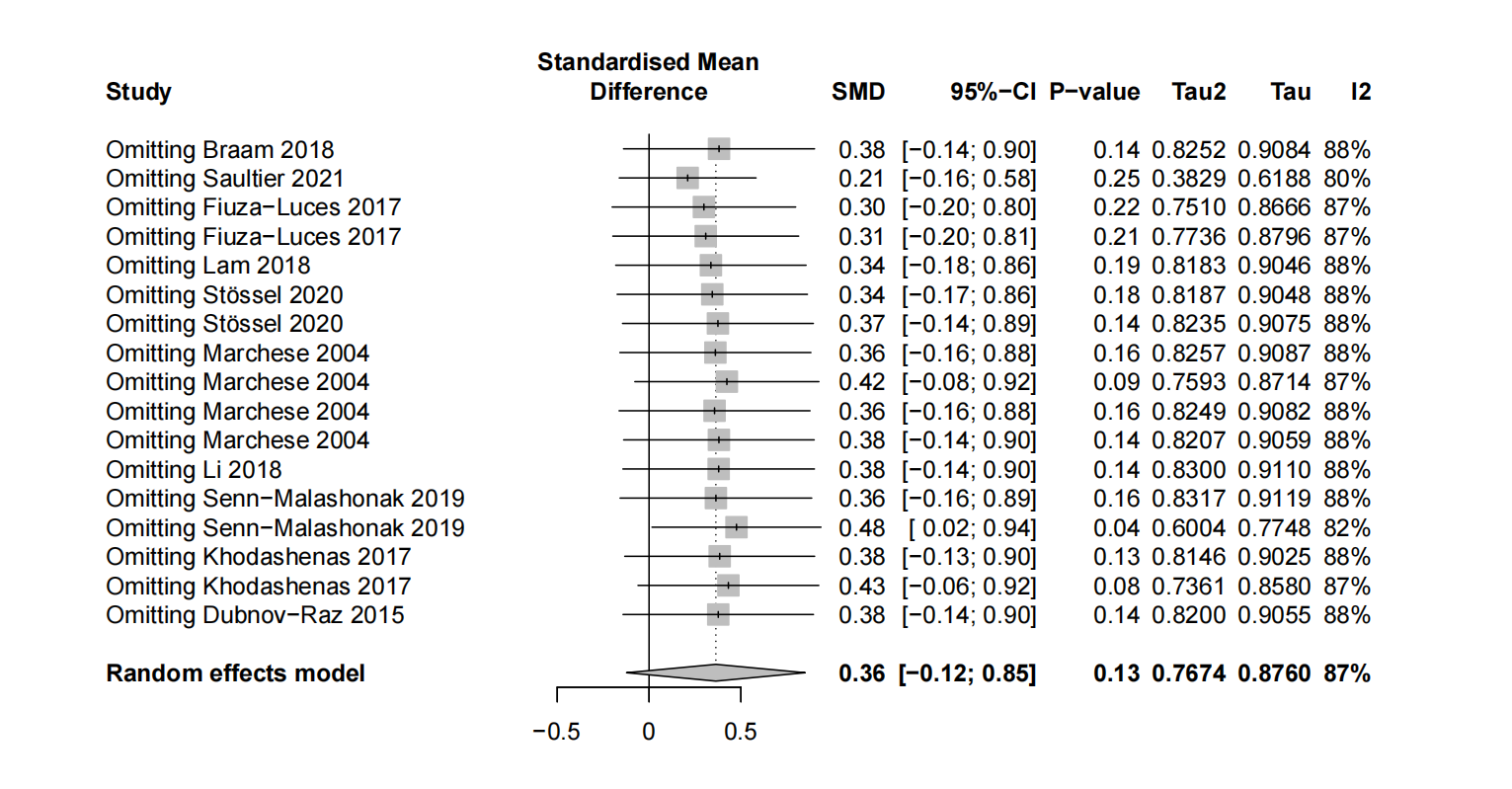


**6.2 Sensitivity analysis based on fatigue**


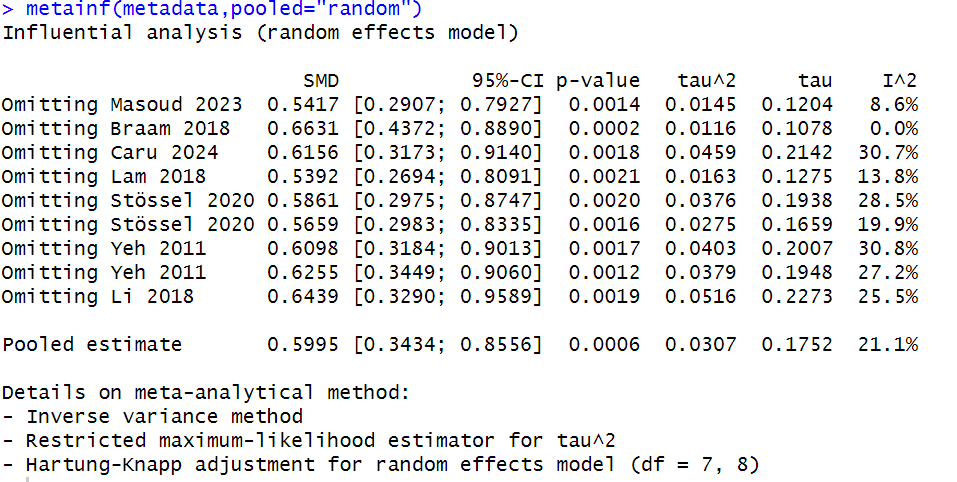


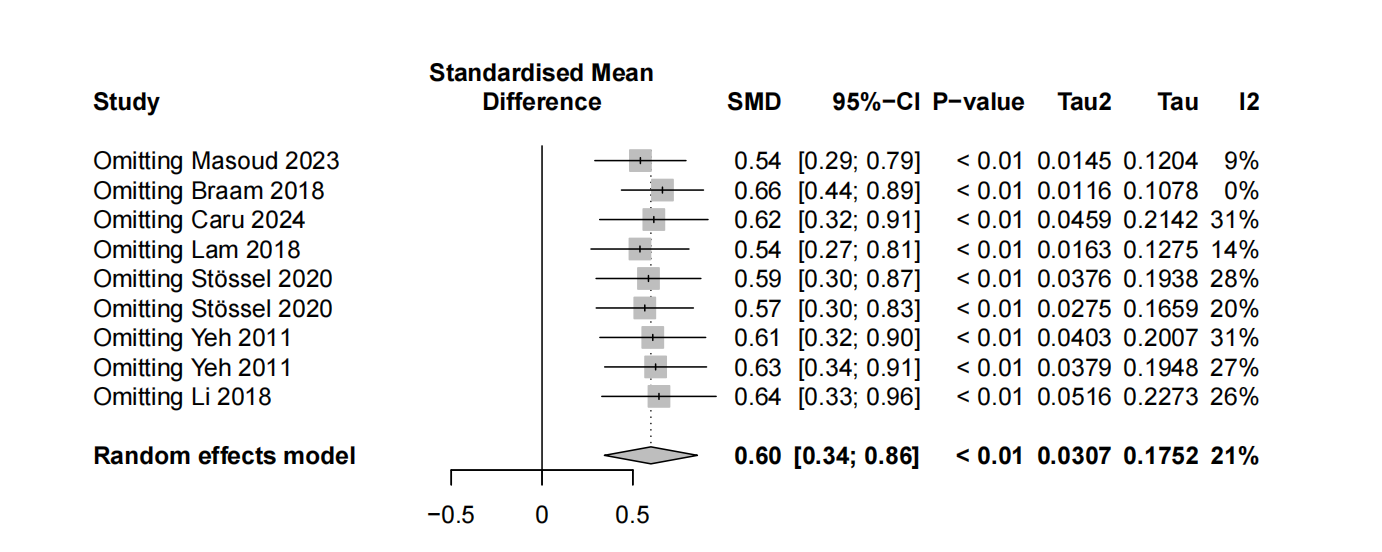


**6.3 Sensitivity analysis based on quality of life**


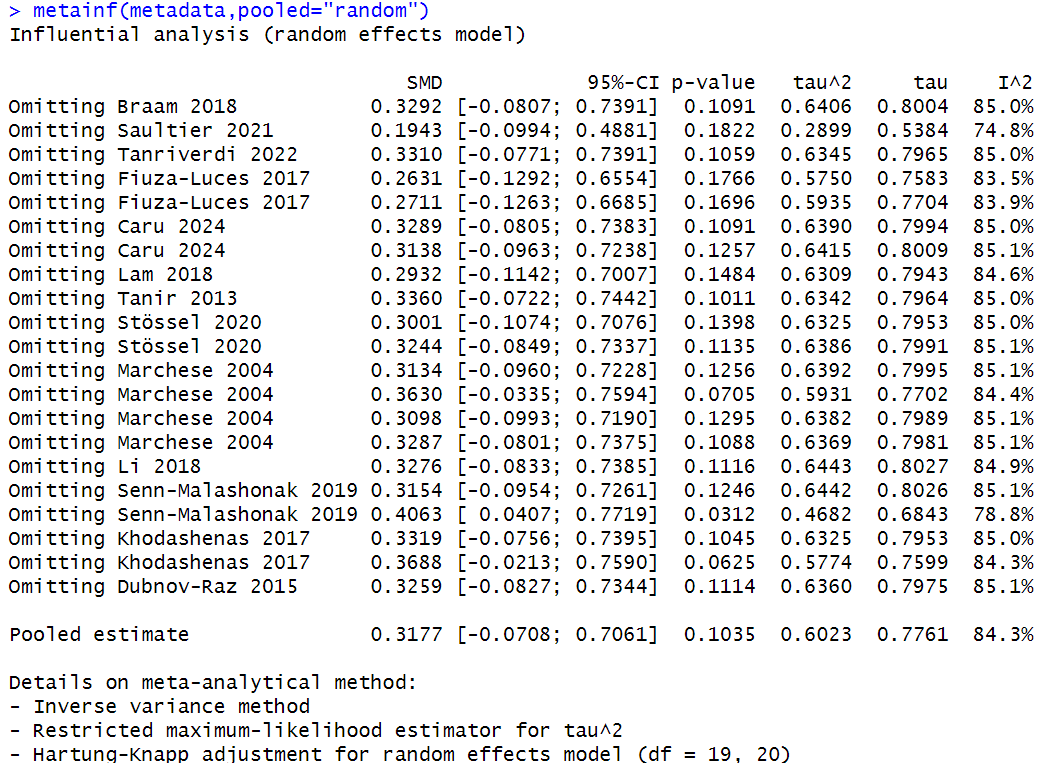


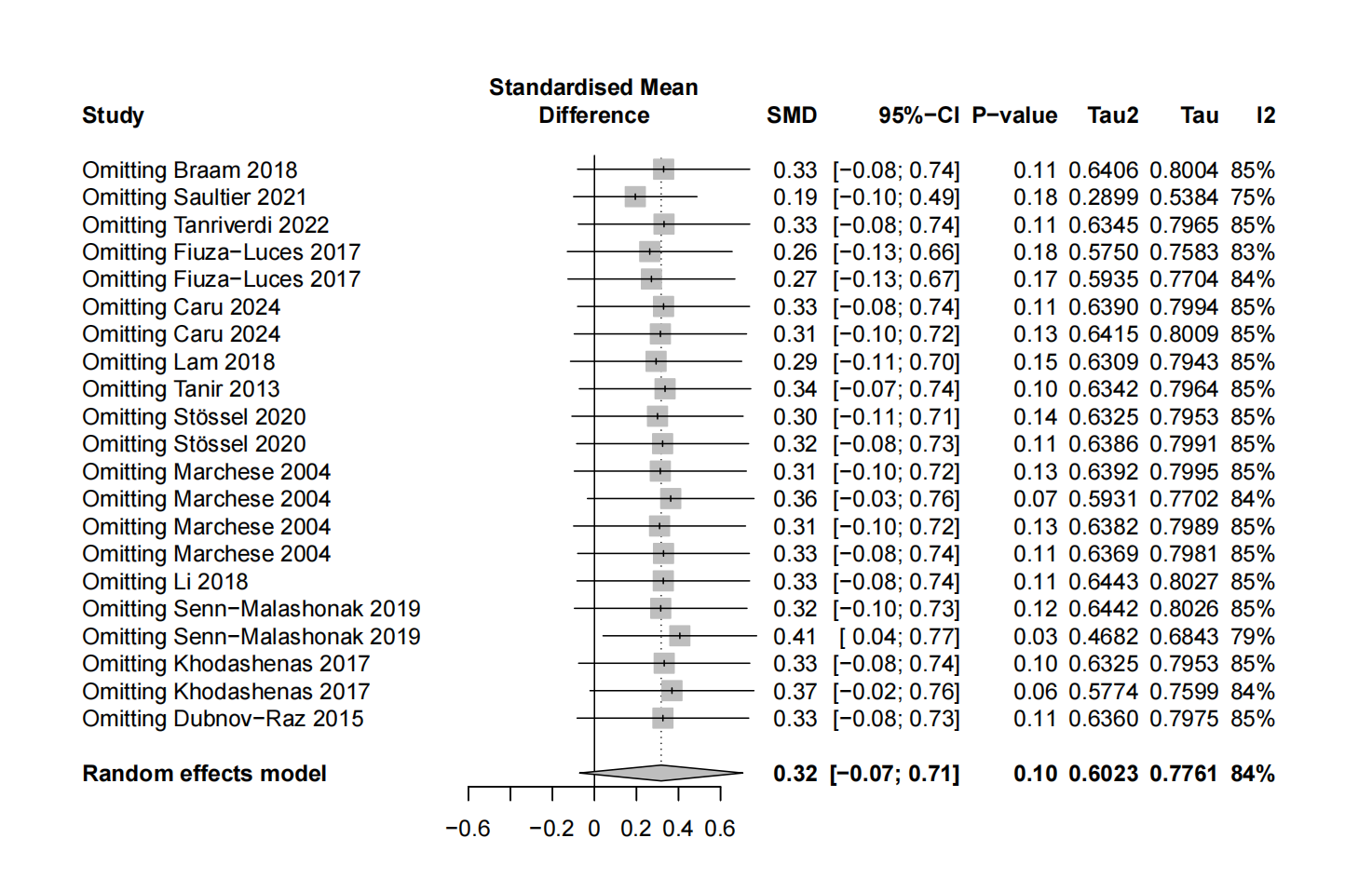


**6.4 Sensitivity analysis based on lower body muscle strength**


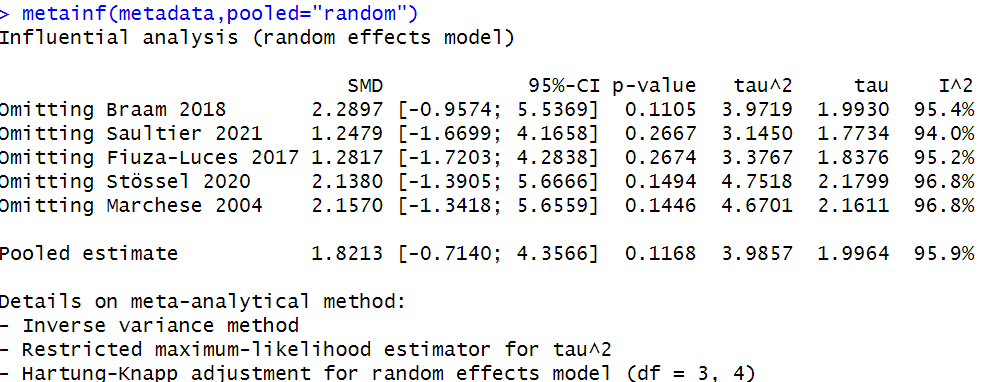


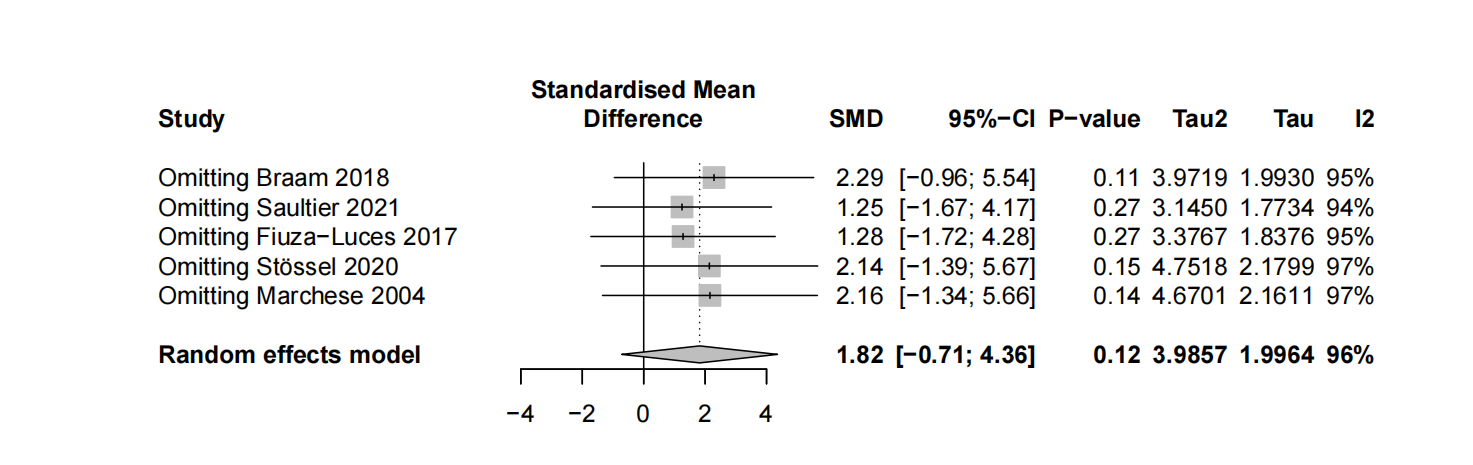


**6.5 Sensitivity analysis based on upper body muscle strength**


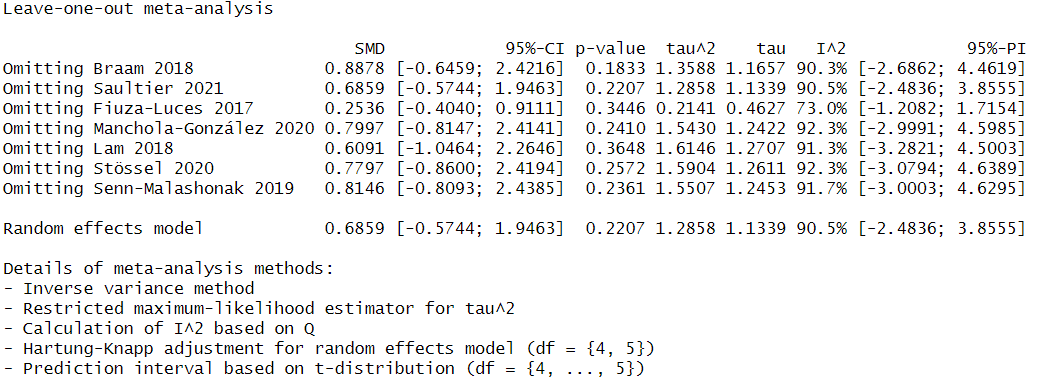


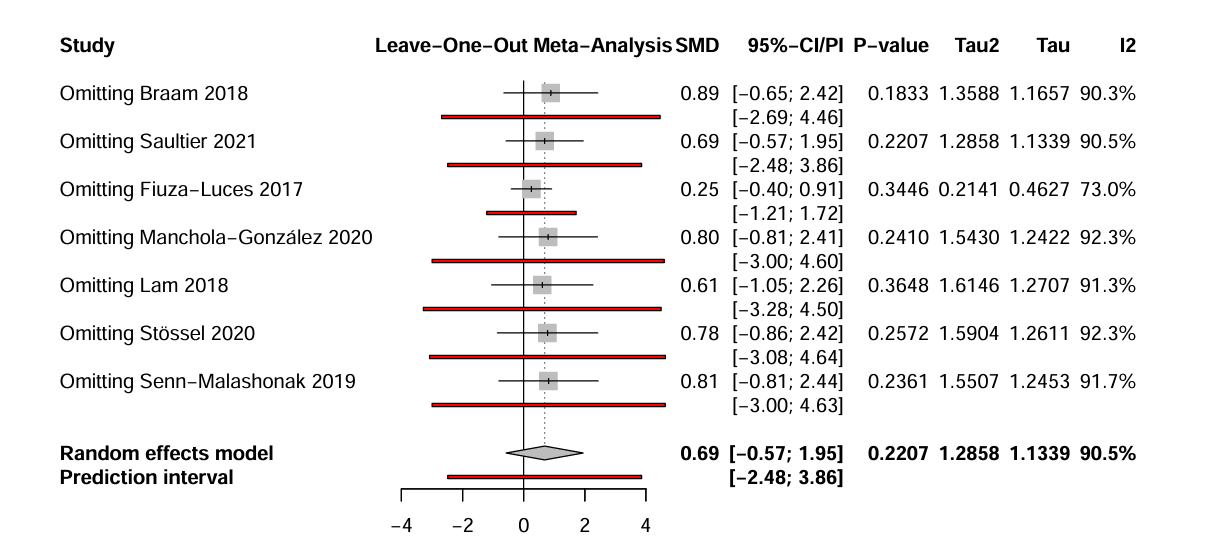


**6.6 Sensitivity analysis based on trunk muscle strength**


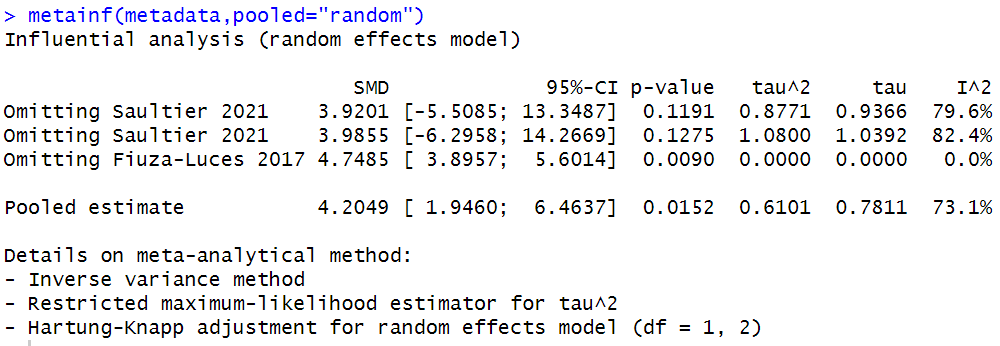


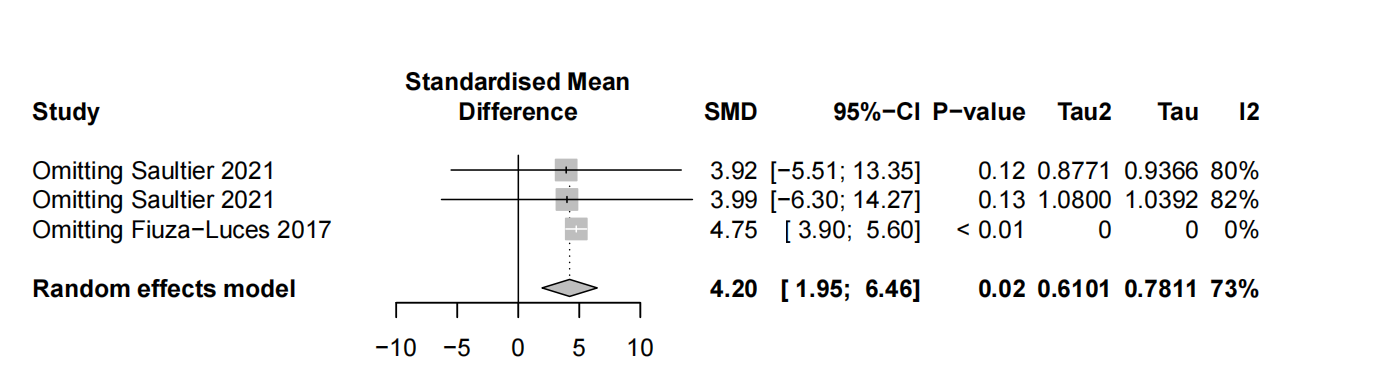


**6.7 Sensitivity analysis based on muscle strength**


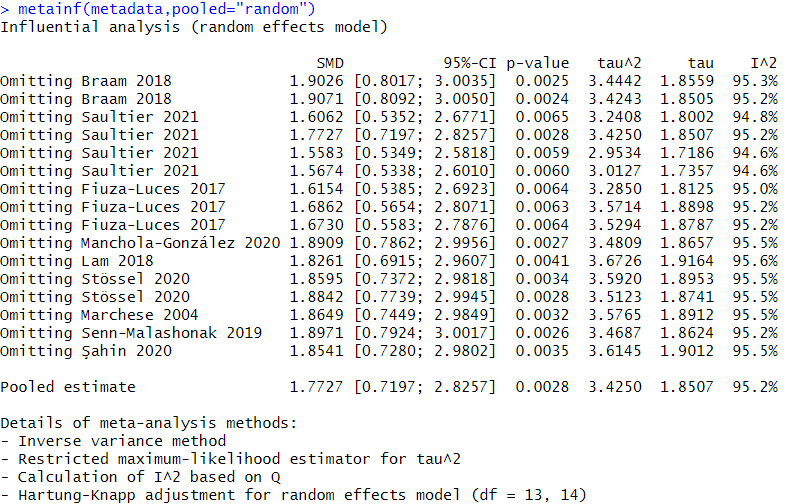


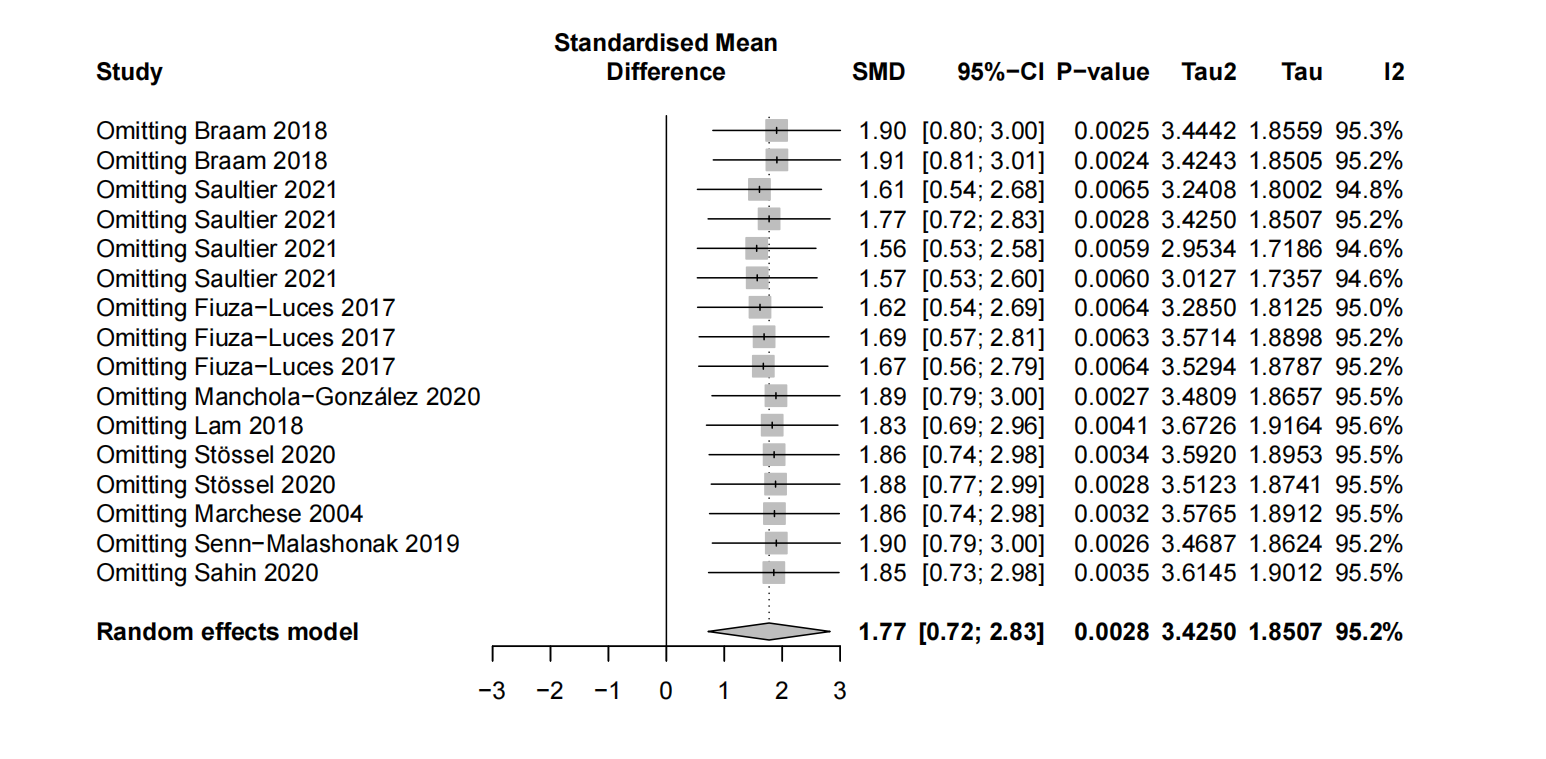


**6.8 Sensitivity analysis based on balance**


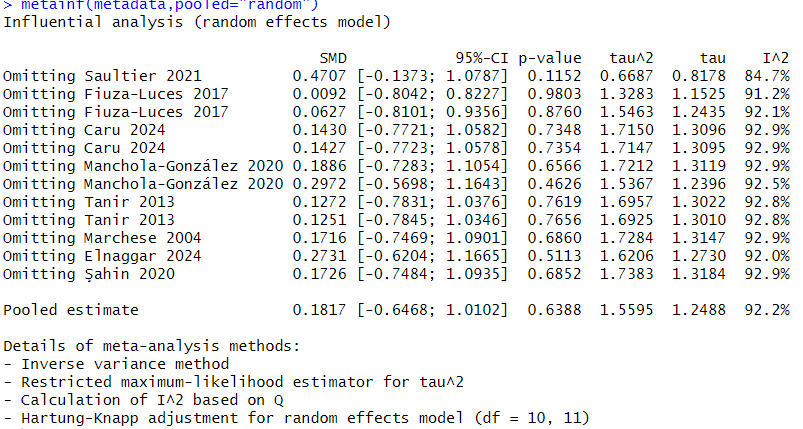


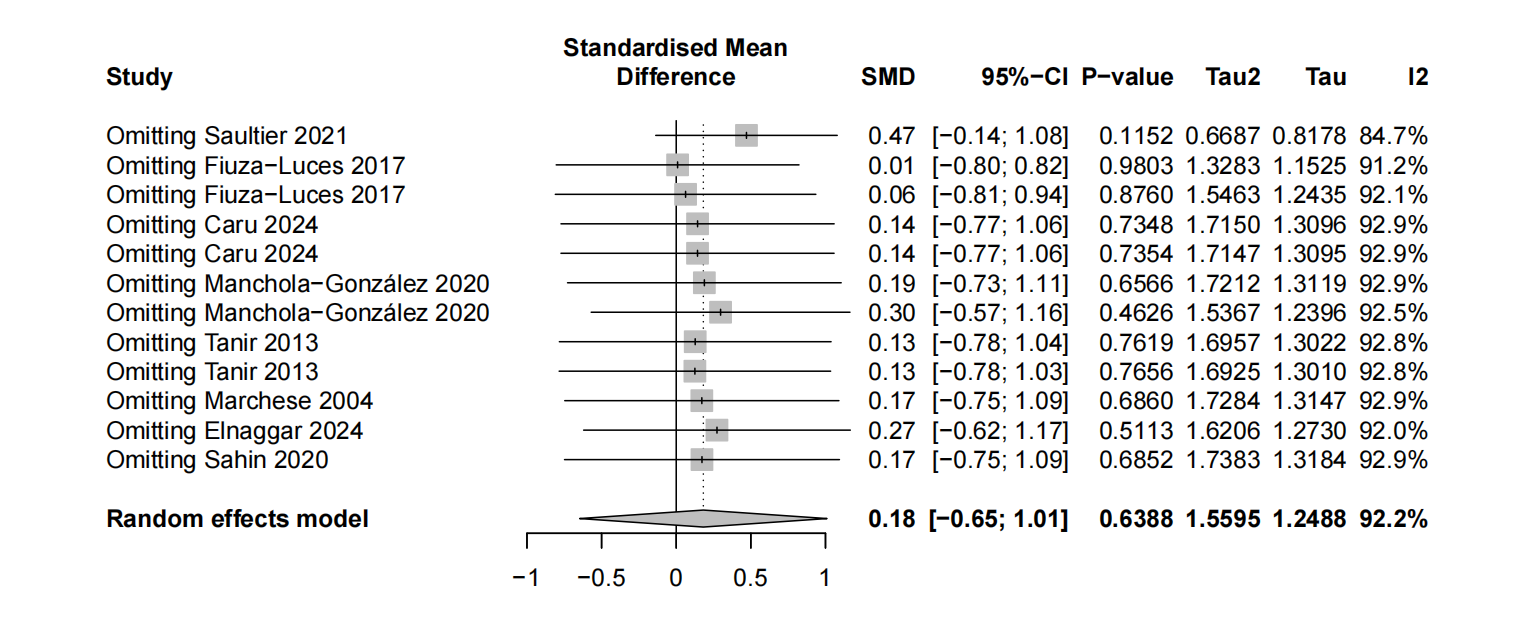


**6.9 Sensitivity analysis based on flexibility**


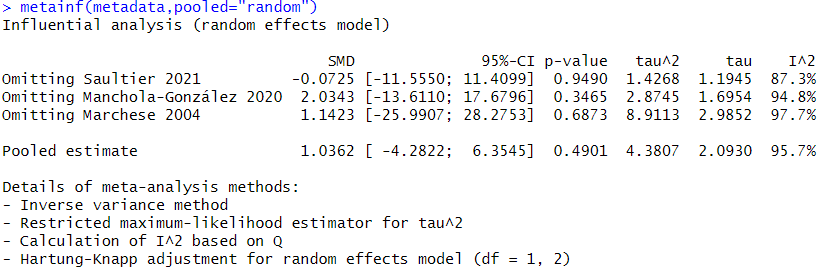


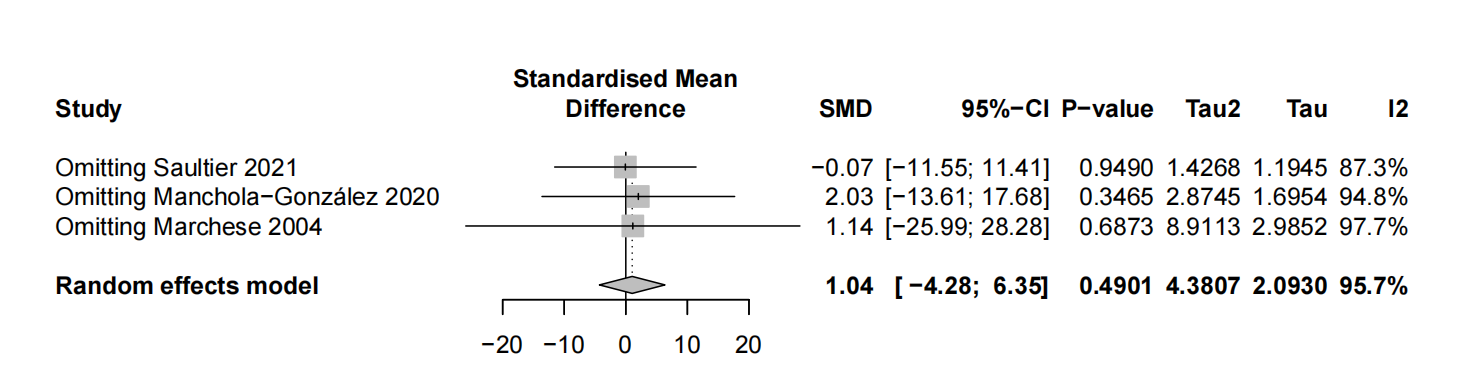


**6.10 Sensitivity analysis based on athletic performance**


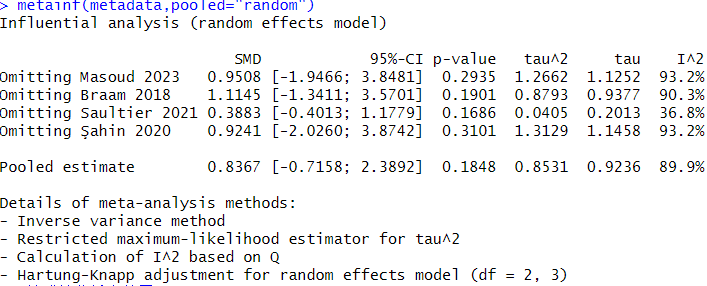


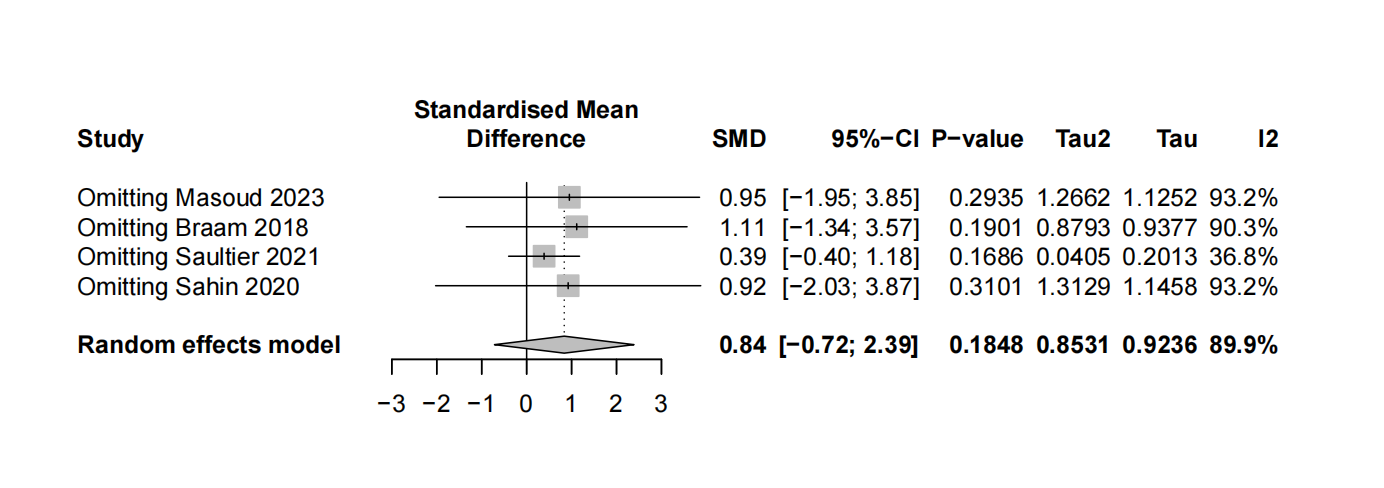


**6.11 Sensitivity analysis based on physical activity behaviour**


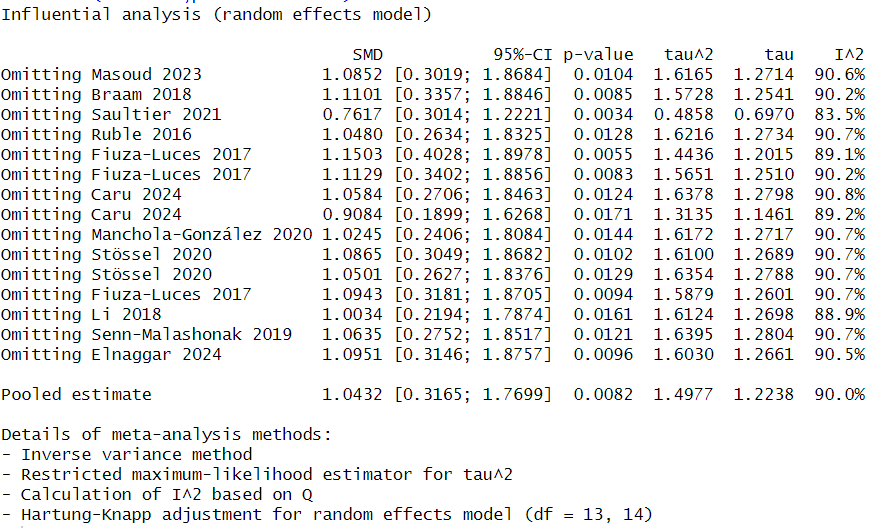


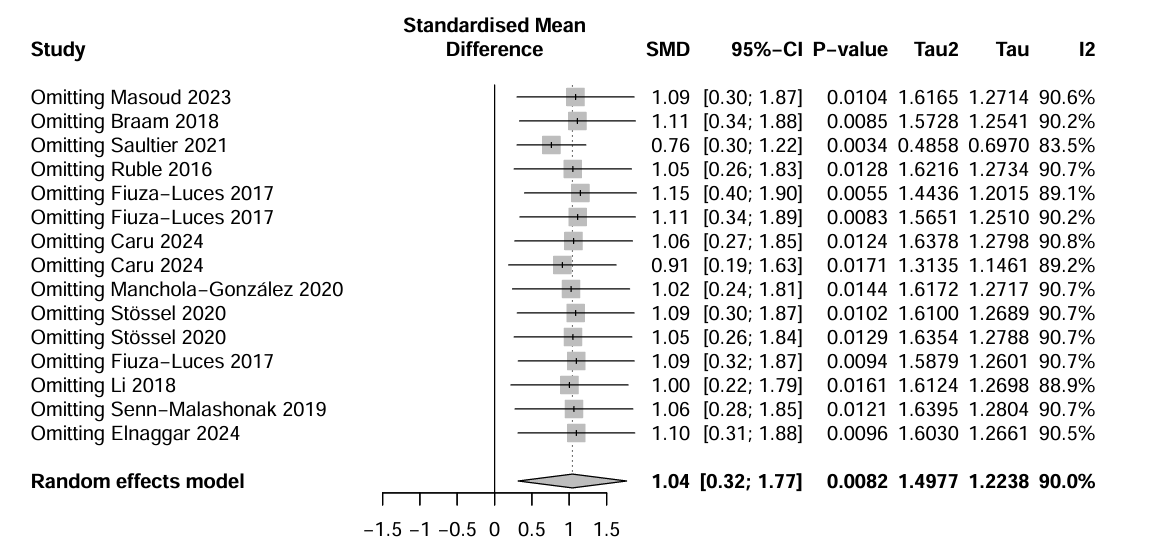
 **6.12 Sensitivity analysis based on physical activity level**


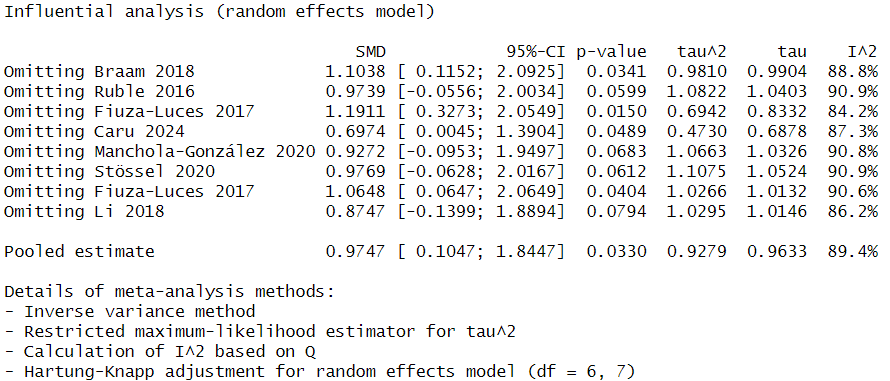


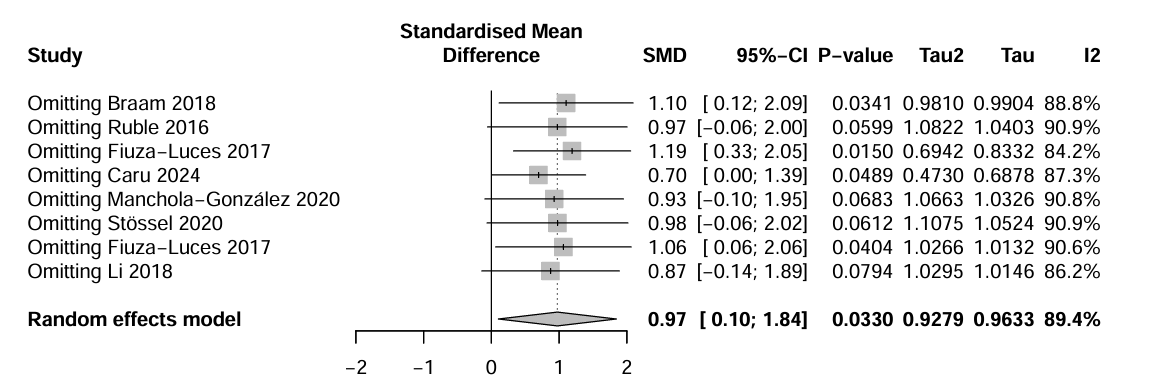


**6.13 Sensitivity analysis based on cardiorespiratory function**


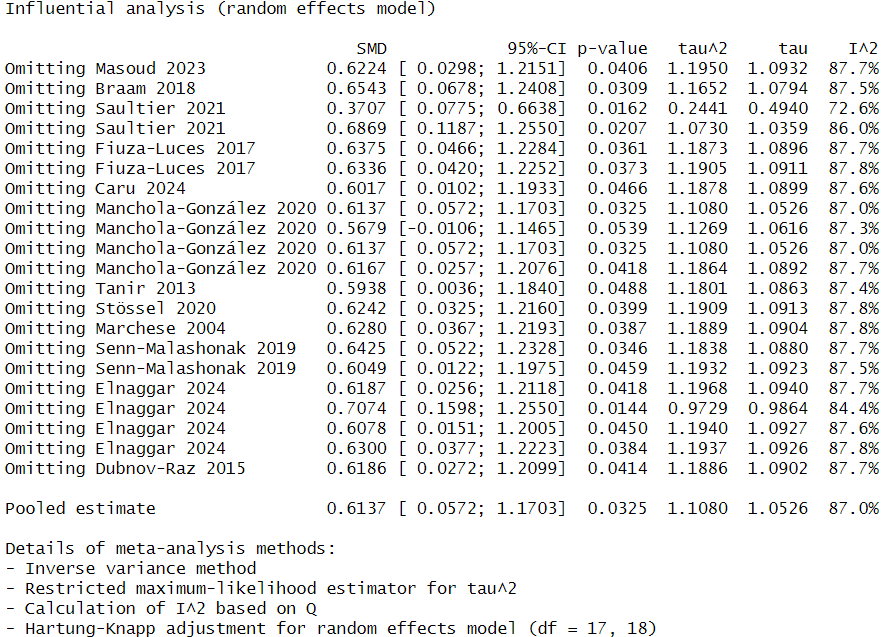


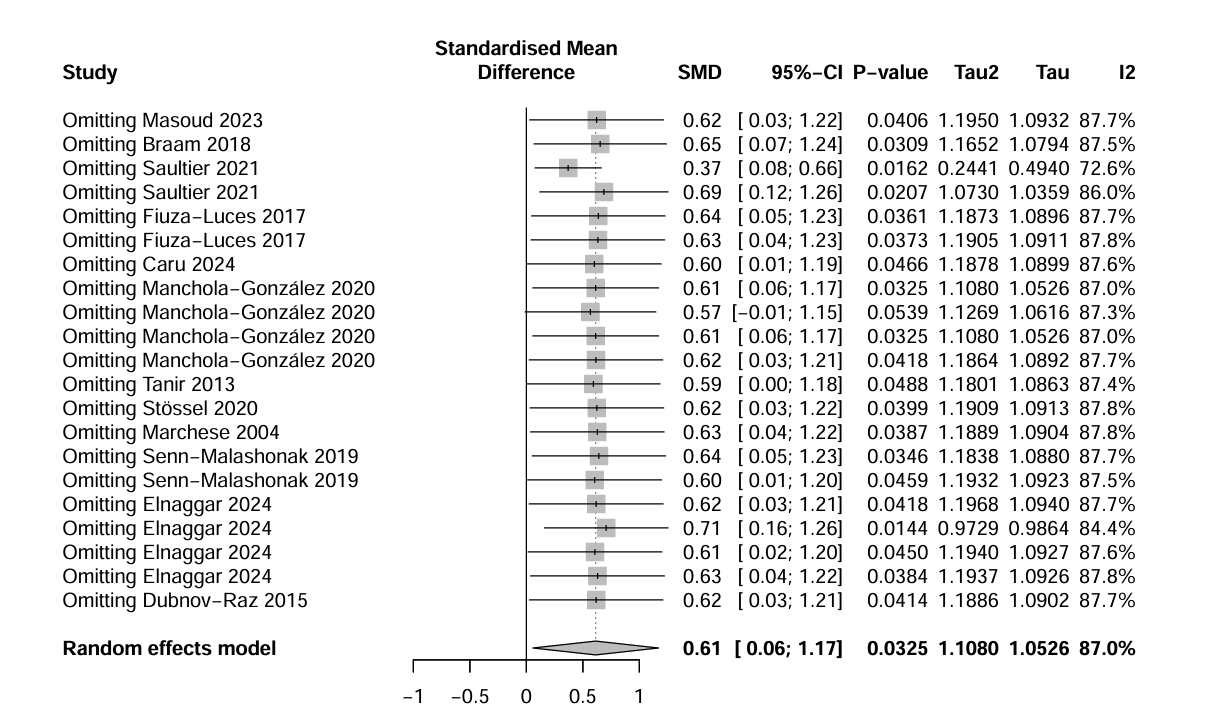


**6.14 Sensitivity analysis based on peak oxygen uptake**


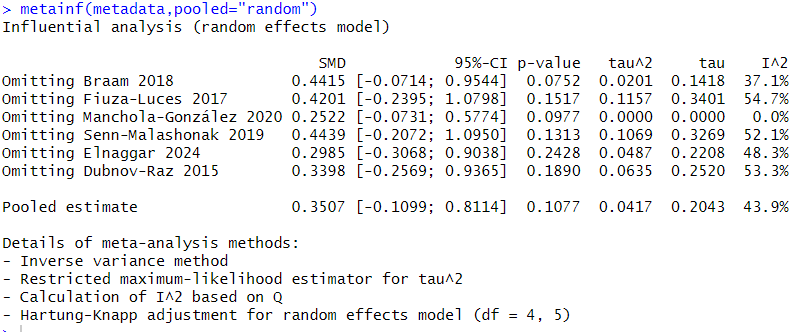


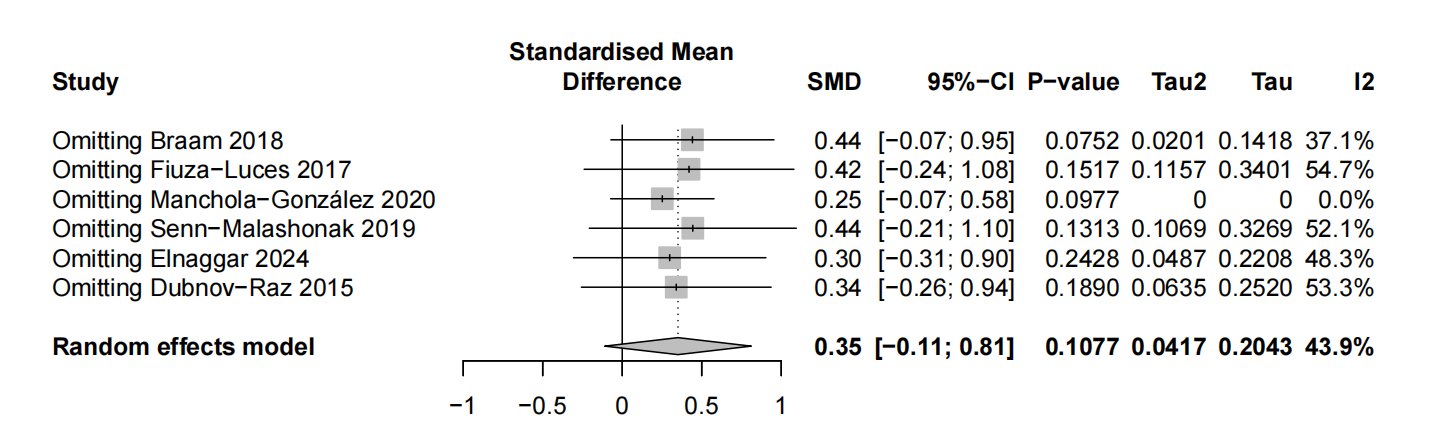


**6.15 Sensitivity analysis based on six-minute walk test**


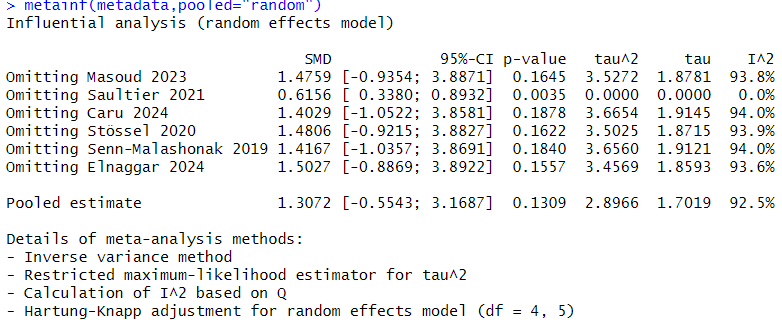


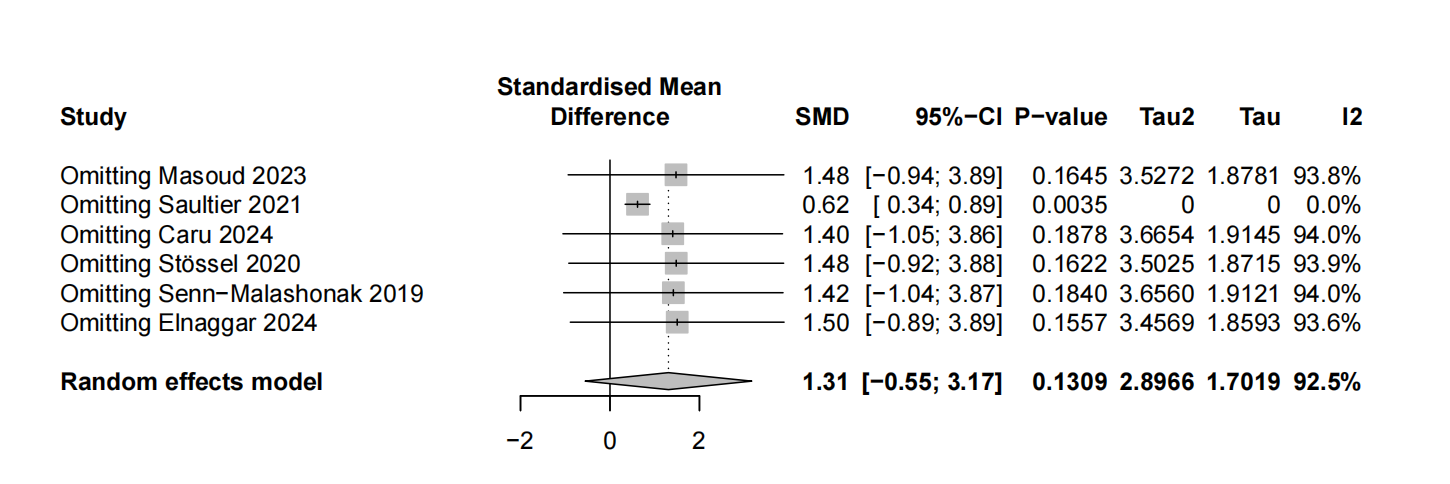


**6.16 Sensitivity analysis based on cognitive function**


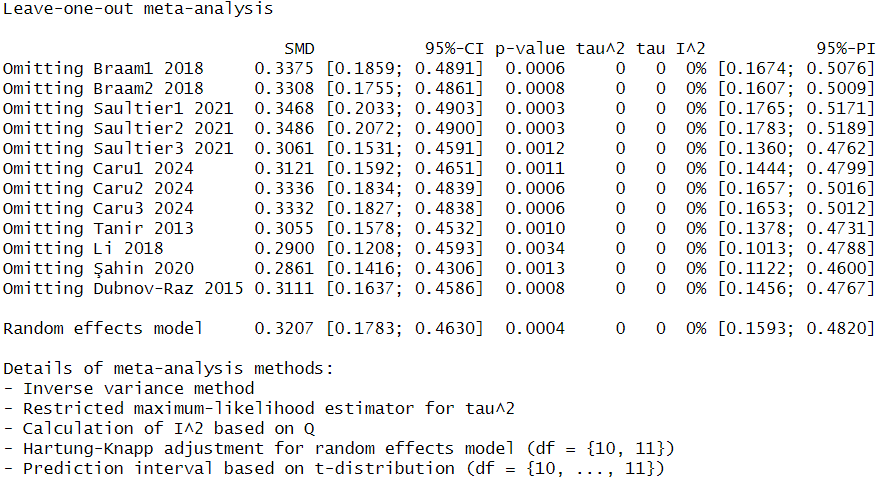


**
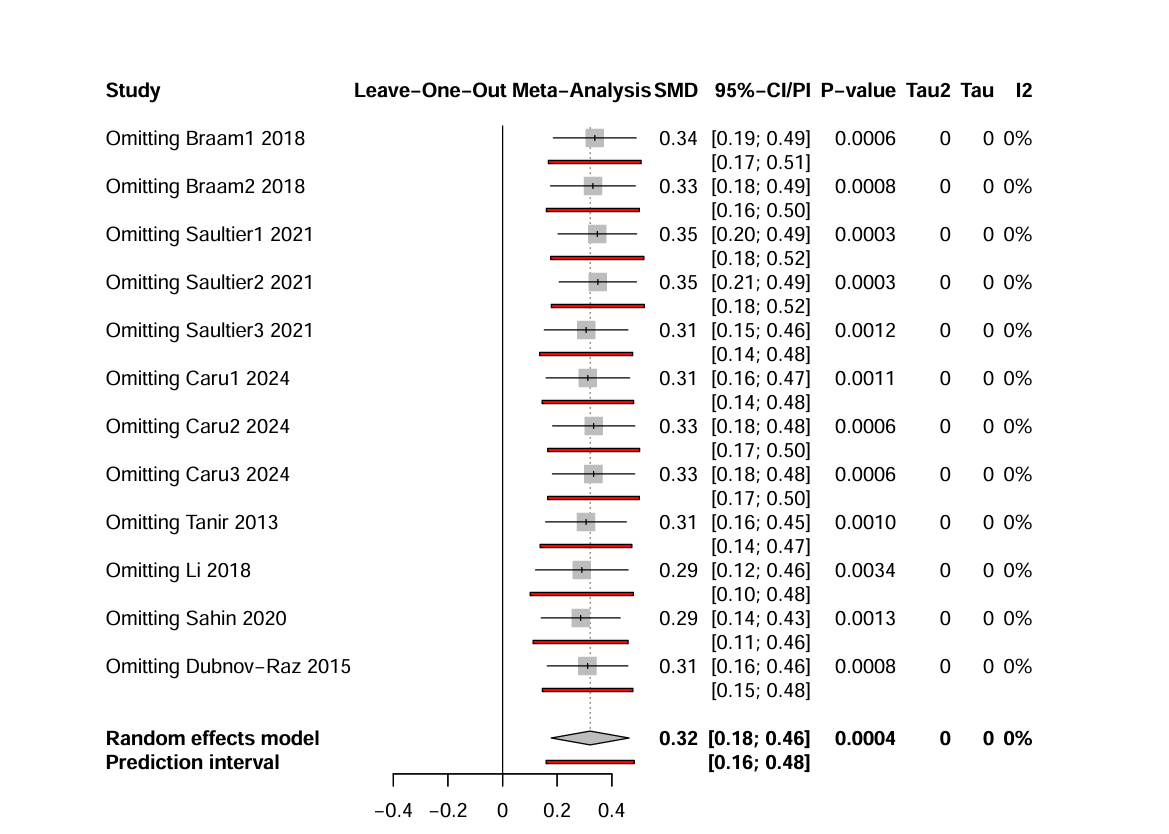
**

**6.17 Sensitivity analysis based on executive functions**


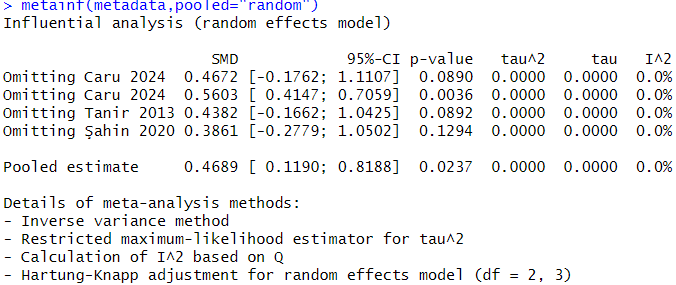


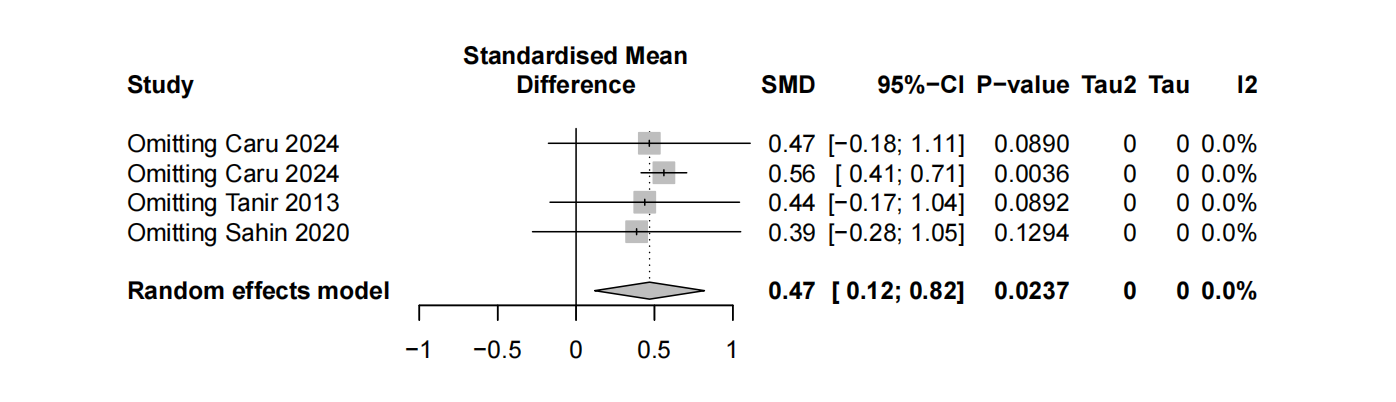


**6.18 Sensitivity analysis based on depressive symptoms**


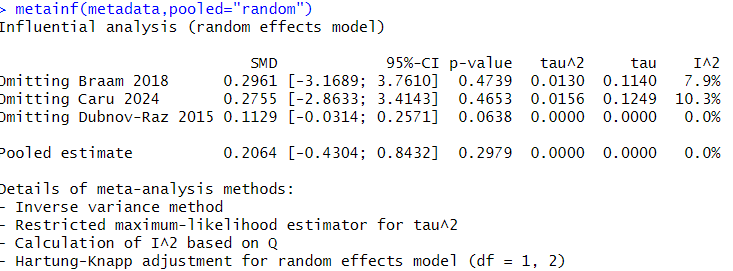


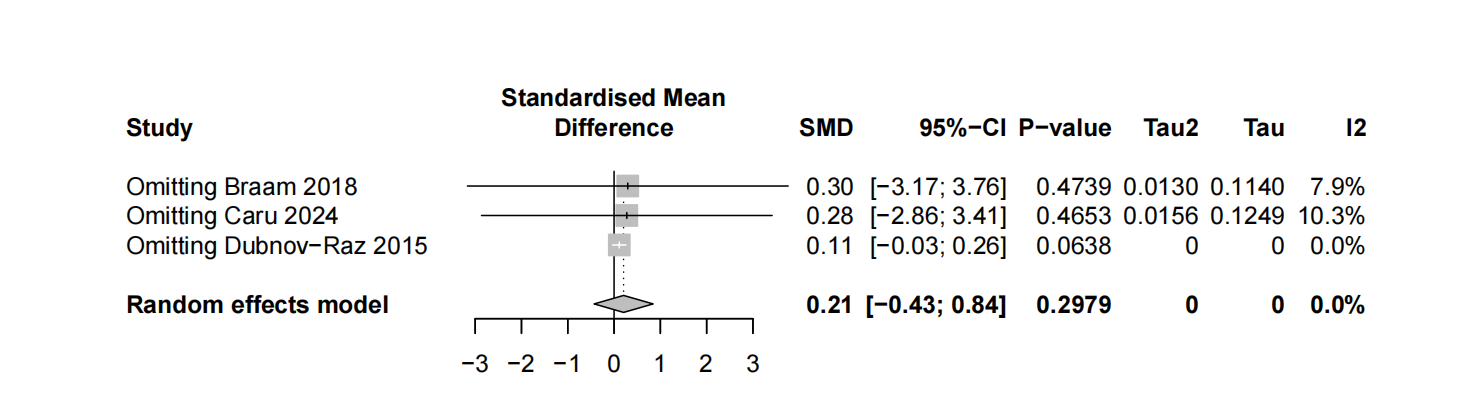


**6.19 Sensitivity analysis based on social function**


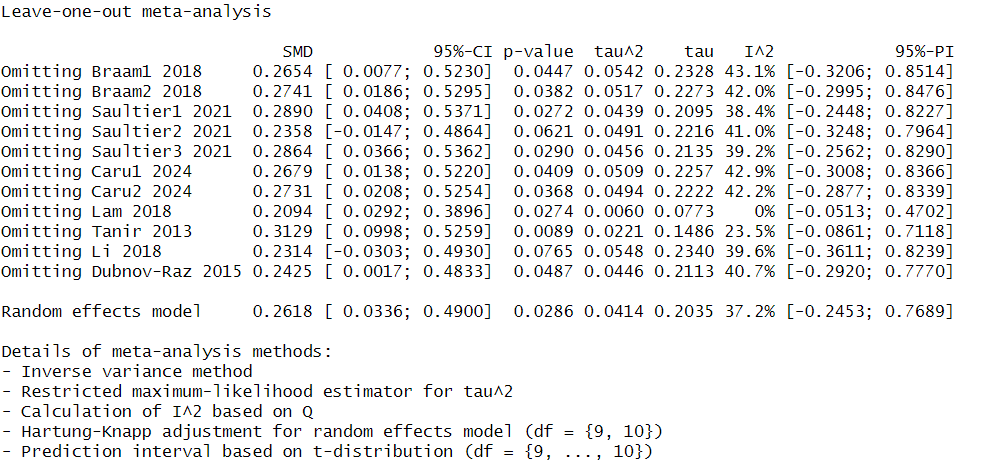


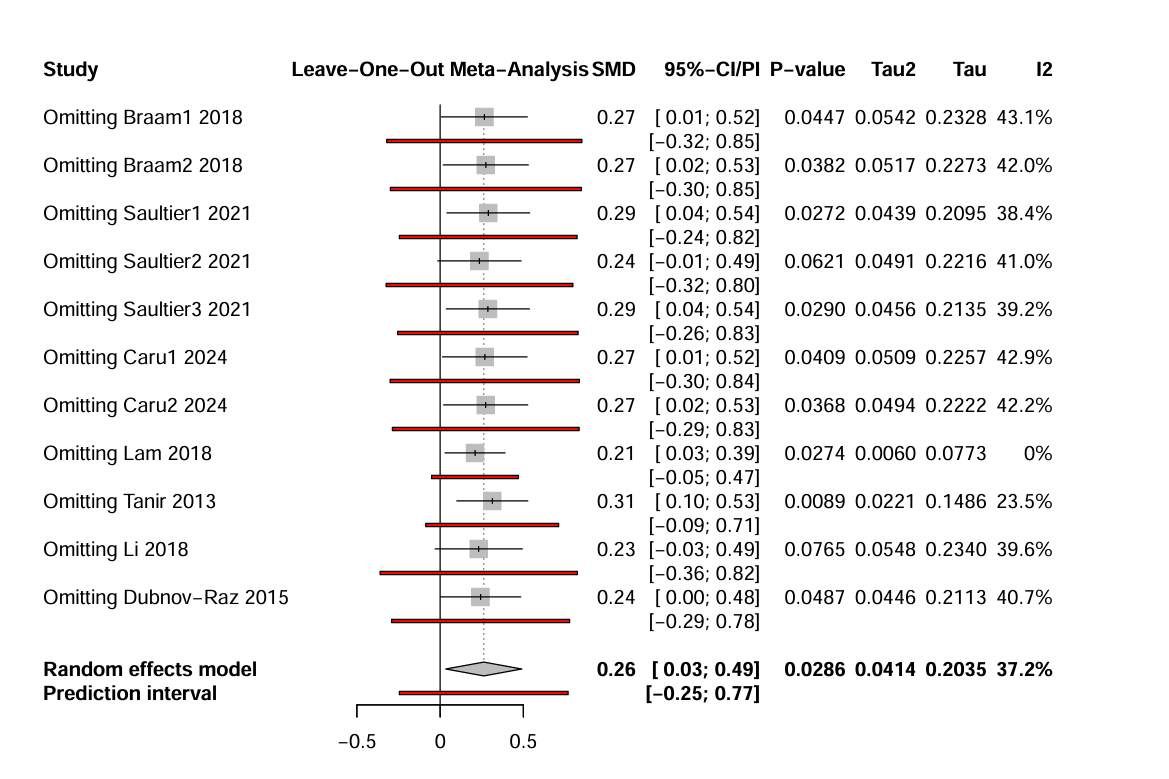


**6.20 Sensitivity analysis based on bone mineral density**


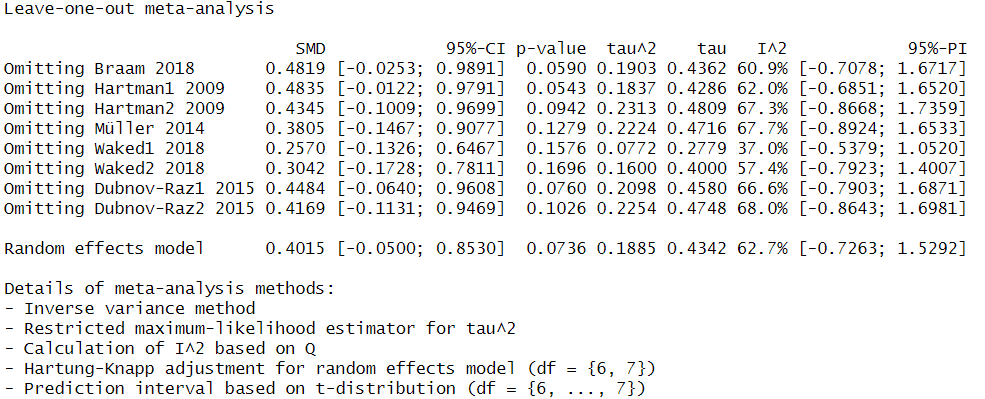


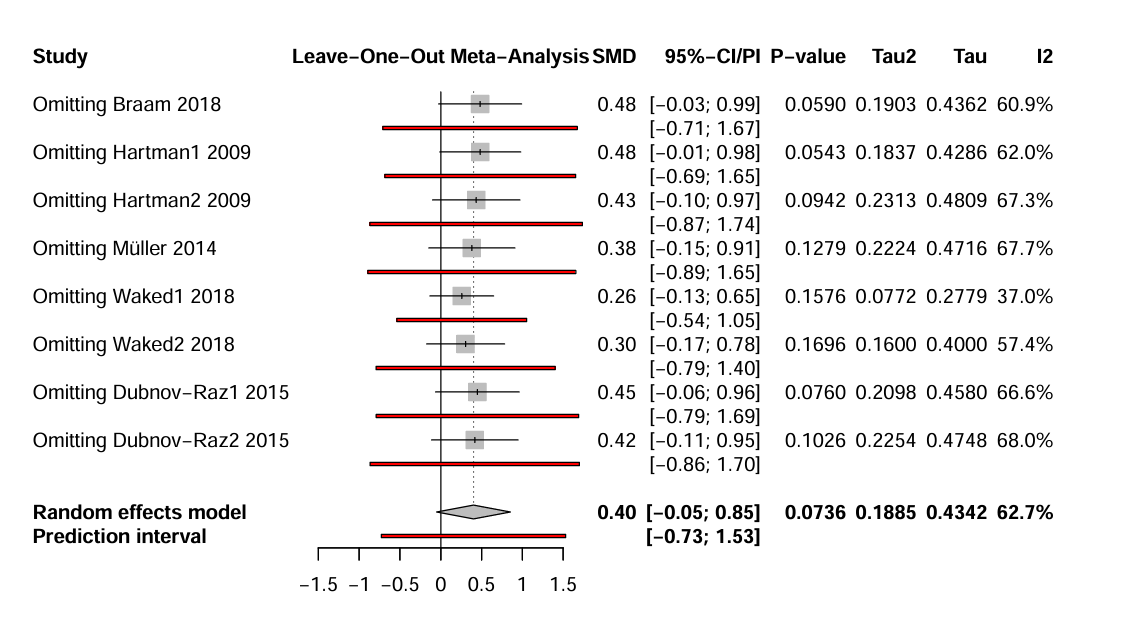


**6.21 Sensitivity analysis based on body mass index**


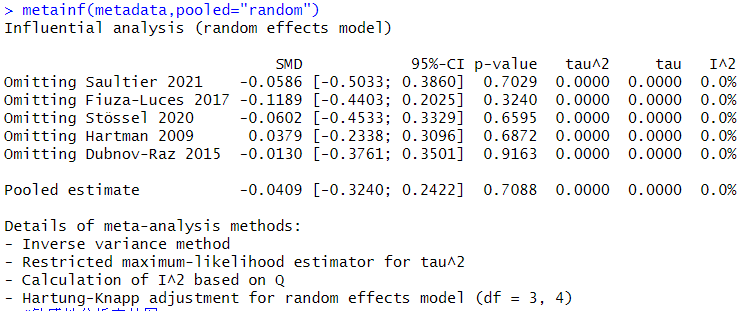


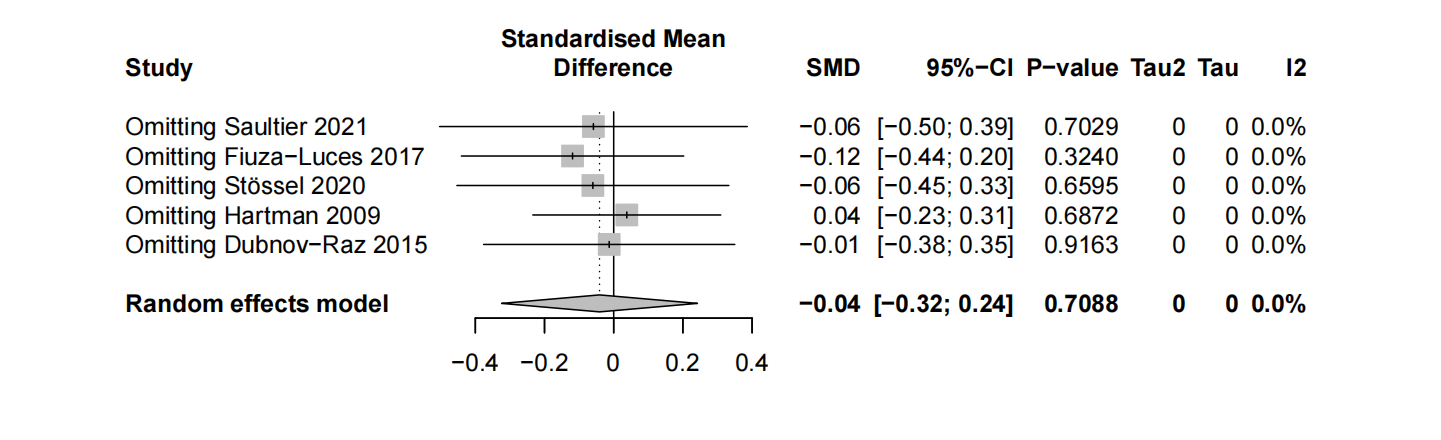


**6.22 Sensitivity analysis based on fat mass percentage**


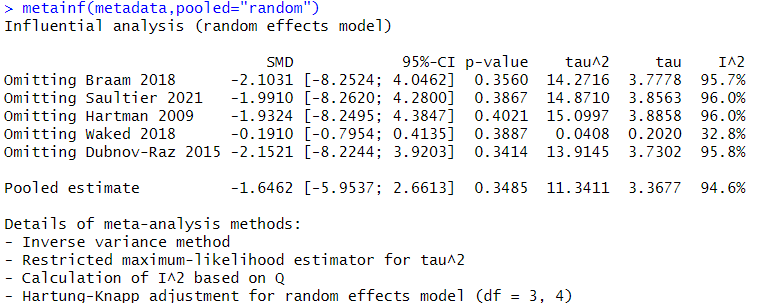


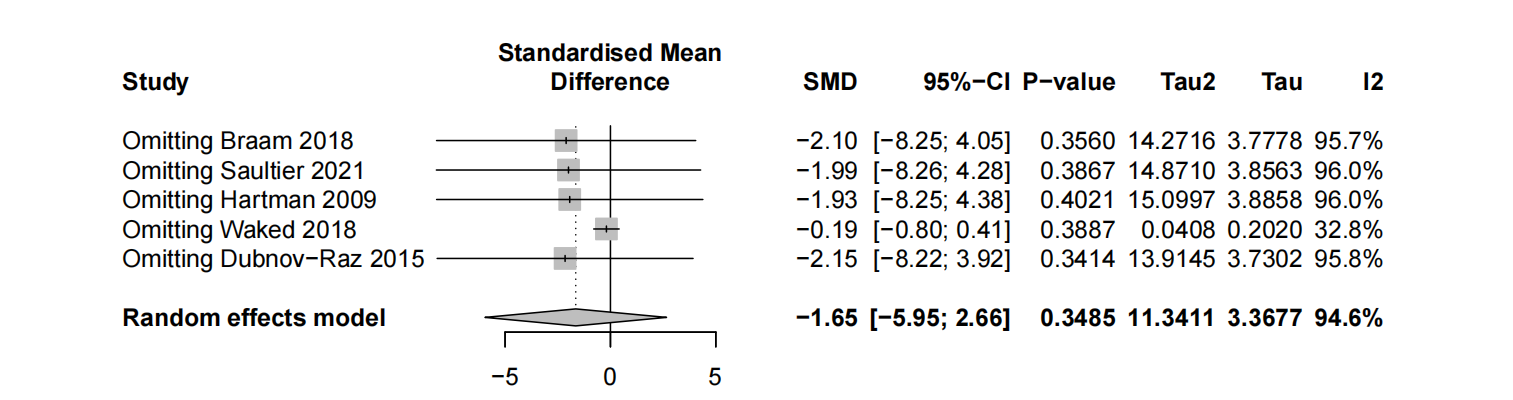


**6.23 Sensitivity analysis based on NK cell level**


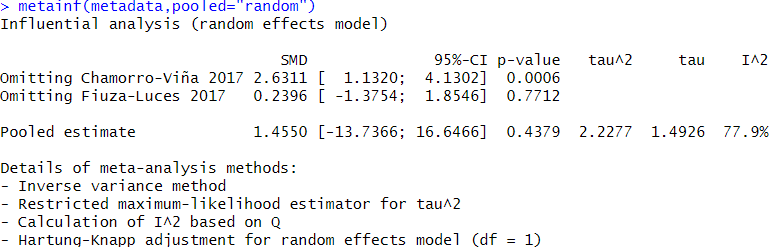


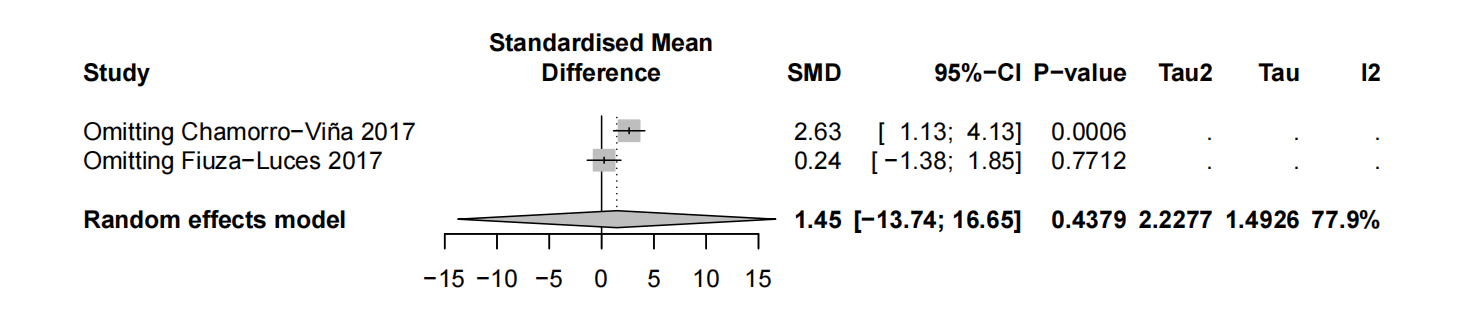


**6.24 Sensitivity analysis based on pro-inflammatory factor**


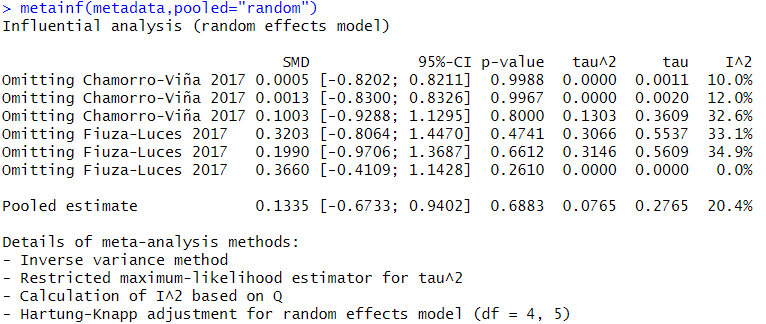


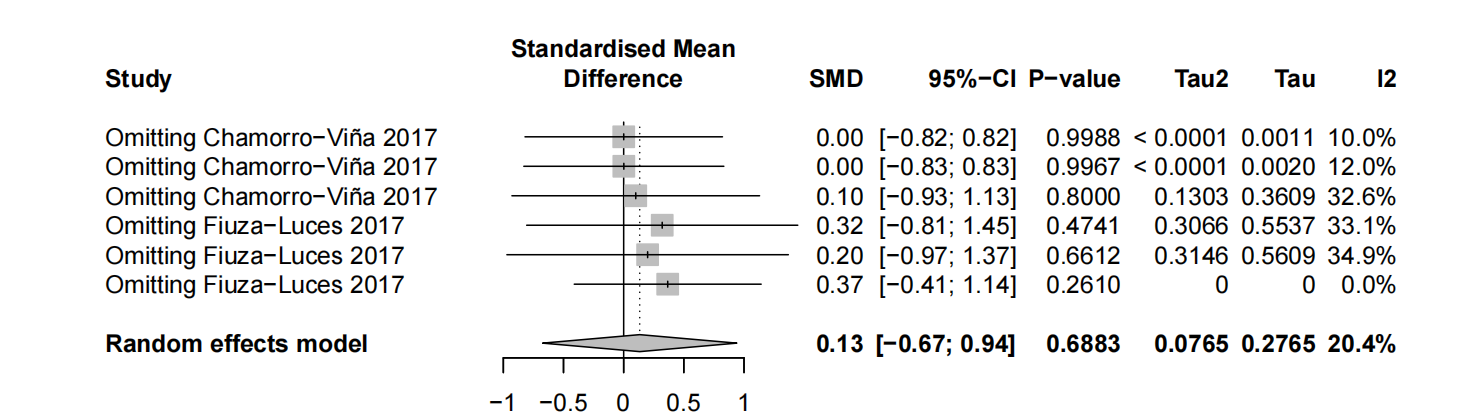


**6.25 Sensitivity analysis based on anti-inflammatory factor**


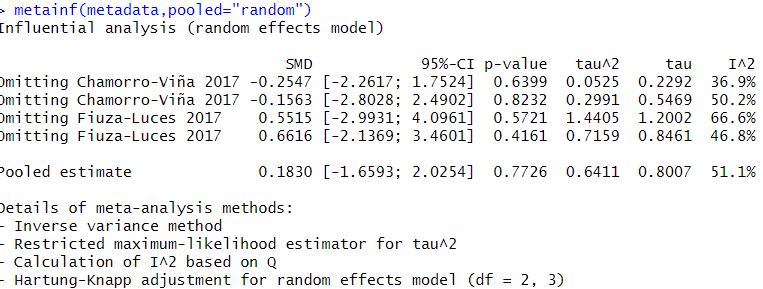


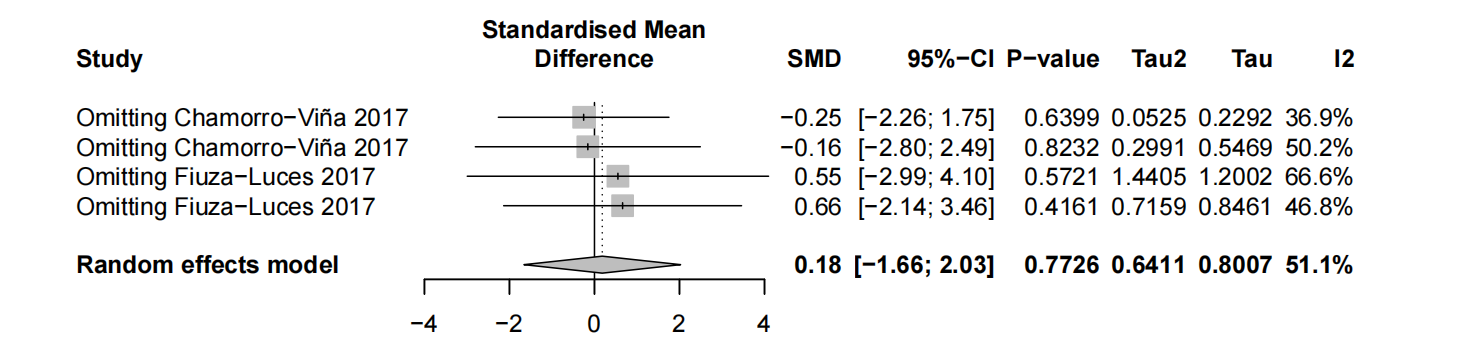


**7. Publication bias**

**7.1 Publication bias based on quality of life scale**

**7.2 Publication bias based on fatigue**

**7.3 Publication bias based on quality of life**

**7.4 Publication bias based on lower body muscle strength**

**7.5 Publication bias based on upper body muscle strength**

**7.6 Publication bias based on trunk muscle strength**

**7.7 Publication bias based on muscle strength**

**7.8 Publication bias based on balance**

**7.9 Publication bias based on flexibility**

**7.10 Publication bias based on athletic performance**

**7.11 Publication bias based on physical activity behaviour**

**7.12 Publication bias based on physical activity level**

**7.13 Publication bias based on cardiorespiratory function**

**7.14 Publication bias based on peak oxygen uptake**

**7.15 Publication bias based on six-minute walk test**

**7.16 Publication bias based on cognitive function**

**7.17 Publication bias based on executive functions**

**7.18 Publication bias based on depressive symptoms**

**7.19 Publication bias based on social function**

**7.20 Publication bias based on bone mineral density**

**7.21 Publication bias based on body mass index**

**7.22 Publication bias based on fat mass percentage**

**7.23 Publication bias based on NK cell level**

**Pro-inflammatory factor**

**7.24 Publication bias based on anti-inflammatory factor**

**8. Results of the trim and fill method**

**8.1 Trim and fill method based on muscle strength**

**8.2 Trim and fill method based on cardiorespiratory function**

**8.3 Trim and fill method based on pro-inflammatory factors**

**9.** **GRADE assessment**

The GRADE approach categorizes the certainty of evidence into four levels—high, moderate, low, and very low—and assesses the confidence that the estimated effect size approximates the true effect^1,2^. When applying GRADE, evidence derived from randomized interventions is initially rated as ‘high’ certainty^3^. Given that our evidence stems from randomized controlled trials evaluating physical activity interventions, we have accordingly assigned a ‘high’ certainty rating to these findings. Subsequent downgrading of evidence certainty was performed based on five domains: risk of bias in individual studies, inconsistency, indirectness, imprecision, and publication bias, as outlined in the GRADE methodology^1,3^. The risk of bias in individual studies was evaluated using the Cochrane Collaboration’s RoB 2 tool for randomized studies (**Supplementary File 4**)^4^. The assessments identified instances of ‘high risk’ or ‘some concerns,’ which may compromise the reliability of the findings—where ‘some concerns’ warrants a downgrade of one level, and ‘high risk’ justifies a two-level downgrade. Inconsistency was gauged through the I² heterogeneity statistic, with values exceeding 90% indicating substantial heterogeneity—and thus, a downgrade by one level. An I² threshold of 90% was chosen, considering that complex public health interventions generally demonstrate greater heterogeneity compared to clinical interventions^5^. Indirectness was attributed to instances where studies could not directly measure the outcomes of interest or when evidence was restricted to one or two specific settings, limiting the applicability of results to broader contexts; such cases warranted an additional downgrade. Imprecision was considered when the meta-analyses of Standardized Mean Differences encompassed zero or were based on a limited number of studies and participants, leading to downgrading by one level. To evaluate publication bias, funnel plots were visually inspected according to outcomes, complemented by Egger’s regression test—applied when at least 3 comparisons were available in the meta-analysis—to detect small-study effects (funnel plots and Egger test results in **Supplementary File 7**).

**GRADE rating approach by outcomes**

| Outcome | Risk of bias | Indirectness | Inconsistency | Imprecision | | Publication bias | Final GRADE |
| --- | --- | --- | --- | --- | --- | --- | --- |
|  | Risk of bias was in all studies | Downgrade by if studies not able to directly measure the intervention, or if evidence available for only 1-2 settings | Downgrade by 1 if I^2^> 90% | Downgrade by if 95% CI of  SMD includes 0 or based on few studies and/or participants | | Egger test  p<0.05 |  |
| Quality of life | Inadequate randomisation in some studies | n=21 comparisons across many countries/settings | 84.30% | 0.32 (-0.07, 0.71) | n=994 | 0.68 | ⊕⊕⊕O |
|  | -1 | 0 | 0 | 0 | | 0 | MODERATE |
| Quality of life scale | Inadequate randomisation in some studies | n=17 comparisons across many countries/settings | 87.20% | 0.36 (-0.12, 0.85) | n=850 | 0.64 | ⊕⊕⊕O |
|  | -1 | 0 | 0 | 0 | | 0 | MODERATE |
| Fatigue | Inadequate randomisation in some studies | n=8 comparisons across many countries/settings | 0.00% | 0.66 (0.43, 0.89) | n=506 | 0.36 | ⊕⊕⊕O |
|  | -1 | 0 | 0 | 0 | | 0 | MODERATE |
| Muscle strength | Inadequate randomisation in some studies | n=15 comparisons across many countries/settings | 95.20% | 1.77 (0.72, 2.83) | n=849 | 0.01 | ⊕OOO |
|  | -1 | 0 | -1 | 0 | | -1 | VERY LOW |
| Lower body muscle strength | Inadequate randomisation in some studies | n=5 comparisons across many countries/settings | 95.00% | 1.82 (-0.71, 4.36) | n=226 | 0.07 | ⊕⊕OO |
|  | -1 | 0 | -1 | 0 | | 0 | LOW |
| Upper body muscle strength | Inadequate randomisation in some studies | n=4 comparisons across many countries/settings | 0.00% | -0.01 (-0.37, 0.34) | n=238 | 0.62 | ⊕⊕⊕O |
|  | -1 | 0 | 0 | 0 | | 0 | MODERATE |
| Trunk muscle strength | Inadequate randomisation in some studies | n=3 comparisons across many countries/settings | 73.10% | 4.20 (1.95, 6.46) | n=173 | - | ⊕OOO |
|  | -1 | 0 | 0 | -1 | | -1 | VERY LOW |
| Balance | Inadequate randomisation in some studies | n=12 comparisons across many countries/settings | 92.20% | 0.18 (-0.65, 1.01) | n=537 | 0.74 | ⊕⊕OO |
|  | -1 | 0 | -1 | 0 | | 0 | LOW |
| Flexibility | Inadequate randomisation in some studies | n=3 comparisons across many countries/settings | 95.70% | 1.04 (-4.28, 6.35) | n=109 | 0.46 | ⊕OOO |
|  | -1 | 0 | -1 | -1 | | 0 | VERY LOW |
| Athletic performance | Inadequate randomisation in some studies | n=4 comparisons across many countries/settings | 89.90% | 0.84 (-0.72, 2.39) | n=197 | 0.76 | ⊕⊕OO |
|  | -1 | 0 | 0 | -1 | | 0 | LOW |
| Physical activity behaviour | Inadequate randomisation in some studies | n=15 comparisons across many countries/settings | 90.00% | 1.04 (0.32, 1.77) | n=767 | 0.59 | ⊕⊕⊕O |
|  | -1 | 0 | 0 | 0 | | 0 | MODERATE |
| Physical activity level | Inadequate randomisation in some studies | n=8 comparisons across many countries/settings | 89.40% | 0.97(0.10, 1.84) | n=415 | 0.81 | ⊕⊕⊕O |
|  | -1 | 0 | 0 | 0 | | 0 | MODERATE |
| Cardiorespiratory health | Inadequate randomisation in some studies | n=19 comparisons across many countries/settings | 87.00% | 0.61 (0.06, 1.17) | n=929 | 0.02 | ⊕⊕OO |
|  | -1 | 0 | 0 | 0 | | -1 | LOW |
| Peak oxygen uptake | Inadequate randomisation in some studies | n=5 comparisons across many countries/settings | 0.00% | 0.25 (-0.07, 0.58) | n=257 | 0.44 | ⊕⊕⊕O |
|  | -1 | 0 | 0 | 0 | | 0 | MODERATE |
| Six-minute walk test | Inadequate randomisation in some studies | n=5 comparisons across many countries/settings | 0.00% | 0.62 (0.34, 0.89) | n=241 | 0.86 | ⊕⊕⊕O |
|  | -1 | 0 | 0 | 0 | | 0 | MODERATE |
| Cognitive function | Inadequate randomisation in some studies | n=12 comparisons across many countries/settings | 0.00% | 0.32 (0.18, 0.46) | n=770 | 0.01 | ⊕⊕OO |
|  | -1 | 0 | 0 | 0 | | -1 | LOW |
| Executive function | Inadequate randomisation in some studies | n=4 comparisons across many countries/settings | 0.00% | 0.47 (0.12, 0.82) | n=213 | 0.54 | ⊕⊕⊕O |
|  | -1 | 0 | 0 | 0 | | 0 | MODERATE |
| Depressive symptoms | Inadequate randomisation in some studies | n=3 comparisons across many countries/settings | 0.00% | 0.21 (-0.43, 0.84) | n=120 | - | ⊕OOO |
|  | -1 | 0 | 0 | -1 | | -1 | VERY LOW |
| Social function | Inadequate randomisation in some studies | n=11 comparisons across many countries/settings | 3.20% | 0.23 (0.05, 0.42) | n=656 | 0.76 | ⊕⊕⊕O |
|  | -1 | 0 | 0 | 0 | | 0 | MODERATE |
| Bone mineral density | Inadequate randomisation in some studies | n=6 comparisons across many countries/settings | 0.00% | 0.09 (-0.15, 0.34) | n=209 | 0.80 | ⊕⊕⊕O |
|  | -1 | 0 | 0 | 0 | | 0 | MODERATE |
| Body mass index | Inadequate randomisation in some studies | n=5 comparisons across many countries/settings | 0.00% | -0.04 (-0.32, 0.23) | n=201 | 0.54 | ⊕⊕⊕O |
|  | -1 | 0 | 0 | 0 | | 0 | MODERATE |
| Fat mass percentage | Inadequate randomisation in some studies | n=5 comparisons across many countries/settings | 94.60% | -1.65 (-5.95, 2.66) | n=229 | 0.1 | ⊕⊕OO |
|  | -1 | 0 | -1 | 0 | | 0 | LOW |
| NK cell level | Inadequate randomisation in some studies | n=2 comparisons across many countries/settings | 77.90% | 1.46 (-13.74, 16.65) | n=21 | - | ⊕OOO |
|  | -1 | 0 | 0 | -1 | | -1 | VERY LOW |
| Pro-inflammatory factors | Inadequate randomisation in some studies | n=6 comparisons across many countries/settings | 20.40% | 0.13 (-0.67, 0.94) | n=63 | 0.04 | ⊕OOO |
|  | -1 | 0 | 0 | -1 | | -1 | VERY LOW |
| Anti-inflammatory factors | Inadequate randomisation in some studies | n=4 comparisons across many countries/settings | 51.10% | 0.18 (-1.66, 2.03) | n=36 | 0.29 | ⊕⊕OO |
|  | -1 | 0 | 0 | -1 | | 0 | LOW |

**10. Adverse events and acceptability analyses**

**10.1** **Acceptability analyses**

**10.2** **Adverse events**

Adverse event details. The information is as follows: in the trial by Tanir 2013 et al, 1 death in the experimental group; in the trial by Chamorro-Viña 2017 et al, 2 deaths in the control group; in the trial by Senn-Malashonak 2019 et al, 5 deaths of subjects; in the trial by Waked 2018 et al, 7 deaths in the control group and 5 deaths in the experimental group ; in the trial of Stössel 2020 et al, minor adverse events including leg pain, poor mental status, and lack of motivation to exercise; in the trial of Müller 2014 et al, subjects experienced relapses and serious complications.

**11. ACSM round table standards**

| Outcomes | ACSM Roundtable Criteria |
| --- | --- |
| Quality of life | √ |
| Quality of life scale | √ |
| Fatigue | √ |
| Muscle strength | √ |
| Lower body muscle strength | **×** |
| Upper body muscle strength | **×** |
| Trunk muscle strength | **×** |
| Balance | **×** |
| Flexibility | **×** |
| Athletic performance | **×** |
| Physical activity behaviour | √ |
| Physical activity level | √ |
| Cardiorespiratory health | **×** |
| Peak oxygen uptake | **×** |
| Six-minute walk test | **×** |
| Cognitive function | √ |
| Executive functions | **×** |
| Depressive symptoms | **×** |
| Social function | √ |
| Bone mineral density | √ |
| Body mass index | **×** |
| Fat mass Percentage | **×** |
| NK cell level | **×** |
| Pro-inflammatory factor | **×** |
| Anti-inflammatory factor | **×** |

**12. Regression analysis**

**12.1 Region regression analysis**

**12.1.1 Regression analysis based on region for quality of life scale**

**12.1.2 Regression analysis based on region for fatigue**

**12.1.3 Regression analysis based on region for quality of life**

**12.1.4 Regression analysis based on region for lower body muscle strength**

**12.1.5 Regression analysis based on region for trunk muscle strength**

**12.1.6 Regression analysis based on region for muscle strength**

**12.1.7 Regression analysis based on region for six-minute walk test**

**12.1.8 Regression analysis based on region for balance**

**12.1.9 Regression analysis based on region for flexibility**

**12.1.10 Regression analysis based on region for athletic performance**

**12.1.11 Regression analysis based on region for physical activity level**

**12.1.12 Regression analysis based on region for physical activity behaviour**

**12.1.13 Regression analysis based on region for peak oxygen uptake**

**12.1.14 Regression analysis based on region for cardiorespiratory function**

**12.1.15 Regression analysis based on region for cognitive function**

**12.1.16 Regression analysis based on region for bone mineral density**

**12.1.17 Regression analysis based on region for body mass index**

**12.1.18 Regression analysis based on region for fat mass percentage**

**12.1.19 Regression analysis based on region for depressive symptoms**

**12.1.20 Regression analysis based on region for social function**

**12.1.21 Regression analysis based on region for executive function**

**12.1.22 Regression analysis based on region for pro-inflammatory factors**

**12.1.23 Regression analysis based on region for anti-inflammatory factors**

**12.2 Age regression analysis**

**12.2.1 Regression analysis based on age for quality of life scale**

**12.2.2 Regression analysis based on age for fatigue**

**12.2.3 Regression analysis based on age for quality of life**

**12.2.4 Regression analysis based on age for lower body muscle strength**

**12.2.5 Regression analysis based on age for upper body muscle strength**

**12.2.6 Regression analysis based on age for trunk muscle strength**

**12.2.7 Regression analysis based on age for muscle strength**

**12.2.8 Regression analysis based on age for six-minute walk test**

**12.2.9 Regression analysis based on age for balance**

**12.2.10 Regression analysis based on age for flexibility**

**12.2.11 Regression analysis based on age for athletic performance**

**12.2.12 Regression analysis based on age for physical activity level**

**12.2.13 Regression analysis based on age for physical activity behaviour**

**12.2.14 Regression analysis based on age for peak oxygen uptake**

**12.2.15 Regression analysis based on age for cardiorespiratory function**

**12.2.16 Regression analysis based on age for bone mineral density**

**12.2.17 Regression analysis based on age for body mass index**

**12.2.18 Regression analysis based on age for fat mass percentage**

**12.2.19 Regression analysis based on age for depressive symptoms**

**12.2.20 Regression analysis based on age for social function**

**12.2.21 Regression analysis based on age for executive function**

**12.2.22 Regression analysis based on age for pro-inflammatory factors**

**12.2.23 Regression analysis based on age for anti-inflammatory factors**

**12.2.24 Regression analysis based on age for cognitive function**

**12.3 Medicine regression analysis**

**12.3.1 Regression analysis based on medicine for quality of life scale**

**12.3.2 Regression analysis based on medicine for fatigue**

**12.3.3 Regression analysis based on medicine for quality of life**

**12.3.4 Regression analysis based on medicine for lower body muscle strength**

**12.3.5 Regression analysis based on medicine for trunk muscle strength**

**12.3.6 Regression analysis based on medicine for muscle strength**

**12.3.7 Regression analysis based on medicine for six-minute walk test**

**12.3.8 Regression analysis based on medicine for balance**

**12.3.9 Regression analysis based on medicine for flexibility**

**12.3.10 Regression analysis based on medicine for athletic performance**

**12.3.11 Regression analysis based on medicine for physical activity level**

**12.3.12 Regression analysis based on medicine for physical activity behaviour**

**12.3.13 Regression analysis based on medicine for peak oxygen uptake**

**12.3.14 Regression analysis based on medicine for cardiorespiratory function**

**12.3.15 Regression analysis based on medicine for bone mineral density**

**12.3.16 Regression analysis based on medicine for body mass index**

**12.3.17 Regression analysis based on medicine for fat mass percentage**

**12.3.18 Regression analysis based on medicine for social function**

**12.3.19 Regression analysis based on medicine for executive function**

**12.3.20 Regression analysis based on medicine for pro-inflammatory factors**

**12.3.21 Regression analysis based on medicine for anti-inflammatory factors**

**12.3.22 Regression analysis based on medicine for cognitive function**

**12.4 Disease types regression analysis**

**12.4.1 Regression analysis based on disease types for quality of life scale**

**12.4.2 Regression analysis based on disease types for fatigue**

**12.4.3 Regression analysis based on disease types for quality of life**

**12.4.4 Regression analysis based on disease types for lower body muscle strength**

**12.4.5 Regression analysis based on disease types for upper body muscle strength**

**12.4.6 Regression analysis based on disease types for trunk muscle strength**

**12.4.7 Regression analysis based on disease types for muscle strength**

**12.4.8 Regression analysis based on disease types for six-minute walk test**

**12.4.9 Regression analysis based on disease types for balance**

**12.4.10 Regression analysis based on disease types for flexibility**

**12.4.11 Regression analysis based on disease types for athletic performance**

**12.4.12 Regression analysis based on disease types for physical activity level**

**12.4.13 Regression analysis based on disease types for physical activity behaviour**

**12.4.14 Regression analysis based on disease types for peak oxygen uptake**

**12.4.15 Regression analysis based on disease types for cardiorespiratory function**

**12.4.16 Regression analysis based on disease types for bone mineral density**

**12.4.17 Regression analysis based on disease types for body mass index**

**12.4.18 Regression analysis based on disease types for fat mass percentage**

**12.4.19 Regression analysis based on disease types for depressive symptoms**

**12.4.20 Regression analysis based on disease types for social function**

**12.4.21 Regression analysis based on disease types for executive function**

**12.4.22 Regression analysis based on disease types for pro-inflammatory factors**

**12.4.23 Regression analysis based on disease types for anti-inflammatory factors**

**12.4.24 Regression analysis based on disease types for cognitive function**

**12.5 Treatment stage regression analysis**

**12.5.1 Regression analysis based on treatment stage for quality of life scale**

**12.5.2 Regression analysis based on treatment stage for fatigue**

**12.5.3 Regression analysis based on treatment stage for quality of life**

**12.5.4 Regression analysis based on treatment stage for lower body muscle strength**

**12.5.5 Regression analysis based on treatment stage for upper body muscle strength**

**12.5.6 Regression analysis based on treatment stage for trunk muscle strength**

**12.5.7 Regression analysis based on treatment stage for muscle strength**

**12.5.8 Regression analysis based on treatment stage for six-minute walk test**

**12.5.9 Regression analysis based on treatment stage for balance**

**12.5.10 Regression analysis based on treatment stage for flexibility**

**12.5.11 Regression analysis based on treatment stage for athletic performance**

**12.5.12 Regression analysis based on treatment stage for physical activity level**

**12.5.13 Regression analysis based on treatment stage for physical activity behaviour**

**12.5.14 Regression analysis based on treatment stage for peak oxygen uptake**

**12.5.15 Regression analysis based on treatment stage for cardiorespiratory function**

**12.5.16 Regression analysis based on treatment stage for bone mineral density**

**12.5.17 Regression analysis based on treatment stage for body mass index**

**12.5.18 Regression analysis based on treatment stage for fat mass percentage**

**12.5.19 Regression analysis based on treatment stage for depressive symptoms**

**12.5.20 Regression analysis based on treatment stage for social function**

**12.5.21 Regression analysis based on treatment stage for executive function**

**12.5.22 Regression analysis based on treatment stage for pro-inflammatory factors**

**12.5.23 Regression analysis based on treatment stage for anti-inflammatory factors**

**12.5.24 Regression analysis based on treatment stage for cognitive function**

**12.6 Sessions regression analysis**

**12.6.1 Regression analysis based on sessions for quality of life scale**

**12.6.2 Regression analysis based on sessions for fatigue**

**12.6.3 Regression analysis based on sessions for quality of life**

**12.6.4 Regression analysis based on sessions for lower body muscle strength**

**12.6.5 Regression analysis based on sessions for upper body muscle strength**

**12.6.6 Regression analysis based on sessions for muscle strength**

**12.6.7 Regression analysis based on sessions for six-minute walk test**

**12.6.8 Regression analysis based on sessions for balance**

**12.6.9 Regression analysis based on sessions for athletic performance**

**12.6.10 Regression analysis based on sessions for physical activity level**

**12.6.11 Regression analysis based on sessions for physical activity behaviour**

**12.6.12 Regression analysis based on sessions for peak oxygen uptake**

**12.6.13 Regression analysis based on sessions for cardiorespiratory function**

**12.6.14 Regression analysis based on sessions for bone mineral density**

**12.6.15 Regression analysis based on sessions for body mass index**

**12.6.16 Regression analysis based on sessions for fat mass percentage**

**12.6.17 Regression analysis based on sessions for depressive symptoms**

**12.6.18 Regression analysis based on sessions for social function**

**12.6.19 Regression analysis based on sessions for executive function**

**12.6.20 Regression analysis based on sessions for pro-inflammatory factors**

**12.6.21 Regression analysis based on sessions for cognitive function**

**12.7 Length regression analysis**

**12.7.1 Regression analysis based on length for quality of life scale**

**12.7.2 Regression analysis based on length for fatigue**

**12.7.3 Regression analysis based on length for quality of life**

**12.7.4 Regression analysis based on length for lower body muscle strength**

**12.7.5 Regression analysis based on length for upper body muscle strength**

**12.7.6 Regression analysis based on length for trunk muscle strength**

**12.7.7 Regression analysis based on length for muscle strength**

**12.7.8 Regression analysis based on length for six-minute walk test**

**12.7.9 Regression analysis based on length for balance**

**12.7.10 Regression analysis based on length for flexibility**

**12.7.11 Regression analysis based on length for athletic performance**

**12.7.12 Regression analysis based on length for physical activity level**

**12.7.13 Regression analysis based on length for physical activity behaviour**

**12.7.14 Regression analysis based on length for peak oxygen uptake**

**12.7.15 Regression analysis based on length for cardiorespiratory function**

**12.7.16 Regression analysis based on length for bone mineral density**

**12.7.17 Regression analysis based on length for body mass index**

**12.7.18 Regression analysis based on length for fat mass percentage**

**12.7.19 Regression analysis based on length for depressive symptoms**

**12.7.20 Regression analysis based on length for social function**

**12.7.21 Regression analysis based on length for executive function**

**12.7.22 Regression analysis based on length for pro-inflammatory factors**

**12.7.23 Regression analysis based on length for anti-inflammatory factors**

**12.7.24 Regression analysis based on length for cognitive function**

**12.8 Sessions duration regression analysis**

**12.8.1 Regression analysis based on sessions duration for quality of life scale**

**12.8.2 Regression analysis based on sessions duration for fatigue**

**12.8.3 Regression analysis based on sessions duration for quality of life**

**12.8.3 Regression analysis based on sessions duration for lower body muscle strength**

**12.8.4 Regression analysis based on sessions duration for upper body muscle strength**

**12.8.5 Regression analysis based on sessions duration for trunk muscle strength**

**12.8.6 Regression analysis based on sessions duration for muscle strength**

**12.8.7 Regression analysis based on sessions duration for six-minute walk test**

**12.8.8 Regression analysis based on sessions duration for balance**

**12.8.9 Regression analysis based on sessions duration for athletic performance**

**12.8.10 Regression analysis based on sessions duration for physical activity level**

**12.8.11 Regression analysis based on sessions duration for physical activity behaviour**

**12.8.12 Regression analysis based on sessions duration for peak oxygen uptake**

**12.8.13 Regression analysis based on sessions duration for cardiorespiratory function**

**12.8.14 Regression analysis based on sessions duration for body mass index**

**12.8.15 Regression analysis based on sessions duration for fat mass percentage**

**12.8.16 Regression analysis based on sessions duration for depressive symptoms**

**12.8.17 Regression analysis based on sessions duration for social function**

**12.8.18 Regression analysis based on sessions duration for executive function**

**12.8.19 Regression analysis based on sessions duration for pro-inflammatory factors**

**12.8.20 Regression analysis based on sessions duration for anti-inflammatory factors**

**12.8.21 Regression analysis based on sessions duration for cognitive function**

**12.9 Exercise intensity regression analysis**

**12.9.1 Regression analysis based on exercise intensity for quality of life scale**

**12.9.2 Regression analysis based on exercise intensity for fatigue**

**12.9.3 Regression analysis based on exercise intensity for quality of life**

**12.9.4 Regression analysis based on exercise intensity for lower body muscle strength**

**12.9.5 Regression analysis based on exercise intensity for upper body muscle strength**

**12.9.6 Regression analysis based on exercise intensity for trunk muscle strength**

**12.9.7 Regression analysis based on exercise intensity for muscle strength**

**12.9.8 Regression analysis based on exercise intensity for six-minute walk test**

**12.9.9 Regression analysis based on exercise intensity for balance**

**12.9.10 Regression analysis based on exercise intensity for flexibility**

**12.9.11 Regression analysis based on exercise intensity for athletic performance**

**12.9.12 Regression analysis based on exercise intensity for physical activity level**

**12.9.13 Regression analysis based on sessions duration for physical activity behaviour**

**12.9.14 Regression analysis based on exercise intensity for peak oxygen uptake**

**12.9.15 Regression analysis based on exercise intensity for cardiorespiratory function**

**12.9.16 Regression analysis based on exercise intensity for bone mineral density**

**12.9.17 Regression analysis based on exercise intensity for body mass index**

**12.9.18 Regression analysis based on exercise intensity for fat mass percentage**

**12.9.19 Regression analysis based on exercise intensity for depressive symptoms**

**12.9.20 Regression analysis based on exercise intensity for social function**

**12.9.21 Regression analysis based on exercise intensity for executive function**

**12.9.22 Regression analysis based on exercise intensity for pro-inflammatory factors**

**12.9.23 Regression analysis based on exercise intensity for anti-inflammatory factors**

**12.9.24 Regression analysis based on exercise intensity for cognitive function**

**12.10 Gender regression analysis**

**12.10.1 Regression analysis based on gender for quality of life scale**

**12.10.2 Regression analysis based on gender for fatigue**

**12.10.3 Regression analysis based on gender for quality of life**

**12.10.4 Regression analysis based on gender for lower body muscle strength**

**12.10.5 Regression analysis based on gender for upper body muscle strength**

**12.10.6 Regression analysis based on gender for trunk muscle strength**

**12.10.7 Regression analysis based on gender for muscle strength**

**12.10.8 Regression analysis based on gender for six-minute walk test**

**12.10.9 Regression analysis based on gender for balance**

**12.10.10 Regression analysis based on gender for athletic performance**

**12.10.11 Regression analysis based on gender for physical activity level**

**12.10.12 Regression analysis based on gender for physical activity behaviour**

**12.10.13 Regression analysis based on gender for peak oxygen uptake**

**12.10.14 Regression analysis based on gender for cardiorespiratory function**

**12.10.15 Regression analysis based on gender for bone mineral density**

**12.10.16 Regression analysis based on gender for body mass index**

**12.10.17 Regression analysis based on gender for fat mass percentage**

**12.10.18 Regression analysis based on gender for depressive symptoms**

**12.10.19 Regression analysis based on gender for social function**

**12.10.20 Regression analysis based on gender for executive function**

**12.10.21 Regression analysis based on gender for pro-inflammatory factors**

**12.10.22 Regression analysis based on gender for anti-inflammatory factors**

**12.10.23 Regression analysis based on gender for cognitive function**

**13.1 Region subgroup analysis**

**13.1.1 Subgroup analysis based on region for quality of life scale**

**13.1.2 Subgroup analysis based on region for fatigue**

**13.1.3 Subgroup analysis based on region for quality of life**

**13.1.4 Subgroup analysis based on region for lower body muscle strength**

**13.1.5 Subgroup analysis based on region for trunk muscle strength**

**13.1.6 Subgroup analysis based on region for muscle strength**

**13.1.7 Subgroup analysis based on region for six-minute walk test**

**13.1.8 Subgroup analysis based on region for balance**

**13.1.9 Subgroup analysis based on region for flexibility**

**13.1.10 Subgroup analysis based on region for athletic performance**

**13.1.11 Subgroup analysis based on region for physical activity level**

**13.1.12 Subgroup analysis based on region for physical activity behaviour**

**13.1.13 Subgroup analysis based on region for peak oxygen uptake**

**13.1.14 Subgroup analysis based on region for cardiorespiratory function**

**13.1.15 Subgroup analysis based on region for bone mineral density**

**13.1.16 Subgroup analysis based on region for body mass index**

**13.1.17 Subgroup analysis based on region for fat mass percentage**

**13.1.18 Subgroup analysis based on region for NK cell level**

**13.1.19 Subgroup analysis based on region for depressive symptoms**

**13.1.20 Subgroup analysis based on region for social function**

**13.1.21 Subgroup analysis based on region for executive function**

**13.1.22 Subgroup analysis based on region for pro-inflammatory factors**

**13.1.23 Subgroup analysis based on region for anti-inflammatory factors**

**13.1.24 Subgroup analysis based on region for cognitive function**

**13.2 Age subgroup analysis**

**13.2.1 Subgroup analysis based on age for quality of life scale**

**13.2.2 Subgroup analysis based on age for fatigue**

**13.2.3 Subgroup analysis based on age for quality of life**

**13.2.4 Subgroup analysis based on age for lower body muscle strength**

**13.2.5 Subgroup analysis based on age for upper body muscle strength**

**13.2.6 Subgroup analysis based on age for trunk muscle strength**

**13.2.7 Subgroup analysis based on age for muscle strength**

**13.2.8 Subgroup analysis based on age for six-minute walk test**

**13.2.9 Subgroup analysis based on age for balance**

**13.2.10 Subgroup analysis based on age for flexibility**

**13.2.11 Subgroup analysis based on age for athletic performance**

**13.2.12 Subgroup analysis based on age for physical activity level**

**13.2.13 Subgroup analysis based on age for physical activity behaviour**

**13.2.14 Subgroup analysis based on age for peak oxygen uptake**

**13.2.15 Subgroup analysis based on age for cardiorespiratory function**

**13.2.16 Subgroup analysis based on age for bone mineral density**

**13.2.17 Subgroup analysis based on age for body mass index**

**13.2.18 Subgroup analysis based on age for fat mass percentage**

**13.2.19 Subgroup analysis based on age for NK cell level**

**13.2.20 Subgroup analysis based on age for depressive symptoms**

**13.2.21 Subgroup analysis based on age for social function**

**13.2.22 Subgroup analysis based on age for executive function**

**13.2.23 Subgroup analysis based on age for pro-inflammatory factors**

**13.2.24 Subgroup analysis based on age for anti-inflammatory factors**

**13.2.25 Subgroup analysis based on age for cognitive function**
