# Supplementary material for: The effect of physical activity on health-related outcomes in children and adolescents with cancer: a systematic review and meta-analysis
Source: Front Oncol. 2026 Mar 31;16:1773060. doi: 10.3389/fonc.2026.1773060 (PMC13076115; doi:10.3389/fonc.2026.1773060)
Supplement: Supplementary file 2 [file DataSheet2.docx]

**13.3 Medicine subgroup analysis**

**13.3.1 Subgroup analysis based on medicine for quality of life scale**

**
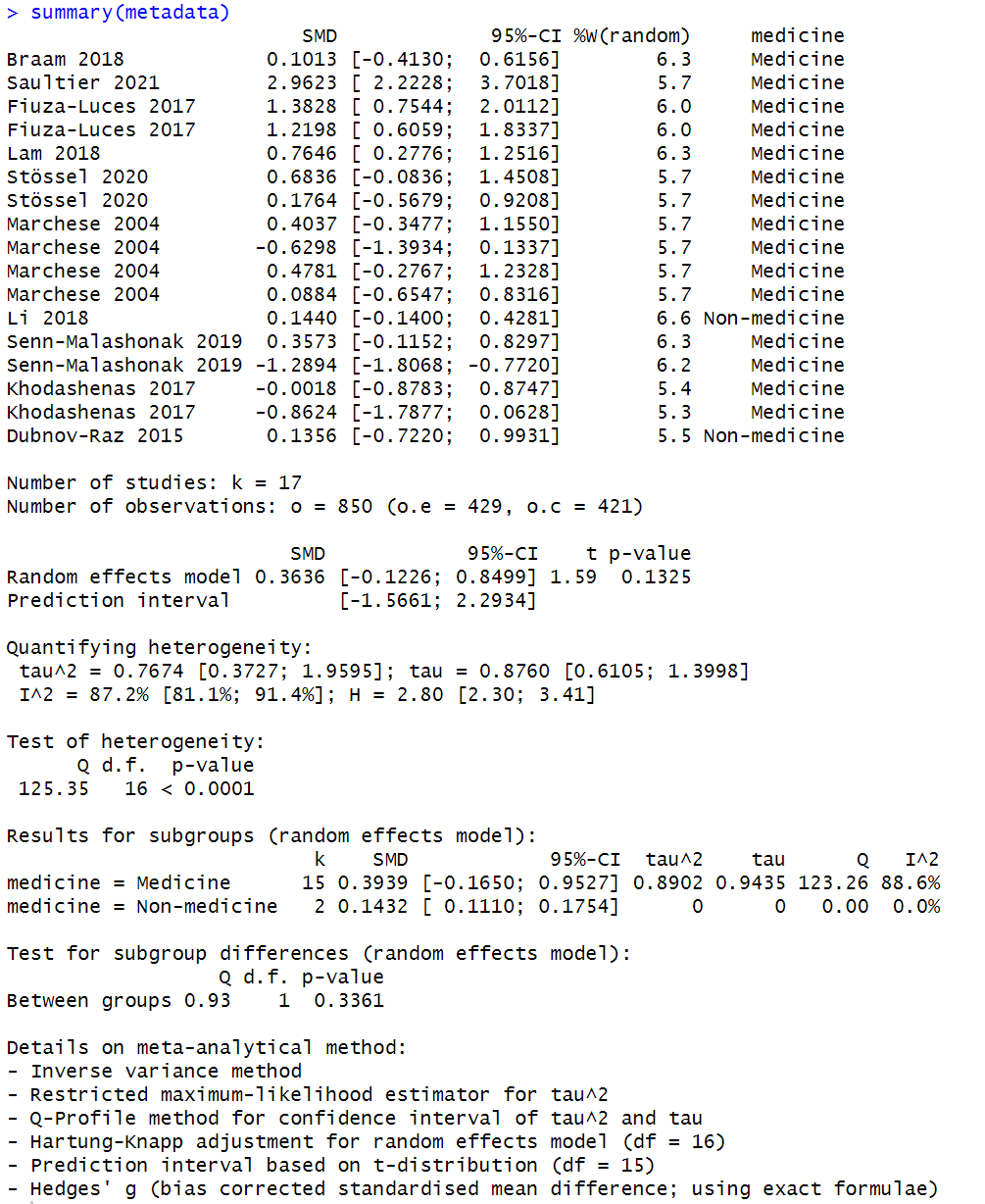
**

**
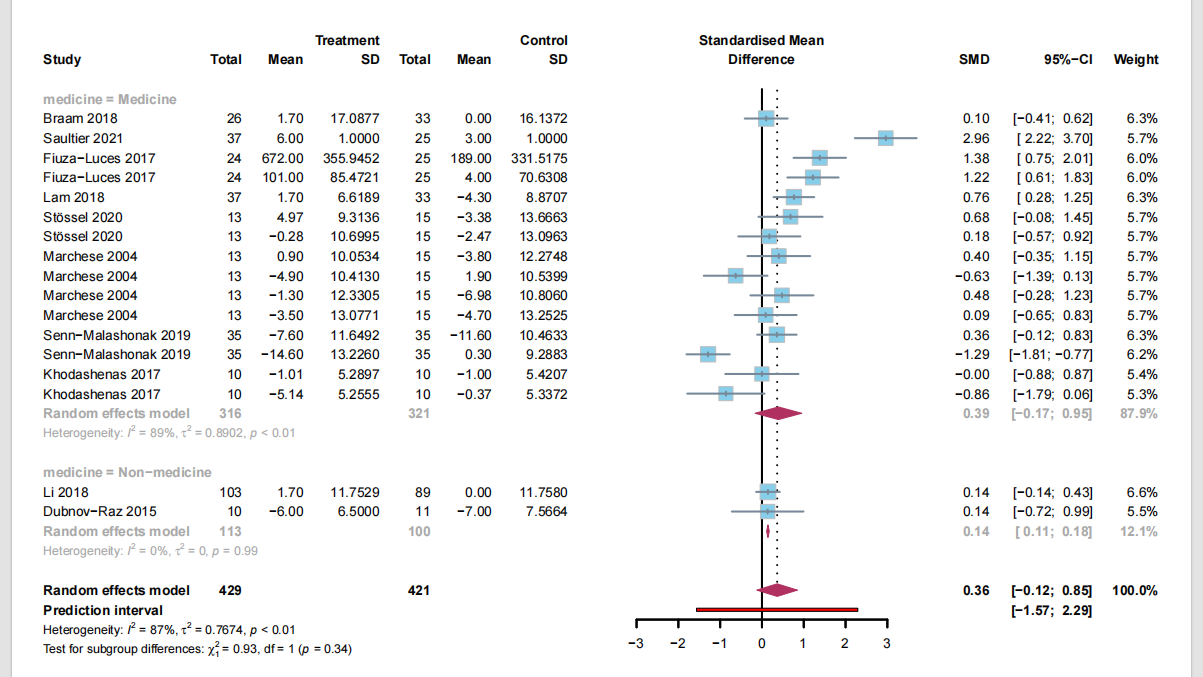
**

**13.3.2 Subgroup analysis based on medicine for fatigue**

**
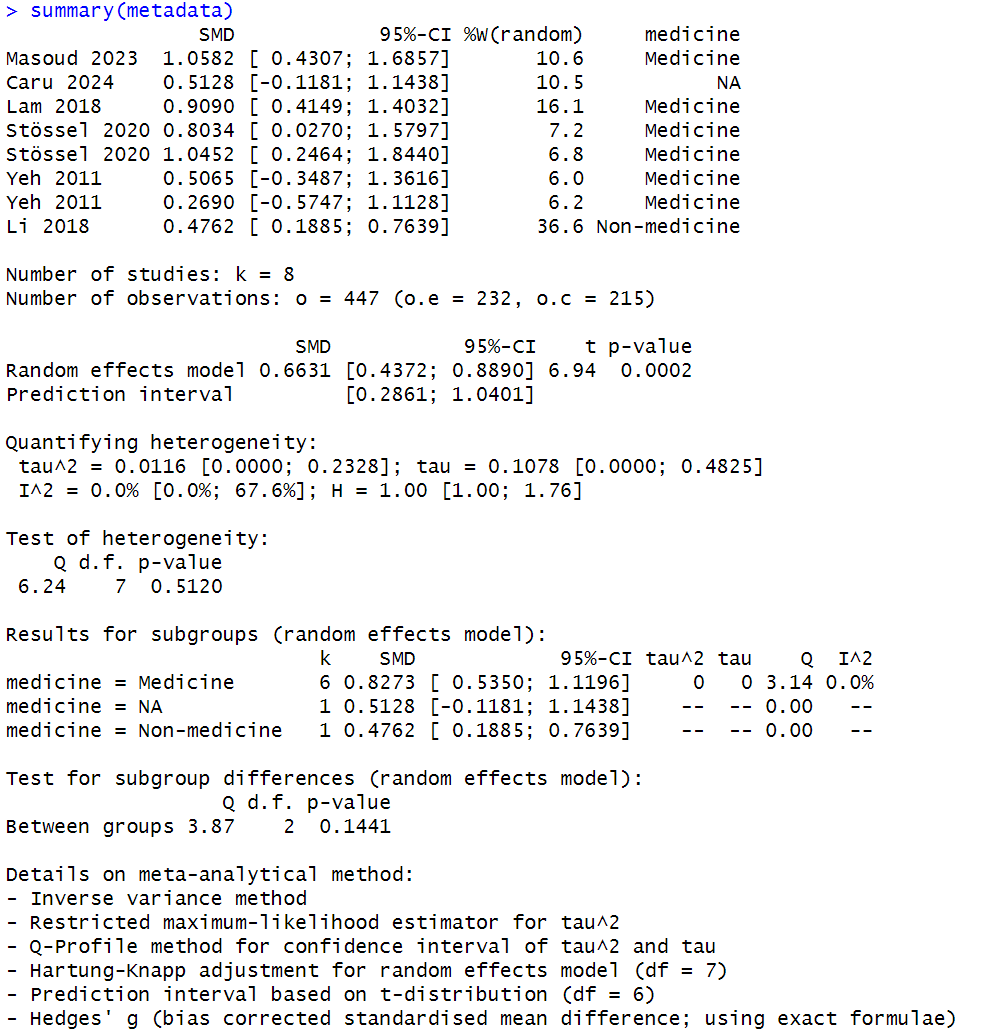
**

**
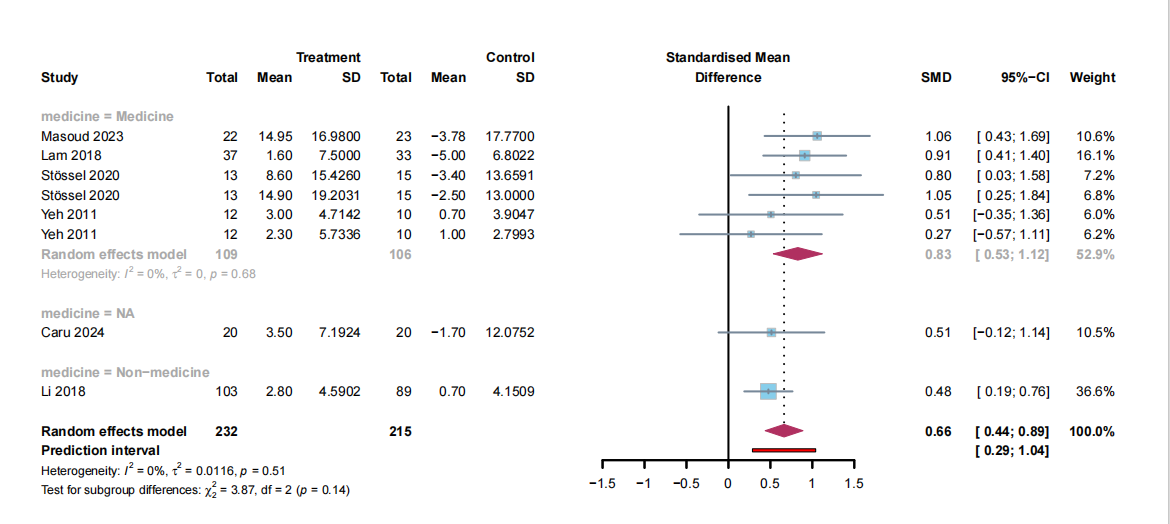
**

**13.3.3 Subgroup analysis based on medicine for quality of life**

**
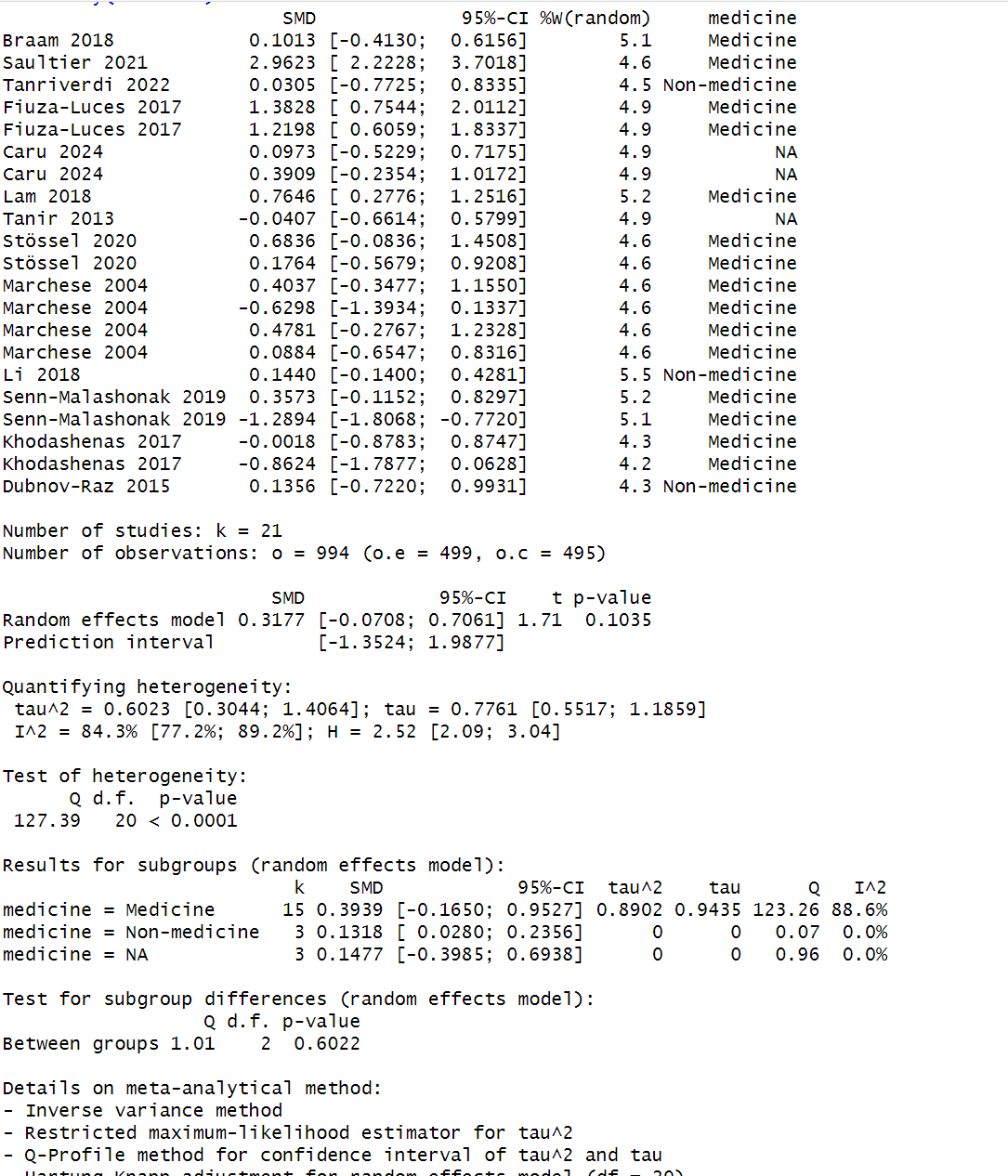
**

**
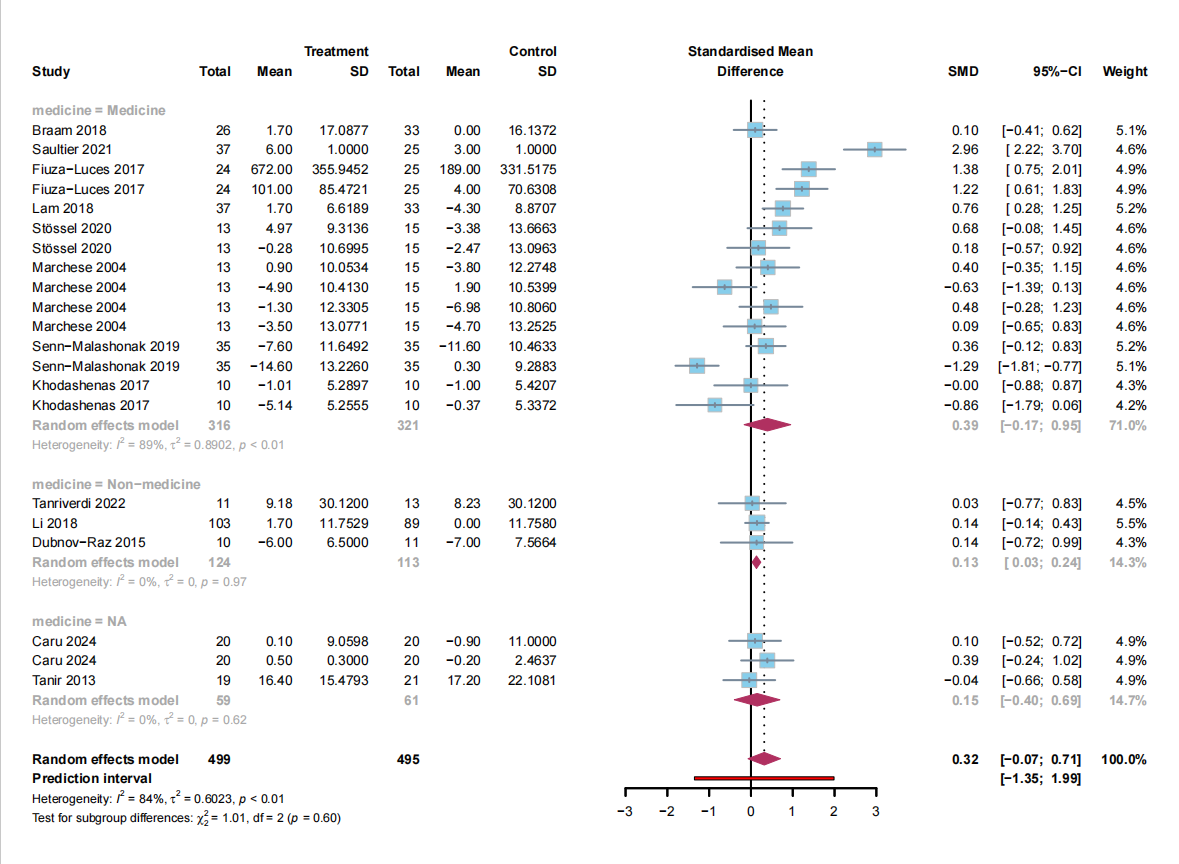
**

**13.3.4 Subgroup analysis based on medicine for lower body muscle strength**

**
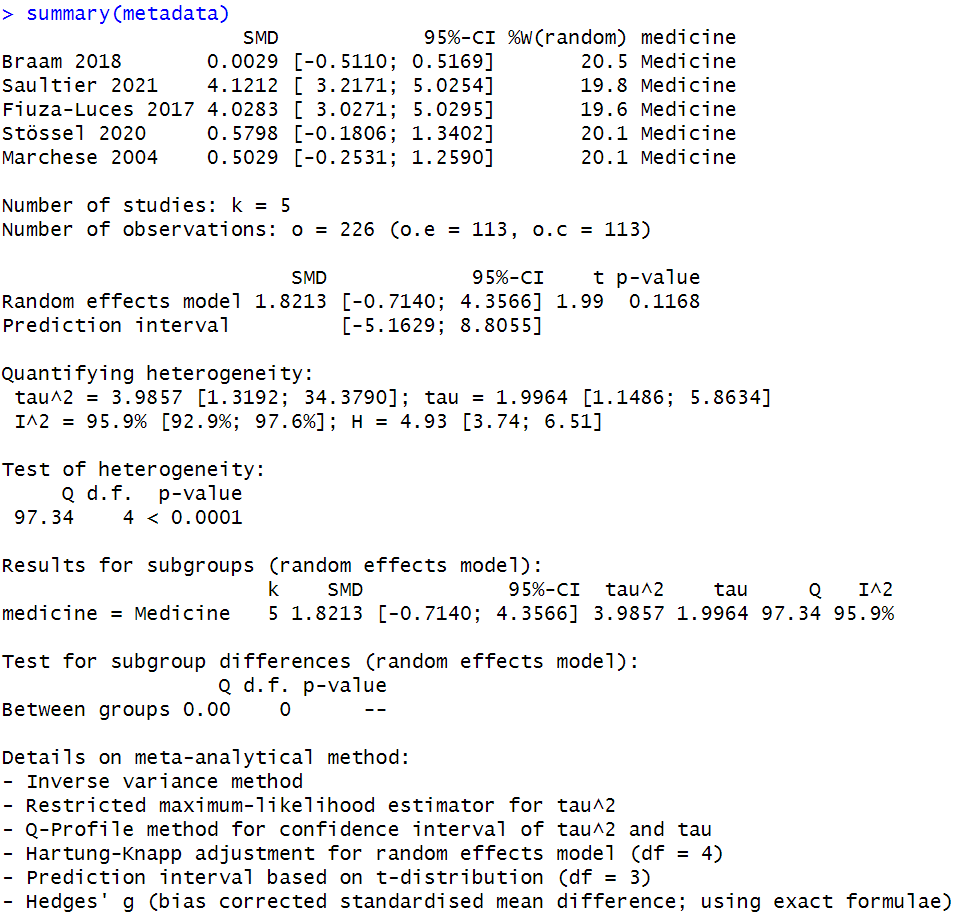
**

**
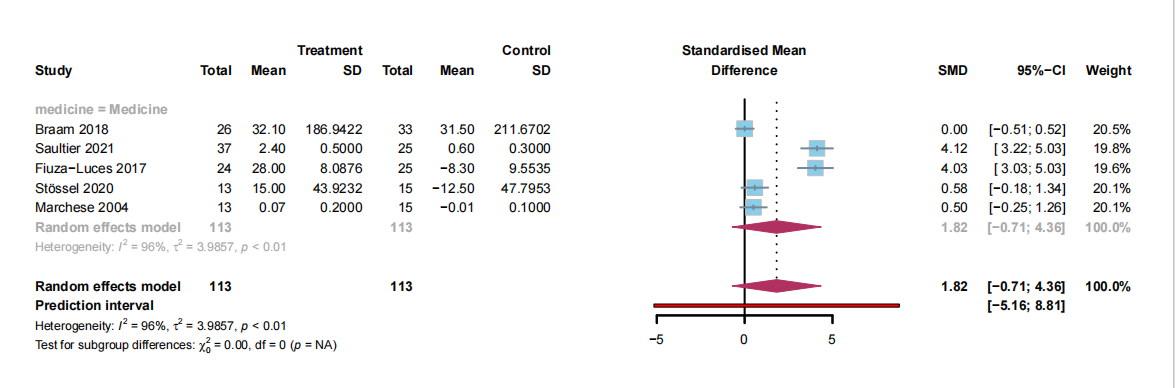
**

**13.3.5 Subgroup analysis based on medicine for trunk muscle strength**

**
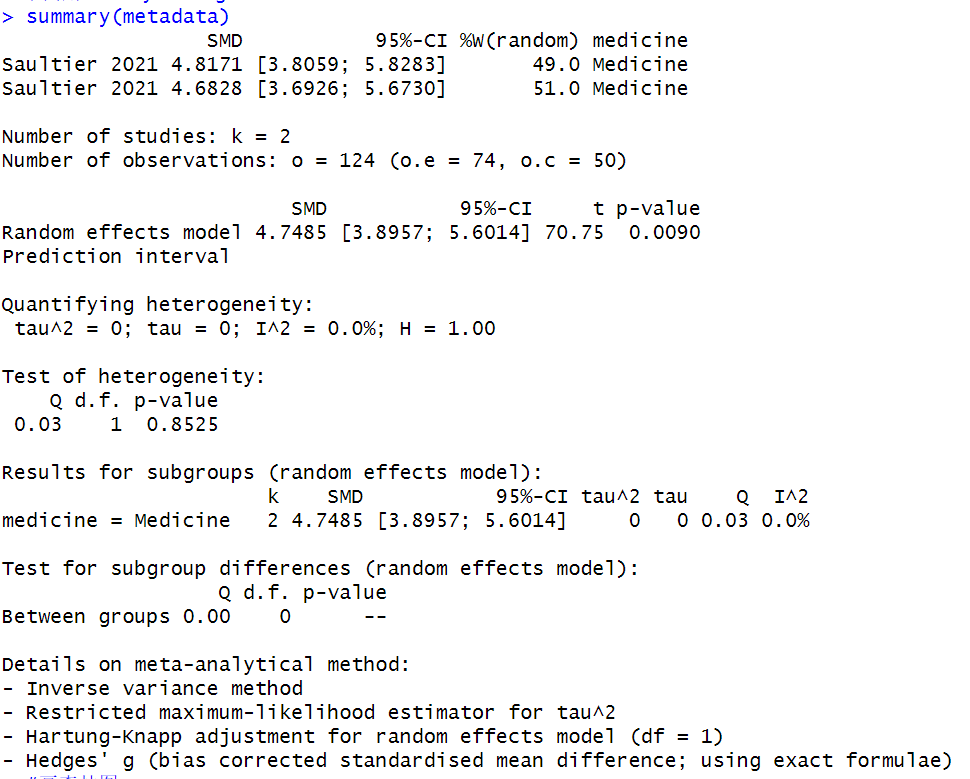
**

**
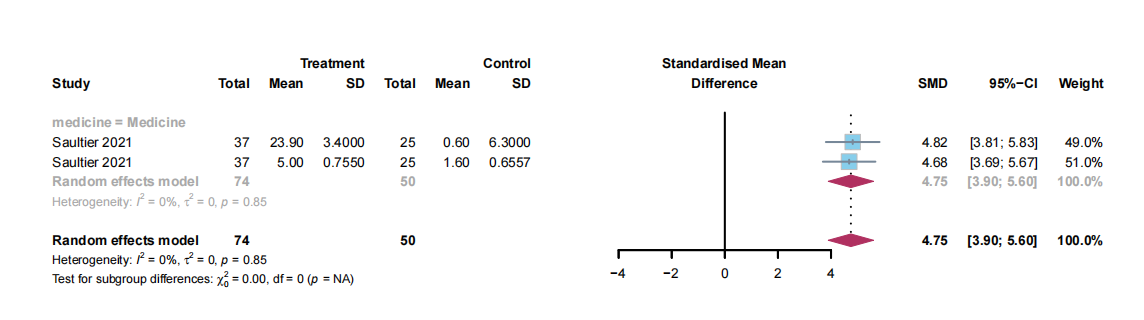
**

**13.3.6 Subgroup analysis based on medicine for muscle strength**

**
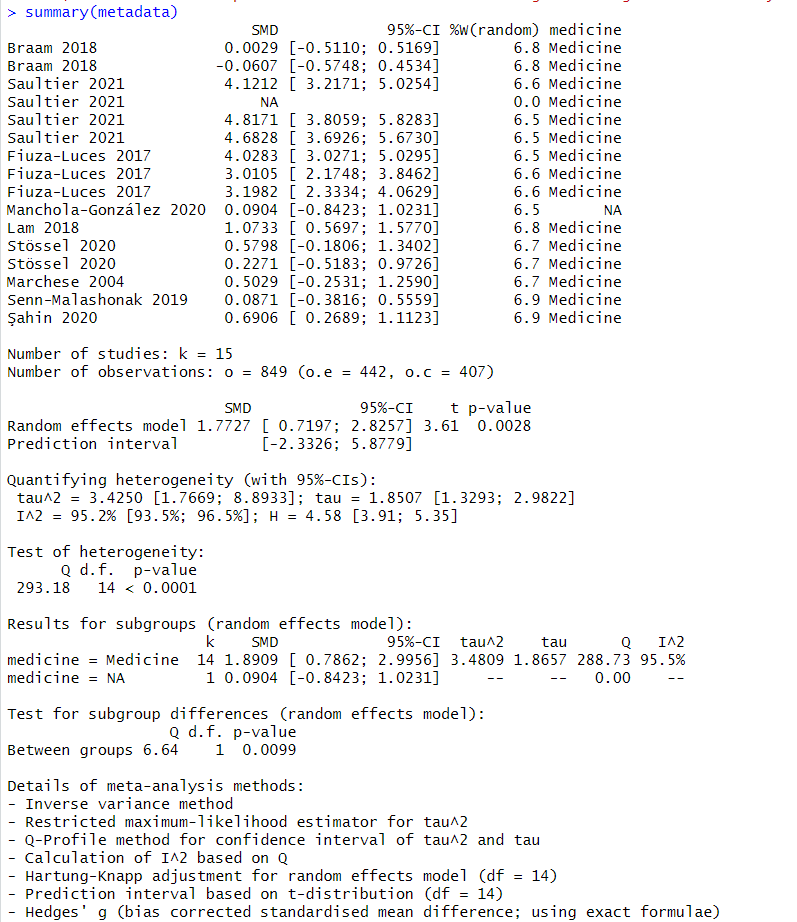
**

**
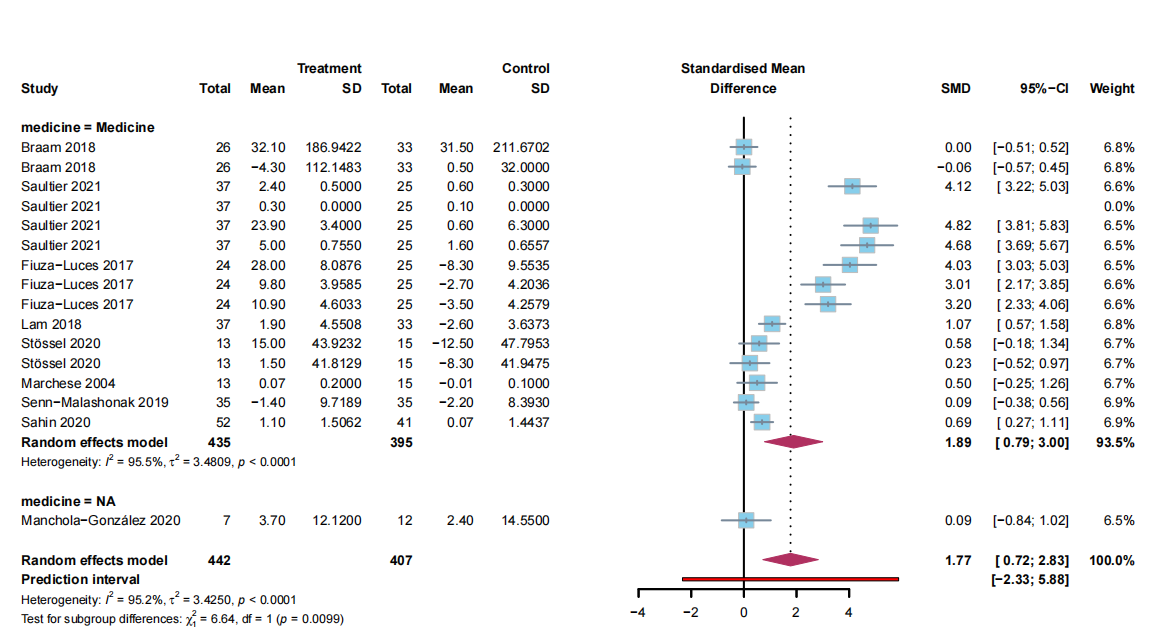
**

**13.3.7 Subgroup analysis based on medicine for six-minute walk test**

**
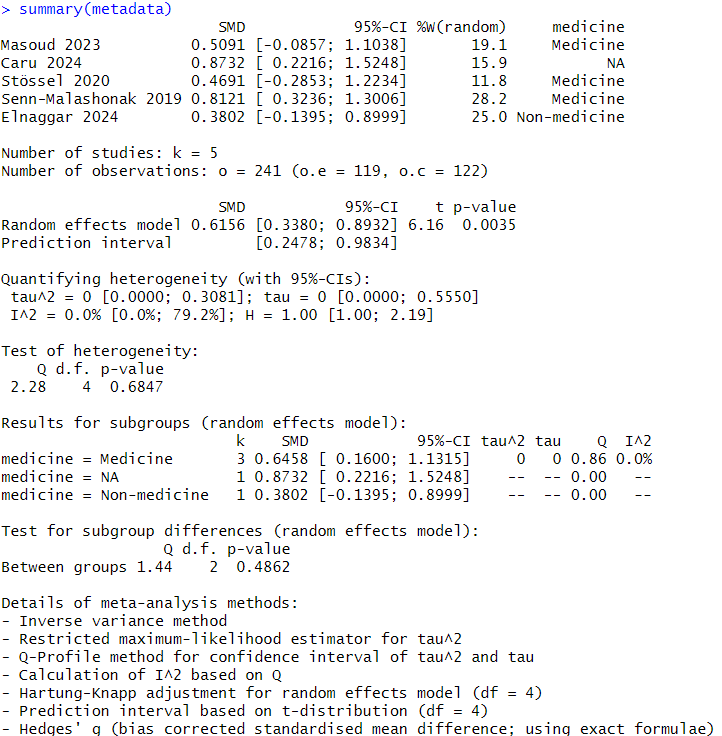
**

**
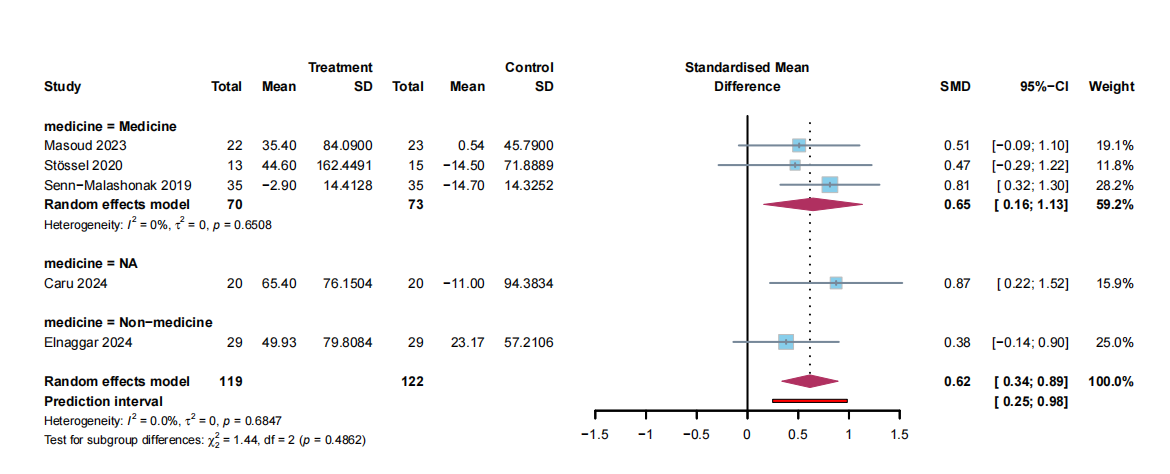
**

**13.3.8 Subgroup analysis based on medicine for balance**

**
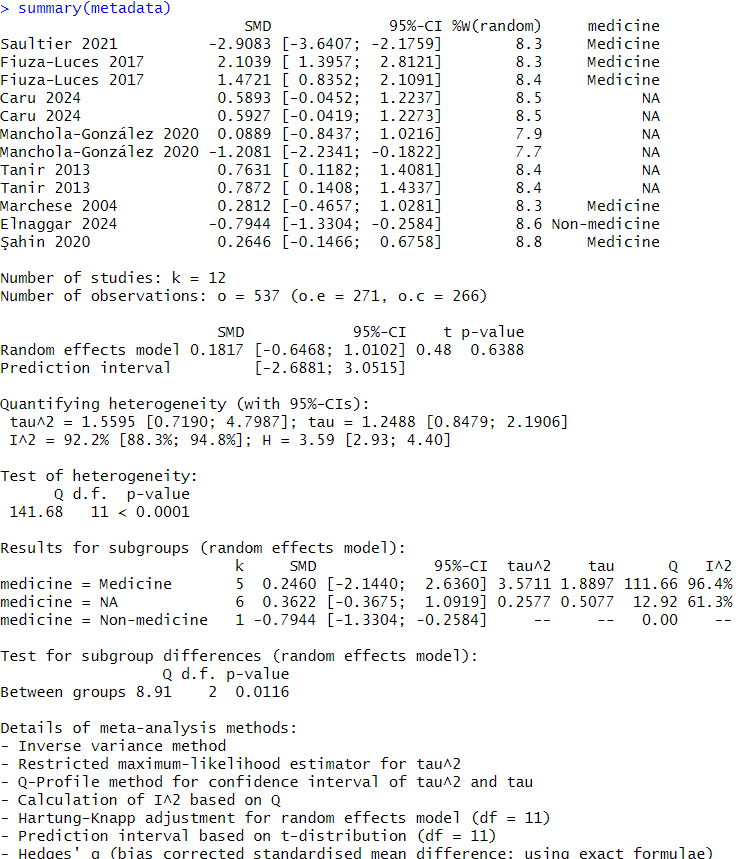
**

**
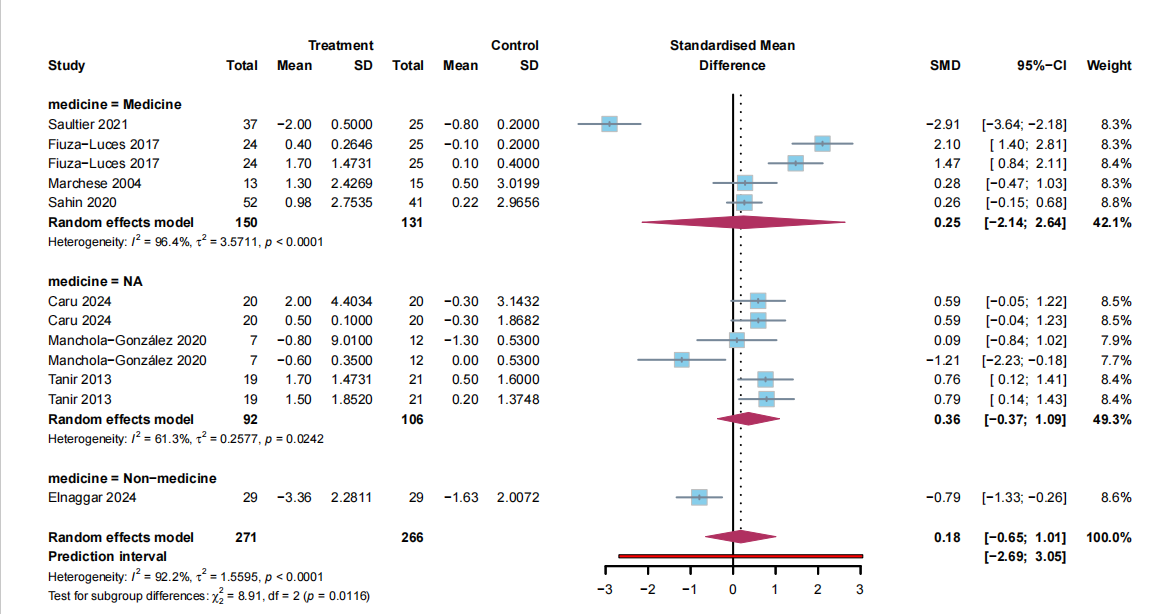
**

**13.3.9 Subgroup analysis based on medicine for flexibility**

**
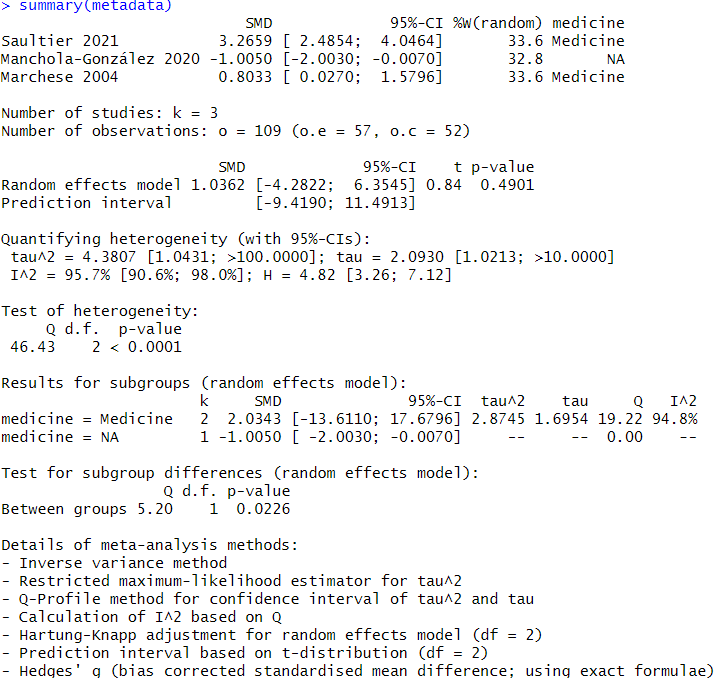
**

**
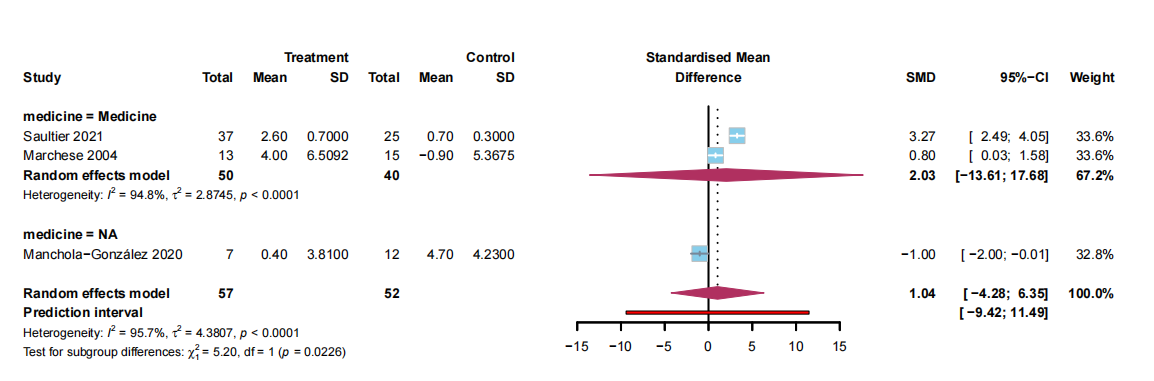
**

**13.3.10 Subgroup analysis based on medicine for athletic performance**

**
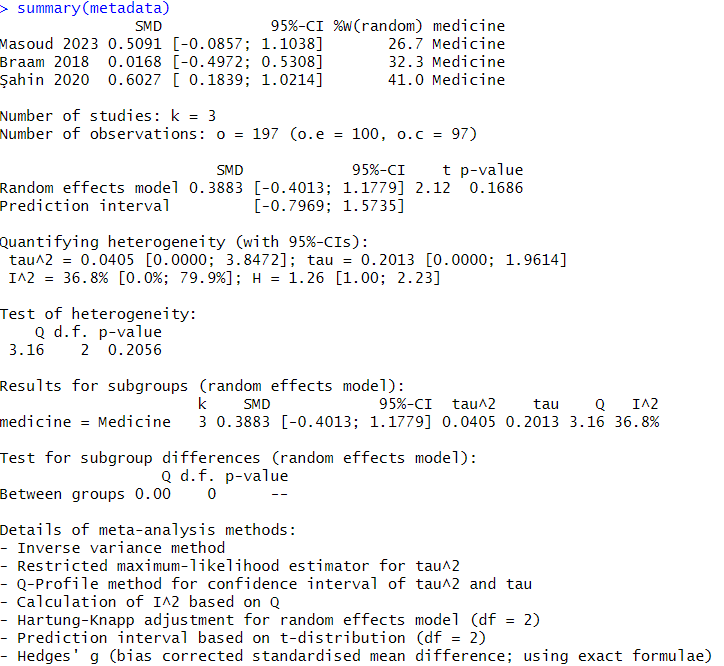
**

**
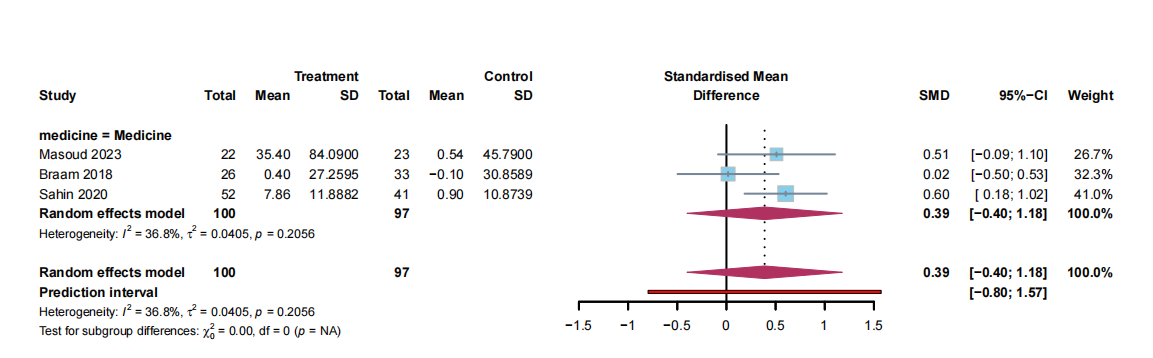
**

**13.3.11 Subgroup analysis based on medicine for physical activity level**

**
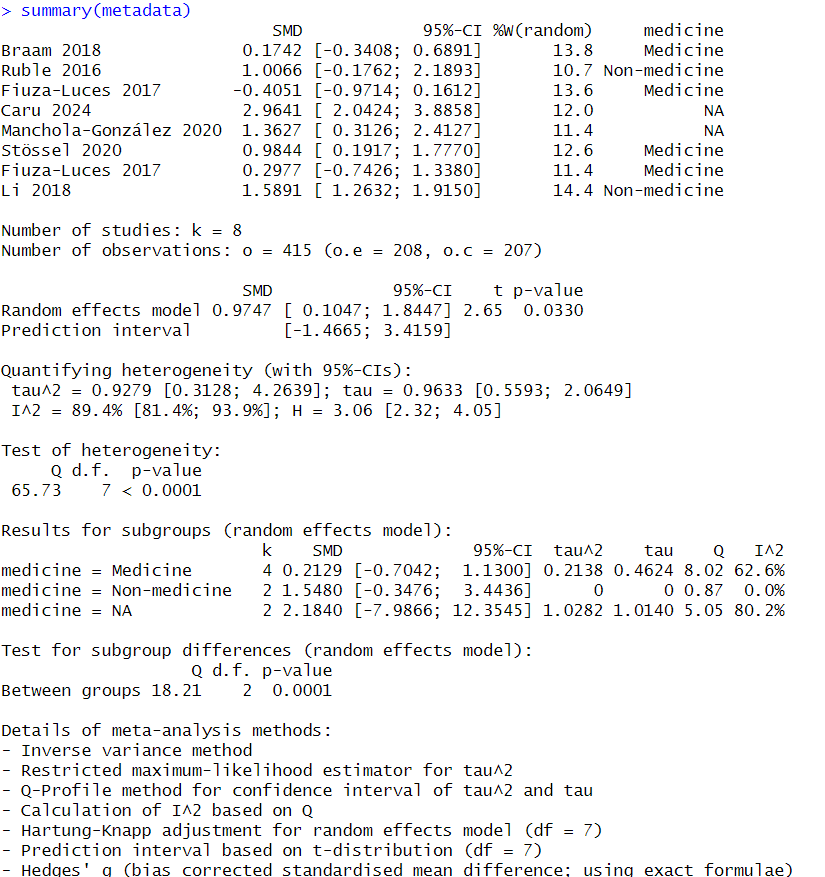
**

**
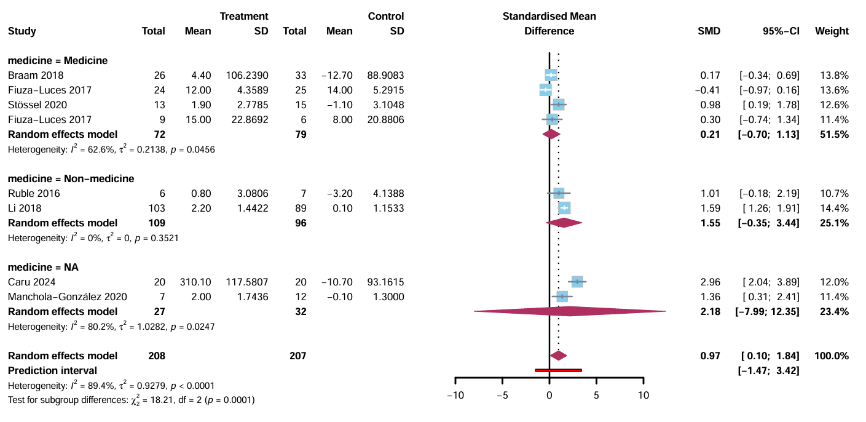
**

**13.3.12 Subgroup analysis based on medicine for physical activity behaviour**

**
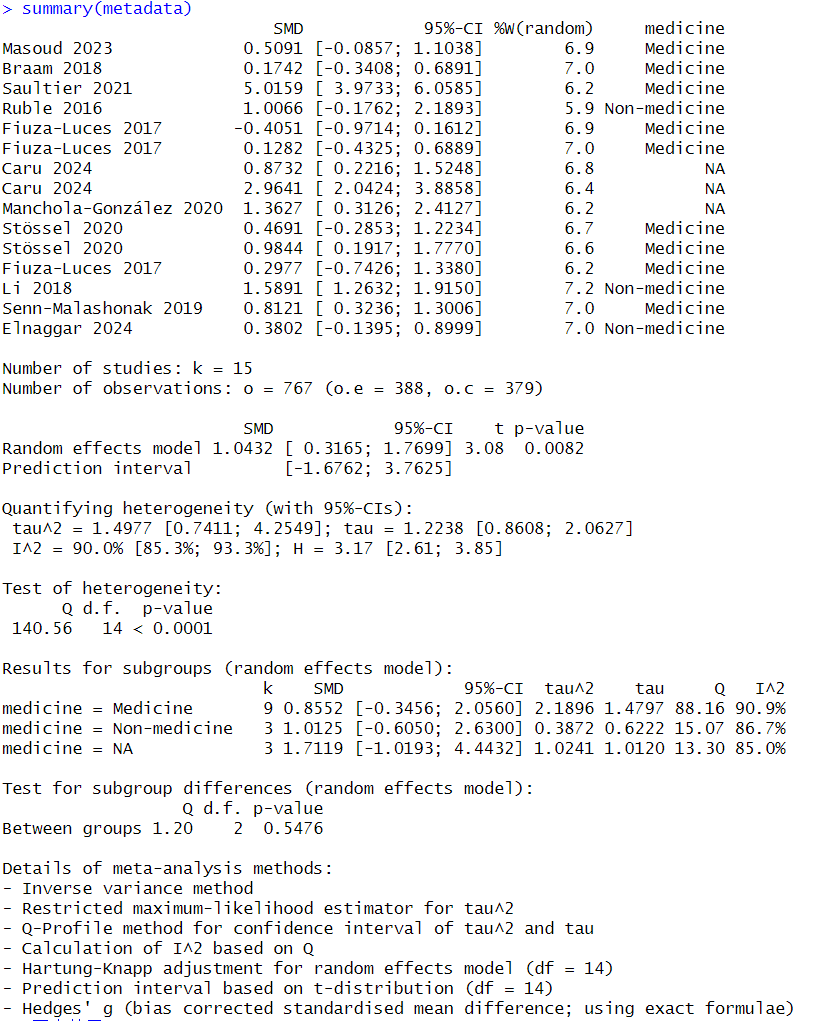
**

**
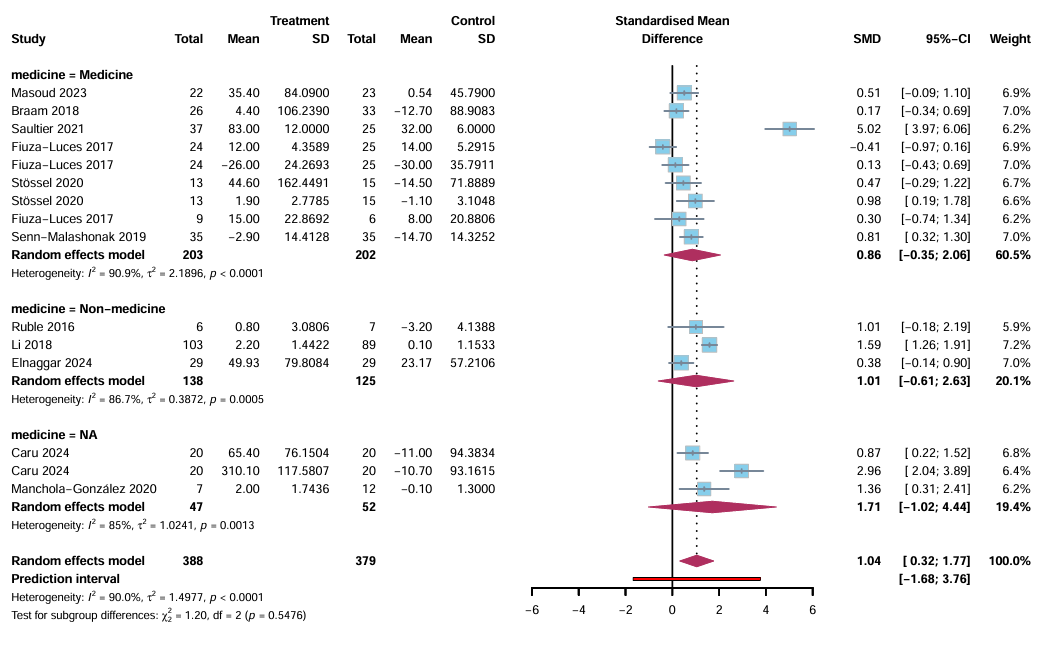
**

**13.3.13 Subgroup analysis based on medicine for peak oxygen uptake**

**
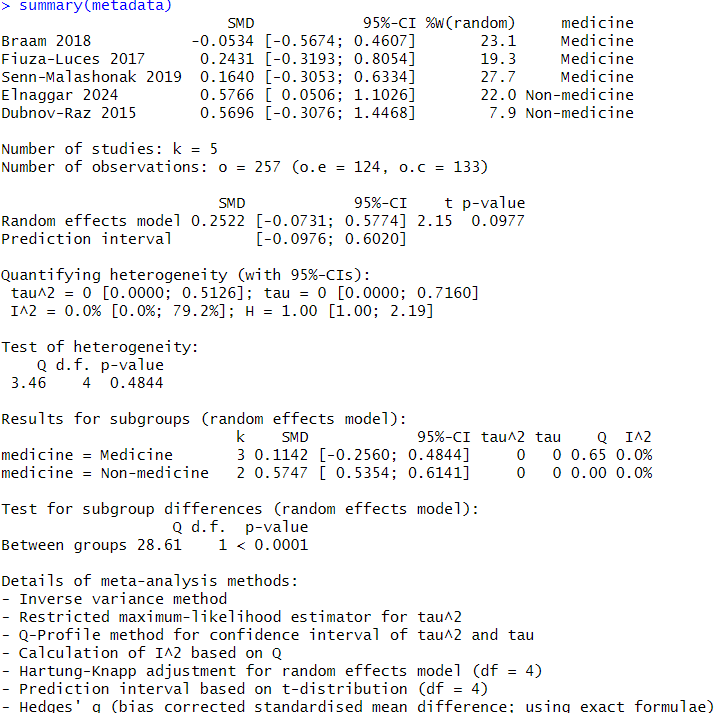
**

**
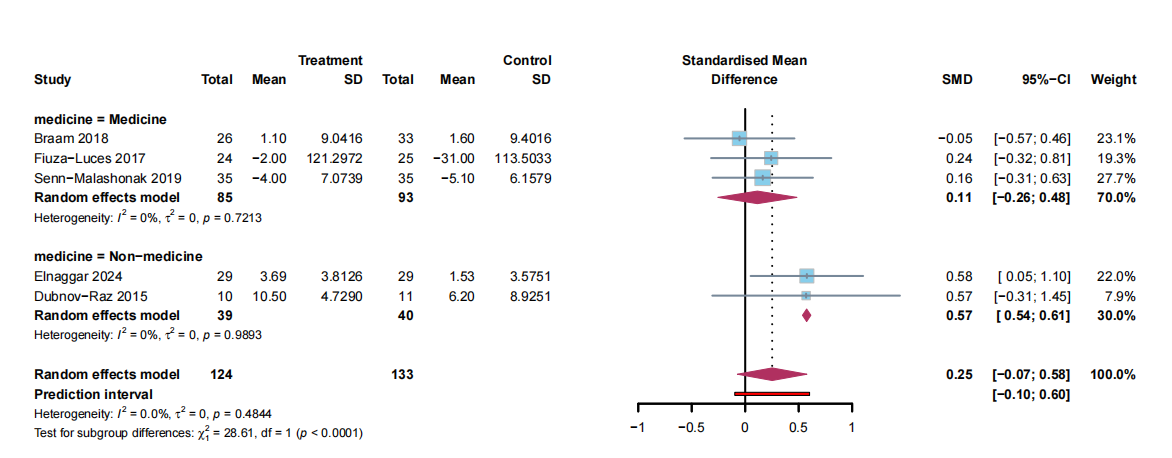
**

**13.3.14 Subgroup analysis based on medicine for cardiorespiratory function**

**
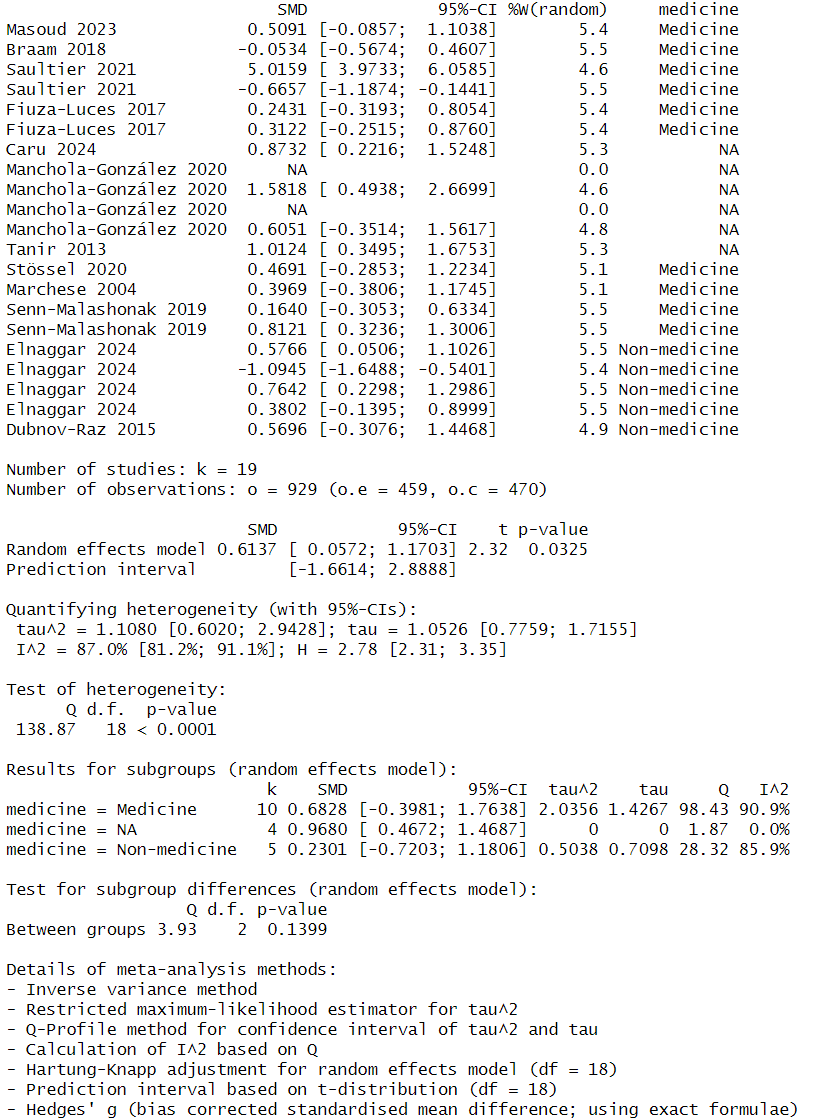
**

**
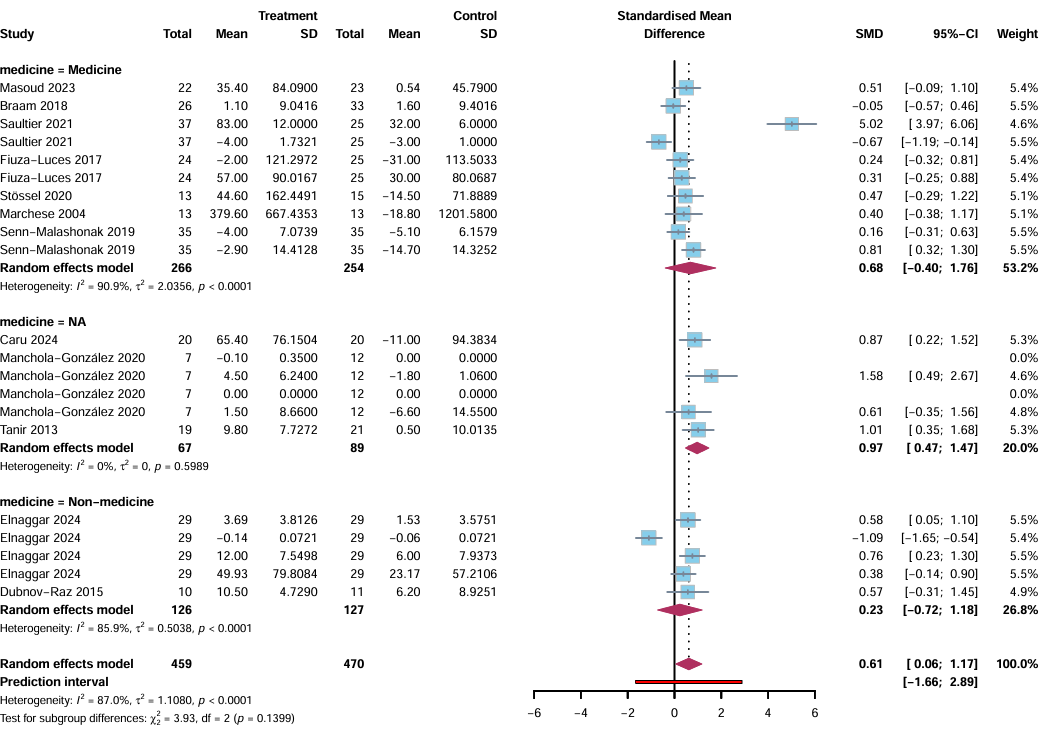
**

**13.3.15 Subgroup analysis based on medicine for bone mineral density**

**
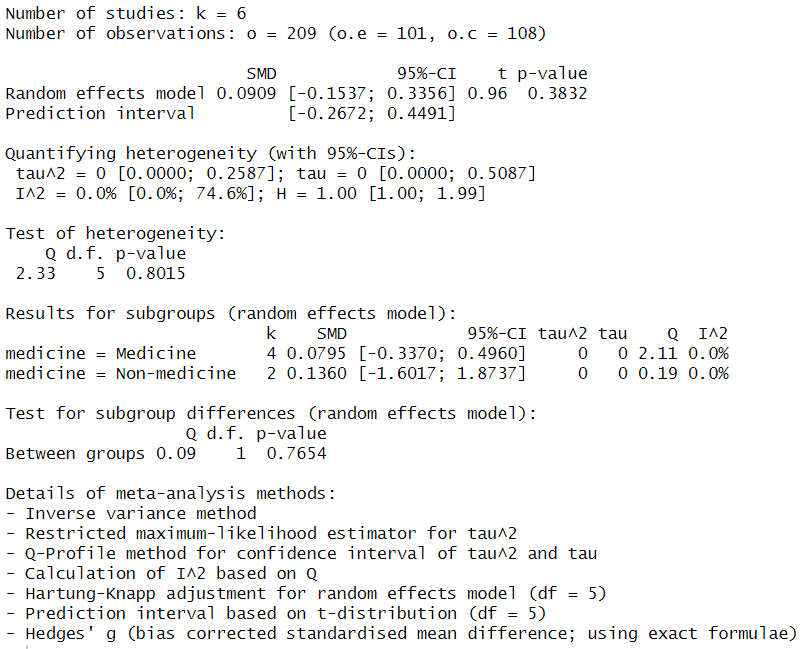
**

**
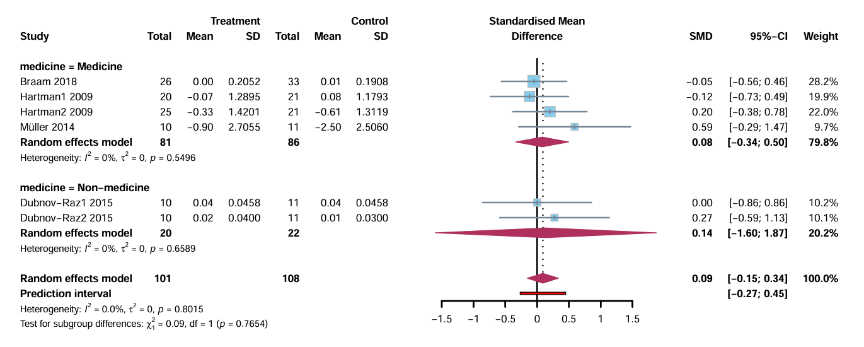
**

**13.3.16 Subgroup analysis based on medicine for body mass index**

**
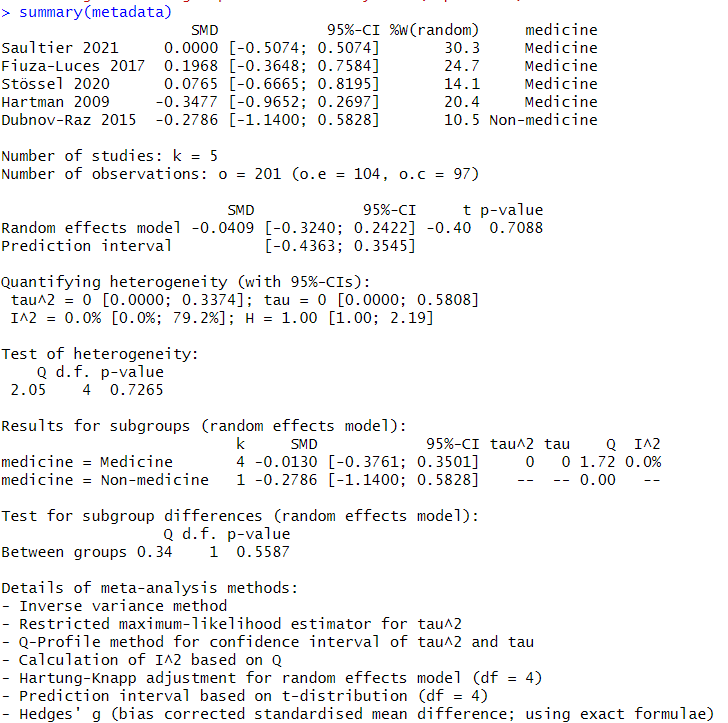
**

**
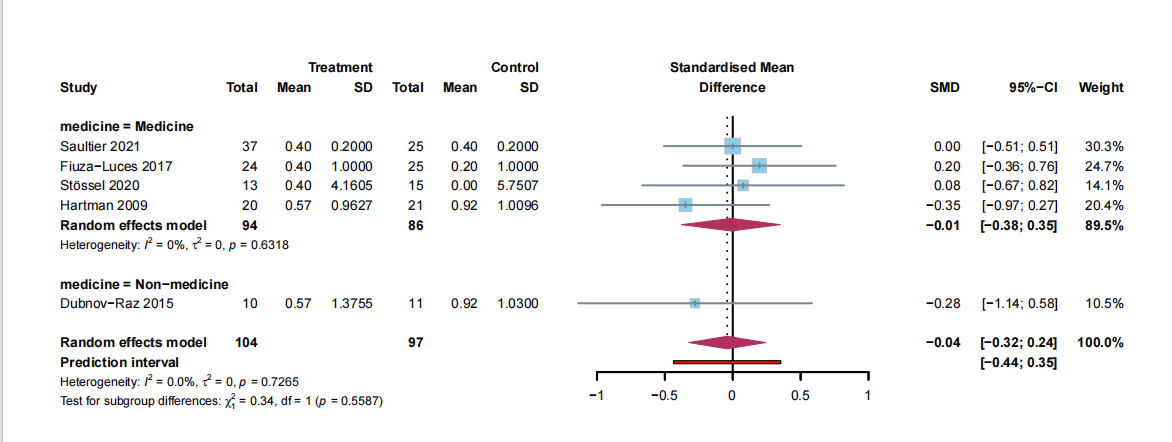
**

**13.3.17 Subgroup analysis based on medicine for fat mass percentage**

**
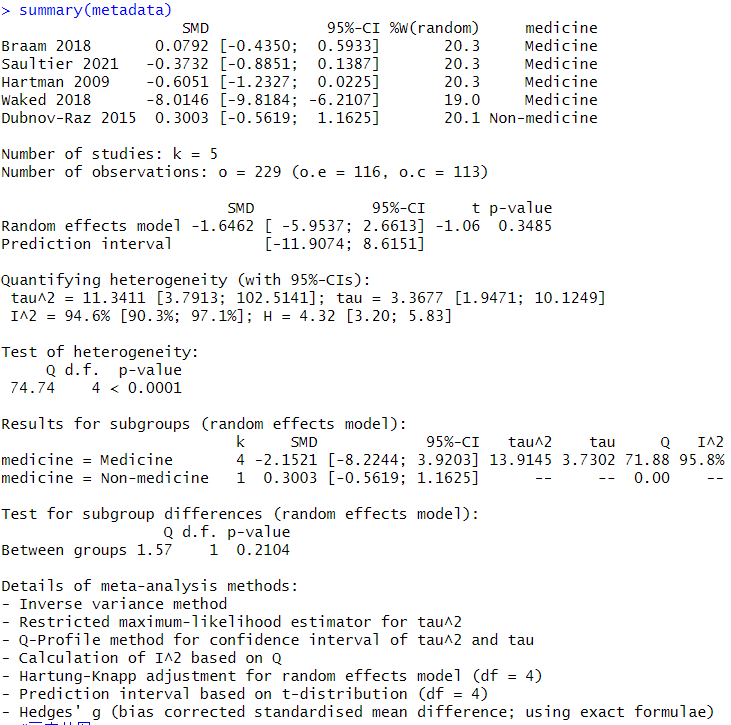
**

**
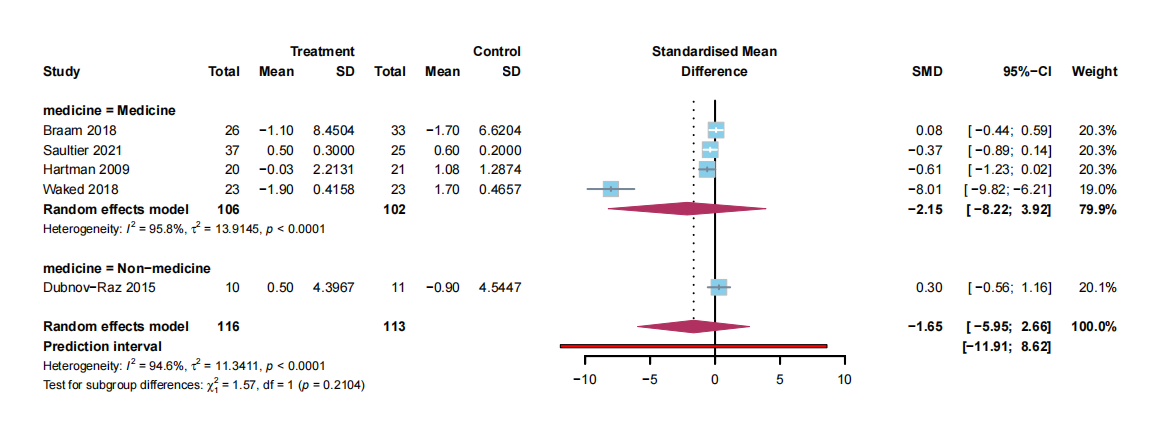
**

**13.3.18 Subgroup analysis based on medicine for NK cell level**

**
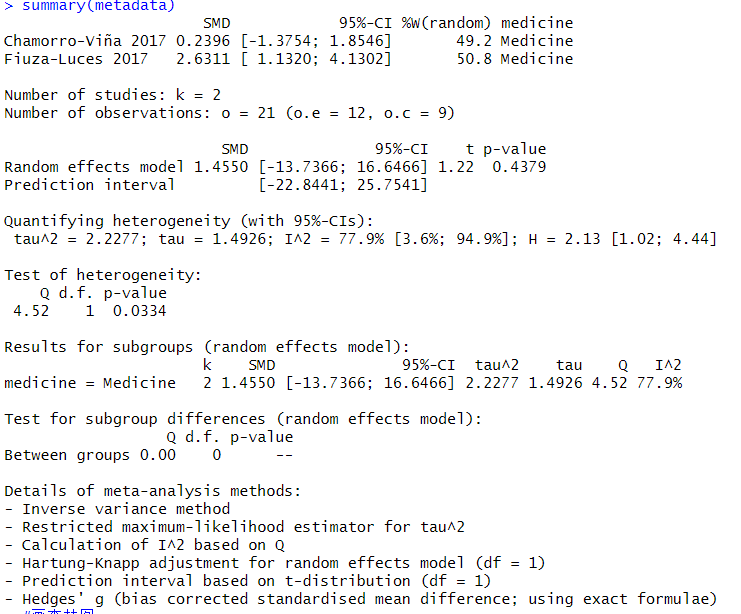
**

**
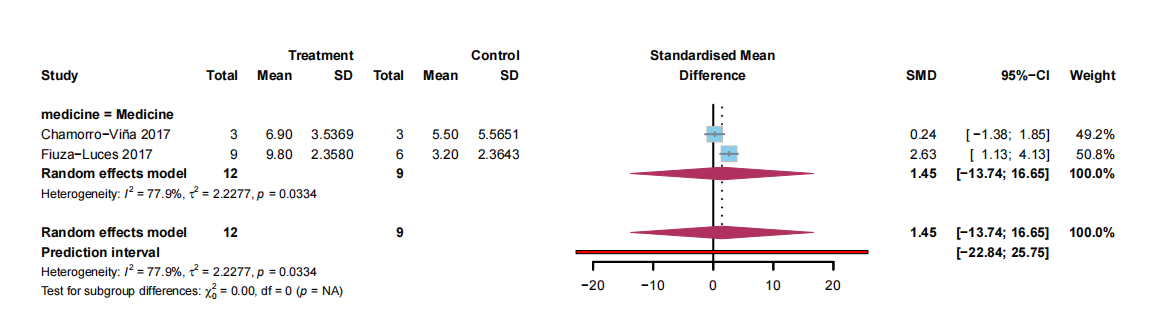
**

**13.3.19 Subgroup analysis based on medicine for depressive symptoms**

**
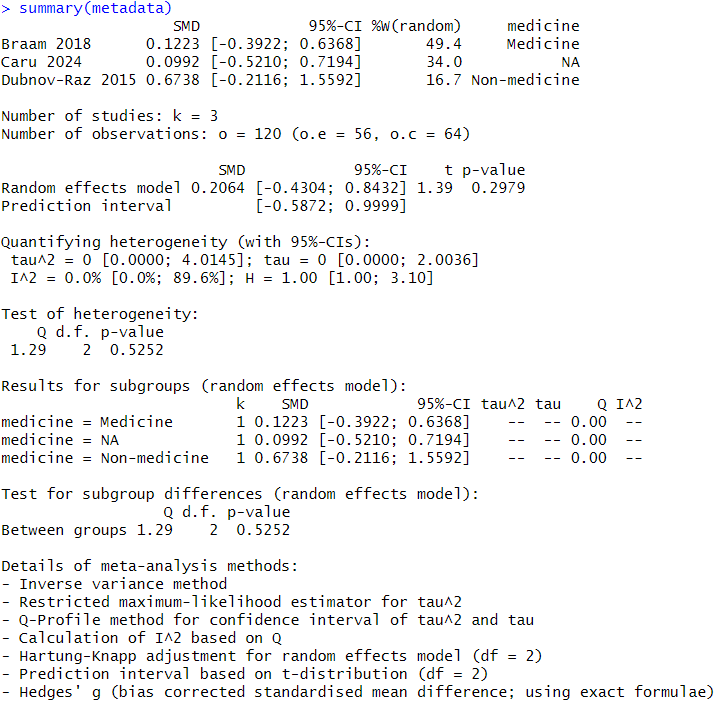
**

**
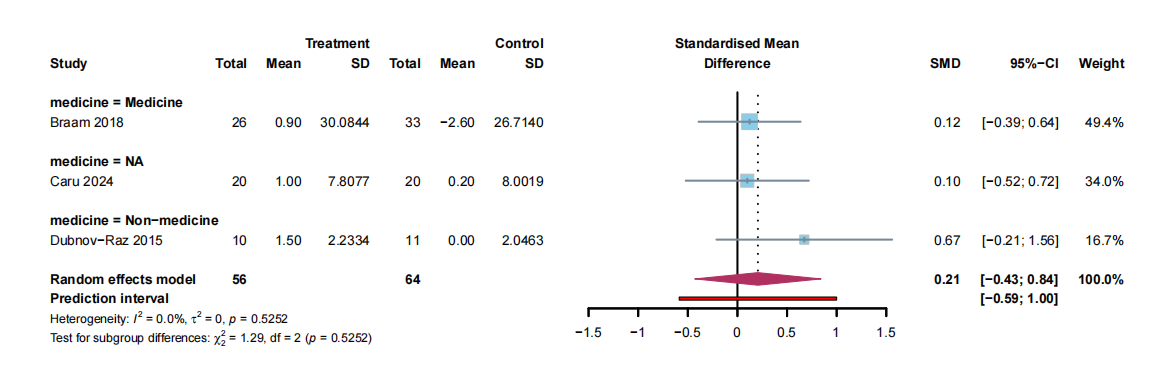
**

**13.3.20 Subgroup analysis based on medicine for social function**

**
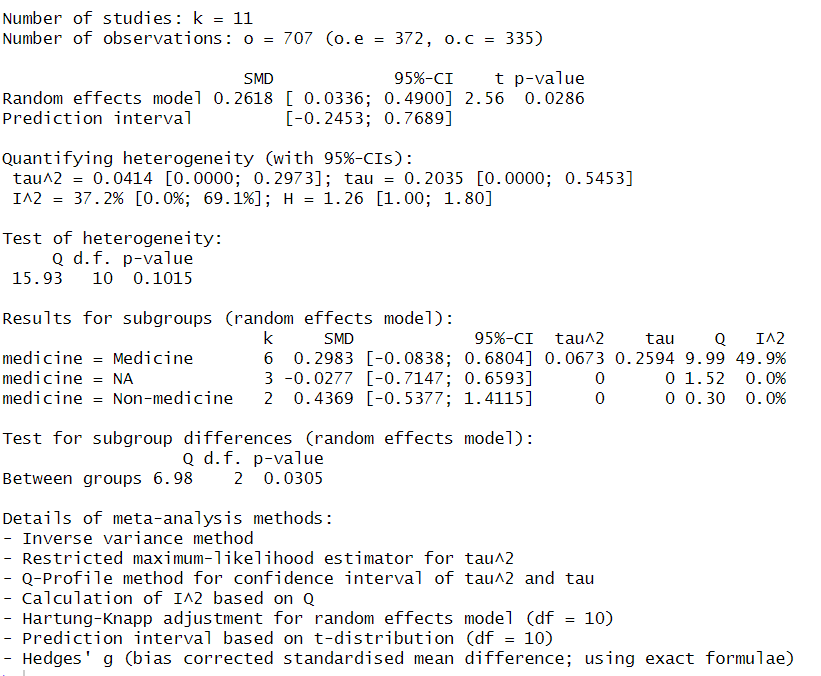
**

**
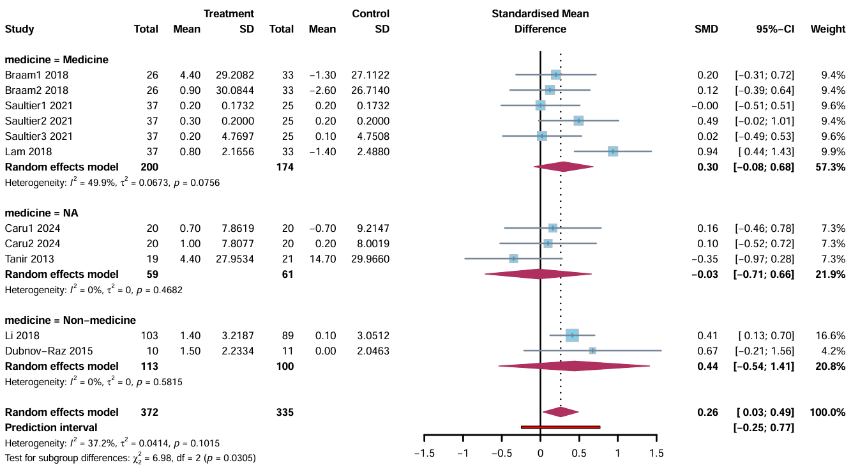
**

**13.3.21 Subgroup analysis based on medicine for executive function**

**
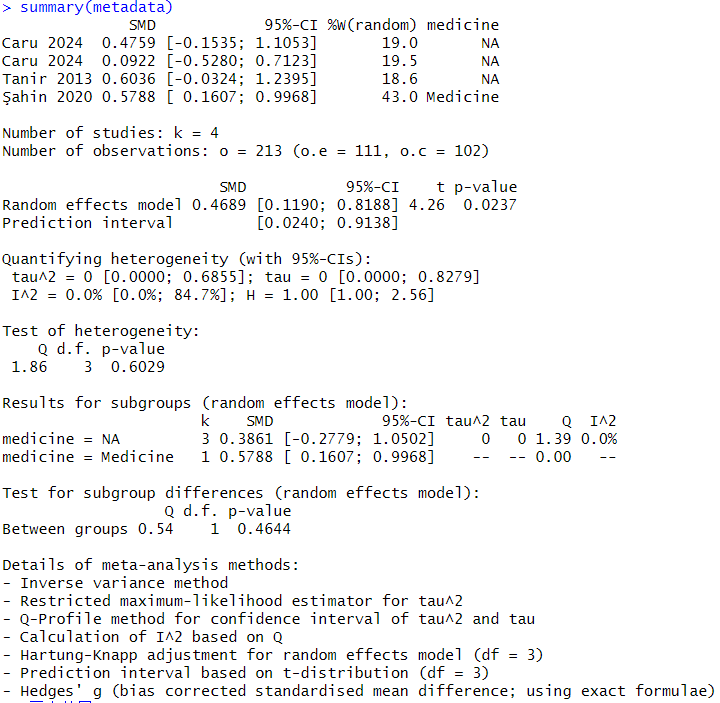
**

**
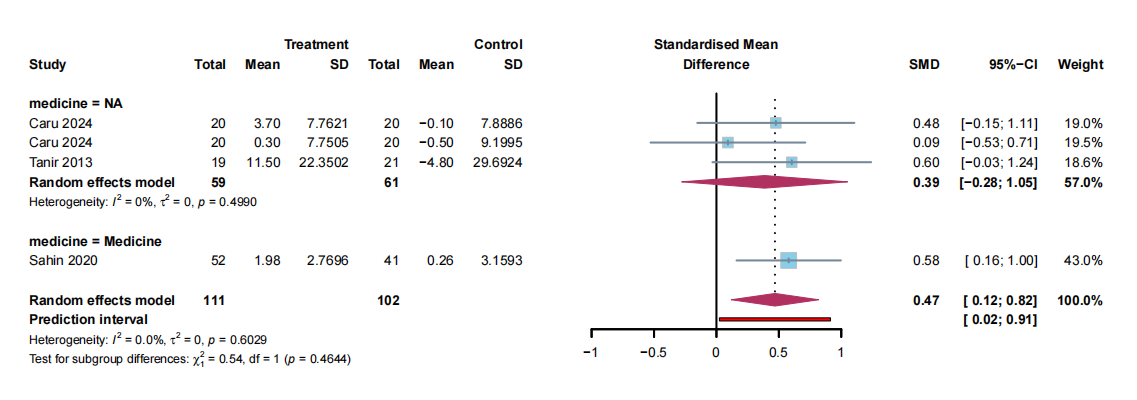
**

**13.3.22 Subgroup analysis based on medicine for pro-inflammatory factors**

**
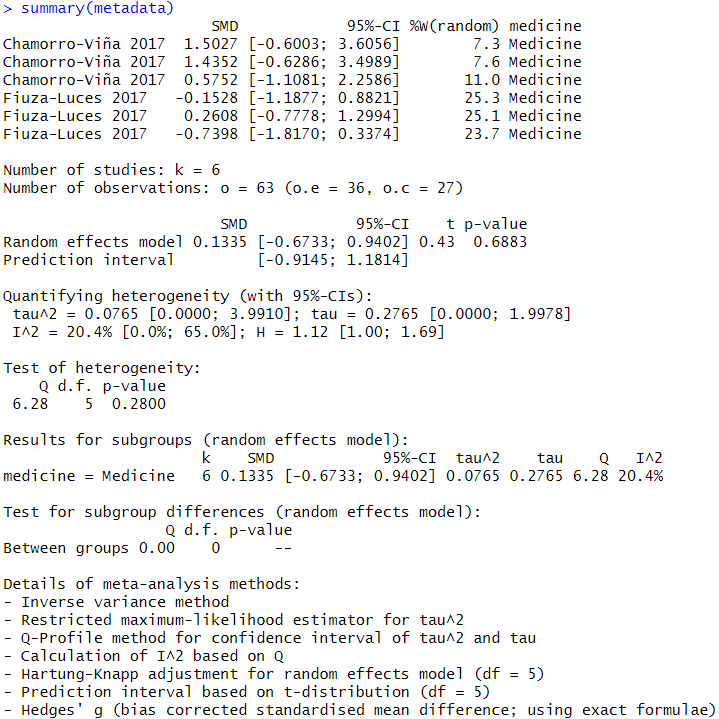
**

**
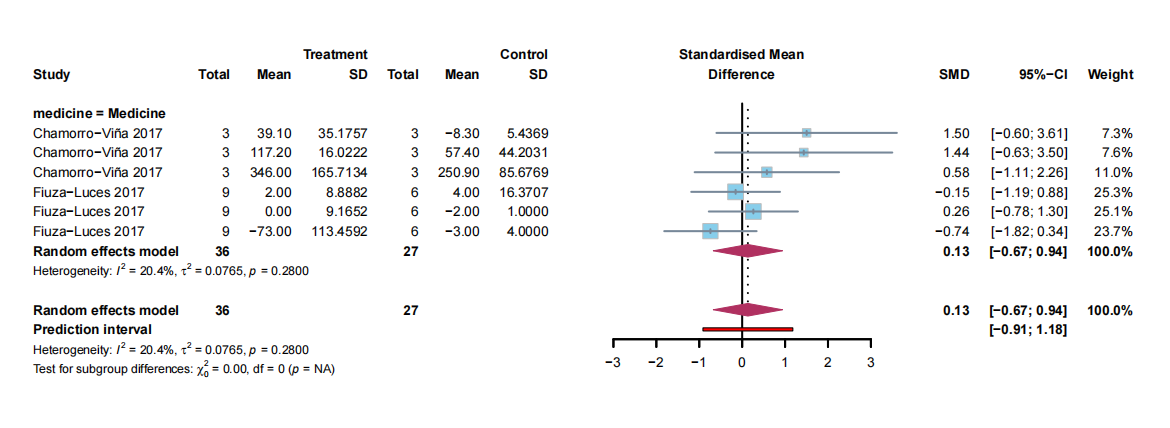
**

**13.3.23 Subgroup analysis based on medicine for anti-inflammatory factors**

**
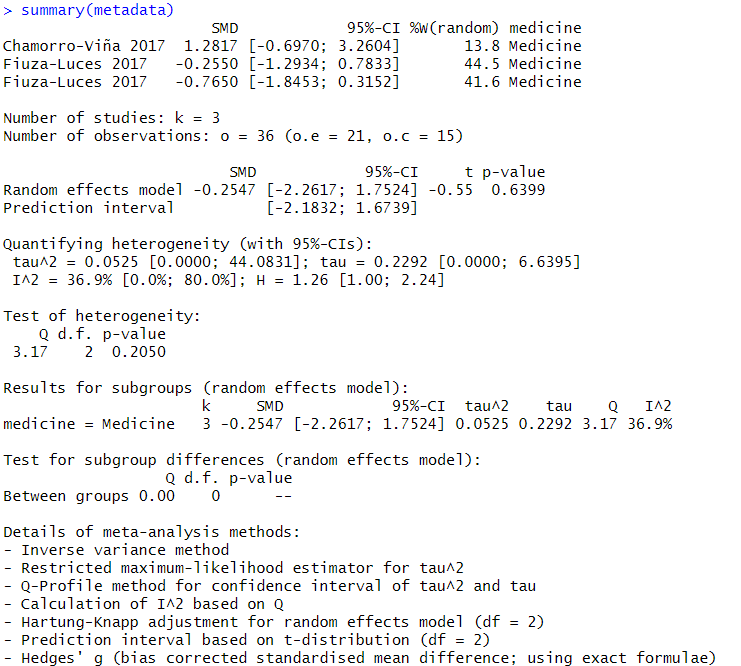
**

**
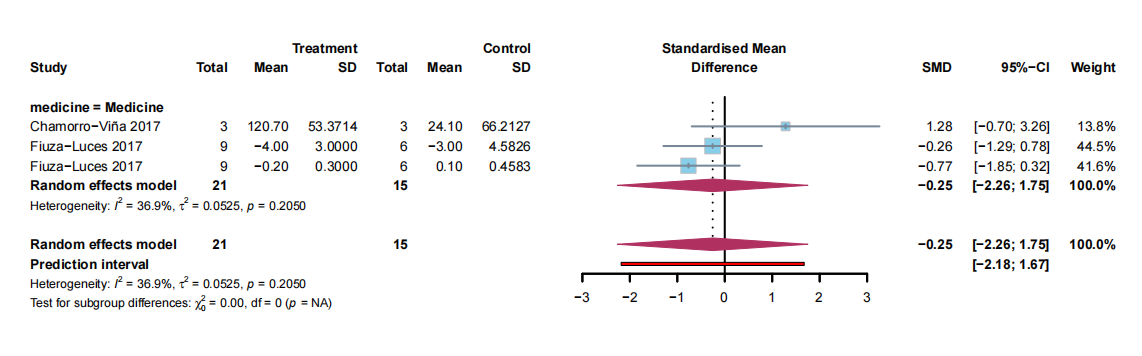
**

**13.3.24 Subgroup analysis based on medicine for cognitive function**

**
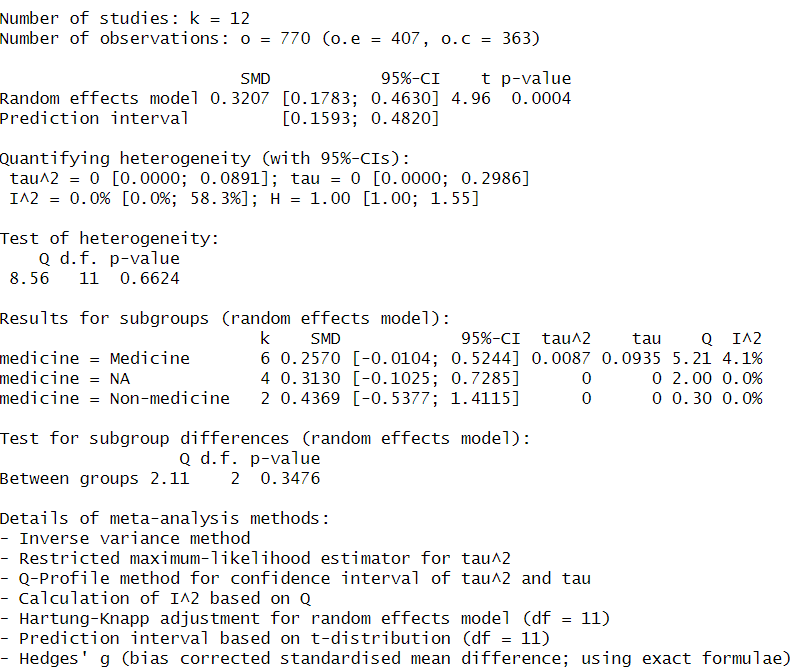
**

**
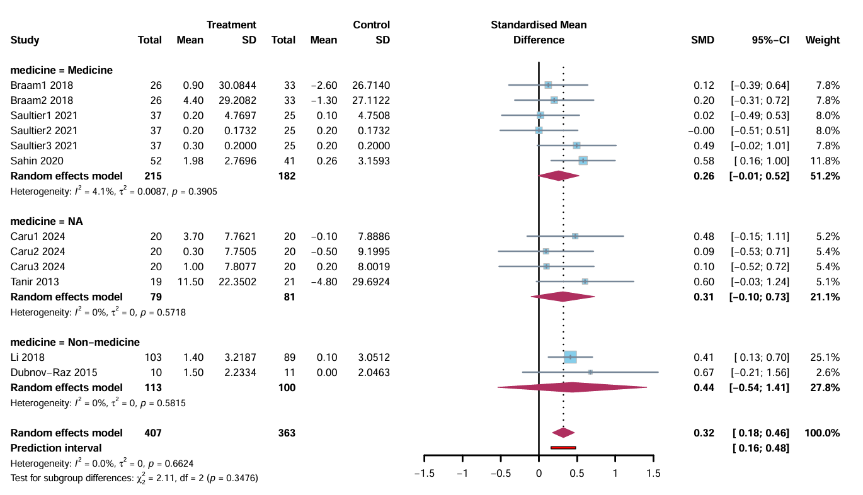
**

**13. Subgroup analysis**

**13.4 Disease types subgroup analysis**

**13.4.1 Subgroup analysis based on disease types for quality of life scale**

**
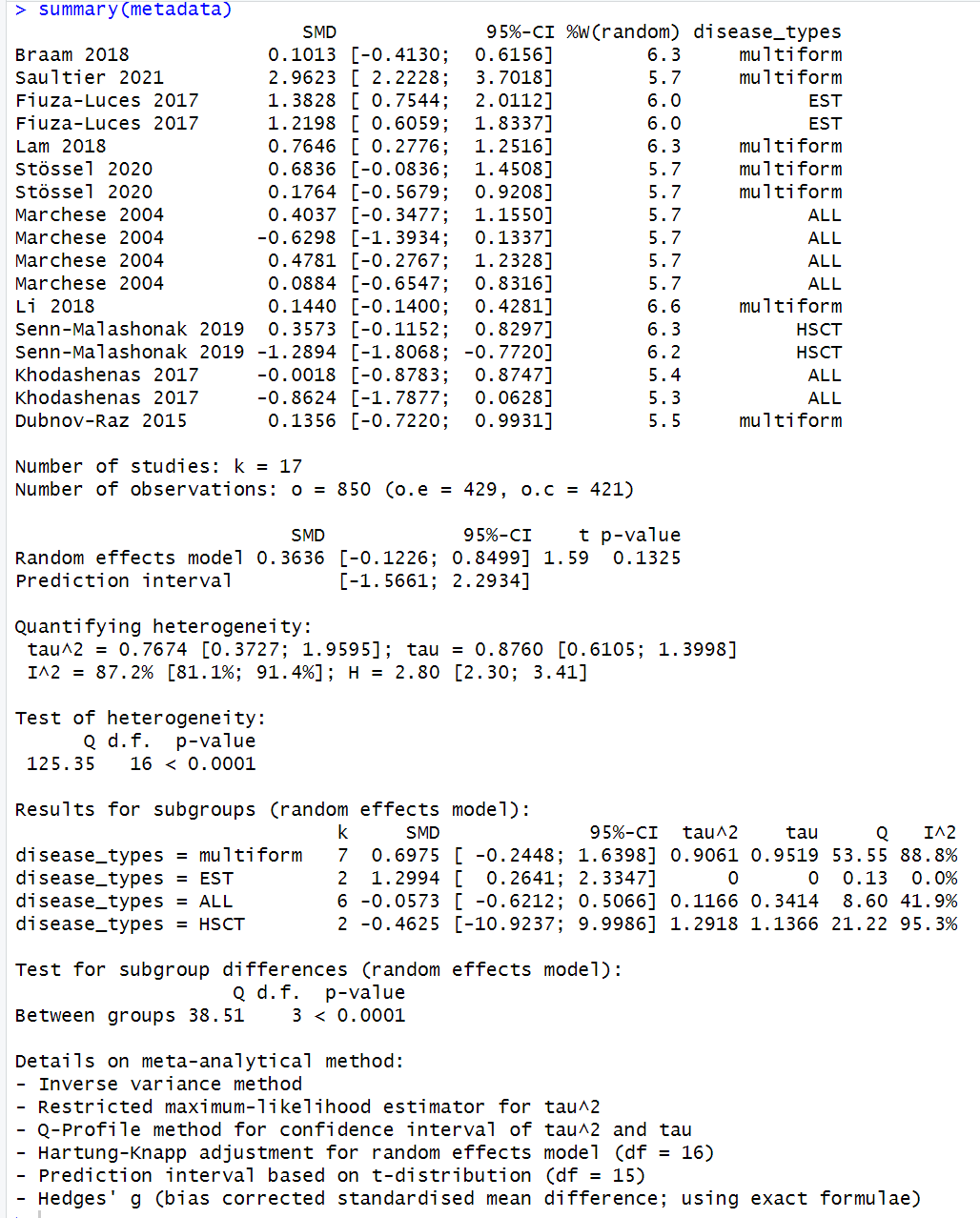
**

**
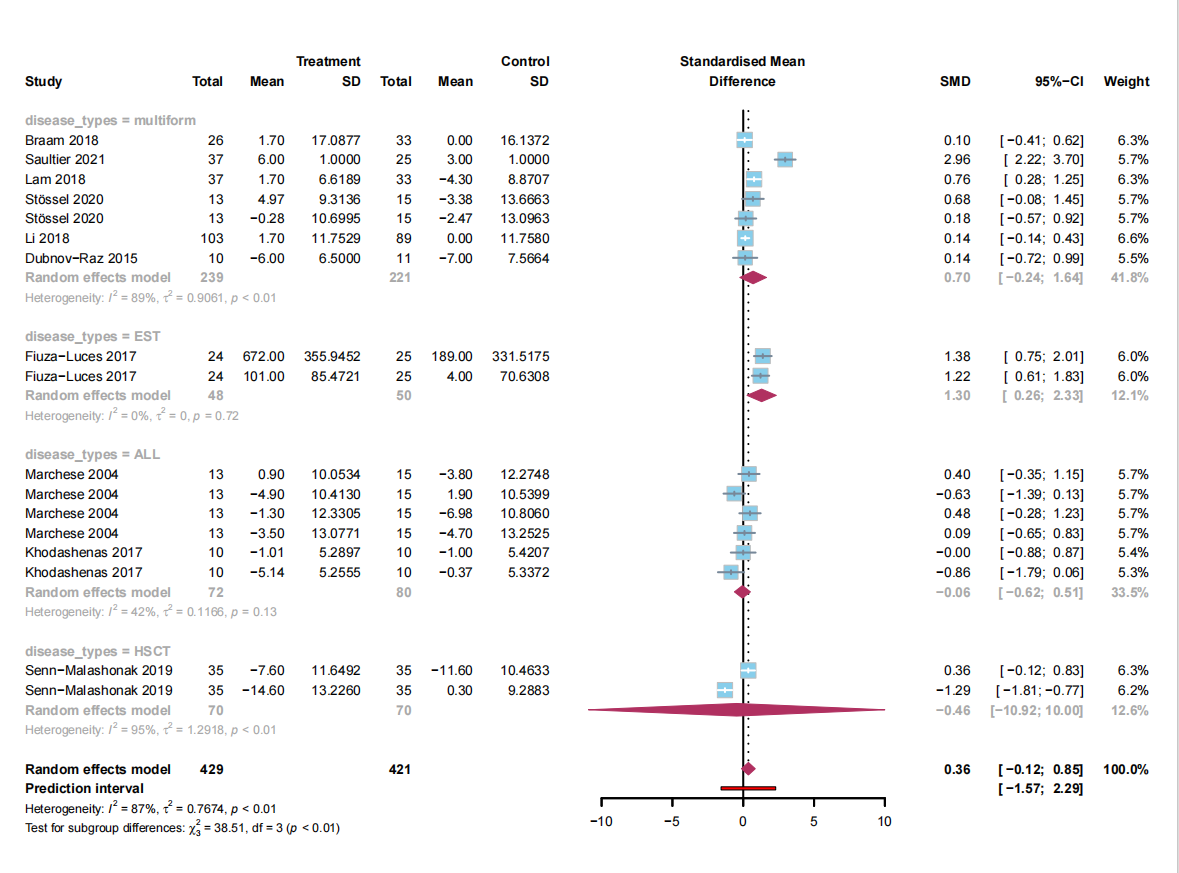
**

**13.4.2 Subgroup analysis based on disease types for fatigue**

**
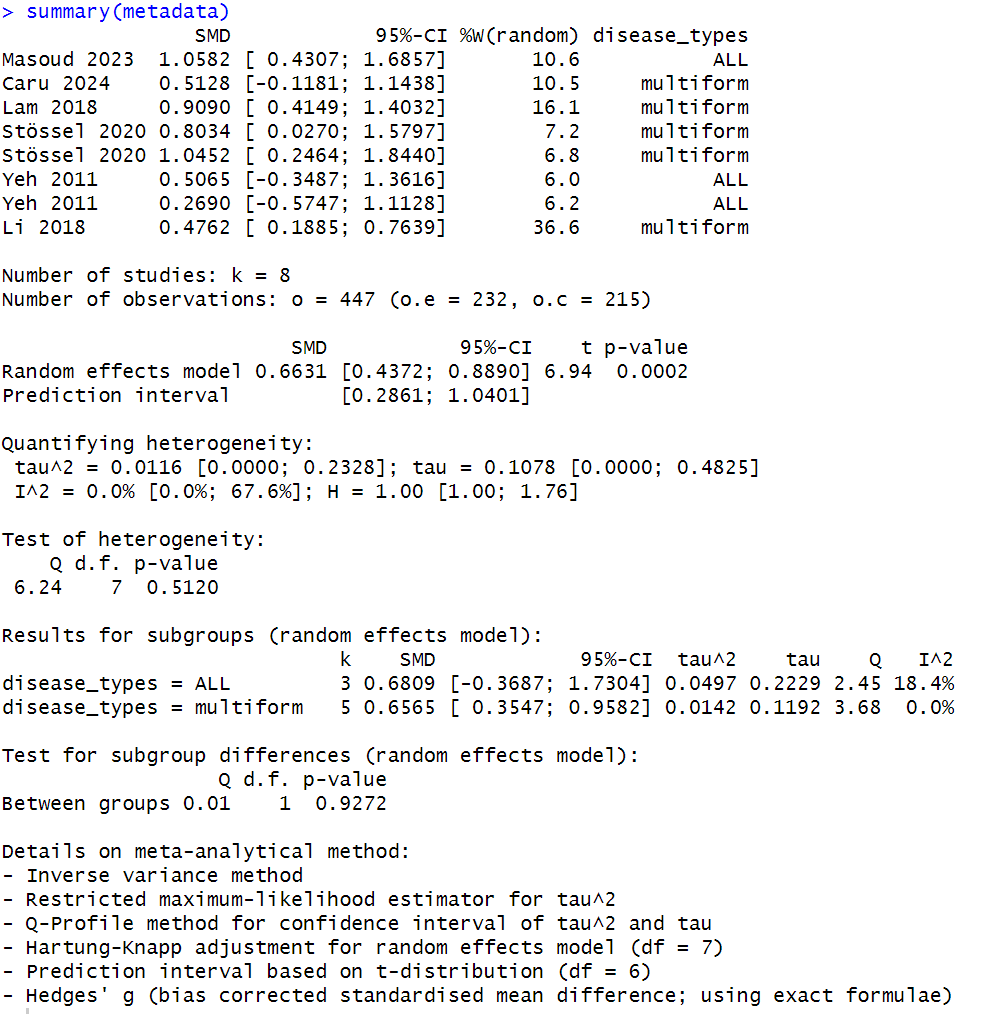
**

**
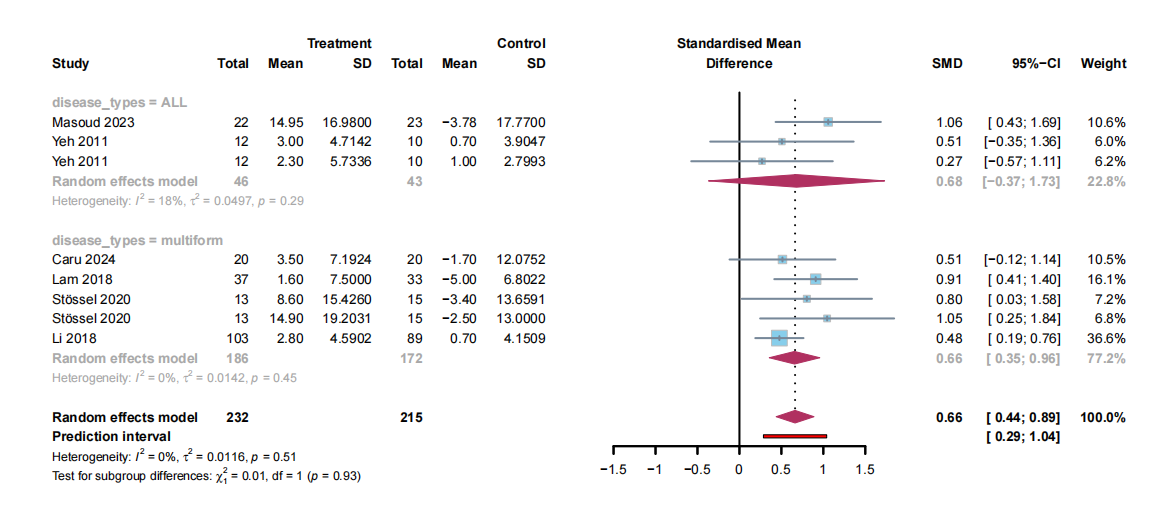
**

**13.4.3 Subgroup analysis based on disease types for quality of life**

**
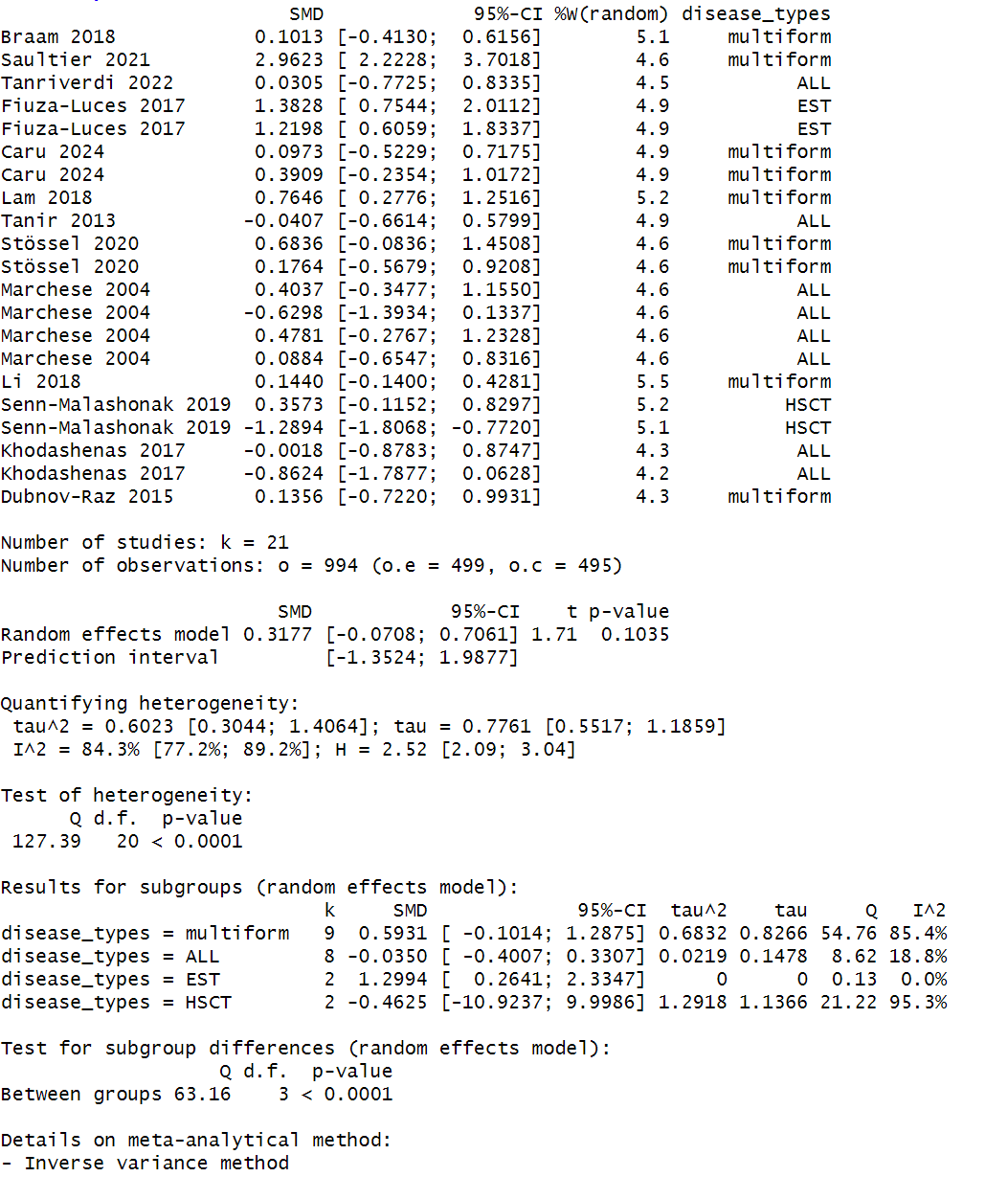
**

**
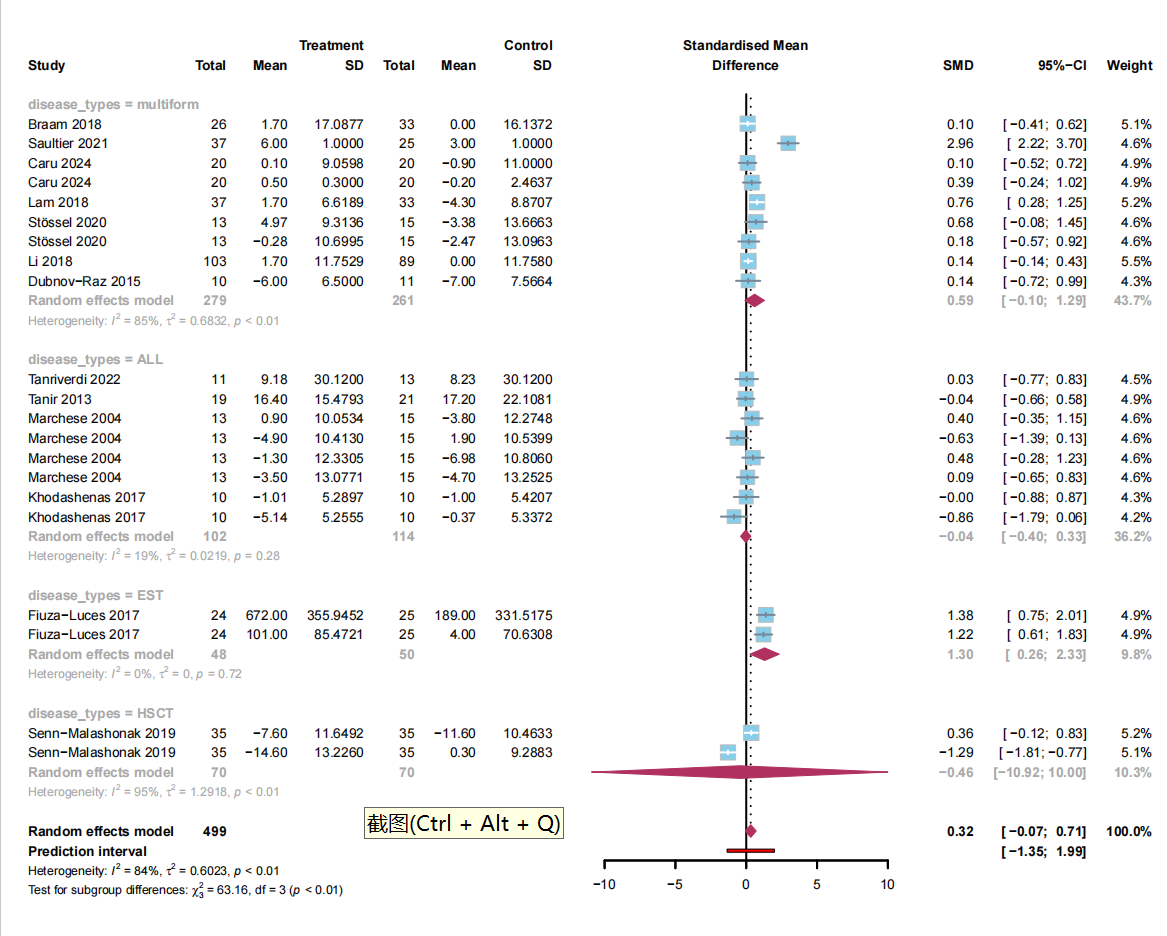
**

**13.4.4 Subgroup analysis based on disease types for lower body muscle strength**

**
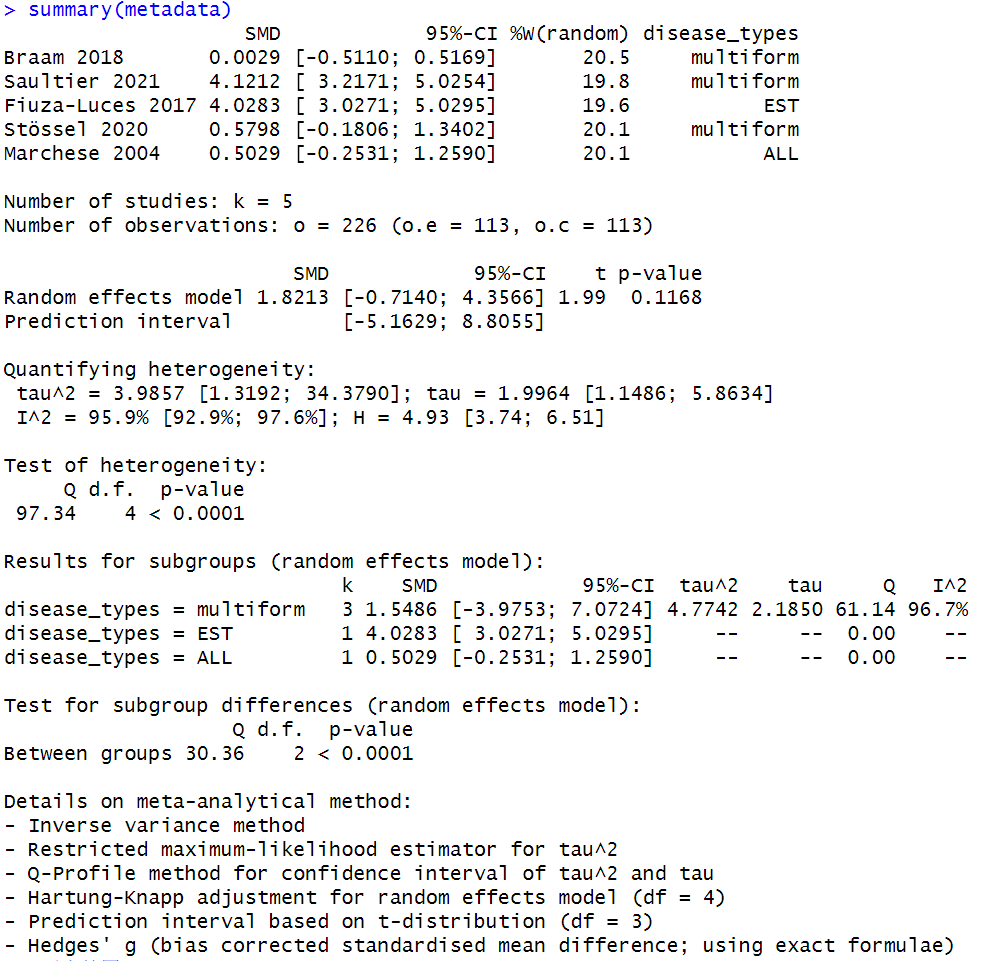
**

**
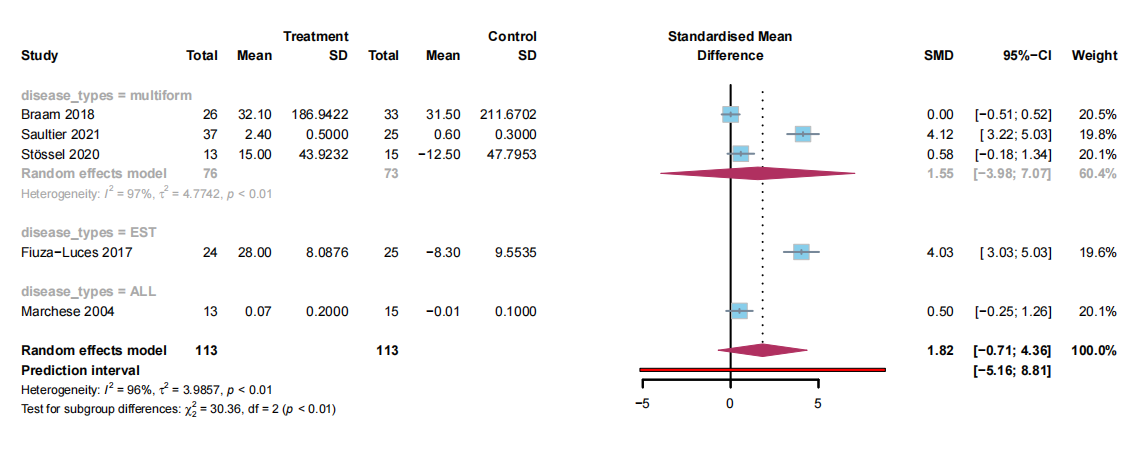
**

**13.4.5 Subgroup analysis based on disease types for upper body muscle strength**

**
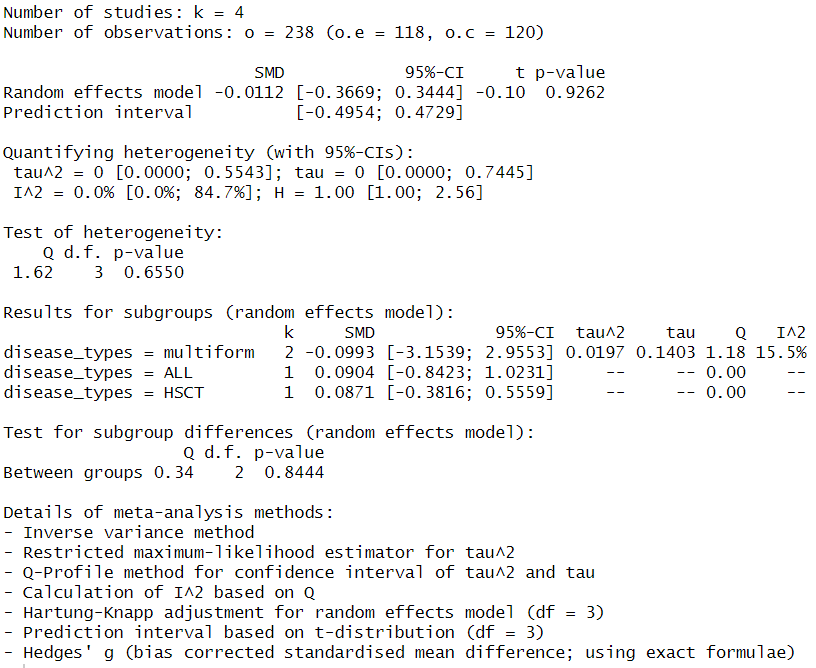
**

**
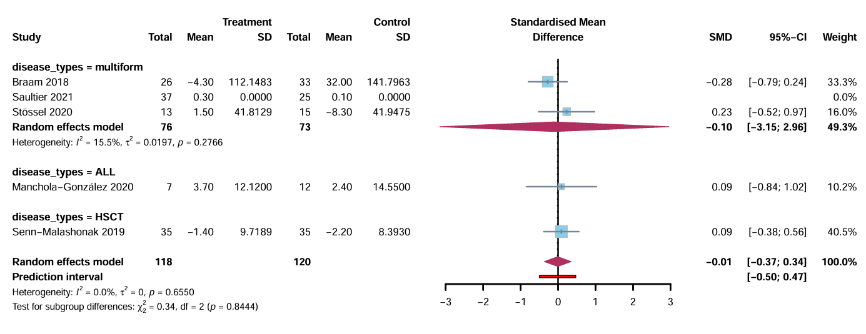
**

**13.4.6 Subgroup analysis based on disease types for trunk muscle strength**

**
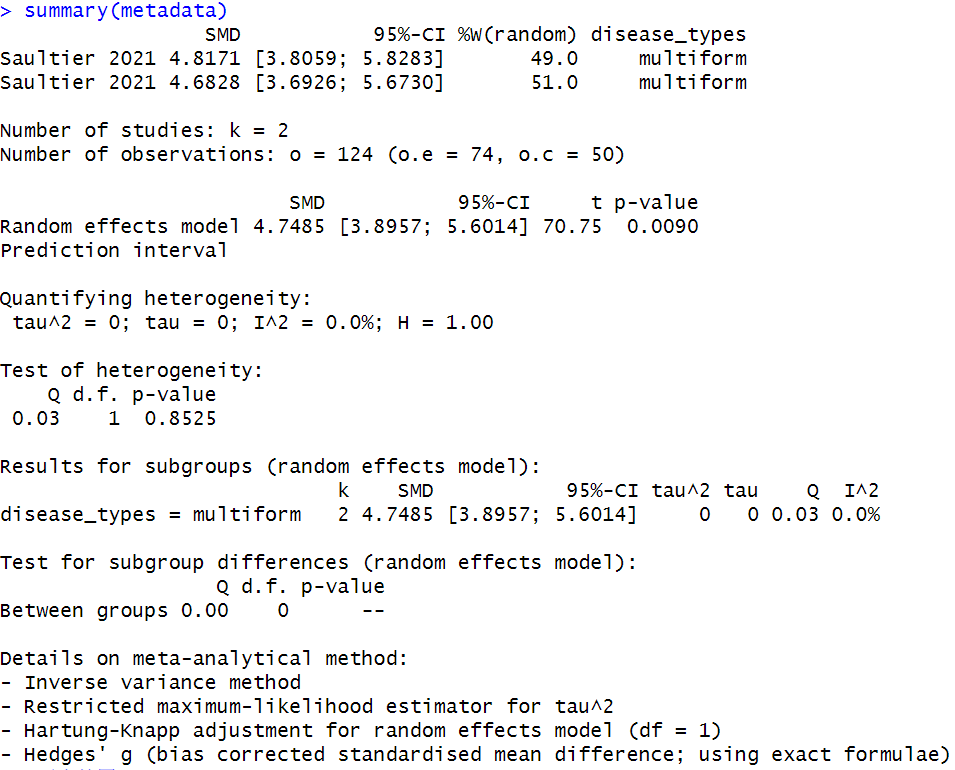
**

**
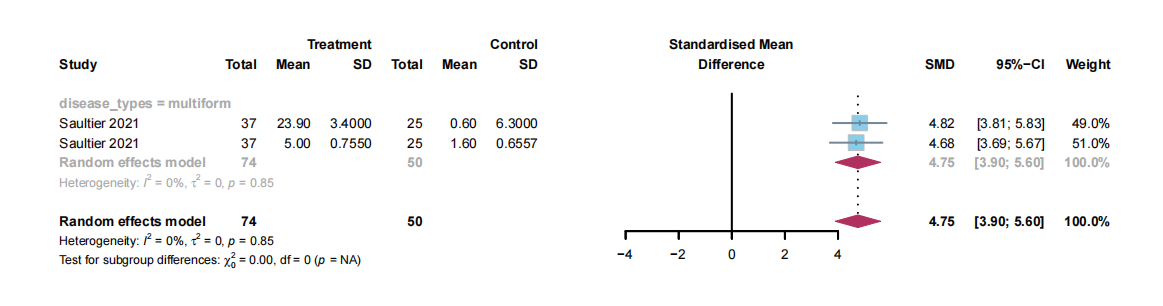
**

**13.4.7 Subgroup analysis based on disease types for muscle strength**

**
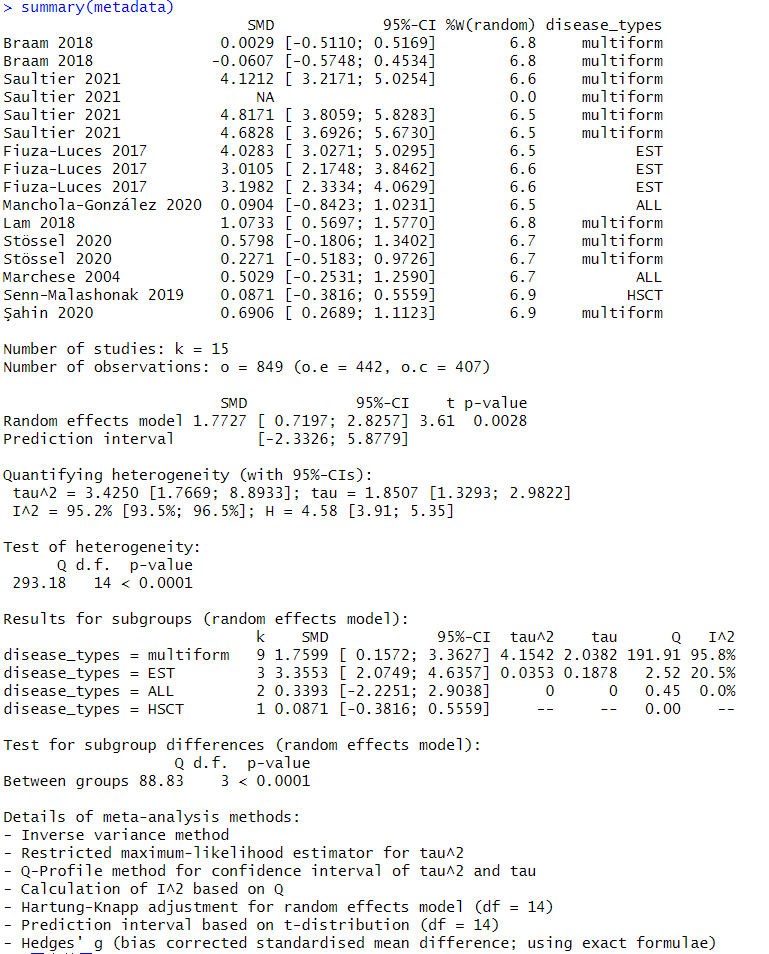
**

**
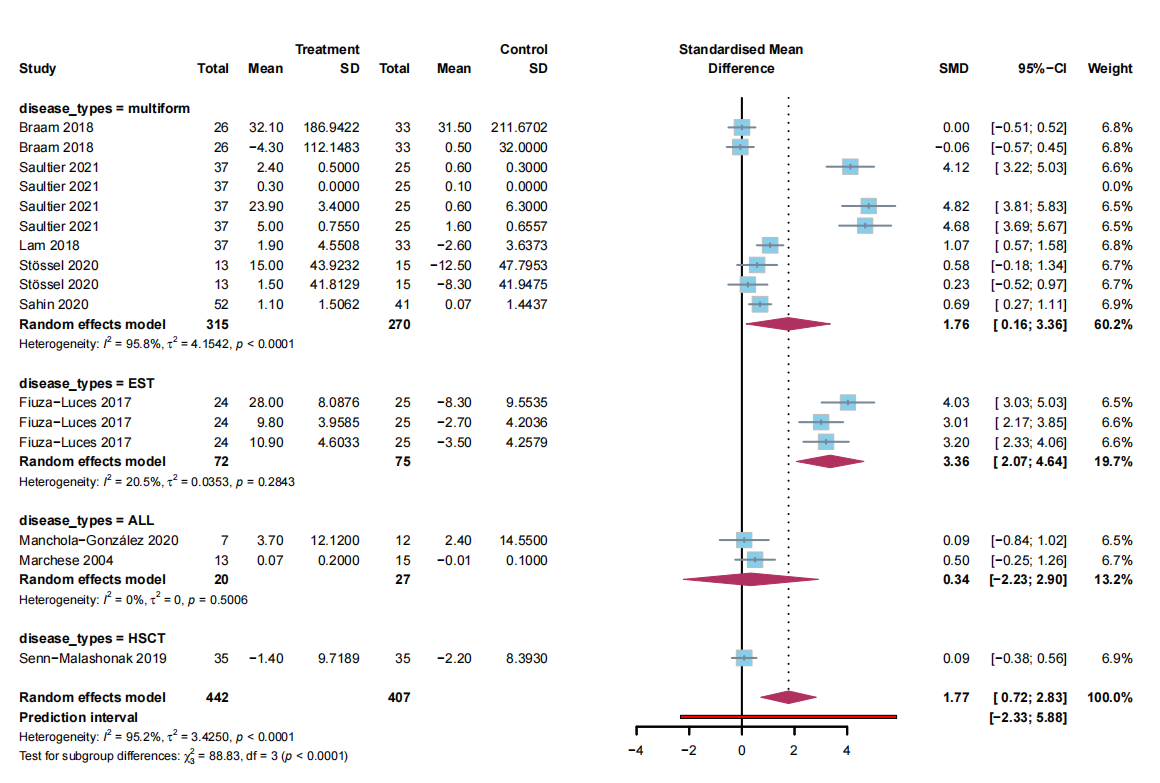
**

**13.4.8 Subgroup analysis based on disease types for six-minute walk test**

**
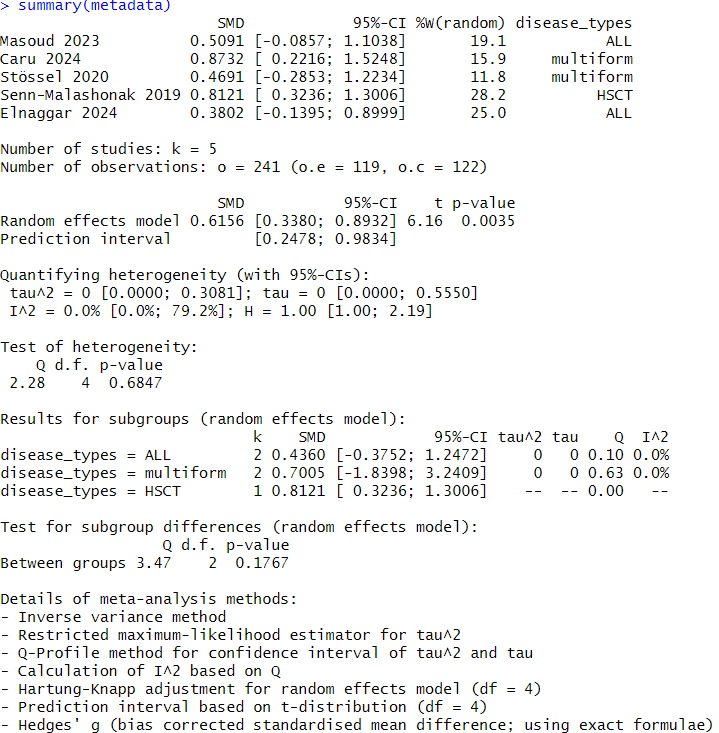
**

**
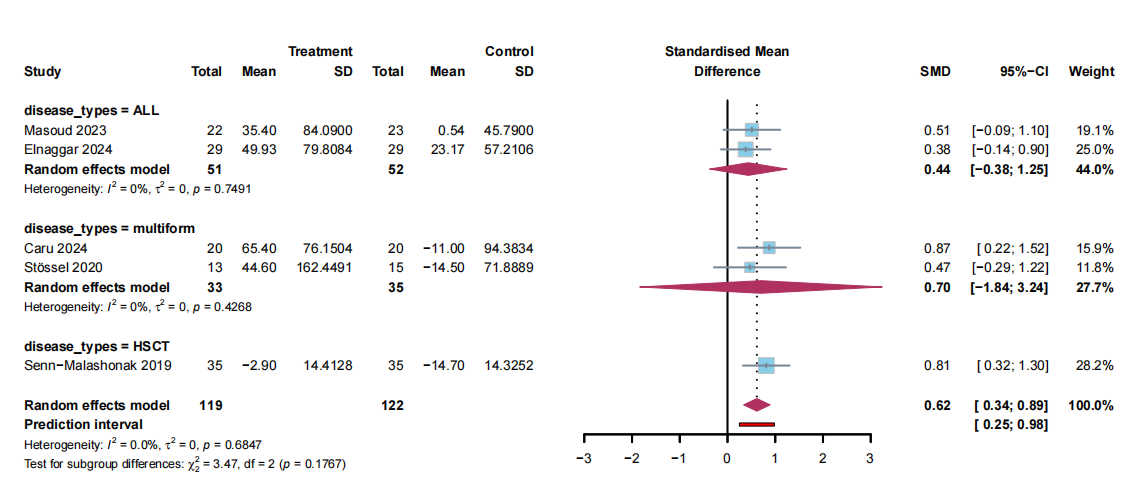
**

**13.4.9 Subgroup analysis based on disease types for balance**

**
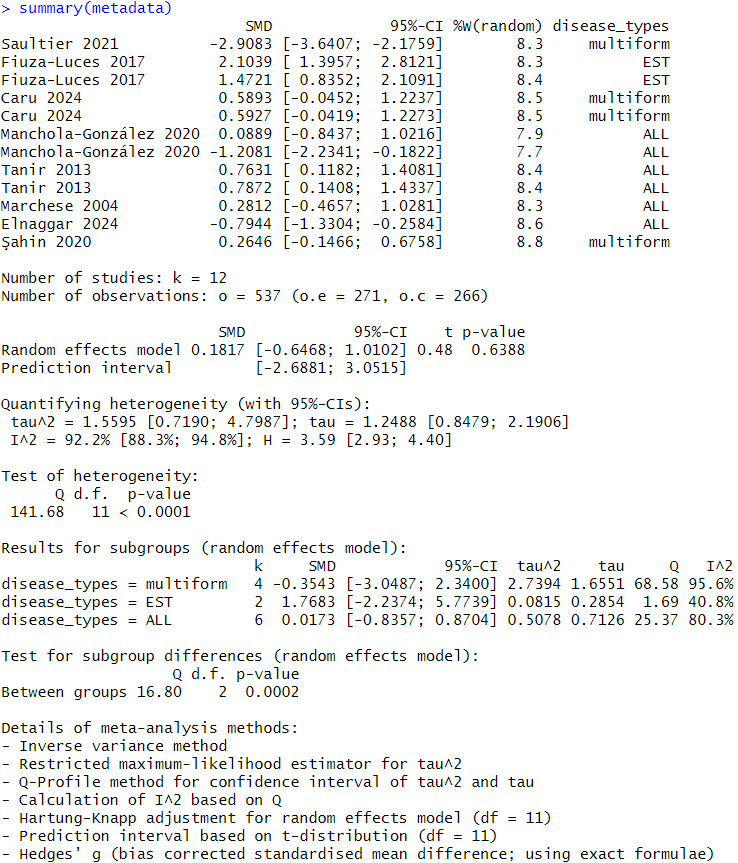
**

**
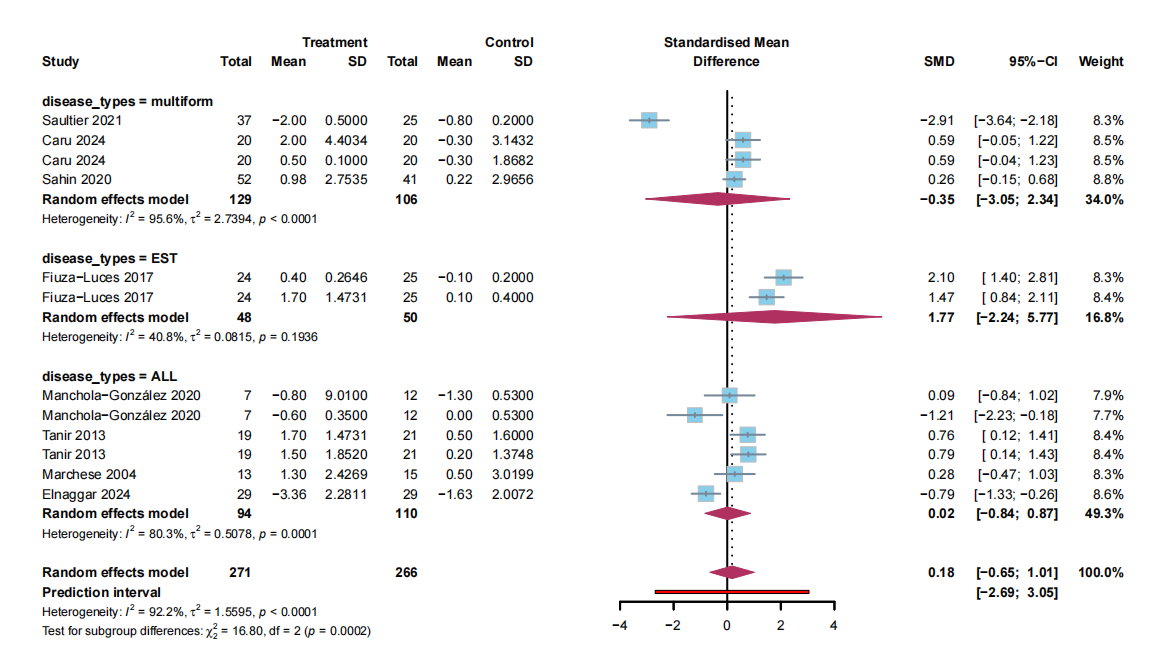
**

**13.4.10 Subgroup analysis based on disease types for flexibility**

**
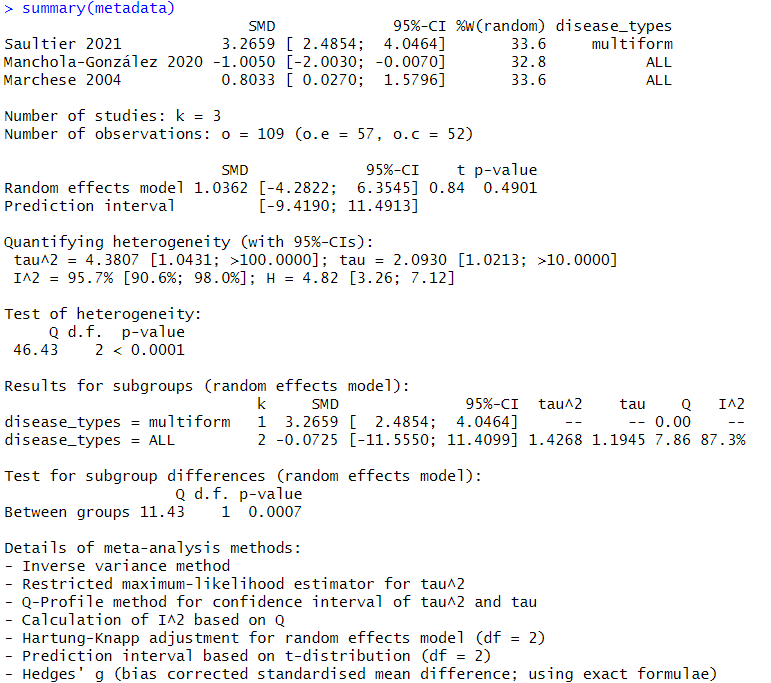
**

**
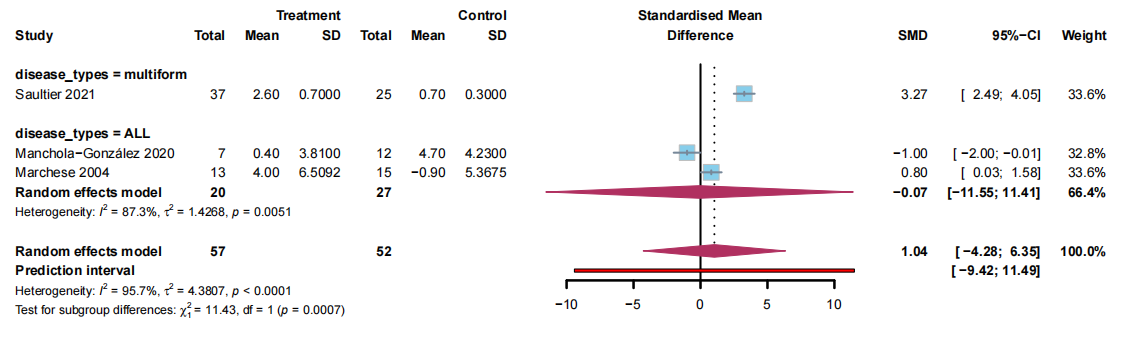
**

**13.4.11 Subgroup analysis based on disease types for athletic performance**

**
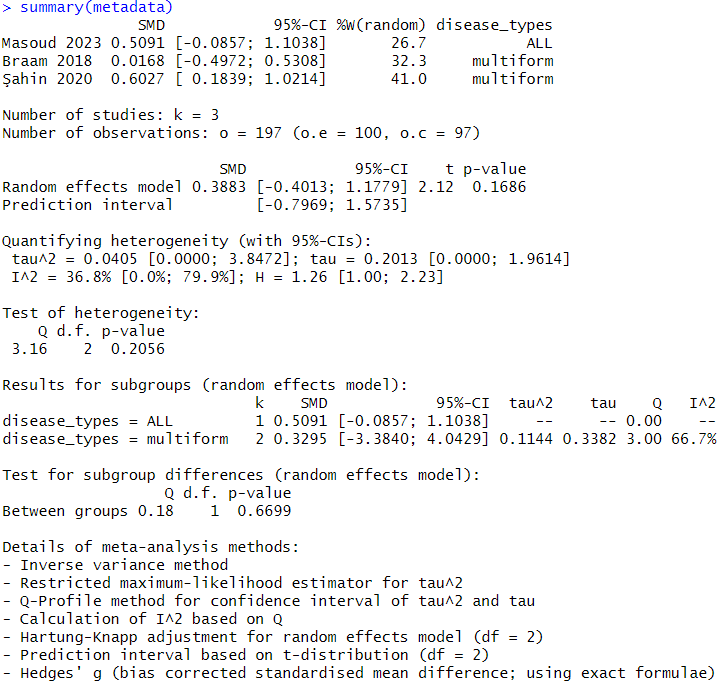
**

**
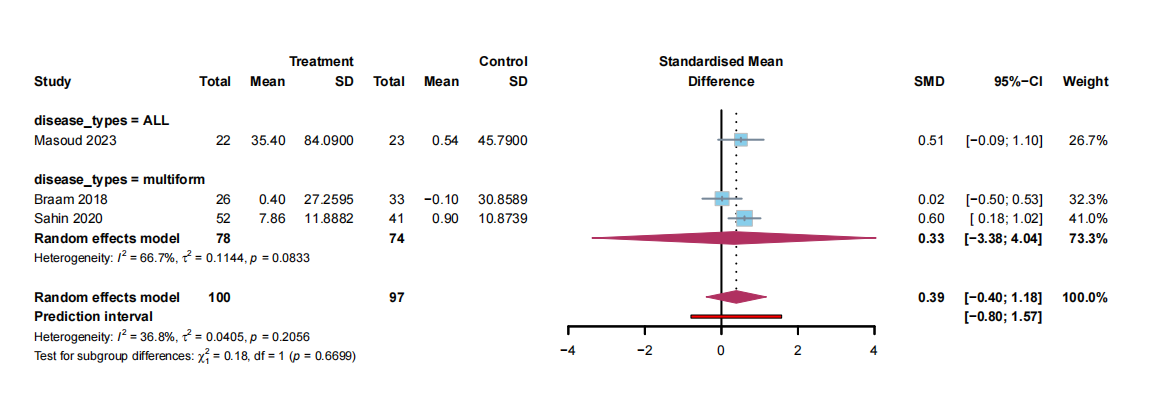
**

**13.4.12 Subgroup analysis based on disease types for physical activity level**

**
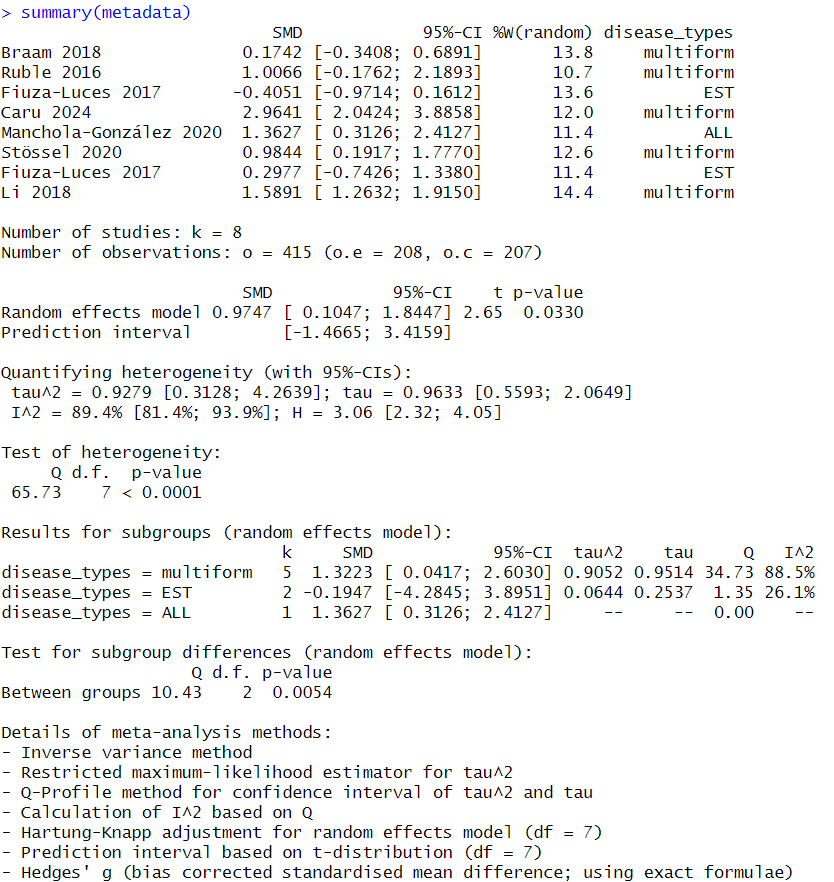
**

**
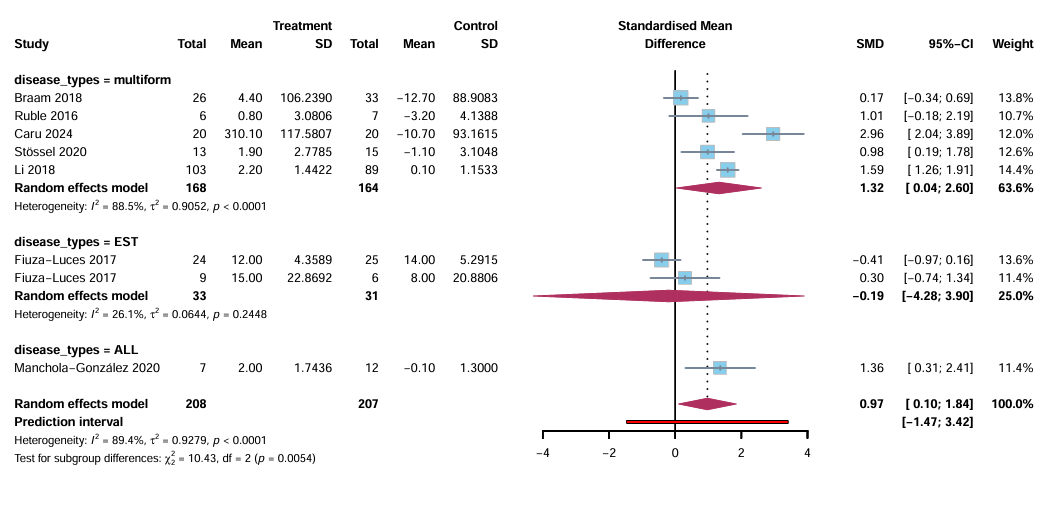
**

**13.4.13 Subgroup analysis based on disease types for physical activity behaviour**

**
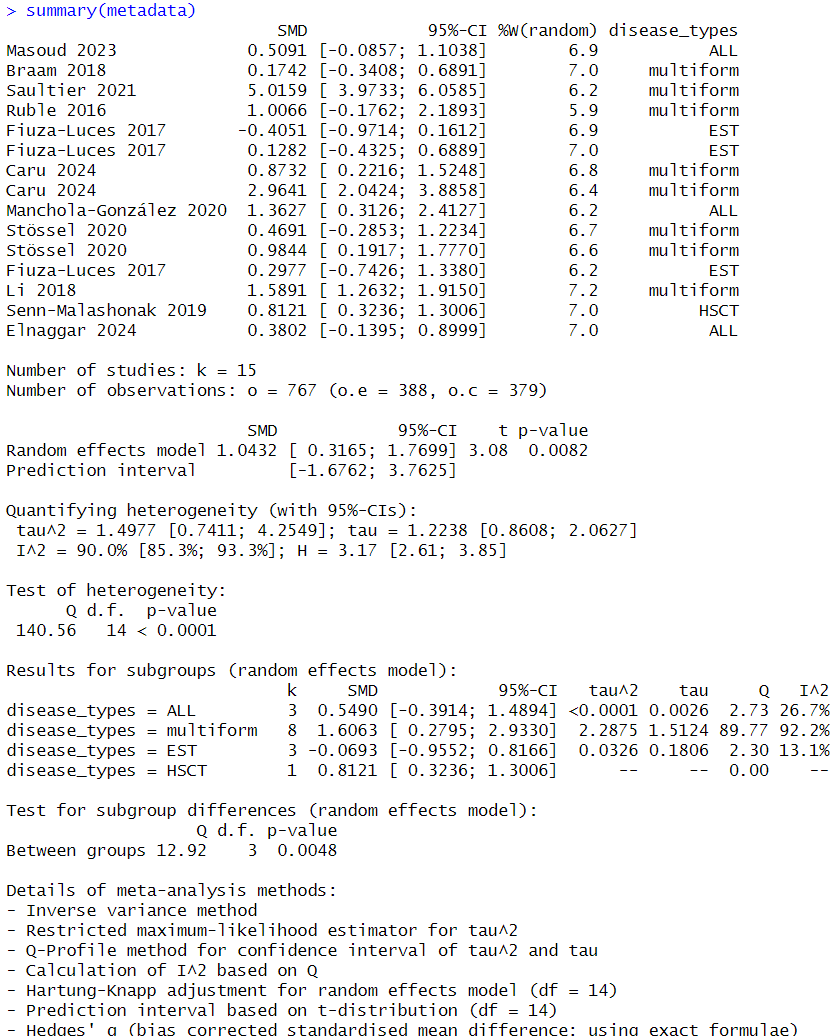
**

**
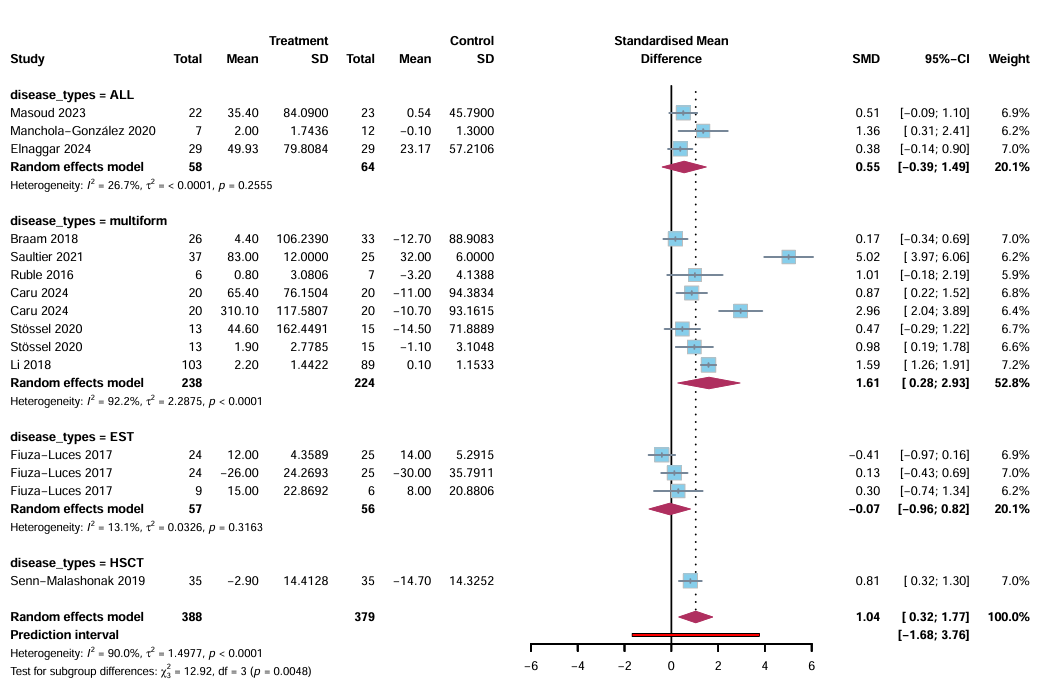
**

**13.4.14 Subgroup analysis based on disease types for peak oxygen uptake**

**
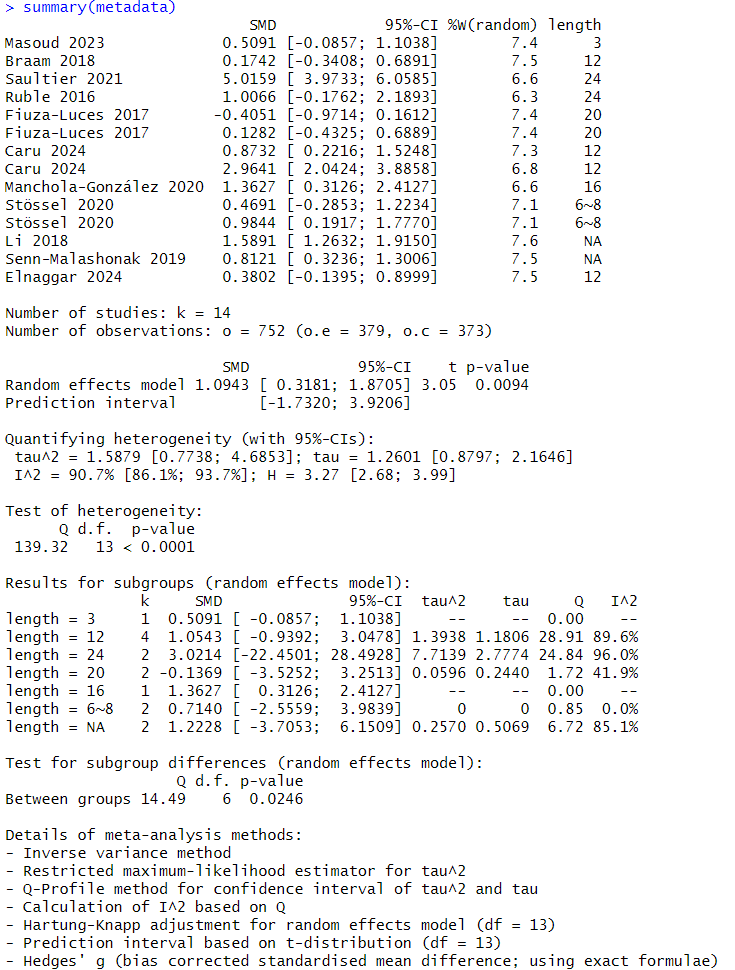
**

**
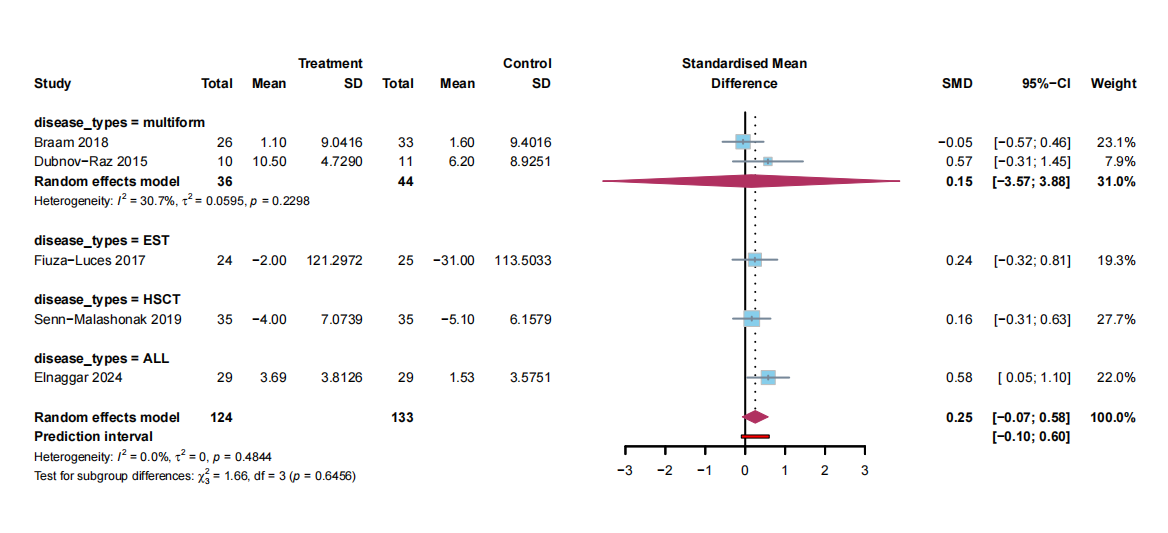
**

**13.4.15 Subgroup analysis based on disease types for cardiorespiratory function**

**
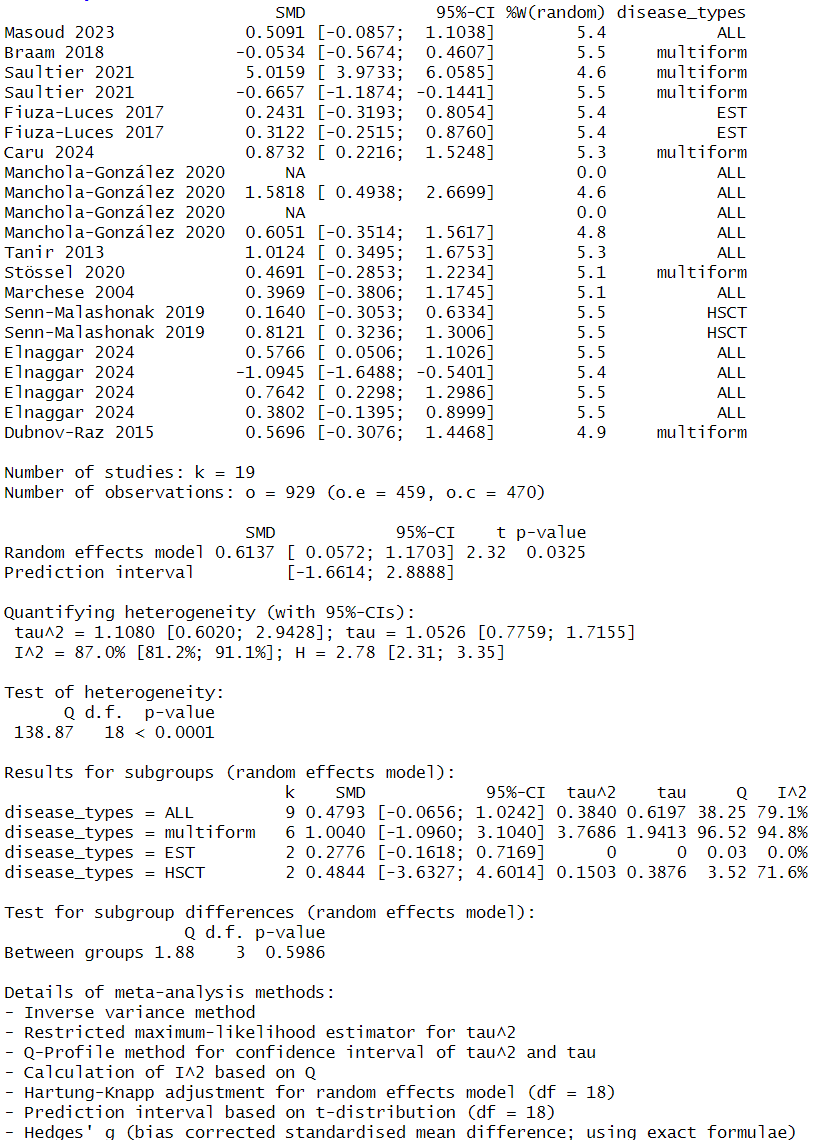
**

**
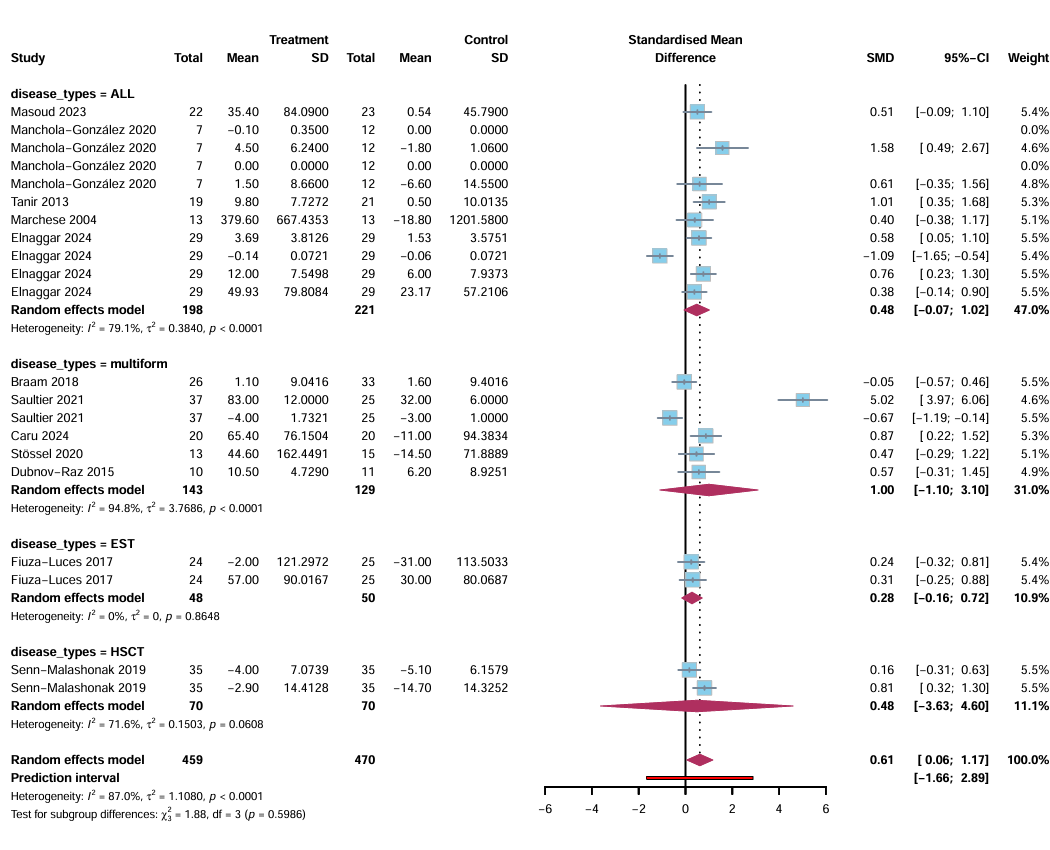
**

**13.4.16 Subgroup analysis based on disease types for bone mineral density**

**
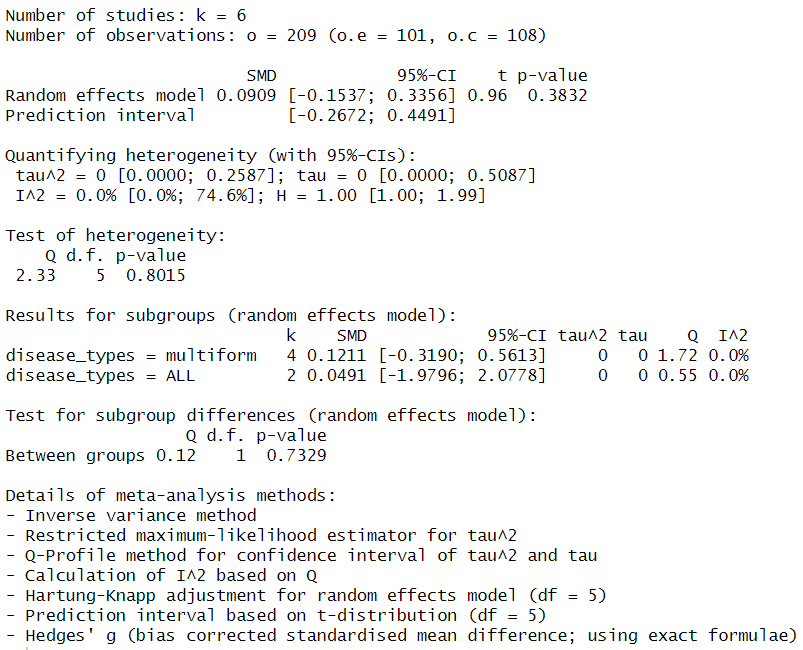
**

**
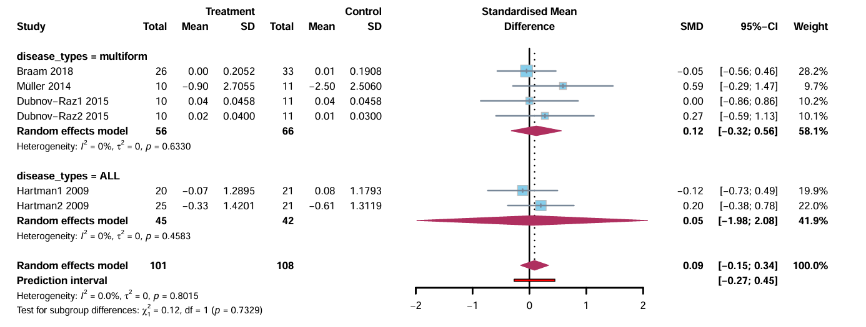
**

**13.4.17 Subgroup analysis based on disease types for body mass index**

**
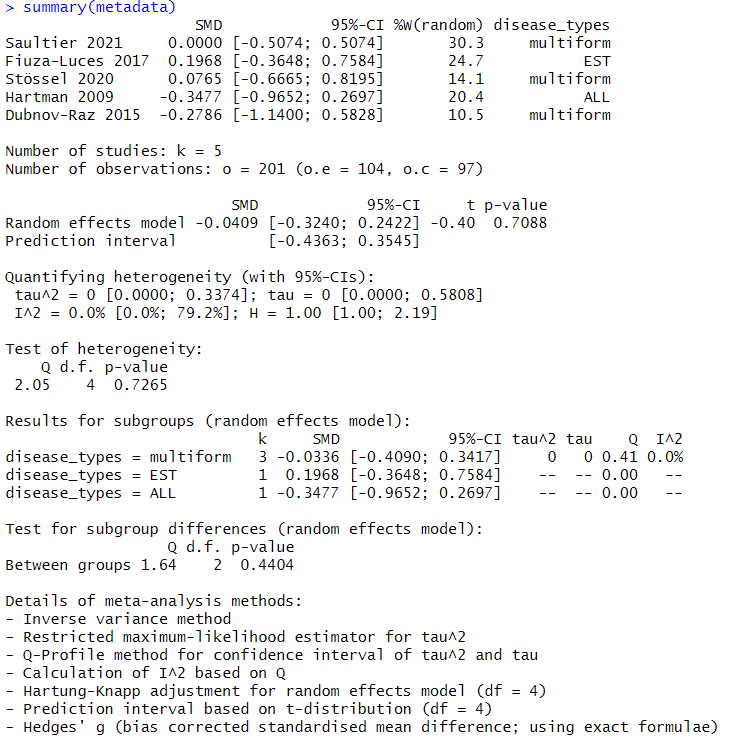
**

**
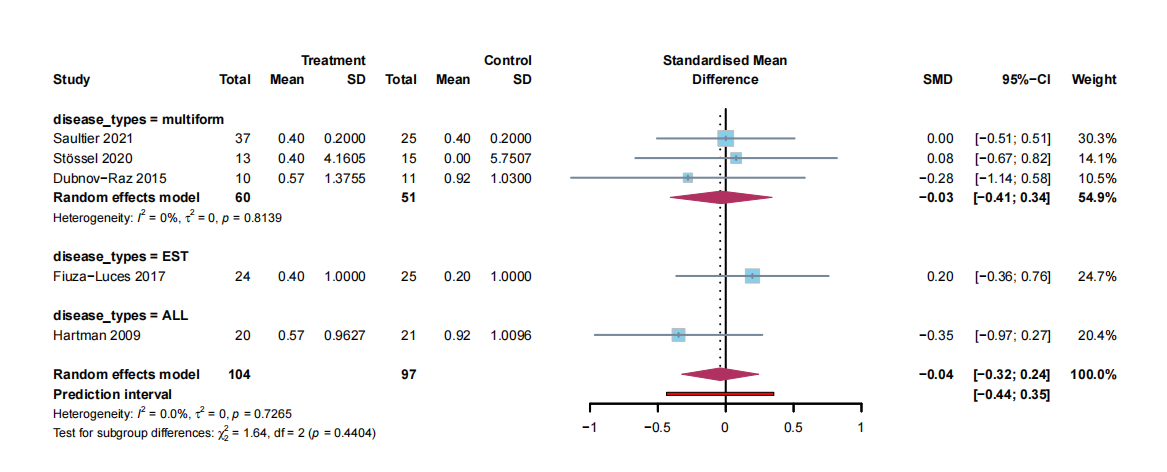
**

**13.4.18 Subgroup analysis based on disease types for fat mass percentage**

**
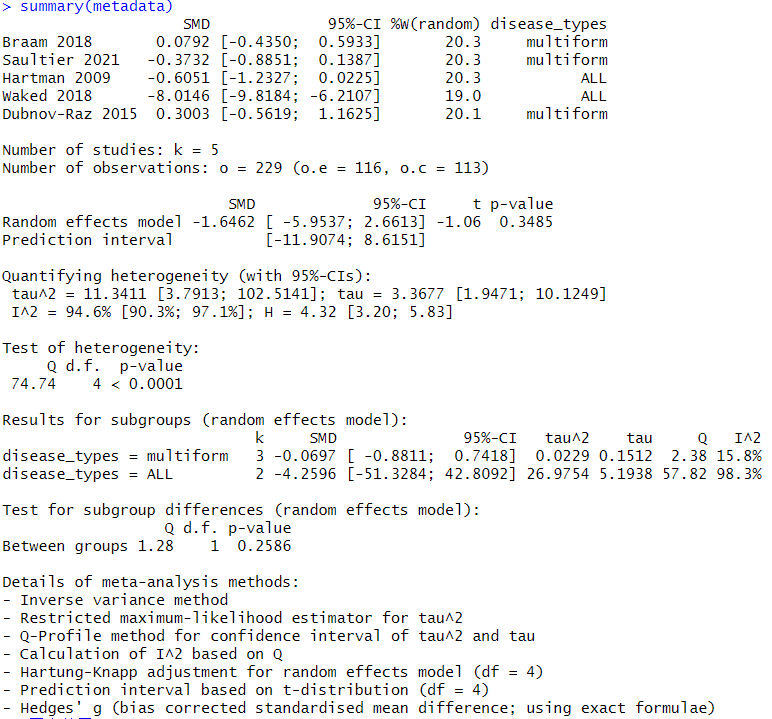
**

**
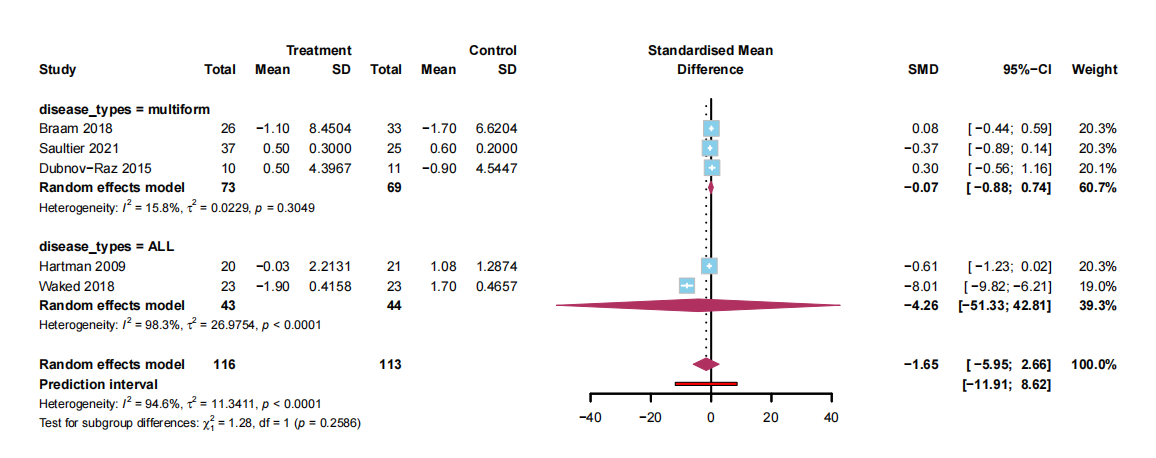
**

**13.4.19 Subgroup analysis based on disease types for NK cell level**

**
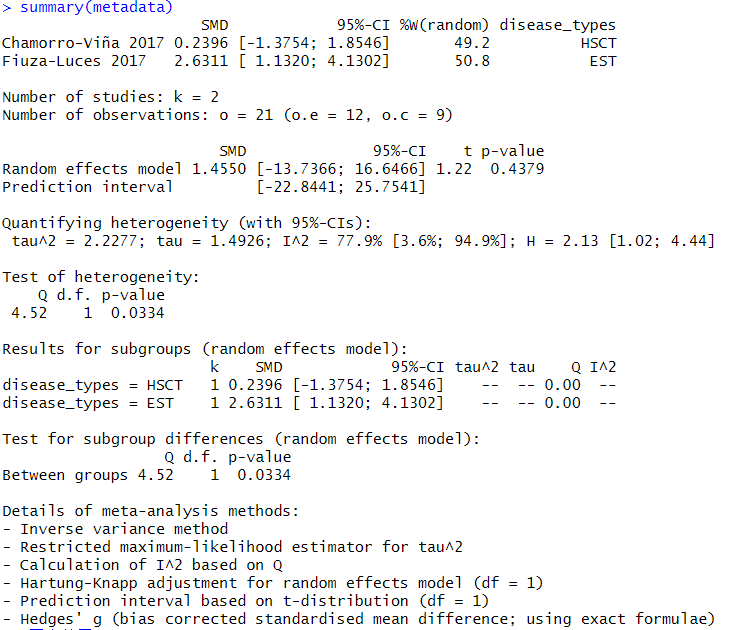
**

**
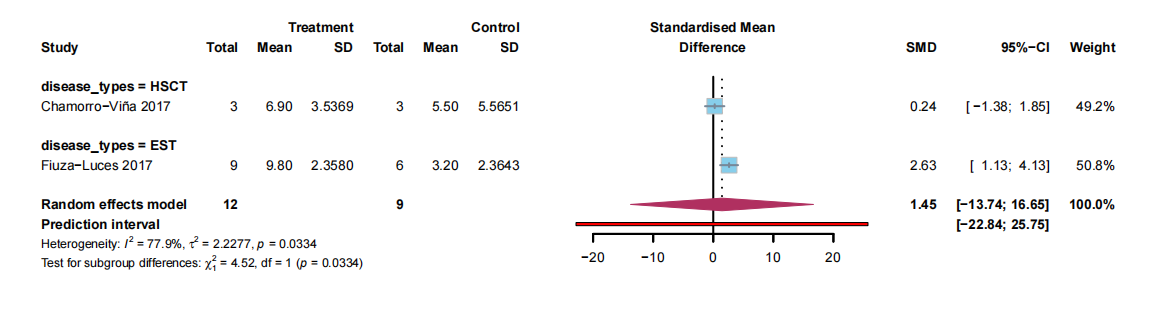
**

**13.4.20 Subgroup analysis based on disease types for depressive symptoms**

**
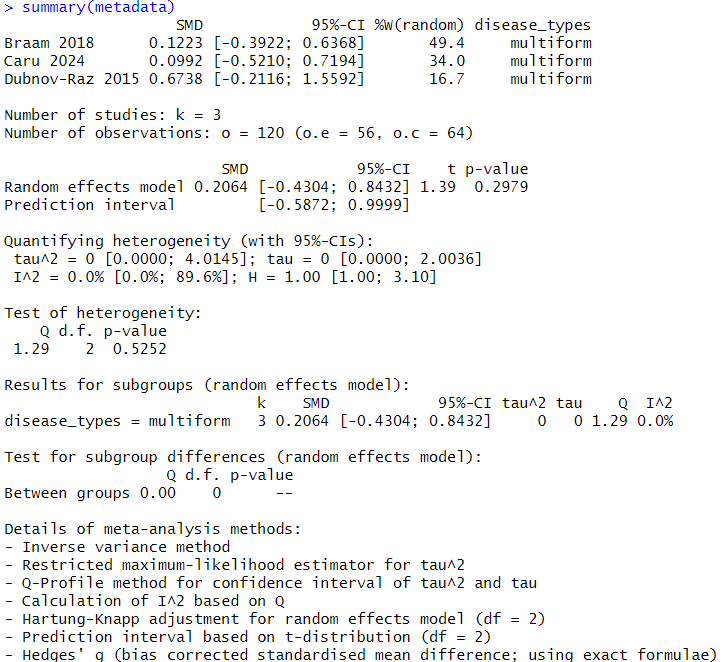
**

**
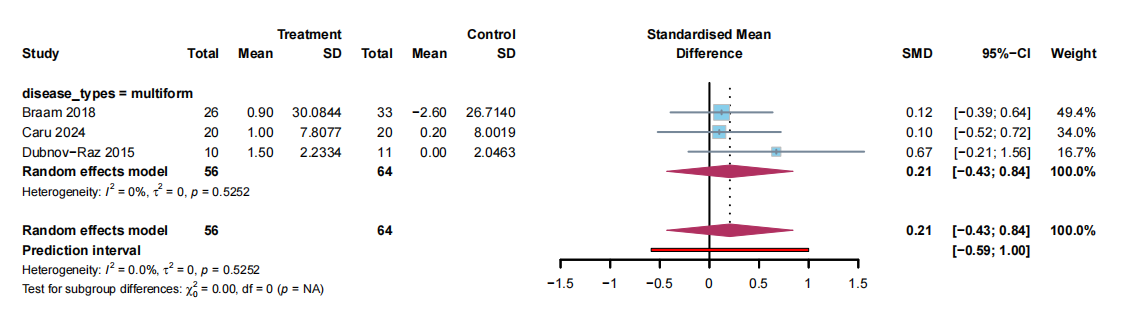
**

**13.4.21 Subgroup analysis based on disease types for social function**

**
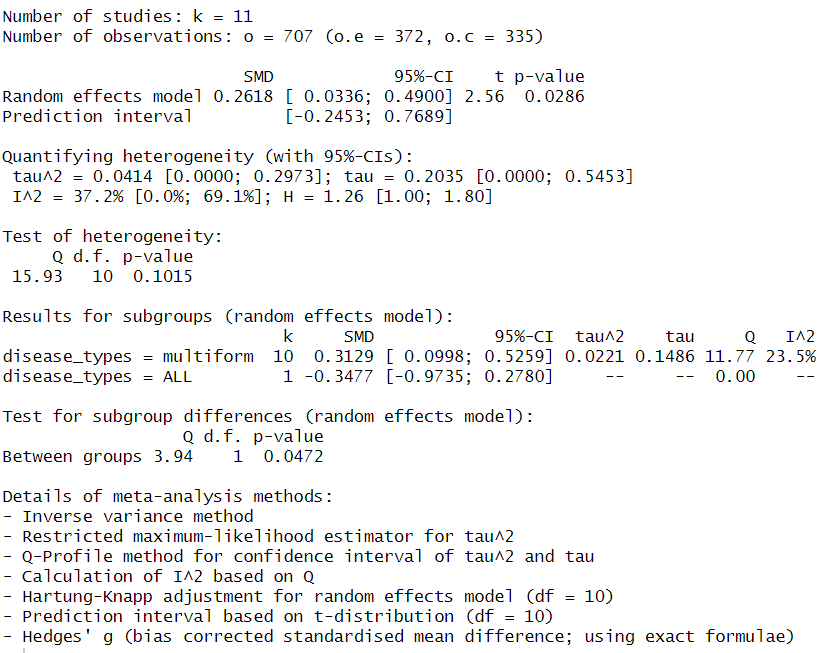
**

**
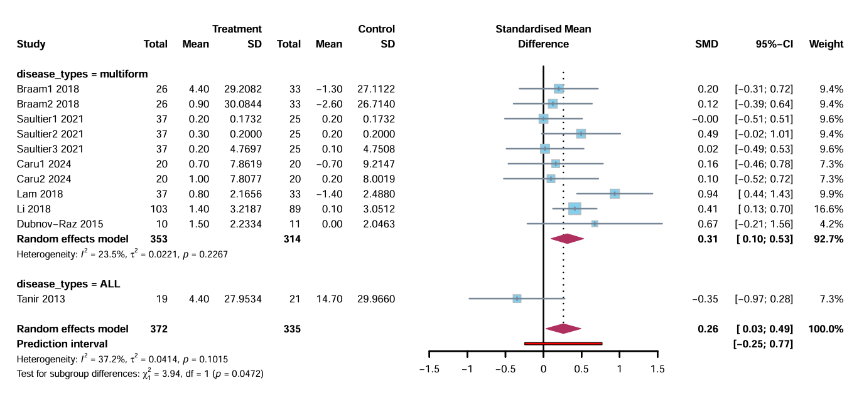
**

**13.4.22 Subgroup analysis based on disease types for executive function**

**
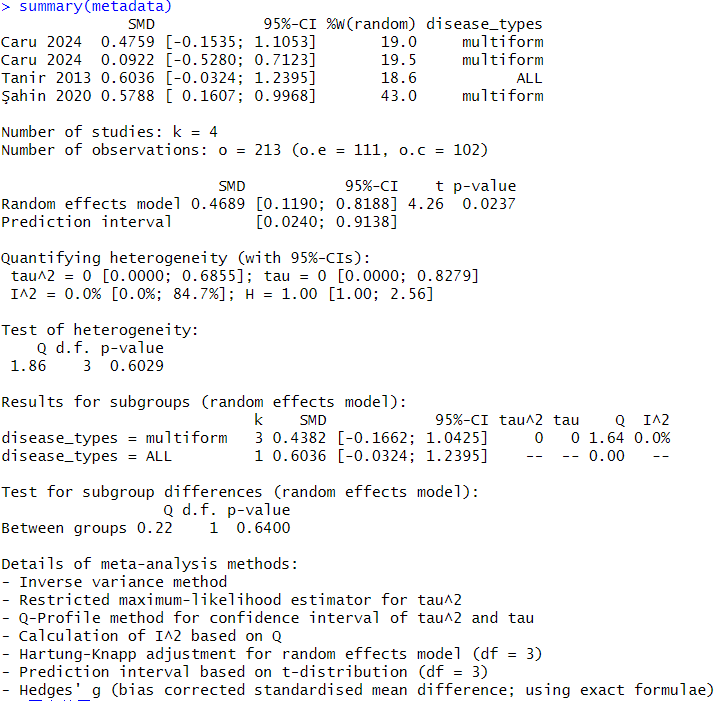
**

**
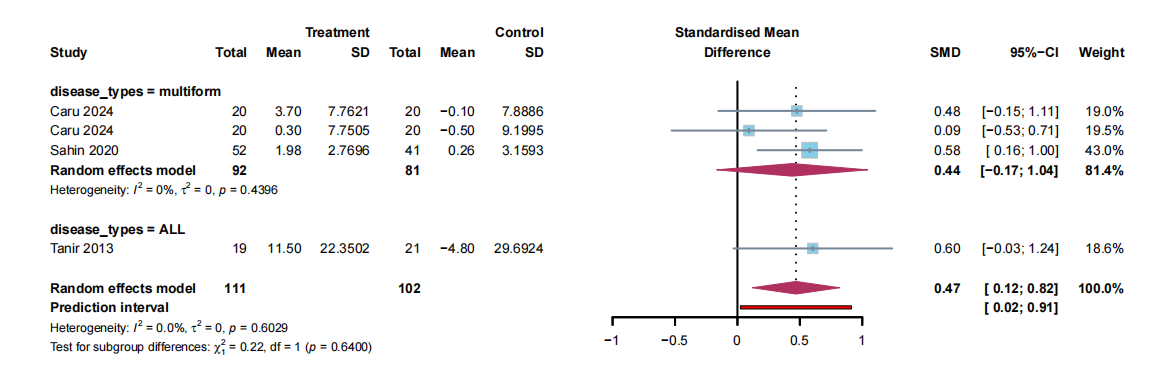
**

**13.4.23 Subgroup analysis based on disease types for pro-inflammatory factors**

**
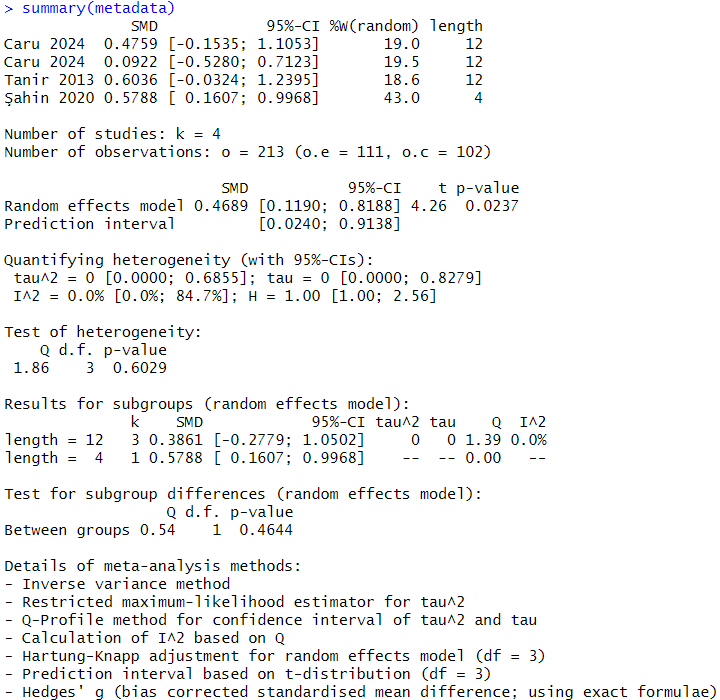
**

**
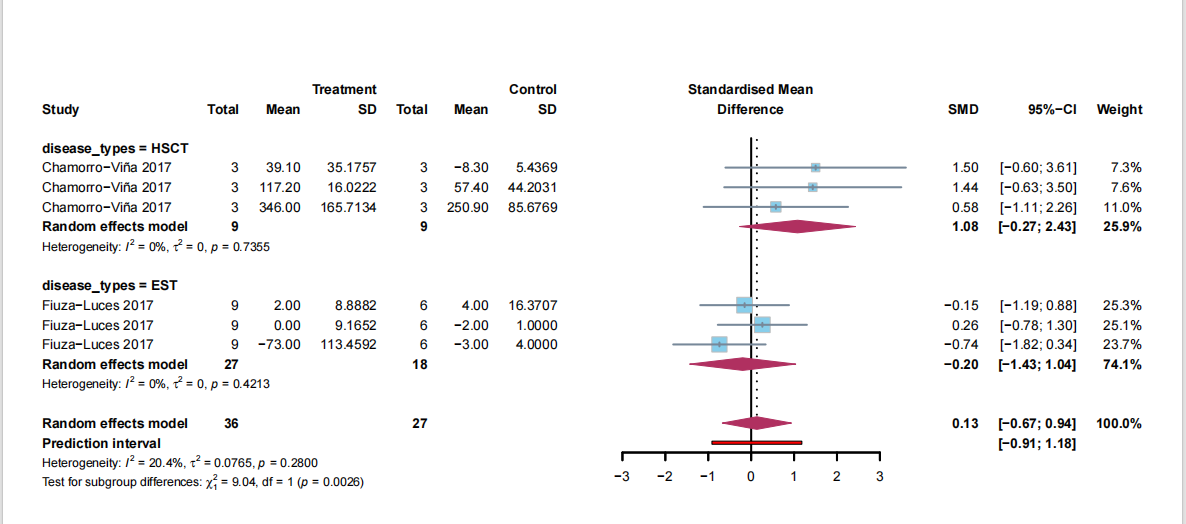
**

**13.4.24 Subgroup analysis based on disease types for anti-inflammatory factors**

**
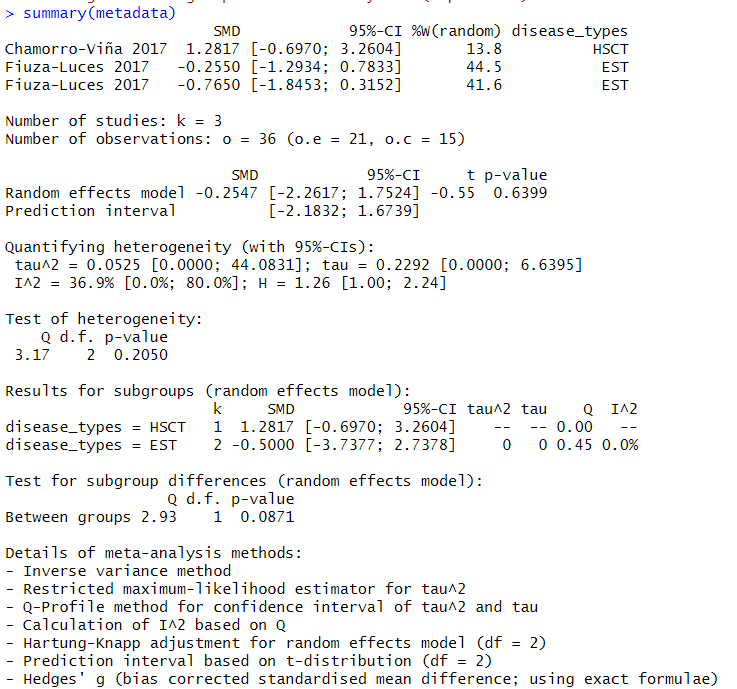
**

**
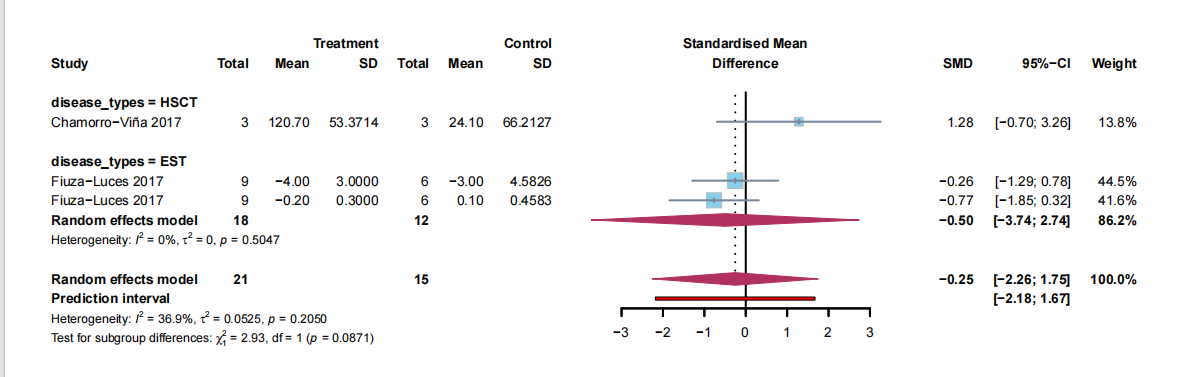
**

**13.4.25 Subgroup analysis based on disease types for cognitive function**

**
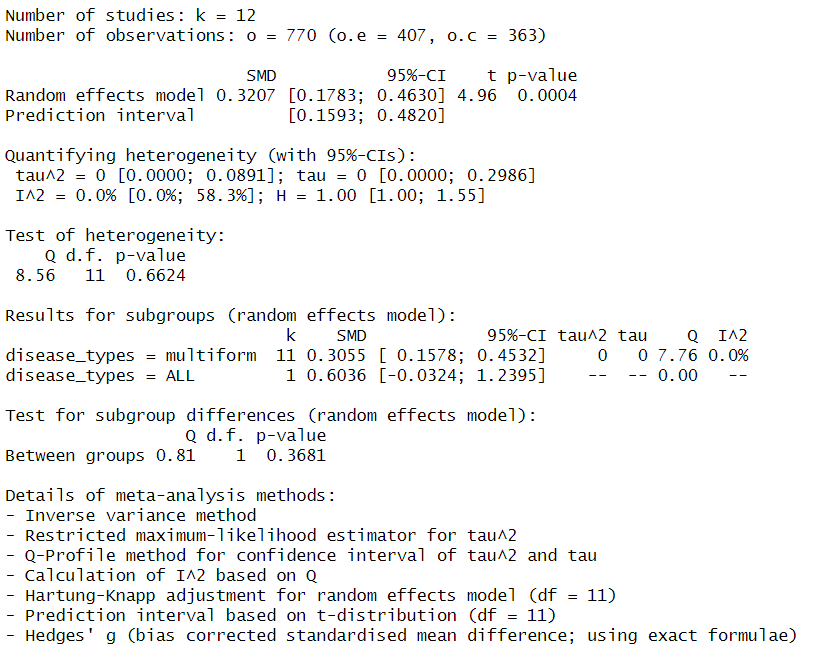
**

**
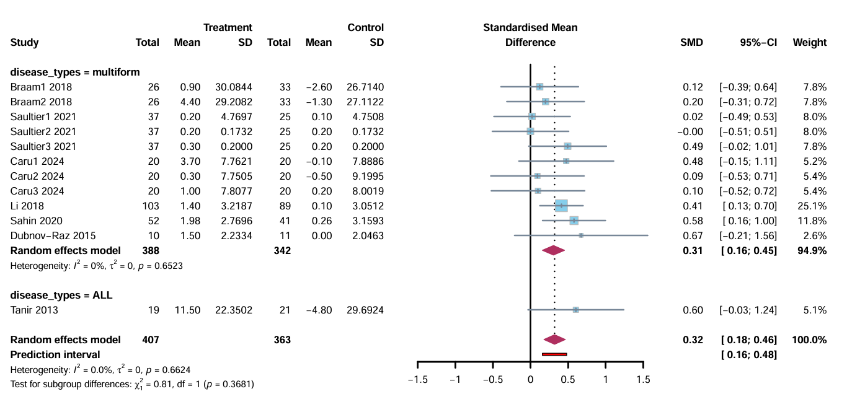
**

**13.5 Treatment stage subgroup analysis**

**13.5.1 Subgroup analysis based on treatment stage for quality of life scale**

**
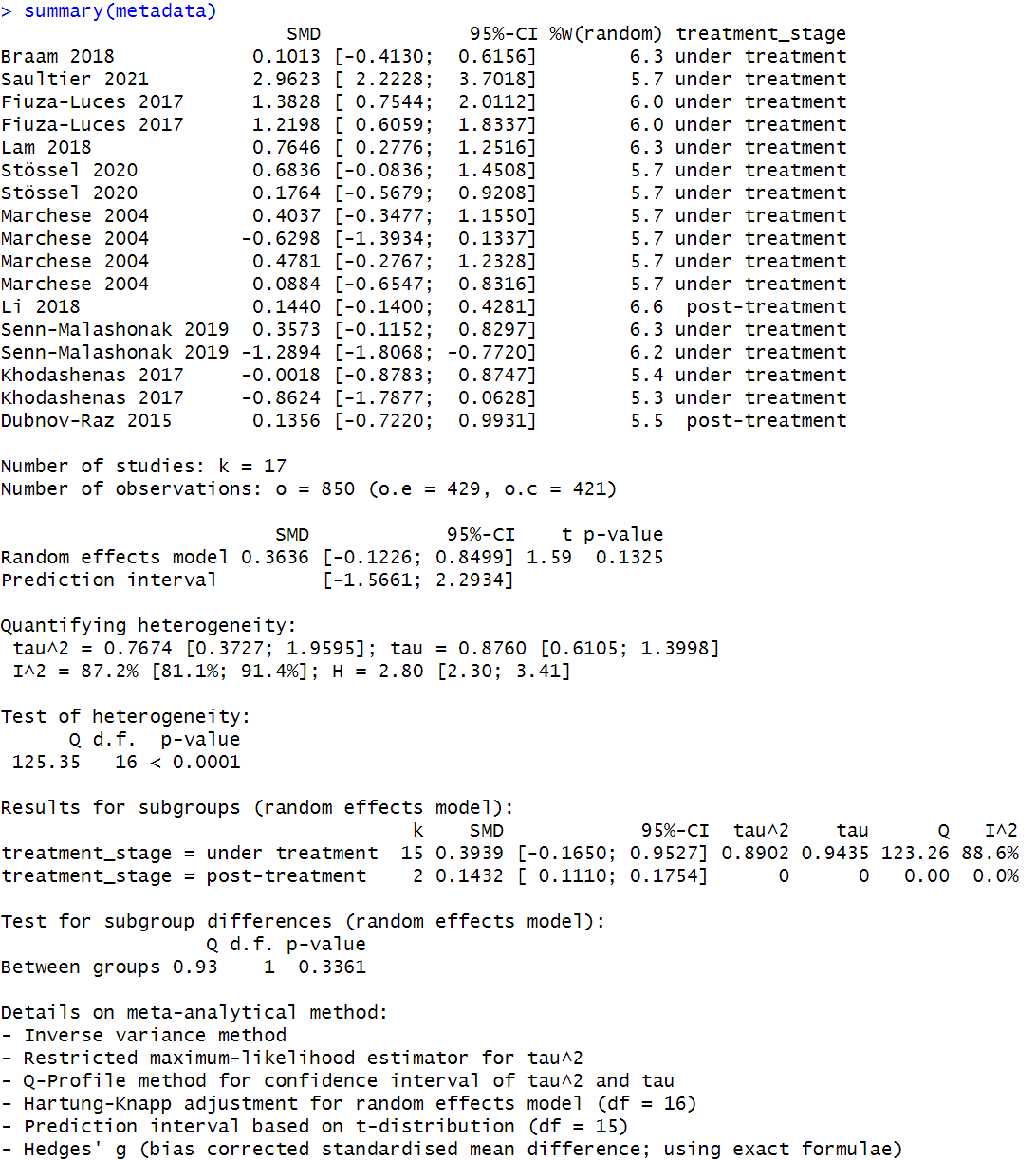
**

**
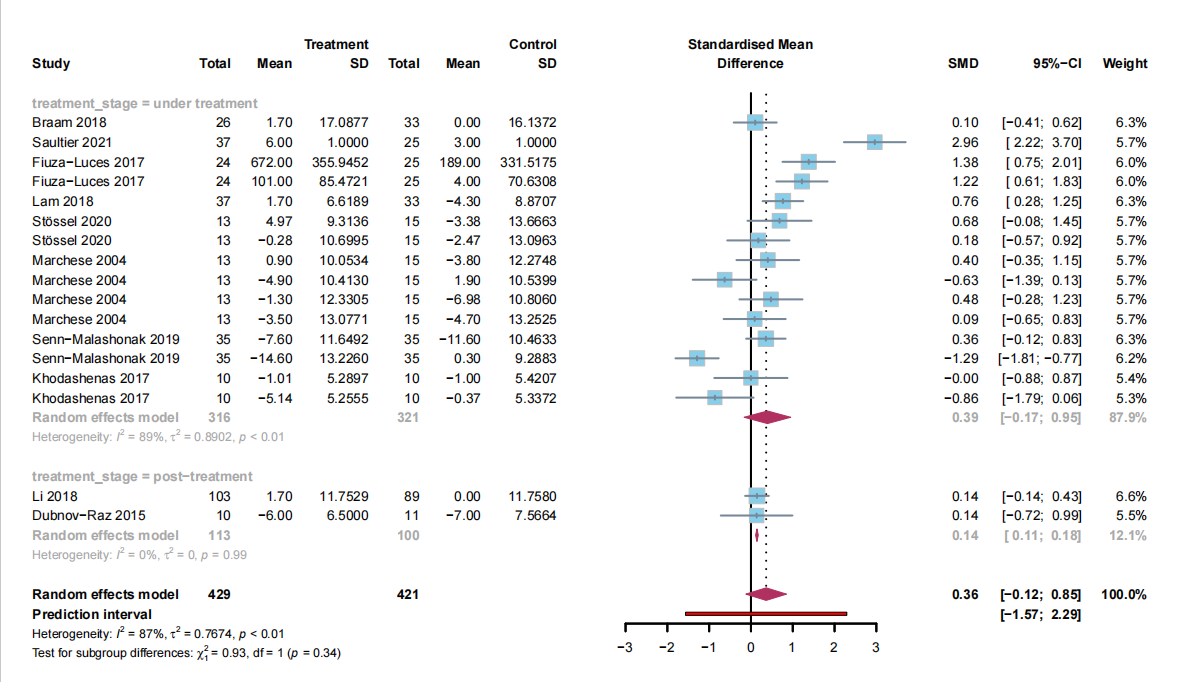
**

**13.5.2 Subgroup analysis based on treatment stage for fatigue**

**13.5.3 Subgroup analysis based on treatment stage for quality of life**

**13.5.4 Subgroup analysis based on treatment stage for lower body muscle strength**

**13.5.5 Subgroup analysis based on treatment stage for upper body muscle strength**

**13.5.6 Subgroup analysis based on treatment stage for trunk muscle strength**

**13.5.7 Subgroup analysis based on treatment stage for muscle strength**

**13.5.8 Subgroup analysis based on treatment stage for six-minute walk test**

**13.5.9 Subgroup analysis based on treatment stage for balance**

**13.5.10 Subgroup analysis based on treatment stage for flexibility**

**13.5.11 Subgroup analysis based on treatment stage for athletic performance**

**13.5.12 Subgroup analysis based on treatment stage for physical activity level**

**13.5.13 Subgroup analysis based on treatment stage for physical activity behaviour**

**13.5.14 Subgroup analysis based on treatment stage for peak oxygen uptake**

**13.5.15 Subgroup analysis based on treatment stage for cardiorespiratory function**

**13.5.16 Subgroup analysis based on treatment stage for bone mineral density**

**13.5.17 Subgroup analysis based on treatment stage for body mass index**

**13.5.18 Subgroup analysis based on treatment stage for fat mass percentage**

**13.5.19 Subgroup analysis based on treatment stage for NK cell level**

**13.5.20 Subgroup analysis based on treatment stage for depressive symptoms**

**13.5.21 Subgroup analysis based on treatment stage for social function**

**13.5.22 Subgroup analysis based on treatment stage for executive function**

**13.5.23 Subgroup analysis based on treatment stage for pro-inflammatory factors**

**13.5.24 Subgroup analysis based on treatment stage for anti-inflammatory factors**

**13.5.25 Subgroup analysis based on treatment stage for cognitive function**

**13.6 Sessions subgroup analysis**

**13.6.1 Subgroup analysis based on sessions for quality of life scale**

**13.6.2 Subgroup analysis based on sessions for fatigue**

**13.6.3 Subgroup analysis based on sessions for quality of life**

**13.6.4 Subgroup analysis based on sessions for lower body muscle strength**

**13.6.5 Subgroup analysis based on sessions for upper body muscle strength**

**13.6.6 Subgroup analysis based on sessions for trunk muscle strength**

**13.6.7 Subgroup analysis based on sessions for muscle strength**

**13.6.8 Subgroup analysis based on sessions for six-minute walk test**

**13.6.9 Subgroup analysis based on sessions for balance**

**13.6.10 Subgroup analysis based on sessions for flexibility**

**13.6.11 Subgroup analysis based on sessions for athletic performance**

**13.6.12 Subgroup analysis based on sessions for physical activity level**

**13.6.13 Subgroup analysis based on sessions for physical activity behaviour**

**13.6.14 Subgroup analysis based on sessions for peak oxygen uptake**

**13.6.15 Subgroup analysis based on sessions for cardiorespiratory function**

**13.6.16 Subgroup analysis based on sessions for bone mineral density**

**13.6.17 Subgroup analysis based on sessions for body mass index**

**13.6.18 Subgroup analysis based on sessions for fat mass percentage**

**13.6.19 Subgroup analysis based on sessions for NK cell level**

**13.6.20 Subgroup analysis based on sessions for depressive symptoms**

**13.6.21 Subgroup analysis based on sessions for social function**

**13.6.22 Subgroup analysis based on sessions for executive function**

**13.6.23 Subgroup analysis based on sessions for pro-inflammatory factors**

**13.6.24 Subgroup analysis based on sessions for anti-inflammatory factors**

**13.6.25 Subgroup analysis based on sessions for cognitive function**

**13.7 Length subgroup analysis**

**13.7.1 Subgroup analysis based on length for quality of life scale**

**13.7.2 Subgroup analysis based on length for fatigue**

**13.7.3 Subgroup analysis based on length for quality of life**

**13.7.4 Subgroup analysis based on length for lower body muscle strength**

**13.7.5 Subgroup analysis based on length for upper body muscle strength**

**13.7.6 Subgroup analysis based on length for trunk muscle strength**

**13.7.7 Subgroup analysis based on length for muscle strength**

**13.7.8 Subgroup analysis based on length for six-minute walk test**

**13.7.9 Subgroup analysis based on length for balance**

**13.7.10 Subgroup analysis based on length for flexibility**

**13.7.11 Subgroup analysis based on length for athletic performance**

**13.7.12 Subgroup analysis based on length for physical activity level**

**13.7.13 Subgroup analysis based on length for physical activity behaviour**

**13.7.14 Subgroup analysis based on length for peak oxygen uptake**

**13.7.15 Subgroup analysis based on length for cardiorespiratory function**

**13.7.16 Subgroup analysis based on length for bone mineral density**

**13.7.17 Subgroup analysis based on length for body mass index**

**13.7.18 Subgroup analysis based on length for fat mass percentage**

**13.7.19 Subgroup analysis based on length for NK cell level**

**13.7.20 Subgroup analysis based on length for depressive symptoms**

**13.7.21 Subgroup analysis based on length for social function**

**13.7.22 Subgroup analysis based on length for executive function**

**13.7.23 Subgroup analysis based on length for pro-inflammatory factors**

**13.7.24 Subgroup analysis based on length for anti-inflammatory factors**

**13.7.25 Subgroup analysis based on length for cognitive function**

**13.8 Sessions duration subgroup Analysis**

**13.8.1 Subgroup analysis based on sessions duration for quality of life scale**

**13.8.2 Subgroup analysis based on sessions duration for fatigue**

**13.8.3 Subgroup analysis based on sessions duration for quality of life**

**13.8.4 Subgroup analysis based on sessions duration for lower body muscle strength**

**13.8.5 Subgroup analysis based on sessions duration for upper body muscle strength**

**13.8.6 Subgroup analysis based on sessions duration for trunk muscle strength**

**13.8.7 Subgroup analysis based on sessions duration for muscle strength**

**13.8.8 Subgroup analysis based on sessions duration for six-minute walk test**

**13.8.9 Subgroup analysis based on sessions duration for balance**

**13.8.10 Subgroup analysis based on sessions duration for flexibility**

**13.8.11 Subgroup analysis based on sessions duration for athletic performance**

**13.8.12 Subgroup analysis based on sessions duration for physical activity level**

**13.8.13 Subgroup analysis based on sessions duration for physical activity behaviour**

**13.8.14 Subgroup analysis based on sessions duration for peak oxygen uptake**

**13.8.15 Subgroup analysis based on sessions duration for cardiorespiratory function**

**13.8.16 Subgroup analysis based on sessions duration for body mass index**

**13.8.17 Subgroup analysis based on sessions duration for fat mass percentage**

**13.8.18 Subgroup analysis based on sessions duration for NK cell level**

**13.8.19 Subgroup analysis based on sessions duration for depressive symptoms**

**13.8.20 Subgroup analysis based on sessions duration for social function**

**13.8.21 Subgroup analysis based on sessions duration for executive function**

**13.8.22 Subgroup analysis based on sessions duration for pro-inflammatory factors**

**13.8.23 Subgroup analysis based on sessions duration for anti-inflammatory factors**

**13.8.24 Subgroup analysis based on sessions duration for cognitive function**

**13.9 Exercise intensity subgroup Analysis**

**13.9.1 Subgroup analysis based on exercise intensity for quality of life scale**

**13.9.2 Subgroup analysis based on exercise for intensity fatigue**

**13.9.3 Subgroup analysis based on exercise intensity for quality of life**

**13.9.4 Subgroup analysis based on exercise intensity for lower body muscle strength**

**13.9.5 Subgroup analysis based on exercise intensity for upper body muscle strength**

**13.9.6 Subgroup analysis based on exercise intensity for trunk muscle strength**

**13.9.7 Subgroup analysis based on exercise intensity for muscle strength**

**13.9.8 Subgroup analysis based on exercise intensity for six-minute walk test**

**13.9.9 Subgroup analysis based on exercise intensity for balance**

**13.9.10 Subgroup analysis based on exercise intensity for flexibility**

**13.9.11 Subgroup analysis based on exercise intensity for athletic performance**

**13.9.12 Subgroup analysis based on exercise intensity for physical activity level**

**13.9.13 Subgroup analysis based on exercise intensity for physical activity behaviour**

**13.9.14 Subgroup analysis based on exercise intensity for peak oxygen uptake**

**13.9.15 Subgroup analysis based on exercise intensity for cardiorespiratory function**

**13.9.16 Subgroup analysis based on exercise intensity for bone mineral density**

**13.9.17 Subgroup analysis based on exercise intensity for body mass index**

**13.9.18 Subgroup analysis based on exercise intensity for fat mass percentage**

**13.9.19 Subgroup analysis based on exercise intensity for NK cell level**

**13.9.20 Subgroup analysis based on exercise intensity for depressive symptoms**

**13.9.21 Subgroup analysis based on exercise intensity for social function**

**13.9.22 Subgroup analysis based on exercise intensity for executive function**

**13.9.23 Subgroup analysis based on exercise intensity for pro-inflammatory factors**

**13.9.24 Subgroup analysis based on exercise intensity for anti-inflammatory factors**

**13.9.25 Subgroup analysis based on exercise intensity for cognitive function**

**14. Included literature**

1. Masoud AE, Shaheen AAM, Algabbani MF, et al. Effectiveness of exergaming in reducing cancer-related fatigue among children with acute lymphoblastic leukemia: a randomized controlled trial. Ann Med. 2023;55(1):2224048.
2. Braam KI, van Dijk-Lokkart EM, Kaspers GJL, et al. Effects of a combined physical and psychosocial training for children with cancer: a randomized controlled trial. BMC Cancer. 2018;18(1):1289.
3. Saultier P, Vallet C, Sotteau F, et al. A randomized trial of physical activity in children and adolescents with cancer. Cancers (Basel). 2021;13(1). doi:10.3390/cancers13010121
4. Ruble K, Scarvalone S, Gallicchio L, et al. Group physical activity intervention for childhood cancer survivors: a pilot study. J Phys Act Health. 2016;13(3):352-359.
5. Tanriverdi M, Cakir E, Akkoyunlu ME, et al. Effect of virtual reality-based exercise intervention on sleep quality in children with acute lymphoblastic leukemia and healthy siblings: a randomized controlled trial. Palliat Support Care. 2022;20(4):455-461.
6. Fiuza-Luces C, Padilla JR, Soares-Miranda L, et al. Exercise intervention in pediatric patients with solid tumors: the physical activity in pediatric cancer trial. Med Sci Sports Exerc. 2017;49(2):223-230.
7. Caru M, Dandekar S, Gordon B, et al. Implementing a behavioral physical activity program in children and adolescent survivors of childhood cancer: a pilot randomized controlled trial. J Behav Med. 2024;47(5):792-803.
8. Manchola-González JD, Bagur-Calafat C, Girabent-Farrés M, et al. Effects of a home-exercise program in childhood survivors of acute lymphoblastic leukemia on physical fitness and physical functioning: results of a randomized clinical trial. Support Care Cancer. 2020;28(7):3171-3178.
9. Lam KKW, Li WHC, Chung OK, et al. An integrated experiential training program with coaching to promote physical activity, and reduce fatigue among children with cancer: a randomized controlled trial. Patient Educ Couns. 2018;101(11):1947-1956.
10. Tanir MK, Kuguoglu S. Impact of exercise on lower activity levels in children with acute lymphoblastic leukemia: a randomized controlled trial from Turkey. Rehabil Nurs. 2013;38(1):48-59.
11. Stössel S, Neu MA, Wingerter A, et al. Benefits of exercise training for children and adolescents undergoing cancer treatment: results from the randomized controlled MUCKI trial. Front Pediatr. 2020;8:243.
12. Hartman A, te Winkel ML, van Beek RD, et al. A randomized trial investigating an exercise program to prevent reduction of bone mineral density and impairment of motor performance during treatment for childhood acute lymphoblastic leukemia. Pediatr Blood Cancer. 2009;53(1):64-71.
13. Müller C, Winter C, Boos J, et al. Effects of an exercise intervention on bone mass in pediatric bone tumor patients. Int J Sports Med. 2014;35(8):696-703.
14. Marchese VG, Chiarello LA, Lange BJ. Effects of physical therapy intervention for children with acute lymphoblastic leukemia. Pediatr Blood Cancer. 2004;42(2):127-133.
15. Yeh CH, Man Wai JP, Lin US, et al. A pilot study to examine the feasibility and effects of a home-based aerobic program on reducing fatigue in children with acute lymphoblastic leukemia. Cancer Nurs. 2011;34(1):3-12.
16. Chamorro-Viña C, Valentín J, Fernández L, et al. Influence of a moderate-intensity exercise program on early NK cell immune recovery in pediatric patients after reduced-intensity hematopoietic stem cell transplantation. Integr Cancer Ther. 2017;16(4):464-472.
17. Fiuza-Luces C, Padilla JR, Valentín J, et al. Effects of exercise on the immune function of pediatric patients with solid tumors: insights from the PAPEC randomized trial. Am J Phys Med Rehabil. 2017;96(11):831-837.
18. Li WHC, Ho KY, Lam KKW, et al. Adventure-based training to promote physical activity and reduce fatigue among childhood cancer survivors: a randomized controlled trial. Int J Nurs Stud. 2018;83:65-74.
19. Senn-Malashonak A, Wallek S, Schmidt K, et al. Psychophysical effects of an exercise therapy during pediatric stem cell transplantation: a randomized controlled trial. Bone Marrow Transplant. 2019;54(11):1827-1835.
20. Elnaggar RK, Osailan AM, Elbanna MF, et al. Effectiveness of a dose-graded aerobic exercise regimen on cardiopulmonary fitness and physical performance in pediatric survivors of acute lymphoblastic leukemia: a randomized clinical trial. J Cancer Surviv. 2025;19(3):1090-1101.
21. Khodashenas E, Badiee Z, Sohrabi M, et al. The effect of an aerobic exercise program on the quality of life in children with cancer. Turk J Pediatr. 2017;59(6):678-683.
22. Şahin S, Akel BS, Huri M, et al. Investigation of the effect of task-oriented rehabilitation program on motor skills of children with childhood cancer: a randomized-controlled trial. Int J Rehabil Res. 2020;43(2):167-174.
23. Dubnov-Raz G, Azar M, Reuveny R, et al. Changes in fitness are associated with changes in body composition and bone health in children after cancer. Acta Paediatr. 2015;104(10):1055-1061.
24. Waked I, Albenasy K. Bone mineral density, lean body mass, and bone biomarkers following physical exercise in children with acute lymphoblastic leukemia undergoing chemotherapy. Iran J Blood Cancer. 2018;10(3):69-75.

**15. PRISMA checklist**

**PRISMA 2020 Checklist**

| **Section and Topic** | **Item #** | **Checklist item** | **Location where item is reported** |
| --- | --- | --- | --- |
| **TITLE** | | |  |
| Title | 1 | Identify the report as a systematic review. | Page 1. Lines1-3 |
| **ABSTRACT** | | |  |
| Abstract | 2 | See the PRISMA 2020 for Abstracts checklist (Table 2). | Page 4. Lines49-83 |
| **INTRODUCTION** | | |  |
| Rationale | 3 | Describe the rationale for the review in the context of existing knowledge. | Page 6-7. Lines 86-119 |
| Objectives | 4 | Provide an explicit statement of the objective(s) or question(s) the review addresses. | Page 7-8. Lines120-134 |
| **METHODS** | | |  |
| Eligibility criteria | 5 | Specify the inclusion and exclusion criteria for the review and how studies were grouped for the syntheses. | Page 8-9. Lines146-161 |
| Information sources | 6 | Specify all databases, registers, websites, organisations, reference lists and other sources searched or consulted to identify studies. Specify the date when each source was last searched or consulted. | Page 8. Lines138-144 |
| Search strategy | 7 | Present the full search strategies for all databases, registers and websites, including any filters and limits used. | Page 8. Lines145 |
| Selection process | 8 | Specify the methods used to decide whether a study met the inclusion criteria of the review, including how many reviewers screened each record and each report retrieved, whether they worked independently, and if applicable, details of automation tools used in the process. | Page 9. Lines164-165 |
| Data collection process | 9 | Specify the methods used to collect data from reports, including how many reviewers collected data from each report, whether they worked independently, any processes for obtaining or confirming data from study investigators, and if applicable, details of automation tools used in the process. | Page 9 Lines165-168 |
| Data items | 10a | List and define all outcomes for which data were sought. Specify whether all results that were compatible with each outcome domain in each study were sought (e.g. for all measures, time points, analyses), and if not, the methods used to decide which results to collect. | Page 9. Lines157-161 |
|  | 10b | List and define all other variables for which data were sought (e.g. participant and intervention characteristics, funding sources). Describe any assumptions made about any missing or unclear information. | Page 9. Lines157-161 |
| Study risk of bias assessment | 11 | Specify the methods used to assess risk of bias in the included studies, including details of the tool(s) used, how many reviewers assessed each study and whether they worked independently, and if applicable, details of automation tools used in the process. | Page 9. Lines165-168 |
| Effect measures | 12 | Specify for each outcome the effect measure(s) (e.g. risk ratio, mean difference) used in the synthesis or presentation of results. | Page 9-10. Lines171-180 |
| Synthesis methods | 13a | Describe the processes used to decide which studies were eligible for each synthesis (e.g. tabulating the study intervention characteristics and comparing against the planned groups for each synthesis (item #5)). | Page 10-11. Lines191-200 |
|  | 13b | Describe any methods required to prepare the data for presentation or synthesis, such as handling of missing summary statistics, or data conversions. | Page 10-11. Lines191-200 |
|  | 13c | Describe any methods used to tabulate or visually display results of individual studies and syntheses. | Page 10-11. Lines191-200 |
|  | 13d | Describe any methods used to synthesize results and provide a rationale for the choice(s). If meta-analysis was performed, describe the model(s), method(s) to identify the presence and extent of statistical heterogeneity, and software package(s) used. | Page 10-11. Lines191-200 |
|  | 13e | Describe any methods used to explore possible causes of heterogeneity among study results (e.g. subgroup analysis, meta-regression). | Page 10-11. Lines191-200 |
|  | 13f | Describe any sensitivity analyses conducted to assess robustness of the synthesized results. | Page 10-11. Lines191-200 |
| Reporting bias assessment | 14 | Describe any methods used to assess risk of bias due to missing results in a synthesis (arising from reporting biases). | Page 10. Lines175-176 |
| Certainty assessment | 15 | Describe any methods used to assess certainty (or confidence) in the body of evidence for an outcome. | Page 11. Lines201-207 |
| **RESULTS** | | |  |
| Study selection | 16a | Describe the results of the search and selection process, from the number of records identified in the search to the number of studies included in the review, ideally using a flow diagram. | Page 11-13. Lines211-222 |
|  | 16b | Cite studies that might appear to meet the inclusion criteria, but which were excluded, and explain why they were excluded. | Page 11-13. Lines211-222 |
| Study characteristics | 17 | Cite each included study and present its characteristics. | Page 11-13. Lines211-222 |
| Risk of bias in studies | 18 | Present assessments of risk of bias for each included study. | Page 14. Lines262-269 |
| Results of individual studies | 19 | For all outcomes, present, for each study: (a) summary statistics for each group (where appropriate) and (b) an effect estimate and its precision (e.g. confidence/credible interval), ideally using structured tables or plots. | Page 12-13. Lines225-253 |
| Results of syntheses | 20a | For each synthesis, briefly summarise the characteristics and risk of bias among contributing studies. | Page 14. Lines262-269 |
|  | 20b | Present results of all statistical syntheses conducted. If meta-analysis was done, present for each the summary estimate and its precision (e.g. confidence/credible interval) and measures of statistical heterogeneity. If comparing groups, describe the direction of the effect. | Page 14. Lines262-269 |
|  | 20c | Present results of all investigations of possible causes of heterogeneity among study results. | Page 14. Lines262-269 |
|  | 20d | Present results of all sensitivity analyses conducted to assess the robustness of the synthesized results. | Page 14. Lines262-269 |
| Reporting biases | 21 | Present assessments of risk of bias due to missing results (arising from reporting biases) for each synthesis assessed. | Page 14. Lines262-269 |
| Certainty of evidence | 22 | Present assessments of certainty (or confidence) in the body of evidence for each outcome assessed. | Page 14. Lines262-269 |
| **DISCUSSION** | | |  |
| Discussion | 23a | Provide a general interpretation of the results in the context of other evidence. | Page 14. Lines273-338 |
|  | 23b | Discuss any limitations of the evidence included in the review. | Page 17-18 Lines340-351 |
|  | 23c | Discuss any limitations of the review processes used. | Page 17-18 Lines340-351 |
|  | 23d | Discuss implications of the results for practice, policy, and future research. | Page 18. Lines354-358 |
| **OTHER INFORMATION** | | |  |
| Registration and protocol | 24a | Provide registration information for the review, including register name and registration number, or state that the review was not registered. | Page 9. Lines167-168 |
|  | 24b | Indicate where the review protocol can be accessed, or state that a protocol was not prepared. | NA |
|  | 24c | Describe and explain any amendments to information provided at registration or in the protocol. | NA |
| Support | 25 | Describe sources of financial or non-financial support for the review, and the role of the funders or sponsors in the review. | Page 19 Lines385-388 |
| Competing interests | 26 | Declare any competing interests of review authors. | Page 19 Lines381-382 |
| Availability of data, code and other materials | 27 | Report which of the following are publicly available and where they can be found: template data collection forms; data extracted from included studies; data used for all analyses; analytic code; any other materials used in the review. | Page 10. Lines374-375 |

**PRISMA 2020 for Abstracts checklist**

| **Section and Topic** | **Item #** | **Checklist item** | **Reported (Yes/No)** |
| --- | --- | --- | --- |
| **TITLE** | | |  |
| Title | 1 | Identify the report as a systematic review. | Yes |
| **BACKGROUND** | | |  |
| Objectives | 2 | Provide an explicit statement of the main objective(s) or question(s) the review addresses. | Yes |
| **METHODS** | | |  |
| Eligibility criteria | 3 | Specify the inclusion and exclusion criteria for the review. | Yes |
| Information sources | 4 | Specify the information sources (e.g. databases, registers) used to identify studies and the date when each was last searched. | Yes |
| Risk of bias | 5 | Specify the methods used to assess risk of bias in the included studies. | Yes |
| Synthesis of results | 6 | Specify the methods used to present and synthesise results. | Yes |
| **RESULTS** | | |  |
| Included studies | 7 | Give the total number of included studies and participants and summarise relevant characteristics of studies. | Yes |
| Synthesis of results | 8 | Present results for main outcomes, preferably indicating the number of included studies and participants for each. If meta-analysis was done, report the summary estimate and confidence/credible interval. If comparing groups, indicate the direction of the effect (i.e. which group is favoured). | Yes |
| **DISCUSSION** | | |  |
| Limitations of evidence | 9 | Provide a brief summary of the limitations of the evidence included in the review (e.g. study risk of bias, inconsistency and imprecision). | Yes |
| Interpretation | 10 | Provide a general interpretation of the results and important implications. | Yes |
| **OTHER** | | |  |
| Funding | 11 | Specify the primary source of funding for the review. | Yes |
| Registration | 12 | Provide the register name and registration number. | Yes |

**16. Reference**

1. Guyatt GH, Oxman AD, Vist GE, et al. GRADE: an emerging consensus on rating quality of evidence and strength of recommendations. *Bmj*. Apr 26 2008;336(7650):924-6. doi:10.1136/bmj.39489.470347.AD

2. Guyatt G, Oxman AD, Akl EA, et al. GRADE guidelines: 1. Introduction-GRADE evidence profiles and summary of findings tables. *J Clin Epidemiol*. Apr 2011;64(4):383-94. doi:10.1016/j.jclinepi.2010.04.026

3. Schünemann. H, Brożek. J, Guyatt. G, Oxma. A. Handbook for Grading the Quality of Evidence And the Strength of Recommendation Using the GRADE Approach. <https://gdt.gradepro.org/app/handbook/handbook.html>

4. Sterne JAC, Savović J, Page MJ, et al. RoB 2: a revised tool for assessing risk of bias in randomised trials. *BMJ*. Aug 28 2019;366:l4898. doi:10.1136/bmj.l4898

5. Murad MH, Almasri J, Alsawas M, Farah W. Grading the quality of evidence in complex interventions: a guide for evidence-based practitioners. *Evid Based Med*. Mar 2017;22(1):20-22. doi:10.1136/ebmed-2016-110577
